# Analyses of open-access multi-omics data sets reveal genetic and expression characteristics of maize *ZmCCT* family genes

**DOI:** 10.1093/aobpla/plab048

**Published:** 2021-08-16

**Authors:** Ming-You Dong, Ling Lei, Xian-Wei Fan, You-Zhi Li

**Affiliations:** State Key Laboratory for Conservation and Utilization of Subtropical Agro-bioresources/College of Life Science and Technology, Guangxi University, 100 Daxue Road, Nanning, Guangxi 530004, P. R. China

**Keywords:** Circadian clock rhythm, flowering, maize, photoperiod response

## Abstract

Flowering in maize (*Zea mays*) is influenced by photoperiod. The CO, CO-like/COL and TOC1 (CCT) domain protein-encoding genes in maize, *ZmCCT*s, are particularly important for photoperiod sensitivity. However, little is known about CCT protein-encoding gene number across plant species or among maize inbred lines. Therefore, we analysed CCT protein-encoding gene number across plant species, and characterized *ZmCCT*s in different inbred lines, including structural variations (SVs), copy number variations (CNVs), expression under stresses, dark-dark (DD) and dark-light (DL) cycles, interaction network and associations with maize quantitative trait loci (QTLs) by referring to the latest v4 genome data of B73. Gene number varied greatly across plant species, more in polyploids than in diploids. The numbers of *ZmCCT*s identified were 58 in B73, 59 in W22, 48 in Mo17, and 57 in Huangzao4 for temperate maize inbred lines, and 68 in tropical maize inbred line SK. Some *ZmCCT*s underwent duplications and presented chromosome collinearity. Structural variations and CNVs were found but they had no germplasm specificity. Forty-two *ZmCCT*s responded to stresses. Expression of 37 *ZmCCT*s in embryonic leaves during seed germination of maize under DD and DL cycles was roughly divided into five patterns of uphill pattern, downhill-pattern, zigzag-pattern, └-pattern and ⅃-pattern, indicating some of them have a potential to perceive dark and/or dark-light transition. Thirty-three *ZmCCT*s were co-expressed with 218 other maize genes; and 24 *ZmCCT*s were associated with known QTLs. The data presented in this study will help inform further functions of *ZmCCT*s.

## Teaser

Flowering in maize (*Zea mays*) is influenced by photoperiod and related to ZmCCT genes. ZmCCTs vary in structural variations and expression under stress, and dark-light and dark-dark cycles; and in copy number (58 in B73, 59 in W22, 48 in Mo17 and 57 in Huangzao4 for temperate maize inbred lines, and 68 in tropical maize inbred line SK). Some ZmCCTs have the potential to perceive dark and/or dark-light transition. Some ZmCCTs are associated with some important QTLs in B73. These demonstrate the importance of ZmCCTs in evolution and domestication as well as in the photoperiod adaptation of maize.

## Introduction

Photoperiod affects flowering in plants including maize (*Zea mays*) (Jackson 2009; Song *et al.* 2015). Modern maize gradually evolved two major types of germplasm after domestication of a tropical teosinte from Mexico and Central America: (i) tropically/subtropically adapted and photoperiod-sensitive, and (ii) temperate-adapted and less photoperiod-sensitive (Doebley 2004; Hung *et al.* 2012). Temperate maize is an autonomous day-neutral plant, and teosinte is an obligate short-day plant that requires uninterrupted long nights to induce flowering (Minow *et al.* 2018). The differences in photoperiod sensitivity are observed in and between maize populations (Jiang *et al.* 1999; Huang *et al.* 2018). Photoperiod sensitivity hinders the improvement of temperate maize through the utilization of subtropical/tropical maize germplasm (Lewis and Goodman 2003; Yamasaki *et al.* 2007; Wang *et al.* 2008).

Growing evidence indicate that many key genes in photoperiodic flowering-time regulatory pathways are conserved across diverse plant species, but unique regulatory pathways are also present in some phylogenetic groups (Coles *et al.* 2010). Maize response to photoperiod is affected only by few of the flowering-time quantitative trait loci (QTLs) (Yang *et al.* 2013). The photoperiod-sensing pathway consists of the conserved upstream genes of *conz1* as the closest homolog of *CONSTANS* (*CO*) in *Arabidopsis*, *gigz1A*, *gigz1B*, *id1*, and the differential downstream *FLOWERING LOCUS T* (*FT*)-like genes such as *ZCN8*, of which the upstream gene components are conserved in maize (Miller *et al.* 2008).

The proteins containing the CO, CO-like/COL and TOC1 (CCT) domain in the C terminus are transcription factors involved in photoperiod sensitivity of plants (Liu *et al.* 2020). The CCT domain-containing proteins were usually divided into three clades including COL, PSEUDO RESPONSE REGULATOR (PRR)-like and CCT MOTIF FAMILY (CMF)-like proteins on the basis of the domains in the N terminus (Liu *et al.* 2020). Many transcription factors related to photoperiod sensitivity in flowering plants contain a CCT domain in their predicted sequence (Liu *et al.* 2020).

The maize CCT domain-containing protein (ZmCCT) genes, *ZmCCT*s, appear to be particularly important for the photoperiod sensitivity of maize (Ducrocq *et al.* 2009; Yang *et al.* 2013) because their consistent and high expression results in the delay in flowering of maize under long day (Hung *et al.* 2012). It was reported that the *ZmCCT* family in maize inbred line B73 contains 53 *ZmCCT* genes based on the version two (v2) B73 genome, with four clades, including COL, PRR-like, CMF-like and TIF[F/Y]XG (TIFY)-like proteins (Jin *et al.* 2018). *ZmCCT*s are the upstream genes of *ZCN8* in the photoperiod pathway, and they repress *ZCN8* expression (Dong *et al.* 2012; Huang *et al.* 2018). A few *ZmCCT*s were functionally identified, of which *ZmCCT9* and *ZmCCT10*, and *ZmCOL3* contribute to flowering-time adaptation of maize from tropical to temperate regions (Yang *et al* 2013; Huang *et al.* 2018; Jin *et al.* 2018). In addition, *ZmCCT*s may also be involved in growth, development, and stress response (Ku *et al.* 2016; Li *et al.* 2016, 2017; Wang *et al.* 2017; Xu *et al.* 2017; Zhang *et al.* 2018). There is a report in which the CCT protein number of numerous genomes was previously informed, but little is yet known about CCT protein-encoding gene number across plant species or the genetic characteristics of *ZmCCT*s among maize inbred lines. We speculate that the CCT protein gene number varies with plant species, and that the expression patterns of *ZmCCT*s in maize are different under continuous dark-dark (DD) versus dark-light (DL) cycles. In this study, we analyse the number of CCT protein-encoding genes of plants and disclose characteristics of *ZmCCT*s including structural variations (SVs); copy number variations (CNVs); expression under stresses, DD and DL cycles; interaction network; and associations with maize QTLs by referring to the latest v4 genome data of B73.

## Materials and Methods

### Analysis of CCT domain-containing proteins and CCT protein-encoding genes

The identification of CCT domain-containing proteins was based on the search of protein data sets using the PF06203CCT hidden Markov model (HMM) (http://pfam.xfam.org/). The protein data sets were those from temperate maize inbred lines B73, Mo17 and W22 (https://maizegdb.org/), temperate maize inbred line Huangzao4 under accession number PRJCA001247 and the tropical small-kernel maize inbred line SK under accession number PRJNA531547 (http://bigd.big.ac.cn/gsa), and phytozome database (https://phytozome.jgi.doe.gov/). The search was conducted using the HMMER3 tool (Finn *et al.* 2011; http://www.ebi.ac.uk/Tools/hmmer/) according to 1 expect threshold (E) < 10^−5^.

Second, to prevent from losing potential CCT proteins in the above HMM-based search, *Arabidopsis* CCT proteins from the *Arabidopsis* Information Resource database (http://www.arabidopsis.org/), and rice CCT proteins from the Rice Genome Annotation database (http://rice.plantbiology.msu.edu/) were used to search the above proteomes from maize and proteomes from other plants through the basic local alignment search tool for proteins (BLASTp) according to 1E < 10^−5^. After removing redundant sequences, CCT proteins were further confirmed by searching the conserved domain database with a threshold of 0.01 and a maximum hit of 500 (Marchler-Bauer *et al.* 2017) and the Pfam database under 1E < 0.05 (Finn *et al.* 2016).

Third, the candidate protein identification (ID) number of maize were used to search and obtain the corresponding gene ID number in the maizegdb database (https://maizegdb.org/) for maize *ZmCCT*s and in the phytozome database (https://phytozome.jgi.doe.gov/pz/portal.html) for CCT protein-encoding genes of other plants. When there were multiple candidate proteins produced by different transcripts of the same gene, the protein with the longest amino acid sequence was selected as the representative to search the corresponding gene ID number.

### Construction of phylogenetic trees of plant species

With the above information, the v0.66839 Toolkit for Biologists integrating various biological data-handling tools (TBtools) was used to generate the Newick (nwk) files with Latin names of species as input files according to the previous methods under the default parameters (Chen *et al.* 2018). The nwk files were employed to construct phylogenetic trees using the v6 Molecular Evolutionary Genetics Analysis software under the default parameters (Tamura *et al.* 2013).

The polyploidy events, such as whole-genome triplication (WGT), whole-genome duplication (WGD) and whole-genome sextuplication (WGS), of plant species came from research in Qu *et al.* (2018).

### Analysis of chromosomal localization, collinearity and duplication time of *ZmCCT*s

The multiple sequence alignment was performed with the target proteins against the v4 B73 proteome data set (https://maizegdb.org/) by using the BLASTp to generate an m8-format file under 1E < 10^−5^. The chromosome collinearity of the genes was analysed based on both the m8-format file and the General Feature Format (GFF) file of the v4 B73 genome by using the toolkit for collinearity detection based on an adjusted MCScan (Wang *et al.* 2012). Duplicate gene pairs were extracted from the collinear genes with gene’s ID number. The collinearity and chromosome localization were plotted using the Amazing Simple Circos tool (Chen *et al.* 2018).

The non-synonymous substitution (*K*_a_) and synonymous substitution (*K*_s_) rates of the duplicate gene pairs were calculated using the Parallel Alignment and back-Translation tool (Zhang *et al.* 2012) and *K*_a_*K*_s__Calculator 2.0 (Wang *et al.* 2010). The gene duplication time was estimated according to the formula: *K*_s_/2*λ*, where *λ* = 6.5 × 10^−9^ (Koch *et al.* 2000).

### Analysis of characteristics and domains/motifs of *ZmCCT*-encoded proteins ZmCCTs, as well as introns and exons of *ZmCCT*s

The molecular weight and isoelectric point of each protein were analysed according to Artimo *et al.* (2012), and subcellular localization was predicted as Yu *et al.* (2006). The amino acid motifs were predicted (Bailey *et al.* 2015) and annotated through the InterProScan database (Mitchell *et al.* 2019). The protein domains were predicted according to Letunic and Bork (2017). The introns, exons and the domains/motifs in the genes were mapped based on the GFF files of the genes following the methods in Chen *et al.* (2018).

### Analysis of SVs and CNVs of *ZmCCT*s

Structural variation analysis of the *ZmCCT*s was based on the maize inbred line B73 v4 genome SV loci data, which were generated by Yang *et al.* (2019) against the SV data set of the maize inbred line SK genome. In brief, with the SV loci of *ZmCCT*s in B73 as reference, the SVs of the *ZmCCT*s in the genomes of other 521 maize inbred lines (http://maizego.org/Resources.html) were scanned according to the methods described by Yang *et al.* (2019).

For CNV analysis, the maize genome re-sequencing data sets with a depth of > 30×, including 17 tropical/subtropical maize inbred lines and 24 temperate maize inbred lines (**see****Supporting Information—Table S1**; https://www.ncbi.nlm.nih.gov/sra/), were first subjected to quality control (Bolger *et al.* 2014). Afterwards, the genome sequences were aligned to the v4 B73 genome by using the V0.7.15 Burrows-Wheeler Alignment software (Li and Durbin 2009), where the parameters used were under threads of 4, a minSeedLen of 32 and mark shorter split hits as secondary (Li and Durbin 2009). The sequences were converted into Binary Alignment/Map (BAM) format files using the Sequence Alignment/Map tools (Li *et al.* 2009). The BAM-format files were used to identify CNVs in other maize lines by using the Picard 1.129 software (http://sourceforge.net/projects/picard/) and the cnvnator 0.3.2 software under 1E < 0.01 (Abyzov *et al.* 2011).

### Expression analysis of *ZmCCT*s

The raw data sets used were the transcriptomes of embryonic leaves during seed germination of *Z*. *mays* cv. White Crystal under DL [dark (11 h)/light (13 h)] cycles (Liu *et al.* 2013) and DD (Chang *et al.* 2019), and the transcriptomes of abiotic (Makarevitch *et al.* 2015; Zenda *et al.* 2019; http://childslab.plantbiology.msu.edu) and biotic stress-treated maize lines (Swart *et al.* 2017). These raw data had three biological replicates.

The gene expression in the embryonic leaves was presented as Fragments Per Kilobase of transcript per Million-fragments mapped (Trapnell *et al.* 2010). For analysis of gene expression in the tissues under stresses, the reads of the *ZmCCT* sequences in the Sequence Read Archive (SRA) file (https://www.ncbi.nlm.nih.gov/sra/) were transformed into FASTQ format by using the SRA toolkit 2.9.2 tool, subjected to quality control by the FastQC 0.11.8 and Trimmomatic 0.38 tools, and then aligned to the bowtie 2-indexed v4 B73 genome (https://maizegdb.org/) by using the TopHat 2.1.1 software (Trapnell *et al.* 2012) to generate the BAM-format files. The reads of *ZmCCT*s were counted by using the featureCounts tool (Liao *et al.* 2014) and then used for differential expression analysis by the OmicShare tools (www.omicshare.com/tools) under the default parameters (Robinson *et al.* 2010). Differentially expressed genes were defined according to |a log2 fold change of read counts| ≥ 1 at *P* < 0.05.

The gene expression heat maps were plotted by using the Amazing Simple HeatMap tool in TBtools (Chen *et al.* 2018).

### Analysis of association of *ZmCCT*s with QTLs

The QTLs associated with *ZmCCT*s were identified through searching the B73 data set (https://bigd.big.ac.cn/gwas/) in the genome-wide association study (GWAS) Atlas (Tian *et al.* 2020) by ID number of *ZmCCT*s.

### Analysis of gene co-expression and gene ontology enrichment

The genes co-expressed with *ZmCCT*s were achieved by searching the maize co-expression data set in the *Arabidopsis thaliana trans*-factor and *cis*-element prediction database-II (ATTED-II) (Obayashi *et al.* 2018; http://atted.jp/) with ID number of *ZmCCT*s. The co-expression networks of the genes were constructed by using the Cytoscape software (Shannon *et al.* 2003). Gene ontology (GO) enrichment was performed using the OmicShare tools under default parameters with a false discovery rate (*Q*) < 0.05 (www.omicshare.com/tools).

## Results

### CCT protein-encoding genes and CCT proteins in plants

**Figure 1.**
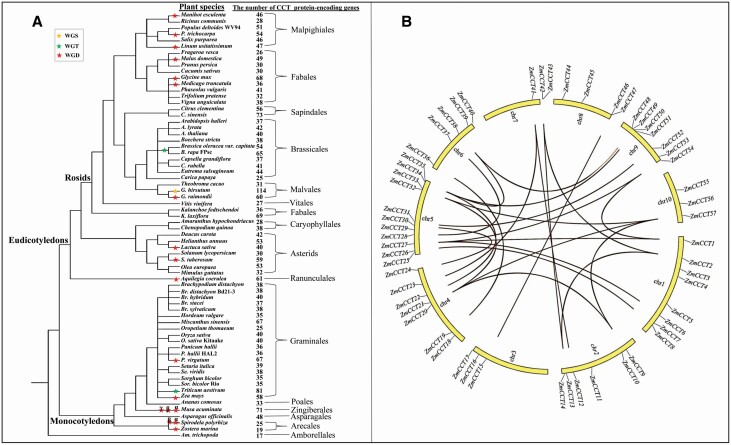
The number and polyploidy events of the CCT protein genes in plants (A), and chromosomal locations and collinearity (B) of *ZmCCT*s except *ZmCCT58* in the v4 B73 genome. Panels A and B just showed *ZmCCT* number in maize inbred line B73, and polyploidy events marked with red stars representing WGD, green stars indicating WGT and a faint yellow star referring to WGS. *α* (duplication), *β* (tetraploidization) and *γ* (hexaploidization) represent evolutionary shuffling events in the ancestral lineages. The polyploidy events were quoted from research in Qu *et al.* (2018). v4, version 4; WGD, whole-genome duplication; WGS, whole-genome sextuplication; WGT, whole-genome triplication; *ZmCCT*, ZmCCT gene.

Based on the CCT HMM model, the number of CCT protein-encoding genes was found to differ among 68 plant species, with up to 114 genes in *Gossypium hirsutum* and only 17 genes in *Amborella trichopoda* (Fig. 1A). The ID number of CCT protein-encoding genes is listed in **Supporting Information—Table S2**.

The temperate maize lines of B73, Huangzao 4, W22 and Mo17 had 58, 37, 59 and 48 *ZmCCT*s **[see****Supporting Information—Table S3****]**, respectively. The tropical maize line SK (Yang *et al.* 2019) possessed 68 *ZmCCT*s. The ID number of the *ZmCCT*s is shown in **Supporting Information—Table S3**. The ZmCCTs had high hydrophilicity because of the negative overall average of hydropathicity, and they differed in amino acid sequence length, molecular weight, isoelectric point, and in subcellular locations **[see****Supporting Information—Table S4****]**.

### Chromosome distribution and duplication, collinearity and genomic structure of *ZmCCT*s in B73

**Figure 2.**
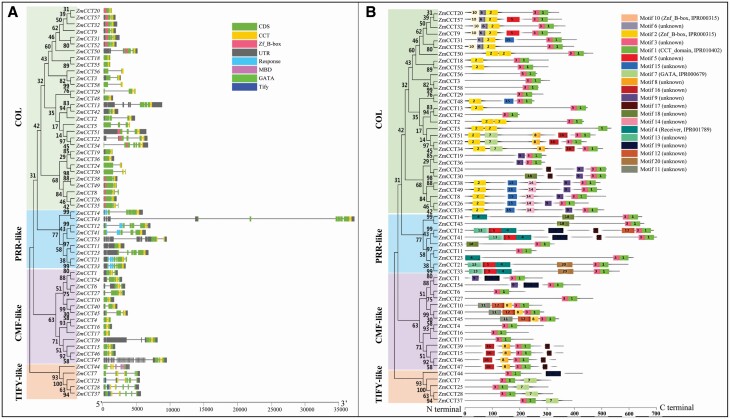
The clades of *ZmCCT*s (A), and the conserved domains/motifs (A) of the predicative ZmCCTs in B73. COL, PRR-like, TIFY-like and CMF-like were four clades of *ZmCCT*s. The figures in the structures in panel (B) referred to motif numbers. ZmCCT, Maize CCT domain-containing protein; *ZmCCT*, ZmCCT gene.

Except for *ZmCCT58*, which was located on the unassembled genome scaffold, the remaining 57 *ZmCCT*s were mapped to 10 chromosomes of B73 (Fig. 1B). A total of 26 pairs of the genes with segment duplication (Table 1) showed collinear relationships (Fig. 1B). Duplication happened between 1.1 MYA (million years ago) for the *ZmCCT*s *28* and *37* pair and 38.947 MYA for both *ZmCCT9* and *ZmCCT20* (Table 1). *ZmCCT*s showed easily noticeable differences in genomic DNA structure in B73 genome (Fig. 2A). The number of introns in *ZmCCT*s of B73 inbred line varied greatly, with no introns in *ZmCCT55***[see****Supporting Information—Table S4****]**.

**Table 1.** Duplications of *ZmCCT*s in maize inbred line B73 according to v4 of the B73 genome. *K*_a_, non-synonymous substitution rate; *K*_s_, synonymous substitution rate; MYA, million years ago; v4, version 4; *ZmCCT*, ZmCCT gene.

Duplicate pair		K_a_	K_s_	*K*_a_/*K*_s_	Estimated time (MYA)	Duplication type
*ZmCCT2*	*ZmCCT13*	0.406	2.917	0.139	22.441	Segmental
*ZmCCT6*	*ZmCCT27*	0.108	0.788	0.137	6.063	Segmental
*ZmCCT1*	*ZmCCT27*	0.406	3.176	0.128	24.429	Segmental
*ZmCCT8*	*ZmCCT26*	0.067	0.427	0.156	3.284	Segmental
*ZmCCT2*	*ZmCCT42*	0.483	2.852	0.169	21.937	Segmental
*ZmCCT1*	*ZmCCT54*	0.086	0.238	0.362	1.833	Segmental
*ZmCCT57*	*ZmCCT9*	0.030	0.242	0.126	1.864	Segmental
*ZmCCT57*	*ZmCCT20*	0.189	4.627	0.041	35.591	Segmental
*ZmCCT57*	*ZmCCT32*	0.173	2.067	0.083	15.897	Segmental
*ZmCCT9*	*ZmCCT20*	0.176	5.063	0.035	38.947	Segmental
*ZmCCT12*	*ZmCCT41*	0.071	0.146	0.486	1.124	Segmental
*ZmCCT13*	*ZmCCT42*	0.107	0.330	0.323	2.542	Segmental
*ZmCCT15*	*ZmCCT46*	0.081	0.445	0.183	3.420	Segmental
*ZmCCT20*	*ZmCCT32*	0.064	0.515	0.125	3.959	Segmental
*ZmCCT21*	*ZmCCT33*	0.061	0.224	0.271	1.726	Segmental
*ZmCCT22*	*ZmCCT34*	0.095	0.288	0.332	2.212	Segmental
*ZmCCT24*	*ZmCCT30*	0.086	0.402	0.215	3.089	Segmental
*ZmCCT19*	*ZmCCT36*	0.164	0.592	0.277	4.555	Segmental
*ZmCCT23*	*ZmCCT41*	0.640	2.468	0.259	18.985	Segmental
*ZmCCT35*	*ZmCCT38*	0.269	2.412	0.112	18.553	Segmental
*ZmCCT28*	*ZmCCT37*	0.047	0.143	0.332	1.100	Segmental
*ZmCCT31*	*ZmCCT52*	0.203	2.627	0.077	20.210	Segmental
*ZmCCT27*	*ZmCCT54*	0.403	2.887	0.139	22.209	Segmental
*ZmCCT34*	*ZmCCT51*	0.247	2.233	0.111	17.179	Segmental
*ZmCCT35*	*ZmCCT49*	0.292	2.275	0.129	17.500	Segmental
*ZmCCT38*	*ZmCCT49*	0.088	0.447	0.197	3.435	Segmental

### Phylogenetic relationships and conserved domains/motifs of ZmCCTs

As previously reported (Jin *et al.* 2018), ZmCCTs were divided into four clades including COL, PRR-like, TIFY-like and CMF-like proteins. In addition to the CCT domain, there were the Zf-B (zinc finger B-box) domain in the COL clade, the pseudoresponse domain in the PRR-like clade, and the methyl CpG binding domain (MBD) and the GATA domain in the TIFY-like clade (Fig. 2B; **see****Supporting Information—Table S5**). In addition to the above domains, ZmCCTs had many conserved amino acid motifs (Fig. 2B; **see****Supporting Information—Table S5**). The motif 3 existed in 57 ZmCCTs but not in ZmCCT5 (Fig. 2B).

### CNVs of *ZmCCT*s

**Figure 3.**
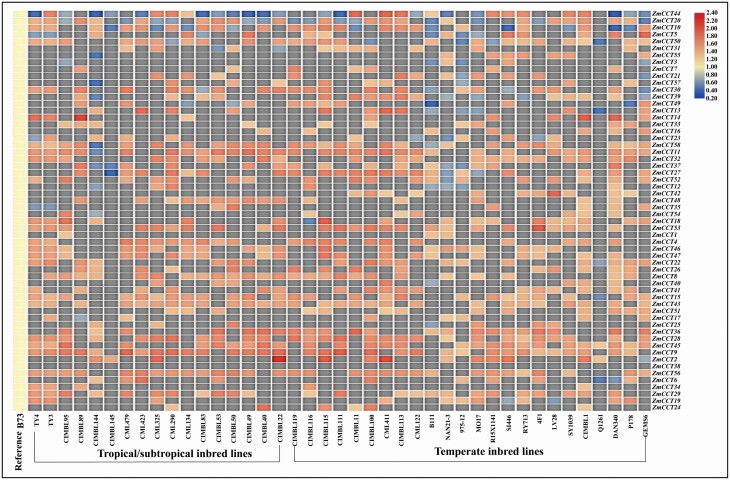
The CNVs of *ZmCCT*s in maize inbred lines with the v4 genome of the inbred line B73 as reference. The CNVs of *ZmCCT*s were analysed based on the genome data set in the literature (Yang *et al.* 2019). CNV, copy number variation; v4, version 4; *ZmCCT*, ZmCCT gene.

With the v4 B73 genome as reference, CNVs of *ZmCCT*s in 41 maize lines (17 tropical/subtropical and 24 temperate lines) were analysed (Fig. 3). The copy number of *ZmCCT*s *8*, *9*, *11*, *15*, *28*, *43*, *45*, *53*, *56* and *58* (accounting for 17.2 % of *ZmCCT*s in maize inbred line B73) increased, involving more than 25 (60.9 %) maize lines. The copy number of *ZmCCT*s *10*, *20* and *44* (accounting for 5.2 % of *ZmCCT*s) decreased, involving more than 10 (24.4 %) maize lines. The copy number of *ZmCCT36* increased in 7 (41.2 %) of 17 tropical/subtropical maize lines and in 15 (62.5 %) of 24 temperate maize lines, respectively. The copy number of *ZmCCT51* increased in 3 (17.7 %) of 17 tropical/subtropical maize lines and in 10 (41.7 %) of 24 temperate maize lines, respectively. The copy number of both *ZmCCT42* and *ZmCCT55* had no changes in tropical/subtropical maize lines but increased in 11 (45.8 %) and 7 (29.2 %) of the 24 temperate maize lines, respectively (Fig. 3).

### SVs of *ZmCCT*s in different maize inbred lines

With the SK genome as reference, *ZmCCT*s *8*, *29*, *30*, *41* and *43* were found to have SVs in the v4 B73 genome, showing deletions and insertions of DNA segments (Table 2). These SVs were divided into three types: AA, for SVs of the same structure as B73 but different structure from SK; TT, for SVs of the same structure as SK but different structure from B73; and NN, for SVs unable to evaluate **[see****Supporting Information—Table S6****]**.

**Table 2.** Structural variations (SVs) of *ZmCCT*s in the v4 B73 genome against the SK genome. The analyses were based on the previous open genome sequence data set in the literature (Yang *et al.* 2019; The data sets were from Yan’s lab of the National Key Laboratory of Crop Genetic Improvement, Huazhong Agricultural University, China. http://maizego.org/Resources.html); SV, structural variation; UTR, untranslated region; v4, version 4; *ZmCCT*, ZmCCT gene.

ZmCCT				SV						
Name	Chromosome number	Gene body range on chromosome		Number	Type	Start	Stop	Length of insertion/deletion (bp)	Region	Distance away from start codon ATG (bp)
		Start	Stop							
*ZmCCT8*	1	272190270	272192279	SV1	Insertion	272190299	272190300	15	Upstream	25 upstream
*ZmCCT29*	5	70352028	70356300	SV2	Insertion	70352067	70352068	13	5′ UTR	4141 upstream
				SV3		70353576	70353577	2302	5′ UTR	2632 upstream
*ZmCCT30*	5	82139629	82142593	SV4	Insertion	82140693	82140694	21	Exon	1064 downstream
				SV5	Deletion	82141884	82141898	14	Intron	1043–2255 downstream
*ZmCCT41*	7	148065548	148071232	SV6	Deletion	148067829	148068185	356	Intron	2970–3326 upstream
				SV7	Deletion	148070516	148070532	16	Intron	623–629 upstream
*ZmCCT43*	7	180509587	180543685	SV8	Insertion	180520885	180520886	23	Intron	4771 upstream
				SV9	Deletion	180520895	180520909	14	Intron	4747 upstream
				SV10	Deletion	180520929	180520950	21	Intron	4706 upstream
				SV11	Deletion	180520961	180521014	53	Intron	4642 upstream
				SV12	Deletion	180521021	180521037	16	Intron	4619 upstream
				SV13	Deletion	180521056	180521069	13	Intron	4587 upstream
				SV14	Deletion	180521075	180521121	46	Intron	4535 upstream
				SV15	Deletion	180521125	180521175	50	Intron	4481 upstream

The genomes of 521 maize inbred lines (http://maizego.org/Resources.html) were scanned by reference to the SVs of *ZmCCT*s in the B73 genome according to the methods described by Yang *et al.* (2019). Consequently, one TT-type SV, SV7, of *ZmCCT41* was found in 287 maize lines. Five TT-type SVs, SVs 9–13, of *ZmCCT43* were found in 375 to 472 maize lines depending on the SV. One AA-type SV, SV2 of *ZmCCT29* was found in 85 maize lines. One AA-type SV, SV4, of *ZmCCT30* was found in 152 lines. One AA-type SV, SV8, of *ZmCCT43* was found in 46 maize lines **[see****Supporting Information—Table S6****]**.

### Expression of *ZmCCT*s under biotic and abiotic stresses

Expression levels of 58 *ZmCCT*s from B73 were analysed in maize lines under abiotic and biotic stresses (Table 3). Forty-two (72.4 %) of 58 *ZmCCT*s responded to the stresses, and they together made a total of 84 differential expression events when compared to those in corresponding unstressed tissues of the same maize lines. The responses to drought, cold and heat accounted for 73 (86.9 %) of 84 differential expression events (Table 3), of which 53 (63.1 %) resulted from *ZmCCT*s in the COL clade, 17 (20.2 %) from *ZmCCT*s in the PRR clade, 12 (14.3 %) from *ZmCCT*s in the CMF clade and 2 (2.4 %) from a TIFY clade (Table 3).

**Table 3.** Expression of 58 *ZmCCT*s from B73 line in leaves of maize inbred lines under abiotic and biotic stresses. The raw open data sets under drought, cold, heat and salt were from Makarevitch *et al.* (2015); Zenda *et al.* (2019), and the raw data set on infection with *C*. *zeina* was from Swart *et al.* (2017), which had three biological repeats. Black bold and negative black bold values indicate up-regulation and down-regulation of gene expression at *p* < 0.05, respectively. *The EF was calculated based on the sequence read counts of the target genes. *C*., *Cercospora*; EF, expression fold; *ZmCCT*, ZmCCT gene.

ZmCCT	Drought-stressed YE8112 leaf		Drought-stressed Mo17 leaf		Cold-stressed B73 leaf		Heat-stressed B73 leaf		Salt-stressed B73 leaf		B73 leaf infected by *C*. *zeina*	
	Log2 EF*	P-value	Log2 EF*	P-value	Log2 EF*	P-value	Log2 EF*	P-value	Log2 EF*	P-value	Log2 EF*	P-value
*ZmCCT1*	−0.08	0.95	−0.67	0.43	**−1.02**	0.00	**1.48**	0.00	−1.30	0.05	**−1.82**	0.00
*ZmCCT2*	0.28	0.23	0.19	0.55	**2.76**	0.00	**−1.10**	0.00	−0.48	0.09	−0.37	0.04
*ZmCCT3*	0.45	0.09	−0.89	0.06	−0.45	0.00	−0.52	0.00	−0.89	0.00	−0.41	0.05
*ZmCCT4*	0.00	1.00	0.00	1.00	3.91	1.00	3.32	1.00	0.00	1.00	0.00	1.00
*ZmCCT5*	−0.26	0.51	−1.25	0.05	**1.86**	0.00	**−1.17**	0.00	−0.20	0.63	−0.38	0.21
*ZmCCT6*	4.91	0.60	0.26	0.92	−0.06	1.00	**1.97**	0.00	0.00	1.00	0.00	1.00
*ZmCCT7*	0.26	0.17	−0.24	0.48	−0.69	0.00	−0.99	0.00	**−1.30**	0.00	0.16	0.39
*ZmCCT8*	0.10	0.75	**−3.65**	0.00	**−3.70**	0.00	**−1.66**	0.00	0.39	0.47	−0.20	0.40
*ZmCCT9*	**1.01**	0.00	−0.86	0.01	**−1.16**	0.00	−0.77	0.00	−0.86	0.00	0.14	0.46
*ZmCCT10*	−4.74	0.57	4.32	1.00	−1.00	0.09	−0.44	0.50	0.63	0.61	−5.06	1.00
*ZmCCT11*	−0.04	0.83	−0.09	0.79	**3.33**	0.00	**−1.71**	0.00	−0.53	0.04	0.03	0.90
*ZmCCT12*	−0.12	0.53	0.41	0.26	**5.30**	0.00	**−1.19**	0.00	0.67	0.01	−0.36	0.05
*ZmCCT13*	0.13	0.93	0.27	0.78	−0.38	0.04	−0.46	0.01	−0.99	0.42	−0.30	0.59
*ZmCCT14*	0.21	0.40	**−1.02**	0.01	**−1.15**	0.00	**−2.58**	0.00	−0.15	0.59	−0.38	0.12
*ZmCCT15*	0.38	0.36	−0.34	0.91	0.33	0.35	**−1.52**	0.00	**−3.73**	0.00	0.43	0.52
*ZmCCT16*	0.00	1.00	6.91	0.14	−0.89	0.64	**3.64**	0.00	0.00	1.00	0.00	1.00
*ZmCCT17*	0.00	1.00	4.91	1.00	0.00	1.00	1.00	1.00	0.00	1.00	0.00	1.00
*ZmCCT18*	0.24	0.61	−0.93	0.13	−1.95	0.10	0.10	1.00	−0.93	0.12	−0.57	0.16
*ZmCCT19*	0.56	0.17	**−2.01**	0.00	**−1.01**	0.00	0.96	0.00	−0.67	0.32	0.27	0.68
*ZmCCT20*	0.40	0.27	−0.26	0.53	−**1.36**	0.00	−0.73	0.00	0.79	0.22	−0.04	1.00
*ZmCCT21*	−0.97	0.00	0.42	0.45	**3.51**	0.00	−0.14	0.59	0.47	0.17	0.17	0.32
*ZmCCT22*	−0.60	0.01	−0.27	0.66	**2.55**	0.00	−0.83	0.00	−0.26	0.49	−0.37	0.12
*ZmCCT23*	−0.30	0.14	−0.01	0.98	**2.90**	0.00	**−1.04**	0.00	0.12	0.61	−0.06	0.75
*ZmCCT24*	−0.61	0.03	**−3.86**	0.00	0.54	0.00	**−3.02**	0.00	**1.98**	0.00	−0.52	0.02
*ZmCCT25*	0.42	0.09	−0.22	0.59	**−1.28**	0.00	−0.35	0.00	−0.65	0.04	−0.20	0.38
*ZmCCT26*	0.61	0.01	**−1.62**	0.00	**−2.45**	0.00	**−1.12**	0.00	−0.08	0.79	0.22	0.29
*ZmCCT27*	−0.30	0.71	0.09	0.85	−0.14	0.82	−0.48	0.35	−1.02	0.20	1.89	0.05
ZmCCT	Drought-stressed YE8112 leaf		Drought-stressed Mo17 leaf		Cold-stressed B73 leaf		Heat-stressed B73 leaf		Salt-stressed B73 leaf		B73 leaf infected by C. zeina	
	Log2 EF*	P-value	Log2 EF*	P-value	Log2 EF*	P-value	Log2 EF*	P-value	Log2 EF*	P-value	Log2 EF*	P-value
*ZmCCT28*	−0.15	0.45	−0.34	0.28	−0.05	0.51	−0.31	0.00	0.28	0.24	−0.21	0.22
*ZmCCT29*	0.00	1.00	0.00	1.00	1.00	1.00	1.00	1.00	0.00	1.00	5.20	1.00
*ZmCCT30*	−0.80	0.00	**−4.34**	0.00	0.88	0.00	**−1.74**	0.00	−0.90	0.10	−0.65	0.00
*ZmCCT31*	0.12	0.52	−0.88	0.01	−0.66	0.00	**1.55**	0.00	−0.72	0.00	−0.57	0.00
*ZmCCT32*	**1.36**	0.00	**−1.07**	0.00	**−1.61**	0.00	−1.20	0.00	**1.50**	0.00	**1.48**	0.00
*ZmCCT33*	−0.93	0.00	0.62	0.07	**6.89**	0.00	**1.46**	0.00	−0.83	0.03	0.07	0.71
*ZmCCT34*	0.10	0.64	−0.72	0.04	**3.21**	0.00	**−1.11**	0.00	0.29	0.33	−0.02	0.94
*ZmCCT35*	0.14	0.49	−0.31	0.36	0.96	0.00	**−1.09**	0.00	0.62	0.07	−0.16	0.46
*ZmCCT36*	1.39	0.56	−1.44	0.43	**−1.13**	0.00	−0.15	0.37	**9.74**	0.00	−6.64	0.28
*ZmCCT37*	0.04	0.85	−0.04	0.89	−0.30	0.00	−0.43	0.00	0.38	0.14	−0.05	0.78
*ZmCCT38*	−0.26	0.24	−**2.13**	0.00	1.07	0.00	**−2.94**	0.00	0.61	0.82	0.27	0.29
*ZmCCT39*	0.10	0.84	−2.70	0.14	−0.53	0.00	**−1.46**	0.00	0.51	0.38	−0.17	1.00
*ZmCCT40*	0.30	0.50	0.03	0.97	−0.88	0.00	−0.19	0.18	−0.66	0.05	−0.30	0.31
*ZmCCT41*	−0.36	0.07	−0.49	0.16	**4.42**	0.00	**−1.25**	0.00	−0.67	0.01	−0.73	0.00
*ZmCCT42*	**3.01**	0.00	5.91	0.60	**−1.26**	0.00	−0.20	0.45	−0.71	0.19	0.35	0.61
*ZmCCT43*	0.10	0.67	**−1.44**	0.00	−0.33	0.00	**−1.15**	0.00	−0.21	0.48	−0.47	0.01
*ZmCCT44*	0.00	1.00	−0.31	0.72	0.00	1.00	0.00	1.00	0.00	1.00	0.00	1.00
*ZmCCT45*	0.17	0.43	−0.11	0.81	−0.38	0.00	−0.92	0.00	−0.19	0.49	−0.16	0.40
*ZmCCT46*	0.04	0.94	−1.28	0.54	0.51	0.14	**−1.47**	0.00	**−4.76**	0.00	**1.38**	0.03
*ZmCCT47*	−0.66	0.52	−0.64	0.81	0.64	0.30	−1.18	0.08	−6.64	0.15	−5.06	1.00
*ZmCCT48*	0.00	1.00	0.00	1.00	0.00	1.00	0.00	1.00	0.00	1.00	0.00	1.00
*ZmCCT49*	**−1.73**	0.00	**−5.05**	0.00	**1.06**	0.00	**−2.36**	0.00	−1.33	0.08	**−1.31**	0.00
*ZmCCT50*	0.11	0.70	0.94	0.02	0.38	0.00	0.47	0.00	−0.95	0.00	0.13	0.52
*ZmCCT51*	−0.27	0.20	**−1.11**	0.03	**2.18**	0.00	**1.77**	0.00	0.22	0.75	0.73	0.36
*ZmCCT52*	0.25	0.21	**−1.47**	0.00	−0.93	0.00	−0.53	0.00	−0.68	0.00	−0.28	0.12
*ZmCCT53*	−0.10	0.63	−0.92	0.01	0.48	0.00	**−1.80**	0.00	0.11	0.66	−0.36	0.05
*ZmCCT54*	−0.05	0.90	−1.37	0.32	−0.79	0.00	**−**1**.08**	0.00	−0.58	0.18	−0.35	0.32
*ZmCCT55*	0.08	0.71	**1.43**	0.01	**−1.64**	0.00	0.23	0.11	−0.44	0.35	0.06	0.80
*ZmCCT56*	1.00	0.61	−4.91	1.00	**−1.98**	0.01	**−1.45**	0.03	−1.43	0.53	0.00	1.00
*ZmCCT57*	0.55	0.02	−0.97	0.00	**−1.85**	0.00	−0.52	0.00	**1.65**	0.00	0.15	0.51
*ZmCCT58*	0.91	0.00	**−1.21**	0.00	**−2.16**	0.00	−0.17	0.12	−0.84	0.11	−0.98	0.01

Of the 42 stress-induced *ZmCCT*s, 28 (66.7 %) responded to heat in B73 leaves, of which 22 (78.6 %) were downregulated and 6 (21.4 %) were upregulated. Twenty-seven (64.3 %) responded to cold in B73 leaves, of which 15 (55.6 %) were downregulated and 12 (44.4 %) were upregulated. Fourteen (33.3 %) responded to drought in leaves of the drought-sensitive line Mo17 (Zenda *et al.* 2019), of which one (7.4 %) was downregulated and three (92.6 %) were upregulated. Only four *ZmCCT*s responded to drought in leaves of drought-tolerant YE8112 (Zenda *et al.* 2019), of which one (25 %) was downregulated and three (75 %) were upregulated. Very few *ZmCCT*s responded to salt and infection with *Cercospora zeina* in B73 leaves (Table 3).

### Expression rhythm of *ZmCCT*s

**Figure 4.**
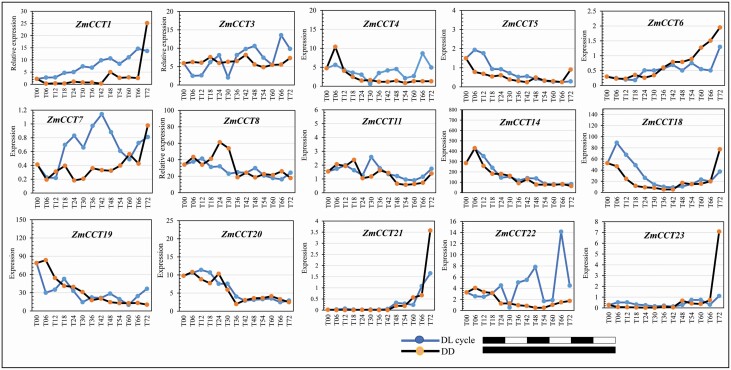

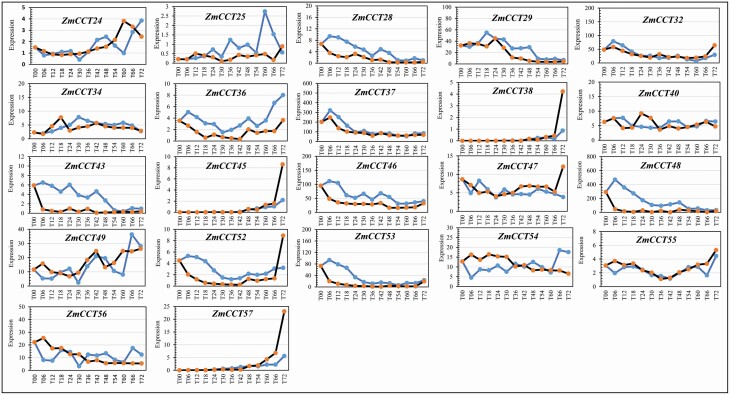
Expression of the *ZmCCT*s at 13 time points in embryonic leaves during seed germination of *Z*. *mays* cv. White Crystal under DL cycles and DD. The results were based on the transcriptome data sets of seeds of *Z*. *mays* cv. White Crystal under the two conditions of DL cycles (Liu *et al.* 2013) and DD (Chang *et al.* 2019). T00 represents dry seed; T06, 12, … represent time points post imbibition of seeds under DL cycles and DD. DD, dark-dark; DL, dark (11 h)/light (13 h); *ZmCCT*, ZmCCT gene.

Based on the transcriptomes of embryonic leaves during seed germination under DL cycles and DD, there were 37 (63.8 %) of 58 *ZmCCT*s in inbred line B73 that showed differential expression (Fig. 4). From the beginning to the end of the treatments, *ZmCCT* expression could be roughly divided into five patterns: uphill pattern (i.e. the expression level tended to increase gradually); downhill-pattern (i.e. the expression level tended to decrease gradually); zigzag-pattern (i.e. the expression level had single or multiple obvious peaks and valleys); └-pattern (i.e. the expression level was high initially, and then it decreased suddenly and remained flat till the end); and ⅃-pattern (the expression level remained low until suddenly increased sharply at the last time point) (Fig. 4).

There were four *ZmCCT*s (i.e. *43*, *46*, *48* and *48*) under DD, and eight *ZmCCT*s (i.e. *8*, *14*, *18*–*20*, *34*, *37* and *53*) under DL that presented the └-pattern. Meanwhile, there were seven *ZmCCT*s (i.e. *1*, *21*, *23*, *38*, *45*, *52* and *57*) under DD, and one *ZmCCT* (i.e. *21*) under DL that presented the ⅃-pattern expression (Fig. 4).

Under the two treatments, in terms of overall expression, the genes with similar expression patterns included *ZmCCT*s *6*, *11*, *14*, *18*, *20*, *21*, *23*, *32*, *34*, *36–38*, *45* and *47*. At the initial time point of the two treatments, the genes with a completely opposite expression pattern included *ZmCCT*s *1*, *3*, *5*, *18*, *19*, *22*, *23*, *28*, *36*, *43*, *46*, *48*, *49* and *52–56*. At the last time point of the two treatments, the genes with a completely opposite expression pattern involved *ZmCCT*s *1*, *3*, *8*, *22*, *24*, *25*, *47*, *49* and *56* (Fig. 4).

### Association of *ZmCCT*s with QTLs

Analysis of the relevant data sets in the GWAS Atlas database revealed that 24 (41.4 %) of 58 *ZmCCT*s were associated with QTLs in the v4 B73 genome (Table 4) in inbred line B73. *ZmCCT*s *11*, *34*, *43*, *57* and *58* were linked to four QTLs. *ZmCCT23* was associated with three QTLs. *ZmCCT*s *1*, *2* and *31* were related to two QTLs. Other 17 *ZmCCT*s were associated with one QTL (Table 4), respectively.

**Table 4.** Association between *ZmCCT*s and QTLs in the v4 B73 genome. The information was extracted from the public data set in the GWAS Atlas (Tian *et al.* 2020). GWAS, genome-wide association study; ID, identification; QTL, quantitative trait locus; SNP, single nucleotide polymorphism.

ZmCCT	SNP ID number	SNP position	QTL/trait	P-values	Other gene(s) covered within the QTL	PubMed ID in NCBI database
*ZmCCT1*	zma191016	chr1:8675749	ear infructescence position	3.80E-04	Zm00001d027598 Zm00001d027596 Zm00001d027597	24514905
	zma43162386	chr1:8674425	leaf length	6.20E-07	Zm00001d027598 Zm00001d027596 Zm00001d027597	21217756
*ZmCCT2*	zma43149907	chr1:60530979	southern leaf blight resistance	1.34E-09	Zm00001d029149	25475173
	zma43156454	chr1:60537720	southern leaf blight resistance	9.37E-10	Zm00001d029149	25475173
*ZmCCT3*	zma43161909	chr1:92222374	ear infructescence position	1.85E-04	Zm00001d029886 Zm00001d029885	24514905
*ZmCCT11*	zma43153974	chr2:145691242	shoot biomass	1.50E-05	Zm00001d004875	27768702
	zma10098914	chr2:145691595	southern leaf blight resistance	8.35E-09	Zm00001d004875	25475173
	zma43157232	chr2:145684456	tassel length	8.47E-11	Zm00001d004875 Zm00001d004874 Zm00001d004875	26801971
*ZmCCT11*	zma43153974	chr2:145691242	transpiration efficiency	1.50E-05	Zm00001d004875	27768702
*ZmCCT12*	zma43145709	chr2:201957407	above ear plant height	2.07E-08	Zm00001d006212	29150689
*ZmCCT14*	zma11374170	chr2:225517150	days to flowering trait	7.58E-09	Zm00001d007240	23840585
*ZmCCT15*	zma15275529	chr3:184827163	plant height	3.46E-07	Zm00001d042958	24514905
*ZmCCT18*	zma43154150	chr4:27316553	ear infructescence position	2.90E-04	Zm00001d049347	24514905
*ZmCCT23*	zma19806124	chr4:200744498	days to silk	2.92E-08	Zm00001d052781 Zm00001d052784 Zm00001d052783	23144785
	zma43155891	chr4:200740173	ear infructescence position	8.34E-05	Zm00001d052782 Zm00001d052781 Zm00001d052781 Zm00001d052783	24514905
	zma43145909	chr4:200738304	tassel branch zone length	1.17E-15	Zm00001d052782 Zm00001d052781 Zm00001d052781	22125498
*ZmCCT27*	zma21185023	chr5:31566925	transpiration rate	4.17E-06	Zm00001d014073 Zm00001d014074	29044609
*ZmCCT29*	zma21907252	chr5:70357243	plant height	1.37E-07	Zm00001d014963	24514905
*ZmCCT30*	zma43149250	chr5:82139979	water use efficiency	6.92E-06	Zm00001d015268 Zm00001d015269	29044609
*ZmCCT31*	zma43153506	chr5:91969815	plant biomass	2.19E-06	Zm00001d015469 Zm00001d015468	29044609
	zma43153506	chr5:91969815	water use efficiency	1.10E-06	Zm00001d015469 Zm00001d015468	29044609
*ZmCCT34*	zma43153430	chr5:209622204	ear infructescence position	3.42E-13	Zm00001d017885 Zm00001d017886	24514905
*ZmCCT34*	zma24511443	chr5:209617684	leaf area trait	5.62E-09	Zm00001d017885 Zm00001d017886	29044609
	zma43153430	chr5:209622204	plant height	5.46E-11	Zm00001d017885 Zm00001d017886	24514905
	zma43157920	chr5:209614341	southern leaf blight resistance	1.47E-06	Zm00001d017885 Zm00001d017885 Zm00001d001732	21217757
*ZmCCT35*	zma43154216	chr5:210408594	leaf area trait	2.40E-07	Zm00001d017939	29044609
*ZmCCT38*	zma43154936	chr6:121365959	days to flowering trait	4.92E-07	Zm00001d037327	24514905
*ZmCCT41*	zma43157943	chr7:148063144	leaf length	2.30E-07	Zm00001d021291 Zm00001d021290	21217756
*ZmCCT43*	zma43155834	chr7:180510629	days to flowering trait	9.63E-12	Zm00001d022590 Zm00001d022590	23840585
	zma43155834	chr7:180510629	days to silk	6.73E-09	Zm00001d022590 Zm00001d022590	23144785
	zma43161601	chr7:180510559	kernel number per row	2.11E-06	Zm00001d022590 Zm00001d022590	29754325
	zma43151879	chr7:180542812	plant height	8.08E-05	Zm00001d022590 Zm00001d022590	24514905
*ZmCCT47*	ma31615680	chr8:174635607	plant height	3.51E-10	Zm00001d012445	24514905
*ZmCCT50*	zma43143021	chr9:36013691	shoot apical meristem volume	4.37E-13	Zm00001d045735	26584889
*ZmCCT52*	zma43151844	chr9:111028354	ear infructescence position	1.85E-12	Zm00001d046925	24514905
*ZmCCT54*	zma43154034	chr9:155206589	number of seminal root	1.16E-05	Zm00001d048369	30472798
*ZmCCT56*	zma43150378	chr10:94434907	photoperiod-sensitive flowering-time trait	8.10E-09	Zm00001d024910 Zm00001d024909	24089449
*ZmCCT56*	zma43147102	chr10:94435667	plant height	8.17E-05	Zm00001d024910 Zm00001d024909	24514905
*ZmCCT58*	zma43150306	B73V4_ctg181:43378	ear infructescence position	2.84E-08	Zm00001d000175 Zm00001d000176 Zm00001d000175 Zm00001d000174	24514905
	zma43150351	B73V4_ctg181:43478	node count	3.60E-09	Zm00001d000175 Zm00001d000176 Zm00001d000174	24514905
	zma43158611	B73V4_ctg181:50751	plant height	7.29E-05	Zm00001d000176	24514905
	zma43158330	B73V4_ctg181:50347	tassel length	1.64E-06	Zm00001d000176	26801971

### Co-expression of *ZmCCT*s with other maize genes

**Figure 5.**
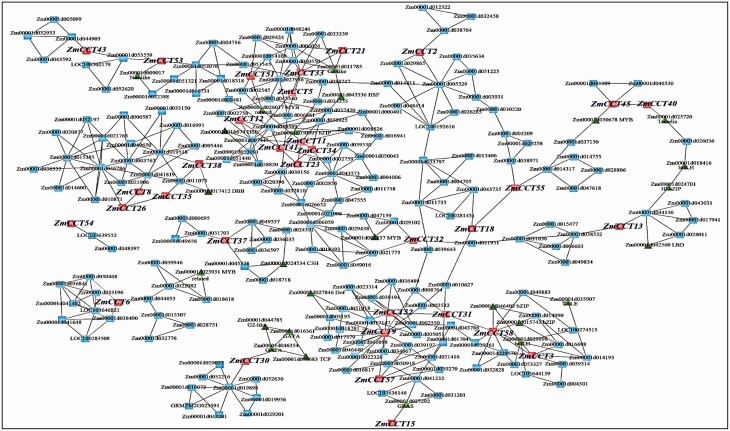
Co-expression of the *ZmCCT*s with other genes in B73. The analysis was based on a data set in the literature (Obayashi *et al.* 2018). *ZmCCT*, ZmCCT gene.

Analysis of the transcriptome data sets of tissue samples of B73 under both control and stresses in the ATTED-II version 9.2 database showed that 33 (56.9 %) of 58 *ZmCCT*s in inbred line B73 were co-expressed with 218 other maize genes, including 16 transcription factors **[see****Supporting Information—Table S7****]**. *ZmCCT*s *6*, *30*, *37* and *54* were in isolated co-expression networks containing a few genes each (Fig. 5). In order to visualize the co-expression networks, the jre_8u_windows_64.exe or the jre_8u_windows_32.exe file was first downloaded and installed followed by installing the Cytoscape_3_3_0_windows_32bit or Cytoscape_3_3_0_windows_32bit file depending on the local Windows system. After installing this software, the reader can double-click and then watch the file ‘Genes co-expressed with *ZmCCT*s in maize inbred line B73’ through the button ‘Zoom In’ on the screen. These files are provided for readers as a compressed supplemental file package in the **Supporting Information**.

**Figure 6.**
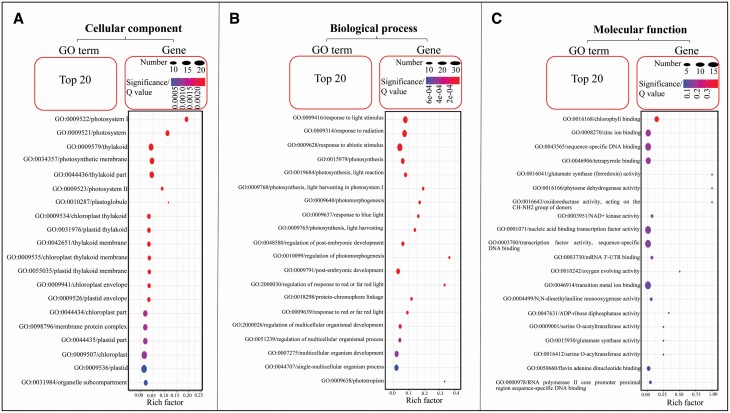
GO enrichment, cellular component (A), biological process (B) and molecular function (C), of the genes co-expressed with *ZmCCT*s in B73. GO, gene ontology; *ZmCCT*, ZmCCT gene. GO, gene ontology; Q, false discovery rate.

The functional categorization by GO analysis indicated that the co-expressed genes were significantly associated with cellular component (Fig. 6A) and biological process (Fig. 6B) rather than molecular function (Fig. 6C).

## Discussion

In this study, it was found that the number of CCT domain-containing genes varies across plant species (Fig. 1A) and among maize inbred lines **[see****Supporting Information—Table S3****]**. The number of *ZmCCT*s identified in maize inbred line B73 based on the v4 B73 genome **[see****Supporting Information—Table S3****]** differed from the 53 *ZmCCT*s that were reported in B73 based on the v2 B73 genome (Jin *et al.* 2018) likely because of the upgrade of gene annotation.

The CNV is one of the most common and most studied forms of SVs (Swanson-Wagner *et al.* 2010). They are most likely caused by non-allelic homologous recombination in plants (Gabur *et al.* 2019). CNVs are closely associated with the chromosome ploidy (Tang and Amon 2013). In this study, plants with higher ploidy had more CCT domain-containing genes compared to diploid species, such as allotetraploid *G*. *hirsutum* (Hu *et al.* 2019), allotetraploid *Glycine max* (Kyriakidou *et al.* 2018), allotetraploid *Triticum aestivum* (Kyriakidou *et al.* 2018), autotetraploid *Solanum tuberosum* (Kyriakidou *et al.* 2018) and tetraploid to octoploid *Panicum virgatum* (Grant 2017) (Fig. 1A). This phenomenon also occurred in different species of the same genus, for example, *G*. *hirsutum* had 1.9 times as many genes as diploid *G*. *raimondii* (Hu *et al.* 2019), *P. virgatum* had 1.86 times as many genes as diploid *P. hallii* (Grant 2017) and *S*. *tuberosum* had 1.96 times as many genes as diploid *S*. *lycopersicum* (Al Shaye *et al.* 2018) (Fig. 1B).

The ancestor of maize was an ancient tetraploid (White and Doebley 1998). However, over time, its genome has reverted to functional diploid (White and Doebley 1998). With diploidization, separation of chromosome segments will lead to the change of maize inbred line-specific CNVs (Eichten *et al.* 2011) which have been found in maize populations (Swanson-Wagner *et al.* 2010). This may partly explain why the genome sizes of B73, Mo17, W22 and SK are marginally different (Springer *et al.* 2018; Yang *et al.* 2019), but the *ZmCCT* number differed among maize inbred lines **[see****Supporting Information—Table S3****]**.

The gene SVs are important clues to domestication and/or breeding (Swanson-Wagner *et al.* 2010) and directly affect trait variations in maize (Gabur *et al.* 2019). More than 3000 SVs have been found in maize. The average length of an individual SV event is about 20 kb but ranges from 1 kb to over 1 Mb in length (Gabur *et al.* 2019). Expression of *ZmCCT*s delays flowering of maize under long day (Hung *et al.* 2012). Therefore, it is reasonably to believe that SVs of 5 *ZmCCT*s *8*, *29*, *30, 41* and *43* in populations of tropical/subtropical and temperate maize lines (Table 2; **see****Supporting Information—Table S6**) were likely specific changes exerted by domestication and/or artificial selection objectives.

Several *ZmCCT*s are associated with flowering-time QTLs (Hung *et al.* 2012), drought and heat tolerance (Ku *et al.* 2016) and stalk rot resistance (Li *et al.* 2017; Wang *et al.* 2017). Quantitative trait loci associated with *ZmCCT*s are important nodes linking the photoperiod to stress tolerance responses under long day, and to how photoperiod affects the adaptability of plants to stresses (Ku *et al.* 2016) and vice versa (Hill and Li 2016). Association of 24 *ZmCCT*s with QTLs suggests that they can be considered important candidate genes of the QTLs, particularly those associated with flowering-related QTLs (Table 4). Responses to abiotic stresses (Table 3) suggest that downregulated expression of *ZmCCT*s would weaken the tolerance of maize to abiotic stress. The co-expression (Fig. 5; **see****Supporting Information—Table S7**) indicated that some *ZmCCT*s were involved with abiotic stimulus. Again, these results together indicate that *ZmCCT*s may play important roles in photoperiod sensitivity and stress adaptability in maize.

During the evenings of long days, CO proteins activate FLOWERING LOCUS T and remain stable through interactions of the LOV domain with FKF1. Meanwhile, FKF1 simultaneously removes CYCLING DOF FACTOR, which is a repressor of CO and *FLOWERING LOCUS T* (Song *et al.* 2012). The COL proteins show a range of sequence identity with CO proteins (Valverde 2011). The genes with COL, CMF and PRR domains promote flowering under short day or delay flowering under long day in cereal crops (Liu *et al.* 2020). In maize, *ZmCOL3* represses circadian clock but enhances *ZmCCT* expression and therefore delays flowering (Jin *et al.* 2018).

The TIFY family, previously known as ZIM, is characterized by a TIF[F/Y]XG sequence motif (Vanholme *et al.* 2007), which has been associated with abiotic and biotic stresses in plants such as maize (Zhang *et al.* 2015). Zf-B/B-box domain-containing proteins from the COL clade are a class of zinc-finger transcription factors with multiple functions (Gangappa and Botto 2014) that act as bridges between light and hormones in plants (Vaishak *et al.* 2019). MBD domain-containing proteins from the TIFY-like clade can enhance transcriptional repression of CCT domain-containing genes and are involved in DNA demethylation and abiotic stress responses. Also, mutation in *AtMBD8* results in a late-flowering phenotype in the C24 ecotype of *Arabidopsis* (Parida *et al.* 2018). GATA domain-containing proteins from the TIFY-like clade have been implicated in light-dependent and nitrate-dependent control of transcription (Reyes *et al.* 2004). The *ZmCCT*s were divided into four distinct clades (Fig. 2A) but the degrees of responses of the clades to the stresses differed in terms of the number of stress-responsive genes (Table 3). Taken all together, in addition to the response to photoperiod, the functions of four clades in *ZmCCT* family are diversified, and even *ZmCCT*s in the same clade appear to have also different functions. Motif 1, identified as a CCT domain motif of ZmCCTs, was only 29-amino acid residue long **[see****Supporting Information—Table S5****]**, shorter than the CCT domain PF06203 which is defined as a 43- to 45-amino acid residue domain in the database (http://pfam.xfam.org/). This is because these amino acid residues identified were only the most conserved core motifs in the CCT domain. Unknown motif 3 was 21-amino acid residue long **[see****Supporting Information—Table S5****]**, existed in 57 ZmCCTs not in ZmCCT, and was closely adjacent to CCT domain (Fig. 2B), suggesting that motif 3 is likely a motif that loses region of the CCT domain.

Photoperiod refers to light time in a rhythmic day-night cycle of 24 h (Velez-Ramirez *et al.* 2011). The critical ‘windows’ of gene expression in the perception of photoperiod are at the transition periods of both dark-light and light-dark (Imaizumi and Kay 2006; Song *et al.* 2015; Shim *et al.* 2017). Therefore, it could be inferred that the *ZmCCT*s └- or ⅃-pattern expression under DD (i.e. *ZmCCT*s *43*, *46*, *48* and *48* for └-pattern and *ZmCCT*s *1*, *21*, *23*, *38*, *45*, *52* and *57* for ⅃-pattern) and under DL cycle (i.e. *ZmCCT*s *8*, *14*, *18*–*20*, *34*, *37* and *53* for └-pattern and *ZmCCT 21* for ⅃-pattern) would be involved in perception of light-dark and/or dark-light transitions (Fig. 4).

## Conclusions

The CCT protein gene number varies greatly across plant species. *ZmCCT* number also varies with maize inbred lines. The data analysed suggest that *ZmCCT*s are involved in photoperiod response and stress adaptability. Based on their expression, it appears that some *ZmCCT*s are induced during dark-light or light-dark transitions. The data presented in this study are informative to further investigating the functions of the *ZmCCT*s.

## Supporting Information

The following additional information is available in the online version of this article—

**Table S1.** Re-sequencing data sets of maize inbred lines in the Sequence Read Archive (SRA) database (https://www.ncbi.nlm.nih.gov/sra/).

**Table S2.** Accession no. of the CCT protein-encoding genes of other plant species other than maize in the database (https://phytozome.jgi.doe.gov/pz/portal.html).

**Table S3.** Accession no. of the *ZmCCT*s in maize inbred lines of B73, Huangzao4, W22, Mo17 and SK.

**Table S4.***ZmCCT*s and ZmCCTs in maize inbred line B73.

**Table S5.** Conserved domains/motifs of maize ZmCCTs.

**Table S6.** Distribution of structural variations (SVs) of *ZmCCT*s in genomes of 521 maize inbred lines.

**Table S7.** Genes co-expressed with *ZmCCT*s in maize inbred line B73.

**Network files.** In order to visualize the co-expression networks, the jre_8u_windows_64.exe or the jre_8u_windows_32.exe file should be downloaded and installed followed by installing the Cytoscape_3_3_0_windows_32bit or Cytoscape_3_3_0_windows_32bit file depending on the local Windows system. After installing this software, the reader can double-click and then watch the file ‘Genes co-expressed with *ZmCCT*s in maize inbred line B73’ through the button ‘Zoom In’ on the screen. These files are provided for readers as a compressed supplemental file package.

plab048_suppl_Supplementary_Table_S1

plab048_suppl_Supplementary_Table_S2

plab048_suppl_Supplementary_Table_S3

plab048_suppl_Supplementary_Table_S4

plab048_suppl_Supplementary_Table_S5

plab048_suppl_Supplementary_Table_S6

plab048_suppl_Supplementary_Table_S7

## Notes

**Phenome, Genome & Environment. Chief Editor:** Colleen Doherty
