# Supplementary material for: Analyses of open-access multi-omics data sets reveal genetic and expression characteristics of maize ZmCCT family genes
Source: AoB Plants. 2021 Aug 16;13(5):plab048. doi: 10.1093/aobpla/plab048 (PMC8459886; doi:10.1093/aobpla/plab048)
Supplement: plab048_suppl_Supplementary_Table_S1 [file plab048_suppl_supplementary_table_s1.docx]

**Table S1** Re-sequencing datasets of maize inbred lines in the SRA database (<https://www.ncbi.nlm.nih.gov/sra/>)

*Note*: ZmCCT, Maize CCT domain-containing protein; *ZmCCT*, ZmCCT gene.

| Inbred line | Germplasm | SRR no. |
| --- | --- | --- |
| TY4 | tropical/subtropical | SRR8906760 |
| TY3 | tropical/subtropical | SRR8907132 |
| CML479 | tropical/subtropical | SRR8907053 |
| CML423 | tropical/subtropical | SRR8906738 |
| CML325 | tropical/subtropical | SRR8907099 |
| CML290 | tropical/subtropical | SRR8906720 |
| CML134 | tropical/subtropical | SRR8906712 |
| CIMBL95 | tropical/subtropical | SRR8906870 |
| CIMBL89 | tropical/subtropical | SRR8906968 |
| CIMBL83 | tropical/subtropical | SRR8906909 |
| CIMBL53 | tropical/subtropical | SRR8906711 |
| CIMBL50 | tropical/subtropical | SRR8906954 |
| CIMBL49 | tropical/subtropical | SRR8906782 |
| CIMBL40 | tropical/subtropical | SRR8906867 |
| CIMBL22 | tropical/subtropical | SRR8907055 |
| CIMBL144 | tropical/subtropical | SRR8906956 |
| CIMBL145 | tropical/subtropical | SRR8906944 |
| CIMBL119 | temperate | SRR8907106 |
| CIMBL116 | temperate | SRR8906964 |
| CIMBL115 | temperate | SRR8906810 |
| CIMBL111 | temperate | SRR8906639 |
| CIMBL11 | temperate | SRR8906719 |
| CIMBL100 | temperate | SRR8906806 |
| CML411 | temperate | SRR8906803 |
| CIMBL113 | temperate | SRR8906913 |
| CML122 | temperate | SRR8907031 |
| Mo17 | temperate | SRR8907080 |
| R15X1141 | temperate | SRR8906848 |
| SI446 | temperate | SRR8906850 |
| B111 | temperate | SRR8906762 |
| RY713 | temperate | SRR8906708 |
| 4F1 | temperate | SRR8906845 |
| LV28 | temperate | SRR8907091 |
| NAN21-3 | temperate | SRR8907100 |
| SY1039 | temperate | SRR8907130 |
| CIMBL1 | temperate | SRR8906869 |
| Q1261 | temperate | SRR8907065 |
| 975-12 | temperate | SRR8906844 |
| GEMS6 | temperate | SRR8907069 |
| DAN340 | temperate | SRR8906840 |
| P178 | temperate | SRR8906912 |
