# Supplementary material for: Analyses of open-access multi-omics data sets reveal genetic and expression characteristics of maize ZmCCT family genes
Source: AoB Plants. 2021 Aug 16;13(5):plab048. doi: 10.1093/aobpla/plab048 (PMC8459886; doi:10.1093/aobpla/plab048)
Supplement: plab048_suppl_Supplementary_Table_S2 [file plab048_suppl_supplementary_table_s2.docx]

| **Table S2** Accession no. of the CCT protein-encoding genes of other plant species other than maize in the database (<https://phytozome.jgi.doe.gov/pz/portal.html>)  *Note*: ZmCCT, Maize CCT domain-containing protein; *ZmCCT*, ZmCCT gene. | | | | |
| --- | --- | --- | --- | --- |
| **Species** | **Accession no.** |  | **Species** | **Accession no.** |
| *Amaranthus hypochondriacus* | AHYPO_000338-RA |  | *Kalanchoe laxiflora* | Kalax.0298s0022 |
| *Amaranthus hypochondriacus* | AHYPO_000463-RA |  | *Kalanchoe laxiflora* | Kalax.0333s0023 |
| *Amaranthus hypochondriacus* | AHYPO_001086-RA |  | *Kalanchoe laxiflora* | Kalax.0333s0057 |
| *Amaranthus hypochondriacus* | AHYPO_001785-RA |  | *Kalanchoe laxiflora* | Kalax.0339s0016 |
| *Amaranthus hypochondriacus* | AHYPO_001937-RA |  | *Kalanchoe laxiflora* | Kalax.0351s0027 |
| *Amaranthus hypochondriacus* | AHYPO_003076-RA |  | *Kalanchoe laxiflora* | Kalax.0359s0024 |
| *Amaranthus hypochondriacus* | AHYPO_004822-RA |  | *Kalanchoe laxiflora* | Kalax.0365s0032 |
| *Amaranthus hypochondriacus* | AHYPO_005135-RA |  | *Kalanchoe laxiflora* | Kalax.0402s0015 |
| *Amaranthus hypochondriacus* | AHYPO_008107-RA |  | *Kalanchoe laxiflora* | Kalax.0408s0042 |
| *Amaranthus hypochondriacus* | AHYPO_009477-RA |  | *Kalanchoe laxiflora* | Kalax.0485s0035 |
| *Amaranthus hypochondriacus* | AHYPO_011127-RA |  | *Kalanchoe laxiflora* | Kalax.0490s0025 |
| *Amaranthus hypochondriacus* | AHYPO_011535-RA |  | *Kalanchoe laxiflora* | Kalax.0500s0001 |
| *Amaranthus hypochondriacus* | AHYPO_011953-RA |  | *Kalanchoe laxiflora* | Kalax.0508s0020 |
| *Amaranthus hypochondriacus* | AHYPO_012236-RA |  | *Kalanchoe laxiflora* | Kalax.0510s0020 |
| *Amaranthus hypochondriacus* | AHYPO_012262-RA |  | *Kalanchoe laxiflora* | Kalax.0530s0001 |
| *Amaranthus hypochondriacus* | AHYPO_012728-RA |  | *Kalanchoe laxiflora* | Kalax.0543s0035 |
| *Amaranthus hypochondriacus* | AHYPO_014205-RA |  | *Kalanchoe laxiflora* | Kalax.0578s0008 |
| *Amaranthus hypochondriacus* | AHYPO_014235-RA |  | *Kalanchoe laxiflora* | Kalax.0598s0005 |
| *Amaranthus hypochondriacus* | AHYPO_015505-RA |  | *Kalanchoe laxiflora* | Kalax.0660s0022 |
| *Amaranthus hypochondriacus* | AHYPO_015509-RA |  | *Kalanchoe laxiflora* | Kalax.0667s0002 |
| *Amaranthus hypochondriacus* | AHYPO_015810-RA |  | *Kalanchoe laxiflora* | Kalax.0741s0011 |
| *Amaranthus hypochondriacus* | AHYPO_016272-RA |  | *Kalanchoe laxiflora* | Kalax.0775s0003 |
| *Amaranthus hypochondriacus* | AHYPO_017676-RA |  | *Kalanchoe laxiflora* | Kalax.0843s0013 |
| *Amaranthus hypochondriacus* | AHYPO_019064-RA |  | *Kalanchoe laxiflora* | Kalax.0872s0013 |
| *Amaranthus hypochondriacus* | AHYPO_019686-RA |  | *Kalanchoe laxiflora* | Kalax.0881s0005 |
| *Amaranthus hypochondriacus* | AHYPO_019822-RA |  | *Kalanchoe laxiflora* | Kalax.0939s0004 |
| *Amaranthus hypochondriacus* | AHYPO_019961-RA |  | *Kalanchoe laxiflora* | Kalax.0942s0006 |
| *Amaranthus hypochondriacus* | AHYPO_020813-RA |  | *Kalanchoe laxiflora* | Kalax.1038s0001 |
| *Amborella trichopoda* | evm_27.model.AmTr_v1.0_scaffold00001.405 | | *Kalanchoe laxiflora* | Kalax.1062s0005 |
| *Amborella trichopoda* | evm_27.model.AmTr_v1.0_scaffold00001.492 | | *Kalanchoe laxiflora* | Kalax.1219s0007 |
| *Amborella trichopoda* | evm_27.model.AmTr_v1.0_scaffold00006.243 | | *Kalanchoe laxiflora* | Kalax.1493s0002 |
| *Amborella trichopoda* | evm_27.model.AmTr_v1.0_scaffold00009.431 | | *Kalanchoe laxiflora* | Kalax.1655s0002 |
| *Amborella trichopoda* | evm_27.model.AmTr_v1.0_scaffold00016.134 | | *Kalanchoe laxiflora* | Kalax.1668s0001 |
| *Amborella trichopoda* | evm_27.model.AmTr_v1.0_scaffold00019.214 | | *Lactuca sativa* | Lsat_1_v5_gn_0_37380.1 |
| *Amborella trichopoda* | evm_27.model.AmTr_v1.0_scaffold00019.427 | | *Lactuca sativa* | Lsat_1_v5_gn_0_5521.1 |
| *Amborella trichopoda* | evm_27.model.AmTr_v1.0_scaffold00021.263 | | *Lactuca sativa* | Lsat_1_v5_gn_0_5541.1 |
| *Amborella trichopoda* | evm_27.model.AmTr_v1.0_scaffold00023.165 | | *Lactuca sativa* | Lsat_1_v5_gn_1_14320.1 |
| *Amborella trichopoda* | evm_27.model.AmTr_v1.0_scaffold00025.176 | | *Lactuca sativa* | Lsat_1_v5_gn_1_75661.1 |
| *Amborella trichopoda* | evm_27.model.AmTr_v1.0_scaffold00032.104 | | *Lactuca sativa* | Lsat_1_v5_gn_2_110060.1 |
| *Amborella trichopoda* | evm_27.model.AmTr_v1.0_scaffold00033.200 | | *Lactuca sativa* | Lsat_1_v5_gn_2_115441.1 |
| *Amborella trichopoda* | evm_27.model.AmTr_v1.0_scaffold00039.227 | | *Lactuca sativa* | Lsat_1_v5_gn_2_135961.1 |
| *Amborella trichopoda* | evm_27.model.AmTr_v1.0_scaffold00061.184 | | *Lactuca sativa* | Lsat_1_v5_gn_2_40740.1 |
| *Amborella trichopoda* | evm_27.model.AmTr_v1.0_scaffold00066.82 | | *Lactuca sativa* | Lsat_1_v5_gn_2_86121.1 |
| *Amborella trichopoda* | evm_27.model.AmTr_v1.0_scaffold00073.16 | | *Lactuca sativa* | Lsat_1_v5_gn_3_59241.1 |
| *Amborella trichopoda* | evm_27.model.AmTr_v1.0_scaffold00111.5 | | *Lactuca sativa* | Lsat_1_v5_gn_4_175001.1 |
| *Ananas comosus* | Aco000055 |  | *Lactuca sativa* | Lsat_1_v5_gn_4_68520.1 |
| *Ananas comosus* | Aco000199 |  | *Lactuca sativa* | Lsat_1_v5_gn_4_88520.1 |
| *Ananas comosus* | Aco001519 |  | *Lactuca sativa* | Lsat_1_v5_gn_5_110200.1 |
| *Ananas comosus* | Aco001932 |  | *Lactuca sativa* | Lsat_1_v5_gn_5_122401.1 |
| *Ananas comosus* | Aco002998 |  | *Lactuca sativa* | Lsat_1_v5_gn_5_21481.1 |
| *Ananas comosus* | Aco003046 |  | *Lactuca sativa* | Lsat_1_v5_gn_5_51381.1 |
| *Ananas comosus* | Aco003091 |  | *Lactuca sativa* | Lsat_1_v5_gn_5_83141.1 |
| *Ananas comosus* | Aco004643 |  | *Lactuca sativa* | Lsat_1_v5_gn_5_86540.1 |
| *Ananas comosus* | Aco005280 |  | *Lactuca sativa* | Lsat_1_v5_gn_6_4580.1 |
| *Ananas comosus* | Aco006545 |  | *Lactuca sativa* | Lsat_1_v5_gn_6_66601.1 |
| *Ananas comosus* | Aco006990 |  | *Lactuca sativa* | Lsat_1_v5_gn_6_85380.1 |
| *Ananas comosus* | Aco007020 |  | *Lactuca sativa* | Lsat_1_v5_gn_7_111641.1 |
| *Ananas comosus* | Aco009467 |  | *Lactuca sativa* | Lsat_1_v5_gn_7_56240.1 |
| *Ananas comosus* | Aco011012 |  | *Lactuca sativa* | Lsat_1_v5_gn_7_6501.1 |
| *Ananas comosus* | Aco011698 |  | *Lactuca sativa* | Lsat_1_v5_gn_7_83621.1 |
| *Ananas comosus* | Aco011701 |  | *Lactuca sativa* | Lsat_1_v5_gn_7_97240.1 |
| *Ananas comosus* | Aco013137 |  | *Lactuca sativa* | Lsat_1_v5_gn_8_146440.1 |
| *Ananas comosus* | Aco014237 |  | *Lactuca sativa* | Lsat_1_v5_gn_8_21260.1 |
| *Ananas comosus* | Aco014592 |  | *Lactuca sativa* | Lsat_1_v5_gn_8_4341.1 |
| *Ananas comosus* | Aco015080 |  | *Lactuca sativa* | Lsat_1_v5_gn_8_5061.1 |
| *Ananas comosus* | Aco016038 |  | *Lactuca sativa* | Lsat_1_v5_gn_8_66520.1 |
| *Ananas comosus* | Aco016766 |  | *Lactuca sativa* | Lsat_1_v5_gn_9_102721.1 |
| *Ananas comosus* | Aco020309 |  | *Lactuca sativa* | Lsat_1_v5_gn_9_18261.1 |
| *Ananas comosus* | Aco021178 |  | *Lactuca sativa* | Lsat_1_v5_gn_9_21300.1 |
| *Ananas comosus* | Aco021314 |  | *Lactuca sativa* | Lsat_1_v5_gn_9_4901.1 |
| *Ananas comosus* | Aco022755 |  | *Lactuca sativa* | Lsat_1_v5_gn_9_58341.1 |
| *Ananas comosus* | Aco023175 |  | *Lactuca sativa* | Lsat_1_v5_gn_9_58381.1 |
| *Ananas comosus* | Aco023820 |  | *Lactuca sativa* | Lsat_1_v5_gn_9_79080.1 |
| *Ananas comosus* | Aco024204 |  | *Linum usitatissimum* | Lus10000566 |
| *Ananas comosus* | Aco025799 |  | *Linum usitatissimum* | Lus10001806 |
| *Ananas comosus* | Aco026137 |  | *Linum usitatissimum* | Lus10002412 |
| *Ananas comosus* | Aco026499 |  | *Linum usitatissimum* | Lus10002578 |
| *Ananas comosus* | Aco028438 |  | *Linum usitatissimum* | Lus10005008 |
| *Aquilegia coerulea* | Anaoc.0001s0477 |  | *Linum usitatissimum* | Lus10005106 |
| *Aquilegia coerulea* | Anaoc.0001s1887 |  | *Linum usitatissimum* | Lus10005936 |
| *Aquilegia coerulea* | Anaoc.0001s1961 |  | *Linum usitatissimum* | Lus10008878 |
| *Aquilegia coerulea* | Anaoc.0002s0841 |  | *Linum usitatissimum* | Lus10008879 |
| *Aquilegia coerulea* | Anaoc.0002s1005 |  | *Linum usitatissimum* | Lus10014018 |
| *Aquilegia coerulea* | Anaoc.0002s1542 |  | *Linum usitatissimum* | Lus10014478 |
| *Aquilegia coerulea* | Anaoc.0002s1815 |  | *Linum usitatissimum* | Lus10014886 |
| *Aquilegia coerulea* | Anaoc.0003s1752 |  | *Linum usitatissimum* | Lus10014919 |
| *Aquilegia coerulea* | Anaoc.0003s2061 |  | *Linum usitatissimum* | Lus10015098 |
| *Aquilegia coerulea* | Anaoc.0003s2134 |  | *Linum usitatissimum* | Lus10015571 |
| *Aquilegia coerulea* | Anaoc.0004s0006 |  | *Linum usitatissimum* | Lus10015619 |
| *Aquilegia coerulea* | Anaoc.0004s0597 |  | *Linum usitatissimum* | Lus10015720 |
| *Aquilegia coerulea* | Anaoc.0004s1417 |  | *Linum usitatissimum* | Lus10019027 |
| *Aquilegia coerulea* | Anaoc.0004s2106 |  | *Linum usitatissimum* | Lus10019077 |
| *Aquilegia coerulea* | Anaoc.0005s1103 |  | *Linum usitatissimum* | Lus10019899 |
| *Aquilegia coerulea* | Anaoc.0005s1230 |  | *Linum usitatissimum* | Lus10020012 |
| *Aquilegia coerulea* | Anaoc.0006s0423 |  | *Linum usitatissimum* | Lus10020105 |
| *Aquilegia coerulea* | Anaoc.0006s0791 |  | *Linum usitatissimum* | Lus10021629 |
| *Aquilegia coerulea* | Anaoc.0006s0943 |  | *Linum usitatissimum* | Lus10022311 |
| *Aquilegia coerulea* | Anaoc.0006s1405 |  | *Linum usitatissimum* | Lus10023229 |
| *Aquilegia coerulea* | Anaoc.0006s1654 |  | *Linum usitatissimum* | Lus10023230 |
| *Aquilegia coerulea* | Anaoc.0007s0986 |  | *Linum usitatissimum* | Lus10023727 |
| *Aquilegia coerulea* | Anaoc.0008s0667 |  | *Linum usitatissimum* | Lus10024655 |
| *Aquilegia coerulea* | Anaoc.0009s0686 |  | *Linum usitatissimum* | Lus10026238 |
| *Aquilegia coerulea* | Anaoc.0010s0896 |  | *Linum usitatissimum* | Lus10026909 |
| *Aquilegia coerulea* | Anaoc.0010s0913 |  | *Linum usitatissimum* | Lus10027344 |
| *Aquilegia coerulea* | Anaoc.0010s0997 |  | *Linum usitatissimum* | Lus10028137 |
| *Aquilegia coerulea* | Anaoc.0010s1013 |  | *Linum usitatissimum* | Lus10028166 |
| *Aquilegia coerulea* | Anaoc.0011s0089 |  | *Linum usitatissimum* | Lus10031192 |
| *Aquilegia coerulea* | Anaoc.0012s0546 |  | *Linum usitatissimum* | Lus10031584 |
| *Aquilegia coerulea* | Anaoc.0012s0547 |  | *Linum usitatissimum* | Lus10031770 |
| *Aquilegia coerulea* | Anaoc.0012s0548 |  | *Linum usitatissimum* | Lus10032288 |
| *Aquilegia coerulea* | Anaoc.0013s0157 |  | *Linum usitatissimum* | Lus10032839 |
| *Aquilegia coerulea* | Anaoc.0013s0162 |  | *Linum usitatissimum* | Lus10032934 |
| *Aquilegia coerulea* | Anaoc.0013s1136 |  | *Linum usitatissimum* | Lus10036419 |
| *Aquilegia coerulea* | Anaoc.0014s0758 |  | *Linum usitatissimum* | Lus10037636 |
| *Aquilegia coerulea* | Anaoc.0014s0881 |  | *Linum usitatissimum* | Lus10039695 |
| *Aquilegia coerulea* | Anaoc.0014s1198 |  | *Linum usitatissimum* | Lus10040900 |
| *Aquilegia coerulea* | Anaoc.0014s1258 |  | *Linum usitatissimum* | Lus10041096 |
| *Aquilegia coerulea* | Anaoc.0015s0081 |  | *Linum usitatissimum* | Lus10041449 |
| *Aquilegia coerulea* | Anaoc.0015s0572 |  | *Linum usitatissimum* | Lus10042431 |
| *Aquilegia coerulea* | Anaoc.0015s0735 |  | *Linum usitatissimum* | Lus10042865 |
| *Aquilegia coerulea* | Anaoc.0016s1205 |  | *Malus domestica* | MDP0000131980 |
| *Aquilegia coerulea* | Anaoc.0018s0111 |  | *Malus domestica* | MDP0000140484 |
| *Aquilegia coerulea* | Anaoc.0018s0827 |  | *Malus domestica* | MDP0000149453 |
| *Aquilegia coerulea* | Anaoc.0019s0094 |  | *Malus domestica* | MDP0000150823 |
| *Aquilegia coerulea* | Anaoc.0019s0830 |  | *Malus domestica* | MDP0000155879 |
| *Aquilegia coerulea* | Anaoc.0020s0239 |  | *Malus domestica* | MDP0000158886 |
| *Aquilegia coerulea* | Anaoc.0020s0389 |  | *Malus domestica* | MDP0000159765 |
| *Aquilegia coerulea* | Anaoc.0042s0003 |  | *Malus domestica* | MDP0000163405 |
| *Aquilegia coerulea* | Anaoc.0582s0026 |  | *Malus domestica* | MDP0000165719 |
| *Aquilegia coerulea* | Anaoc.0587s0014 |  | *Malus domestica* | MDP0000171024 |
| *Aquilegia coerulea* | Anaoc.0599s0018 |  | *Malus domestica* | MDP0000177126 |
| *Aquilegia coerulea* | Anaoc.0619s0005 |  | *Malus domestica* | MDP0000185616 |
| *Aquilegia coerulea* | Anaoc.0698s0002 |  | *Malus domestica* | MDP0000192617 |
| *Aquilegia coerulea* | Anaoc.0934s0007 |  | *Malus domestica* | MDP0000202669 |
| *Aquilegia coerulea* | Anaoc.1025s0010 |  | *Malus domestica* | MDP0000203014 |
| *Aquilegia coerulea* | Anaoc.1075s0006 |  | *Malus domestica* | MDP0000208320 |
| *Aquilegia coerulea* | Anaoc.1088s0006 |  | *Malus domestica* | MDP0000211664 |
| *Aquilegia coerulea* | Anaoc.1542s0004 |  | *Malus domestica* | MDP0000232355 |
| *Aquilegia coerulea* | Anaoc.1573s0001 |  | *Malus domestica* | MDP0000241871 |
| *Arabidopsis halleri* | Araha.0138s0005 |  | *Malus domestica* | MDP0000244436 |
| *Arabidopsis halleri* | Araha.0550s0054 |  | *Malus domestica* | MDP0000271388 |
| *Arabidopsis halleri* | Araha.1101s0004 |  | *Malus domestica* | MDP0000283079 |
| *Arabidopsis halleri* | Araha.11355s0003 |  | *Malus domestica* | MDP0000286458 |
| *Arabidopsis halleri* | Araha.1159s0014 |  | *Malus domestica* | MDP0000287839 |
| *Arabidopsis halleri* | Araha.14182s0001 |  | *Malus domestica* | MDP0000294359 |
| *Arabidopsis halleri* | Araha.1448s0017 |  | *Malus domestica* | MDP0000297093 |
| *Arabidopsis halleri* | Araha.17146s0001 |  | *Malus domestica* | MDP0000298635 |
| *Arabidopsis halleri* | Araha.2210s0007 |  | *Malus domestica* | MDP0000300696 |
| *Arabidopsis halleri* | Araha.22349s0004 |  | *Malus domestica* | MDP0000302297 |
| *Arabidopsis halleri* | Araha.23363s0006 |  | *Malus domestica* | MDP0000303048 |
| *Arabidopsis halleri* | Araha.23435s0010 |  | *Malus domestica* | MDP0000307370 |
| *Arabidopsis halleri* | Araha.23705s0002 |  | *Malus domestica* | MDP0000310268 |
| *Arabidopsis halleri* | Araha.2389s0021 |  | *Malus domestica* | MDP0000314259 |
| *Arabidopsis halleri* | Araha.24414s0002 |  | *Malus domestica* | MDP0000315507 |
| *Arabidopsis halleri* | Araha.24857s0002 |  | *Malus domestica* | MDP0000316985 |
| *Arabidopsis halleri* | Araha.2816s0002 |  | *Malus domestica* | MDP0000318167 |
| *Arabidopsis halleri* | Araha.2816s0003 |  | *Malus domestica* | MDP0000318891 |
| *Arabidopsis halleri* | Araha.33986s0003 |  | *Malus domestica* | MDP0000321380 |
| *Arabidopsis halleri* | Araha.3398s0005 |  | *Malus domestica* | MDP0000428137 |
| *Arabidopsis halleri* | Araha.39199s0004 |  | *Malus domestica* | MDP0000453272 |
| *Arabidopsis halleri* | Araha.4285s0013 |  | *Malus domestica* | MDP0000509877 |
| *Arabidopsis halleri* | Araha.4474s0004 |  | *Malus domestica* | MDP0000512791 |
| *Arabidopsis halleri* | Araha.4706s0012 |  | *Malus domestica* | MDP0000522954 |
| *Arabidopsis halleri* | Araha.4814s0001 |  | *Malus domestica* | MDP0000565292 |
| *Arabidopsis halleri* | Araha.53282s0001 |  | *Malus domestica* | MDP0000609638 |
| *Arabidopsis halleri* | Araha.53482s0001 |  | *Malus domestica* | MDP0000713113 |
| *Arabidopsis halleri* | Araha.6366s0014 |  | *Malus domestica* | MDP0000782323 |
| *Arabidopsis halleri* | Araha.6503s0003 |  | *Malus domestica* | MDP0000815065 |
| *Arabidopsis halleri* | Araha.6834s0003 |  | *Malus domestica* | MDP0000917419 |
| *Arabidopsis halleri* | Araha.71287s0001 |  | *Manihot esculenta* | Manes.01G012000 |
| *Arabidopsis halleri* | Araha.73009s0001 |  | *Manihot esculenta* | Manes.01G024100 |
| *Arabidopsis halleri* | Araha.7679s0002 |  | *Manihot esculenta* | Manes.01G043200 |
| *Arabidopsis halleri* | Araha.7888s0002 |  | *Manihot esculenta* | Manes.01G060500 |
| *Arabidopsis halleri* | Araha.8306s0003 |  | *Manihot esculenta* | Manes.01G106200 |
| *Arabidopsis halleri* | Araha.8989s0003 |  | *Manihot esculenta* | Manes.01G214400 |
| *Arabidopsis halleri* | Araha.9118s0005 |  | *Manihot esculenta* | Manes.01G239500 |
| *Arabidopsis lyrata* | AL1G14150 |  | *Manihot esculenta* | Manes.02G021800 |
| *Arabidopsis lyrata* | AL1G15070 |  | *Manihot esculenta* | Manes.02G062700 |
| *Arabidopsis lyrata* | AL1G17160 |  | *Manihot esculenta* | Manes.02G216600 |
| *Arabidopsis lyrata* | AL1G39860 |  | *Manihot esculenta* | Manes.03G006000 |
| *Arabidopsis lyrata* | AL1G41560 |  | *Manihot esculenta* | Manes.03G159600 |
| *Arabidopsis lyrata* | AL1G55990 |  | *Manihot esculenta* | Manes.04G027400 |
| *Arabidopsis lyrata* | AL1G59510 |  | *Manihot esculenta* | Manes.05G016200 |
| *Arabidopsis lyrata* | AL2G11320 |  | *Manihot esculenta* | Manes.05G137600 |
| *Arabidopsis lyrata* | AL2G27420 |  | *Manihot esculenta* | Manes.05G189500 |
| *Arabidopsis lyrata* | AL2G33690 |  | *Manihot esculenta* | Manes.05G189600 |
| *Arabidopsis lyrata* | AL39U10080 |  | *Manihot esculenta* | Manes.06G019300 |
| *Arabidopsis lyrata* | AL3G11680 |  | *Manihot esculenta* | Manes.06G028200 |
| *Arabidopsis lyrata* | AL3G19010 |  | *Manihot esculenta* | Manes.06G068600 |
| *Arabidopsis lyrata* | AL3G24560 |  | *Manihot esculenta* | Manes.06G089800 |
| *Arabidopsis lyrata* | AL3G35170 |  | *Manihot esculenta* | Manes.06G159900 |
| *Arabidopsis lyrata* | AL3G36066 |  | *Manihot esculenta* | Manes.06G173600 |
| *Arabidopsis lyrata* | AL4G15780 |  | *Manihot esculenta* | Manes.07G041200 |
| *Arabidopsis lyrata* | AL4G16370 |  | *Manihot esculenta* | Manes.07G041300 |
| *Arabidopsis lyrata* | AL4G29000 |  | *Manihot esculenta* | Manes.08G001600 |
| *Arabidopsis lyrata* | AL4G29170 |  | *Manihot esculenta* | Manes.08G103500 |
| *Arabidopsis lyrata* | AL4G46260 |  | *Manihot esculenta* | Manes.09G185700 |
| *Arabidopsis lyrata* | AL4G47470 |  | *Manihot esculenta* | Manes.10G097400 |
| *Arabidopsis lyrata* | AL6G12070 |  | *Manihot esculenta* | Manes.11G079100 |
| *Arabidopsis lyrata* | AL6G25080 |  | *Manihot esculenta* | Manes.11G101000 |
| *Arabidopsis lyrata* | AL6G26710 |  | *Manihot esculenta* | Manes.11G137200 |
| *Arabidopsis lyrata* | AL6G26730 |  | *Manihot esculenta* | Manes.13G059800 |
| *Arabidopsis lyrata* | AL6G36050 |  | *Manihot esculenta* | Manes.13G073500 |
| *Arabidopsis lyrata* | AL6G36600 |  | *Manihot esculenta* | Manes.13G091300 |
| *Arabidopsis lyrata* | AL7G24730 |  | *Manihot esculenta* | Manes.14G006300 |
| *Arabidopsis lyrata* | AL7G27010 |  | *Manihot esculenta* | Manes.14G012100 |
| *Arabidopsis lyrata* | AL7G28750 |  | *Manihot esculenta* | Manes.14G081500 |
| *Arabidopsis lyrata* | AL7G40360 |  | *Manihot esculenta* | Manes.14G102400 |
| *Arabidopsis lyrata* | AL7G48040 |  | *Manihot esculenta* | Manes.14G161500 |
| *Arabidopsis lyrata* | AL8G20980 |  | *Manihot esculenta* | Manes.15G045300 |
| *Arabidopsis lyrata* | AL8G20990 |  | *Manihot esculenta* | Manes.15G154300 |
| *Arabidopsis lyrata* | AL8G23580 |  | *Manihot esculenta* | Manes.16G133200 |
| *Arabidopsis lyrata* | AL8G28120 |  | *Manihot esculenta* | Manes.18G056300 |
| *Arabidopsis lyrata* | AL8G33110 |  | *Manihot esculenta* | Manes.18G056400 |
| *Arabidopsis lyrata* | AL8G33690 |  | *Manihot esculenta* | Manes.S099400 |
| *Arabidopsis lyrata* | AL8G36560 |  | *Medicago truncatula* | Medtr1g008220 |
| *Arabidopsis lyrata* | AL8G36680 |  | *Medicago truncatula* | Medtr1g013450 |
| *Arabidopsis lyrata* | AL8G38050 |  | *Medicago truncatula* | Medtr1g044785 |
| *Arabidopsis thaliana* | AT1G04500 |  | *Medicago truncatula* | Medtr1g067110 |
| *Arabidopsis thaliana* | AT1G05290 |  | *Medicago truncatula* | Medtr1g073350 |
| *Arabidopsis thaliana* | AT1G07050 |  | *Medicago truncatula* | Medtr1g110870 |
| *Arabidopsis thaliana* | AT1G25440 |  | *Medicago truncatula* | Medtr2g068730 |
| *Arabidopsis thaliana* | AT1G28050 |  | *Medicago truncatula* | Medtr2g088900 |
| *Arabidopsis thaliana* | AT1G49130 |  | *Medicago truncatula* | Medtr2g096080 |
| *Arabidopsis thaliana* | AT1G51600 |  | *Medicago truncatula* | Medtr3g037390 |
| *Arabidopsis thaliana* | AT1G63820 |  | *Medicago truncatula* | Medtr3g082630 |
| *Arabidopsis thaliana* | AT1G68520 |  | *Medicago truncatula* | Medtr3g091340 |
| *Arabidopsis thaliana* | AT1G73870 |  | *Medicago truncatula* | Medtr3g092780 |
| *Arabidopsis thaliana* | AT2G24790 |  | *Medicago truncatula* | Medtr3g100040 |
| *Arabidopsis thaliana* | AT2G32310 |  | *Medicago truncatula* | Medtr3g100050 |
| *Arabidopsis thaliana* | AT2G33350 |  | *Medicago truncatula* | Medtr3g105710 |
| *Arabidopsis thaliana* | AT2G33500 |  | *Medicago truncatula* | Medtr4g008090 |
| *Arabidopsis thaliana* | AT2G46670 |  | *Medicago truncatula* | Medtr4g061360 |
| *Arabidopsis thaliana* | AT2G46790 |  | *Medicago truncatula* | Medtr4g061823 |
| *Arabidopsis thaliana* | AT2G47890 |  | *Medicago truncatula* | Medtr4g061910 |
| *Arabidopsis thaliana* | AT3G02380 |  | *Medicago truncatula* | Medtr4g093730 |
| *Arabidopsis thaliana* | AT3G07650 |  | *Medicago truncatula* | Medtr4g108880 |
| *Arabidopsis thaliana* | AT3G12890 |  | *Medicago truncatula* | Medtr4g127420 |
| *Arabidopsis thaliana* | AT3G21175 |  | *Medicago truncatula* | Medtr4g128930 |
| *Arabidopsis thaliana* | AT3G21880 |  | *Medicago truncatula* | Medtr5g010120 |
| *Arabidopsis thaliana* | AT4G15250 |  | *Medicago truncatula* | Medtr5g066510 |
| *Arabidopsis thaliana* | AT4G24470 |  | *Medicago truncatula* | Medtr5g069480 |
| *Arabidopsis thaliana* | AT4G25990 |  | *Medicago truncatula* | Medtr5g072780 |
| *Arabidopsis thaliana* | AT4G27900 |  | *Medicago truncatula* | Medtr7g018170 |
| *Arabidopsis thaliana* | AT5G02810 |  | *Medicago truncatula* | Medtr7g032240 |
| *Arabidopsis thaliana* | AT5G14370 |  | *Medicago truncatula* | Medtr7g083540 |
| *Arabidopsis thaliana* | AT5G15840 |  | *Medicago truncatula* | Medtr7g108150 |
| *Arabidopsis thaliana* | AT5G15850 |  | *Medicago truncatula* | Medtr7g118260 |
| *Arabidopsis thaliana* | AT5G24470 |  | *Medicago truncatula* | Medtr8g024260 |
| *Arabidopsis thaliana* | AT5G24930 |  | *Medicago truncatula* | Medtr8g098725 |
| *Arabidopsis thaliana* | AT5G41380 |  | *Medicago truncatula* | Medtr8g104190 |
| *Arabidopsis thaliana* | AT5G48250 |  | *Mimulus guttatus* | Migut.A00403 |
| *Arabidopsis thaliana* | AT5G53420 |  | *Mimulus guttatus* | Migut.A00747 |
| *Arabidopsis thaliana* | AT5G57180 |  | *Mimulus guttatus* | Migut.B00065 |
| *Arabidopsis thaliana* | AT5G57660 |  | *Mimulus guttatus* | Migut.B00543 |
| *Arabidopsis thaliana* | AT5G59990 |  | *Mimulus guttatus* | Migut.D00173 |
| *Arabidopsis thaliana* | AT5G60100 |  | *Mimulus guttatus* | Migut.D01764 |
| *Arabidopsis thaliana* | AT5G61380 |  | *Mimulus guttatus* | Migut.D02156 |
| *Asparagus officinalis* | evm.model.AsparagusV1_01.1140 | | *Mimulus guttatus* | Migut.E00340 |
| *Asparagus officinalis* | evm.model.AsparagusV1_01.3029 | | *Mimulus guttatus* | Migut.E01434 |
| *Asparagus officinalis* | evm.model.AsparagusV1_01.519 | | *Mimulus guttatus* | Migut.F00152 |
| *Asparagus officinalis* | evm.model.AsparagusV1_01.529 | | *Mimulus guttatus* | Migut.F00223 |
| *Asparagus officinalis* | evm.model.AsparagusV1_01.628 | | *Mimulus guttatus* | Migut.F00987 |
| *Asparagus officinalis* | evm.model.AsparagusV1_01.639 | | *Mimulus guttatus* | Migut.F01036 |
| *Asparagus officinalis* | evm.model.AsparagusV1_01.828 | | *Mimulus guttatus* | Migut.G00546 |
| *Asparagus officinalis* | evm.model.AsparagusV1_03.1549 | | *Mimulus guttatus* | Migut.H00566 |
| *Asparagus officinalis* | evm.model.AsparagusV1_03.1641 | | *Mimulus guttatus* | Migut.H01218 |
| *Asparagus officinalis* | evm.model.AsparagusV1_03.2672 | | *Mimulus guttatus* | Migut.I00209 |
| *Asparagus officinalis* | evm.model.AsparagusV1_04.1537 | | *Mimulus guttatus* | Migut.I00345 |
| *Asparagus officinalis* | evm.model.AsparagusV1_04.3307 | | *Mimulus guttatus* | Migut.I01184 |
| *Asparagus officinalis* | evm.model.AsparagusV1_04.594 | | *Mimulus guttatus* | Migut.J00402 |
| *Asparagus officinalis* | evm.model.AsparagusV1_05.1978 | | *Mimulus guttatus* | Migut.J00797 |
| *Asparagus officinalis* | evm.model.AsparagusV1_05.2184 | | *Mimulus guttatus* | Migut.K00262 |
| *Asparagus officinalis* | evm.model.AsparagusV1_05.2270 | | *Mimulus guttatus* | Migut.K01031 |
| *Asparagus officinalis* | evm.model.AsparagusV1_05.2387 | | *Mimulus guttatus* | Migut.K01424 |
| *Asparagus officinalis* | evm.model.AsparagusV1_05.3527 | | *Mimulus guttatus* | Migut.L01064 |
| *Asparagus officinalis* | evm.model.AsparagusV1_06.495 | | *Mimulus guttatus* | Migut.L01650 |
| *Asparagus officinalis* | evm.model.AsparagusV1_07.1501 | | *Mimulus guttatus* | Migut.L01879 |
| *Asparagus officinalis* | evm.model.AsparagusV1_07.2039 | | *Mimulus guttatus* | Migut.M00064 |
| *Asparagus officinalis* | evm.model.AsparagusV1_07.342 | | *Mimulus guttatus* | Migut.M01461 |
| *Asparagus officinalis* | evm.model.AsparagusV1_09.122 | | *Mimulus guttatus* | Migut.M01547 |
| *Asparagus officinalis* | evm.model.AsparagusV1_10.1896 | | *Mimulus guttatus* | Migut.N00298 |
| *Asparagus officinalis* | evm.model.AsparagusV1_10.448 | | *Mimulus guttatus* | Migut.N01100 |
| *Asparagus officinalis* | evm.model.supercontig_1.291 | | *Miscanthus sinensis* | Misin01G107300 |
| *Asparagus officinalis* | evm.model.supercontig_123.53 | | *Miscanthus sinensis* | Misin01G124800 |
| *Asparagus officinalis* | evm.model.supercontig_1332.1 | | *Miscanthus sinensis* | Misin01G207800 |
| *Asparagus officinalis* | evm.model.supercontig_136.56 | | *Miscanthus sinensis* | Misin01G282300 |
| *Asparagus officinalis* | evm.model.supercontig_139.32 | | *Miscanthus sinensis* | Misin01G392400 |
| *Asparagus officinalis* | evm.model.supercontig_155.28 | | *Miscanthus sinensis* | Misin01G488500 |
| *Asparagus officinalis* | evm.model.supercontig_180.14 | | *Miscanthus sinensis* | Misin02G079400 |
| *Asparagus officinalis* | evm.model.supercontig_19.26 | | *Miscanthus sinensis* | Misin02G095900 |
| *Asparagus officinalis* | evm.model.supercontig_193.20 | | *Miscanthus sinensis* | Misin02G124000 |
| *Asparagus officinalis* | evm.model.supercontig_2.373 | | *Miscanthus sinensis* | Misin02G189700 |
| *Asparagus officinalis* | evm.model.supercontig_20.16 | | *Miscanthus sinensis* | Misin02G347500 |
| *Asparagus officinalis* | evm.model.supercontig_3.152 | | *Miscanthus sinensis* | Misin02G387600 |
| *Asparagus officinalis* | evm.model.supercontig_3.516 | | *Miscanthus sinensis* | Misin03G237600 |
| *Asparagus officinalis* | evm.model.supercontig_3.74 | | *Miscanthus sinensis* | Misin03G354800 |
| *Asparagus officinalis* | evm.model.supercontig_3189.2 | | *Miscanthus sinensis* | Misin04G258200 |
| *Asparagus officinalis* | evm.model.supercontig_331.5 | | *Miscanthus sinensis* | Misin04G387300 |
| *Asparagus officinalis* | evm.model.supercontig_44.6 | | *Miscanthus sinensis* | Misin05G345900 |
| *Asparagus officinalis* | evm.model.supercontig_70.6 | | *Miscanthus sinensis* | Misin06G314500 |
| *Asparagus officinalis* | evm.model.supercontig_8.239 | | *Miscanthus sinensis* | Misin07G008600 |
| *Asparagus officinalis* | evm.model.supercontig_862.1 | | *Miscanthus sinensis* | Misin07G039200 |
| *Asparagus officinalis* | evm.model.supercontig_9.175 | | *Miscanthus sinensis* | Misin07G060800 |
| *Asparagus officinalis* | evm.model.supercontig_95.76 | | *Miscanthus sinensis* | Misin07G150300 |
| *Asparagus officinalis* | evm.model.supercontig_95.8 | | *Miscanthus sinensis* | Misin07G188800 |
| *Boechera stricta* | Bostr.0597s0159 |  | *Miscanthus sinensis* | Misin07G226100 |
| *Boechera stricta* | Bostr.12659s0412 |  | *Miscanthus sinensis* | Misin07G264800 |
| *Boechera stricta* | Bostr.13083s0065 |  | *Miscanthus sinensis* | Misin07G407000 |
| *Boechera stricta* | Bostr.13208s0028 |  | *Miscanthus sinensis* | Misin07G408500 |
| *Boechera stricta* | Bostr.1460s0153 |  | *Miscanthus sinensis* | Misin07G493800 |
| *Boechera stricta* | Bostr.15697s0122 |  | *Miscanthus sinensis* | Misin07G496400 |
| *Boechera stricta* | Bostr.15774s0100 |  | *Miscanthus sinensis* | Misin08G004400 |
| *Boechera stricta* | Bostr.16926s0001 |  | *Miscanthus sinensis* | Misin08G038300 |
| *Boechera stricta* | Bostr.19424s0458 |  | *Miscanthus sinensis* | Misin08G063000 |
| *Boechera stricta* | Bostr.2128s0165 |  | *Miscanthus sinensis* | Misin08G196200 |
| *Boechera stricta* | Bostr.22252s0262 |  | *Miscanthus sinensis* | Misin08G202400 |
| *Boechera stricta* | Bostr.23794s0751 |  | *Miscanthus sinensis* | Misin08G266500 |
| *Boechera stricta* | Bostr.23794s0768 |  | *Miscanthus sinensis* | Misin08G284300 |
| *Boechera stricta* | Bostr.25219s0252 |  | *Miscanthus sinensis* | Misin09G036300 |
| *Boechera stricta* | Bostr.25219s0441 |  | *Miscanthus sinensis* | Misin11G003800 |
| *Boechera stricta* | Bostr.25993s0087 |  | *Miscanthus sinensis* | Misin11G129200 |
| *Boechera stricta* | Bostr.26675s0048 |  | *Miscanthus sinensis* | Misin12G136600 |
| *Boechera stricta* | Bostr.26675s0264 |  | *Miscanthus sinensis* | Misin13G016500 |
| *Boechera stricta* | Bostr.26675s0299 |  | *Miscanthus sinensis* | Misin13G071800 |
| *Boechera stricta* | Bostr.26833s0102 |  | *Miscanthus sinensis* | Misin13G168000 |
| *Boechera stricta* | Bostr.26833s0225 |  | *Miscanthus sinensis* | Misin14G005300 |
| *Boechera stricta* | Bostr.26833s0236 |  | *Miscanthus sinensis* | Misin16G168400 |
| *Boechera stricta* | Bostr.26833s0489 |  | *Miscanthus sinensis* | Misin16G262800 |
| *Boechera stricta* | Bostr.26833s0540 |  | *Miscanthus sinensis* | Misin17G178200 |
| *Boechera stricta* | Bostr.26833s0940 |  | *Miscanthus sinensis* | Misin17G178300 |
| *Boechera stricta* | Bostr.26959s0039 |  | *Miscanthus sinensis* | Misin17G267300 |
| *Boechera stricta* | Bostr.2902s0174 |  | *Miscanthus sinensis* | Misin18G116100 |
| *Boechera stricta* | Bostr.2902s0319 |  | *Miscanthus sinensis* | Misin18G123200 |
| *Boechera stricta* | Bostr.2902s0320 |  | *Miscanthus sinensis* | Misin18G133200 |
| *Boechera stricta* | Bostr.3288s0031 |  | *Miscanthus sinensis* | Misin18G212400 |
| *Boechera stricta* | Bostr.3640s0062 |  | *Miscanthus sinensis* | Misin18G250300 |
| *Boechera stricta* | Bostr.5022s0134 |  | *Miscanthus sinensis* | Misin19G004800 |
| *Boechera stricta* | Bostr.5763s0069 |  | *Miscanthus sinensis* | Misin19G117100 |
| *Boechera stricta* | Bostr.7200s0151 |  | *Miscanthus sinensis* | Misin19G121300 |
| *Boechera stricta* | Bostr.7867s0254 |  | *Miscanthus sinensis* | Misin19G128500 |
| *Boechera stricta* | Bostr.7867s0417 |  | *Miscanthus sinensis* | Misin19G195000 |
| *Boechera stricta* | Bostr.7867s0621 |  | *Miscanthus sinensis* | Misin19G240800 |
| *Boechera stricta* | Bostr.8705s0012 |  | *Miscanthus sinensis* | MisinT021600 |
| *Brachypodium distachyon* | Bradi1g09550 |  | *Miscanthus sinensis* | MisinT053700 |
| *Brachypodium distachyon* | Bradi1g11310 |  | *Miscanthus sinensis* | MisinT116100 |
| *Brachypodium distachyon* | Bradi1g12330 |  | *Miscanthus sinensis* | MisinT348300 |
| *Brachypodium distachyon* | Bradi1g16490 |  | *Miscanthus sinensis* | MisinT408800 |
| *Brachypodium distachyon* | Bradi1g18407 |  | *Miscanthus sinensis* | MisinT440100 |
| *Brachypodium distachyon* | Bradi1g31280 |  | *Miscanthus sinensis* | MisinT447800 |
| *Brachypodium distachyon* | Bradi1g33980 |  | *Musa acuminata* | GSMUA_Achr10P03960_001 |
| *Brachypodium distachyon* | Bradi1g34060 |  | *Musa acuminata* | GSMUA_Achr10P05820_001 |
| *Brachypodium distachyon* | Bradi1g43220 |  | *Musa acuminata* | GSMUA_Achr10P06260_001 |
| *Brachypodium distachyon* | Bradi1g43670 |  | *Musa acuminata* | GSMUA_Achr10P06310_001 |
| *Brachypodium distachyon* | Bradi1g43990 |  | *Musa acuminata* | GSMUA_Achr10P07970_001 |
| *Brachypodium distachyon* | Bradi1g52360 |  | *Musa acuminata* | GSMUA_Achr10P15340_001 |
| *Brachypodium distachyon* | Bradi1g62420 |  | *Musa acuminata* | GSMUA_Achr10P23700_001 |
| *Brachypodium distachyon* | Bradi1g65910 |  | *Musa acuminata* | GSMUA_Achr10P26840_001 |
| *Brachypodium distachyon* | Bradi1g75760 |  | *Musa acuminata* | GSMUA_Achr10P26880_001 |
| *Brachypodium distachyon* | Bradi2g14220 |  | *Musa acuminata* | GSMUA_Achr10P29990_001 |
| *Brachypodium distachyon* | Bradi2g22800 |  | *Musa acuminata* | GSMUA_Achr10P31360_001 |
| *Brachypodium distachyon* | Bradi2g54260 |  | *Musa acuminata* | GSMUA_Achr11P02540_001 |
| *Brachypodium distachyon* | Bradi3g03770 |  | *Musa acuminata* | GSMUA_Achr11P08210_001 |
| *Brachypodium distachyon* | Bradi3g03800 |  | *Musa acuminata* | GSMUA_Achr11P08470_001 |
| *Brachypodium distachyon* | Bradi3g03810 |  | *Musa acuminata* | GSMUA_Achr11P08750_001 |
| *Brachypodium distachyon* | Bradi3g05800 |  | *Musa acuminata* | GSMUA_Achr1P23420_001 |
| *Brachypodium distachyon* | Bradi3g10010 |  | *Musa acuminata* | GSMUA_Achr2P04520_001 |
| *Brachypodium distachyon* | Bradi3g13960 |  | *Musa acuminata* | GSMUA_Achr2P04570_001 |
| *Brachypodium distachyon* | Bradi3g19010 |  | *Musa acuminata* | GSMUA_Achr2P04960_001 |
| *Brachypodium distachyon* | Bradi3g28290 |  | *Musa acuminata* | GSMUA_Achr2P06760_001 |
| *Brachypodium distachyon* | Bradi3g41500 |  | *Musa acuminata* | GSMUA_Achr2P15290_001 |
| *Brachypodium distachyon* | Bradi3g48447 |  | *Musa acuminata* | GSMUA_Achr2P16090_001 |
| *Brachypodium distachyon* | Bradi3g48880 |  | *Musa acuminata* | GSMUA_Achr3P04260_001 |
| *Brachypodium distachyon* | Bradi3g56260 |  | *Musa acuminata* | GSMUA_Achr3P05620_001 |
| *Brachypodium distachyon* | Bradi3g56490 |  | *Musa acuminata* | GSMUA_Achr3P08880_001 |
| *Brachypodium distachyon* | Bradi3g57000 |  | *Musa acuminata* | GSMUA_Achr3P14530_001 |
| *Brachypodium distachyon* | Bradi4g15720 |  | *Musa acuminata* | GSMUA_Achr3P28130_001 |
| *Brachypodium distachyon* | Bradi4g24967 |  | *Musa acuminata* | GSMUA_Achr4P04970_001 |
| *Brachypodium distachyon* | Bradi4g36077 |  | *Musa acuminata* | GSMUA_Achr4P05170_001 |
| *Brachypodium distachyon* | Bradi4g45327 |  | *Musa acuminata* | GSMUA_Achr4P16810_001 |
| *Brachypodium distachyon* | Bradi4g45330 |  | *Musa acuminata* | GSMUA_Achr4P18680_001 |
| *Brachypodium distachyon* | Bradi5g14600 |  | *Musa acuminata* | GSMUA_Achr4P26570_001 |
| *Brachypodium distachyon Bd21-3* | BdiBd21-3.1G0129900 | | *Musa acuminata* | GSMUA_Achr4P30370_001 |
| *Brachypodium distachyon Bd21-3* | BdiBd21-3.1G0151100 | | *Musa acuminata* | GSMUA_Achr4P30600_001 |
| *Brachypodium distachyon Bd21-3* | BdiBd21-3.1G0164700 | | *Musa acuminata* | GSMUA_Achr4P30670_001 |
| *Brachypodium distachyon Bd21-3* | BdiBd21-3.1G0218200 | | *Musa acuminata* | GSMUA_Achr4P31170_001 |
| *Brachypodium distachyon Bd21-3* | BdiBd21-3.1G0240500 | | *Musa acuminata* | GSMUA_Achr5P04270_001 |
| *Brachypodium distachyon Bd21-3* | BdiBd21-3.1G0419300 | | *Musa acuminata* | GSMUA_Achr5P12990_001 |
| *Brachypodium distachyon Bd21-3* | BdiBd21-3.1G0458800 | | *Musa acuminata* | GSMUA_Achr5P13470_001 |
| *Brachypodium distachyon Bd21-3* | BdiBd21-3.1G0459600 | | *Musa acuminata* | GSMUA_Achr5P15400_001 |
| *Brachypodium distachyon Bd21-3* | BdiBd21-3.1G0561000 | | *Musa acuminata* | GSMUA_Achr5P15710_001 |
| *Brachypodium distachyon Bd21-3* | BdiBd21-3.1G0567700 | | *Musa acuminata* | GSMUA_Achr5P20450_001 |
| *Brachypodium distachyon Bd21-3* | BdiBd21-3.1G0573000 | | *Musa acuminata* | GSMUA_Achr5P22460_001 |
| *Brachypodium distachyon Bd21-3* | BdiBd21-3.1G0696100 | | *Musa acuminata* | GSMUA_Achr6P01780_001 |
| *Brachypodium distachyon Bd21-3* | BdiBd21-3.1G0843700 | | *Musa acuminata* | GSMUA_Achr6P04510_001 |
| *Brachypodium distachyon Bd21-3* | BdiBd21-3.1G0887100 | | *Musa acuminata* | GSMUA_Achr6P05120_001 |
| *Brachypodium distachyon Bd21-3* | BdiBd21-3.1G1011500 | | *Musa acuminata* | GSMUA_Achr6P06130_001 |
| *Brachypodium distachyon Bd21-3* | BdiBd21-3.2G0187900 | | *Musa acuminata* | GSMUA_Achr6P26640_001 |
| *Brachypodium distachyon Bd21-3* | BdiBd21-3.2G0299800 | | *Musa acuminata* | GSMUA_Achr7P01650_001 |
| *Brachypodium distachyon Bd21-3* | BdiBd21-3.2G0694200 | | *Musa acuminata* | GSMUA_Achr7P10370_001 |
| *Brachypodium distachyon Bd21-3* | BdiBd21-3.3G0047300 | | *Musa acuminata* | GSMUA_Achr7P12560_001 |
| *Brachypodium distachyon Bd21-3* | BdiBd21-3.3G0047600 | | *Musa acuminata* | GSMUA_Achr7P16370_001 |
| *Brachypodium distachyon Bd21-3* | BdiBd21-3.3G0047700 | | *Musa acuminata* | GSMUA_Achr7P18710_001 |
| *Brachypodium distachyon Bd21-3* | BdiBd21-3.3G0075200 | | *Musa acuminata* | GSMUA_Achr7P19660_001 |
| *Brachypodium distachyon Bd21-3* | BdiBd21-3.3G0136000 | | *Musa acuminata* | GSMUA_Achr7P20150_001 |
| *Brachypodium distachyon Bd21-3* | BdiBd21-3.3G0184300 | | *Musa acuminata* | GSMUA_Achr8P01990_001 |
| *Brachypodium distachyon Bd21-3* | BdiBd21-3.3G0264400 | | *Musa acuminata* | GSMUA_Achr8P12620_001 |
| *Brachypodium distachyon Bd21-3* | BdiBd21-3.3G0367700 | | *Musa acuminata* | GSMUA_Achr8P27500_001 |
| *Brachypodium distachyon Bd21-3* | BdiBd21-3.3G0548600 | | *Musa acuminata* | GSMUA_Achr9P04240_001 |
| *Brachypodium distachyon Bd21-3* | BdiBd21-3.3G0642200 | | *Musa acuminata* | GSMUA_Achr9P06150_001 |
| *Brachypodium distachyon Bd21-3* | BdiBd21-3.3G0649000 | | *Musa acuminata* | GSMUA_Achr9P08940_001 |
| *Brachypodium distachyon Bd21-3* | BdiBd21-3.3G0744500 | | *Musa acuminata* | GSMUA_Achr9P12860_001 |
| *Brachypodium distachyon Bd21-3* | BdiBd21-3.3G0746900 | | *Musa acuminata* | GSMUA_Achr9P13870_001 |
| *Brachypodium distachyon Bd21-3* | BdiBd21-3.3G0753700 | | *Musa acuminata* | GSMUA_Achr9P19550_001 |
| *Brachypodium distachyon Bd21-3* | BdiBd21-3.4G0227400 | | *Musa acuminata* | GSMUA_Achr9P19630_001 |
| *Brachypodium distachyon Bd21-3* | BdiBd21-3.4G0353400 | | *Musa acuminata* | GSMUA_Achr9P22690_001 |
| *Brachypodium distachyon Bd21-3* | BdiBd21-3.4G0502400 | | *Musa acuminata* | GSMUA_Achr9P24280_001 |
| *Brachypodium distachyon Bd21-3* | BdiBd21-3.4G0628000 | | *Musa acuminata* | GSMUA_Achr9P28950_001 |
| *Brachypodium distachyon Bd21-3* | BdiBd21-3.4G0628100 | | *Musa acuminata* | GSMUA_Achr9P28960_001 |
| *Brachypodium distachyon Bd21-3* | BdiBd21-3.5G0187400 | | *Musa acuminata* | GSMUA_AchrUn_randomP10310_001 |
| *Brachypodium hybridum* | Brahy.S01G0103100 |  | *Musa acuminata* | GSMUA_AchrUn_randomP18110_001 |
| *Brachypodium hybridum* | Brahy.S02G0037500 |  | *Olea europaea* | Oeu003282 |
| *Brachypodium hybridum* | Brahy.S02G0146600 |  | *Olea europaea* | Oeu006383 |
| *Brachypodium hybridum* | Brahy.S02G0191300 |  | *Olea europaea* | Oeu006847 |
| *Brachypodium hybridum* | Brahy.S02G0283300 |  | *Olea europaea* | Oeu007103 |
| *Brachypodium hybridum* | Brahy.S02G0300500 |  | *Olea europaea* | Oeu007124 |
| *Brachypodium hybridum* | Brahy.S02G0318500 |  | *Olea europaea* | Oeu007741 |
| *Brachypodium hybridum* | Brahy.S03G0049500 |  | *Olea europaea* | Oeu007918 |
| *Brachypodium hybridum* | Brahy.S03G0072700 |  | *Olea europaea* | Oeu008760 |
| *Brachypodium hybridum* | Brahy.S03G0161900 |  | *Olea europaea* | Oeu010522 |
| *Brachypodium hybridum* | Brahy.S03G0218700 |  | *Olea europaea* | Oeu011083 |
| *Brachypodium hybridum* | Brahy.S03G0318300 |  | *Olea europaea* | Oeu011225 |
| *Brachypodium hybridum* | Brahy.S04G0045400 |  | *Olea europaea* | Oeu014161 |
| *Brachypodium hybridum* | Brahy.S04G0050500 |  | *Olea europaea* | Oeu014200 |
| *Brachypodium hybridum* | Brahy.S04G0053100 |  | *Olea europaea* | Oeu014501 |
| *Brachypodium hybridum* | Brahy.S04G0144000 |  | *Olea europaea* | Oeu018819 |
| *Brachypodium hybridum* | Brahy.S04G0150700 |  | *Olea europaea* | Oeu023759 |
| *Brachypodium hybridum* | Brahy.S04G0244400 |  | *Olea europaea* | Oeu025976 |
| *Brachypodium hybridum* | Brahy.S04G0301700 |  | *Olea europaea* | Oeu028593 |
| *Brachypodium hybridum* | Brahy.S04G0327900 |  | *Olea europaea* | Oeu029682 |
| *Brachypodium hybridum* | Brahy.S04G0328000 |  | *Olea europaea* | Oeu030283 |
| *Brachypodium hybridum* | Brahy.S04G0328300 |  | *Olea europaea* | Oeu033423 |
| *Brachypodium hybridum* | Brahy.S05G0019500 |  | *Olea europaea* | Oeu033426 |
| *Brachypodium hybridum* | Brahy.S05G0212900 |  | *Olea europaea* | Oeu033840 |
| *Brachypodium hybridum* | Brahy.S05G0329500 |  | *Olea europaea* | Oeu034246 |
| *Brachypodium hybridum* | Brahy.S05G0329600 |  | *Olea europaea* | Oeu034800 |
| *Brachypodium hybridum* | Brahy.S05G0330700 |  | *Olea europaea* | Oeu034816 |
| *Brachypodium hybridum* | Brahy.S06G0000500 |  | *Olea europaea* | Oeu036614 |
| *Brachypodium hybridum* | Brahy.S06G0020000 |  | *Olea europaea* | Oeu036992 |
| *Brachypodium hybridum* | Brahy.S07G0002400 |  | *Olea europaea* | Oeu037086 |
| *Brachypodium hybridum* | Brahy.S07G0103900 |  | *Olea europaea* | Oeu038068 |
| *Brachypodium hybridum* | Brahy.S07G0108700 |  | *Olea europaea* | Oeu039281 |
| *Brachypodium hybridum* | Brahy.S07G0115000 |  | *Olea europaea* | Oeu040265 |
| *Brachypodium hybridum* | Brahy.S07G0214900 |  | *Olea europaea* | Oeu040996 |
| *Brachypodium hybridum* | Brahy.S07G0215600 |  | *Olea europaea* | Oeu041316 |
| *Brachypodium hybridum* | Brahy.S07G0248500 |  | *Olea europaea* | Oeu045851 |
| *Brachypodium hybridum* | Brahy.S08G0002200 |  | *Olea europaea* | Oeu047002 |
| *Brachypodium hybridum* | Brahy.S08G0096500 |  | *Olea europaea* | Oeu048536 |
| *Brachypodium hybridum* | Brahy.S09G0134700 |  | *Olea europaea* | Oeu049644 |
| *Brachypodium hybridum* | Brahy.S10G0147800 |  | *Olea europaea* | Oeu050163 |
| *Brachypodium stacei* | Brast01G096800 |  | *Olea europaea* | Oeu050450 |
| *Brachypodium stacei* | Brast02G035400 |  | *Olea europaea* | Oeu051670 |
| *Brachypodium stacei* | Brast02G139800 |  | *Olea europaea* | Oeu054235 |
| *Brachypodium stacei* | Brast02G182000 |  | *Olea europaea* | Oeu054774 |
| *Brachypodium stacei* | Brast02G265300 |  | *Olea europaea* | Oeu055060 |
| *Brachypodium stacei* | Brast02G280600 |  | *Olea europaea* | Oeu055390 |
| *Brachypodium stacei* | Brast02G297100 |  | *Olea europaea* | Oeu057561 |
| *Brachypodium stacei* | Brast03G045900 |  | *Olea europaea* | Oeu058835 |
| *Brachypodium stacei* | Brast03G066800 |  | *Olea europaea* | Oeu059560 |
| *Brachypodium stacei* | Brast03G144100 |  | *Olea europaea* | Oeu061030 |
| *Brachypodium stacei* | Brast03G292100 |  | *Olea europaea* | Oeu061387 |
| *Brachypodium stacei* | Brast04G042300 |  | *Olea europaea* | Oeu062310 |
| *Brachypodium stacei* | Brast04G047300 |  | *Olea europaea* | Oeu063546 |
| *Brachypodium stacei* | Brast04G049800 |  | *Olea europaea* | Oeu064853 |
| *Brachypodium stacei* | Brast04G136200 |  | *Oropetium thomaeum* | Oropetium_20150105_00024A |
| *Brachypodium stacei* | Brast04G141500 |  | *Oropetium thomaeum* | Oropetium_20150105_00953A |
| *Brachypodium stacei* | Brast04G226800 |  | *Oropetium thomaeum* | Oropetium_20150105_02724A |
| *Brachypodium stacei* | Brast04G279200 |  | *Oropetium thomaeum* | Oropetium_20150105_06572A |
| *Brachypodium stacei* | Brast04G303200 |  | *Oropetium thomaeum* | Oropetium_20150105_08179A |
| *Brachypodium stacei* | Brast04G303300 |  | *Oropetium thomaeum* | Oropetium_20150105_12006A |
| *Brachypodium stacei* | Brast04G303800 |  | *Oropetium thomaeum* | Oropetium_20150105_13784A |
| *Brachypodium stacei* | Brast05G017900 |  | *Oropetium thomaeum* | Oropetium_20150105_13880A |
| *Brachypodium stacei* | Brast05G192200 |  | *Oropetium thomaeum* | Oropetium_20150105_14345A |
| *Brachypodium stacei* | Brast05G301100 |  | *Oropetium thomaeum* | Oropetium_20150105_14652A |
| *Brachypodium stacei* | Brast06G004700 |  | *Oropetium thomaeum* | Oropetium_20150105_14995A |
| *Brachypodium stacei* | Brast06G019400 |  | *Oropetium thomaeum* | Oropetium_20150105_15716A |
| *Brachypodium stacei* | Brast07G001900 |  | *Oropetium thomaeum* | Oropetium_20150105_16034A |
| *Brachypodium stacei* | Brast07G095900 |  | *Oropetium thomaeum* | Oropetium_20150105_16080A |
| *Brachypodium stacei* | Brast07G100700 |  | *Oropetium thomaeum* | Oropetium_20150105_16131A |
| *Brachypodium stacei* | Brast07G106300 |  | *Oropetium thomaeum* | Oropetium_20150105_16269A |
| *Brachypodium stacei* | Brast07G193100 |  | *Oropetium thomaeum* | Oropetium_20150105_16846A |
| *Brachypodium stacei* | Brast07G193700 |  | *Oropetium thomaeum* | Oropetium_20150105_18374A |
| *Brachypodium stacei* | Brast07G223600 |  | *Oropetium thomaeum* | Oropetium_20150105_18805A |
| *Brachypodium stacei* | Brast08G001700 |  | *Oropetium thomaeum* | Oropetium_20150105_19136A |
| *Brachypodium stacei* | Brast08G091200 |  | *Oropetium thomaeum* | Oropetium_20150105_19194A |
| *Brachypodium stacei* | Brast09G131600 |  | *Oropetium thomaeum* | Oropetium_20150105_19920A |
| *Brachypodium stacei* | Brast10G130700 |  | *Oropetium thomaeum* | Oropetium_20150105_20020A |
| *Brachypodium sylvaticum* | Brasy1G132500 |  | *Oropetium thomaeum* | Oropetium_20150105_20580A |
| *Brachypodium sylvaticum* | Brasy2G169500 |  | *Oropetium thomaeum* | Oropetium_20150105_23800A |
| *Brachypodium sylvaticum* | Brasy2G212700 |  | *Oryza sativa* | Os01g61900 |
| *Brachypodium sylvaticum* | Brasy2G288300 |  | *Oryza sativa* | Os02g01990 |
| *Brachypodium sylvaticum* | Brasy2G310000 |  | *Oryza sativa* | Os02g05470 |
| *Brachypodium sylvaticum* | Brasy2G328900 |  | *Oryza sativa* | Os02g05510 |
| *Brachypodium sylvaticum* | Brasy3G067300 |  | *Oryza sativa* | Os02g08150 |
| *Brachypodium sylvaticum* | Brasy3G097700 |  | *Oryza sativa* | Os02g39710 |
| *Brachypodium sylvaticum* | Brasy3G165900 |  | *Oryza sativa* | Os02g40510 |
| *Brachypodium sylvaticum* | Brasy3G325700 |  | *Oryza sativa* | Os02g49230 |
| *Brachypodium sylvaticum* | Brasy4G040900 |  | *Oryza sativa* | Os02g49880 |
| *Brachypodium sylvaticum* | Brasy4G047600 |  | *Oryza sativa* | Os03g04620 |
| *Brachypodium sylvaticum* | Brasy4G050300 |  | *Oryza sativa* | Os03g17570 |
| *Brachypodium sylvaticum* | Brasy4G119000 |  | *Oryza sativa* | Os03g22770 |
| *Brachypodium sylvaticum* | Brasy4G131900 |  | *Oryza sativa* | Os03g47970 |
| *Brachypodium sylvaticum* | Brasy4G150900 |  | *Oryza sativa* | Os03g50310 |
| *Brachypodium sylvaticum* | Brasy4G266100 |  | *Oryza sativa* | Os03g52450 |
| *Brachypodium sylvaticum* | Brasy4G348500 |  | *Oryza sativa* | Os04g42020 |
| *Brachypodium sylvaticum* | Brasy4G386700 |  | *Oryza sativa* | Os05g38990 |
| *Brachypodium sylvaticum* | Brasy4G386900 |  | *Oryza sativa* | Os05g51690 |
| *Brachypodium sylvaticum* | Brasy4G387300 |  | *Oryza sativa* | Os06g01340 |
| *Brachypodium sylvaticum* | Brasy5G134000 |  | *Oryza sativa* | Os06g15330 |
| *Brachypodium sylvaticum* | Brasy5G243300 |  | *Oryza sativa* | Os06g16370 |
| *Brachypodium sylvaticum* | Brasy5G402000 |  | *Oryza sativa* | Os06g19444 |
| *Brachypodium sylvaticum* | Brasy5G524000 |  | *Oryza sativa* | Os06g44450 |
| *Brachypodium sylvaticum* | Brasy5G524100 |  | *Oryza sativa* | Os06g48534 |
| *Brachypodium sylvaticum* | Brasy6G000600 |  | *Oryza sativa* | Os06g48610 |
| *Brachypodium sylvaticum* | Brasy6G024900 |  | *Oryza sativa* | Os07g15770 |
| *Brachypodium sylvaticum* | Brasy7G001800 |  | *Oryza sativa* | Os07g47140 |
| *Brachypodium sylvaticum* | Brasy7G105600 |  | *Oryza sativa* | Os07g49460 |
| *Brachypodium sylvaticum* | Brasy7G111900 |  | *Oryza sativa* | Os08g15050 |
| *Brachypodium sylvaticum* | Brasy7G116100 |  | *Oryza sativa* | Os08g42440 |
| *Brachypodium sylvaticum* | Brasy7G208800 |  | *Oryza sativa* | Os09g06464 |
| *Brachypodium sylvaticum* | Brasy8G002300 |  | *Oryza sativa* | Os09g33550 |
| *Brachypodium sylvaticum* | Brasy8G114900 |  | *Oryza sativa* | Os09g36220 |
| *Brachypodium sylvaticum* | Brasy9G048500 |  | *Oryza sativa* | Os10g32900 |
| *Brachypodium sylvaticum* | Brasy9G049200 |  | *Oryza sativa* | Os10g41100 |
| *Brachypodium sylvaticum* | Brasy9G197600 |  | *Oryza sativa* | Os11g01074 |
| *Brassica oleracea var. capitata* | Bol004903 |  | *Oryza sativa* | Os11g01100 |
| *Brassica oleracea* var*. capitata* | Bol007693 |  | *Oryza sativa* | Os11g05930 |
| *Brassica oleracea* var*. capitata* | Bol008165 |  | *Oryza sativa* | Os12g01080 |
| *Brassica oleracea* var*. capitata* | Bol008435 |  | *Oryza sativa* | Os12g01100 |
| *Brassica oleracea* var*. capitata* | Bol008920 |  | *Oryza sativa* | Os12g16160 |
| *Brassica oleracea* var*. capitata* | Bol009539 |  | *Oryza sativa Kitaake* | OsKitaake01g391100 |
| *Brassica oleracea* var*. capitata* | Bol010087 |  | *Oryza sativa Kitaake* | OsKitaake02g008400 |
| *Brassica oleracea* var*. capitata* | Bol010673 |  | *Oryza sativa Kitaake* | OsKitaake02g038500 |
| *Brassica oleracea* var*. capitata* | Bol011586 |  | *Oryza sativa Kitaake* | OsKitaake02g038900 |
| *Brassica oleracea* var*. capitata* | Bol012197 |  | *Oryza sativa Kitaake* | OsKitaake02g061400 |
| *Brassica oleracea* var*. capitata* | Bol012243 |  | *Oryza sativa Kitaake* | OsKitaake02g243100 |
| *Brassica oleracea* var*. capitata* | Bol014211 |  | *Oryza sativa Kitaake* | OsKitaake02g249300 |
| *Brassica oleracea* var*. capitata* | Bol014241 |  | *Oryza sativa Kitaake* | OsKitaake02g315600 |
| *Brassica oleracea* var*. capitata* | Bol015765 |  | *Oryza sativa Kitaake* | OsKitaake02g321800 |
| *Brassica oleracea* var*. capitata* | Bol016452 |  | *Oryza sativa Kitaake* | OsKitaake03g032500 |
| *Brassica oleracea* var*. capitata* | Bol017021 |  | *Oryza sativa Kitaake* | OsKitaake03g135300 |
| *Brassica oleracea* var*. capitata* | Bol017253 |  | *Oryza sativa Kitaake* | OsKitaake03g177500 |
| *Brassica oleracea* var*. capitata* | Bol017260 |  | *Oryza sativa Kitaake* | OsKitaake03g293400 |
| *Brassica oleracea* var*. capitata* | Bol018313 |  | *Oryza sativa Kitaake* | OsKitaake03g311400 |
| *Brassica oleracea* var*. capitata* | Bol018802 |  | *Oryza sativa Kitaake* | OsKitaake03g328800 |
| *Brassica oleracea* var*. capitata* | Bol021161 |  | *Oryza sativa Kitaake* | OsKitaake04g184400 |
| *Brassica oleracea* var*. capitata* | Bol021256 |  | *Oryza sativa Kitaake* | OsKitaake05g191000 |
| *Brassica oleracea* var*. capitata* | Bol021803 |  | *Oryza sativa Kitaake* | OsKitaake05g287200 |
| *Brassica oleracea* var*. capitata* | Bol022384 |  | *Oryza sativa Kitaake* | OsKitaake05g287400. |
| *Brassica oleracea* var*. capitata* | Bol022421 |  | *Oryza sativa Kitaake* | OsKitaake06g001300 |
| *Brassica oleracea* var*. capitata* | Bol023231 |  | *Oryza sativa Kitaake* | OsKitaake06g107200 |
| *Brassica oleracea* var*. capitata* | Bol023364 |  | *Oryza sativa Kitaake* | OsKitaake06g112900 |
| *Brassica oleracea* var*. capitata* | Bol024985 |  | *Oryza sativa Kitaake* | OsKitaake06g124900 |
| *Brassica oleracea* var*. capitata* | Bol026603 |  | *Oryza sativa Kitaake* | OsKitaake06g237800 |
| *Brassica oleracea* var*. capitata* | Bol027263 |  | *Oryza sativa Kitaake* | OsKitaake06g268600 |
| *Brassica oleracea* var*. capitata* | Bol027993 |  | *Oryza sativa Kitaake* | OsKitaake06g269200 |
| *Brassica oleracea* var*. capitata* | Bol028665 |  | *Oryza sativa Kitaake* | OsKitaake07g095000 |
| *Brassica oleracea* var*. capitata* | Bol030488 |  | *Oryza sativa Kitaake* | OsKitaake07g265600 |
| *Brassica oleracea* var*. capitata* | Bol030489 |  | *Oryza sativa Kitaake* | OsKitaake07g285400 |
| *Brassica oleracea* var*. capitata* | Bol033105 |  | *Oryza sativa Kitaake* | OsKitaake08g014500 |
| *Brassica oleracea* var*. capitata* | Bol034286 |  | *Oryza sativa Kitaake* | OsKitaake08g231500 |
| *Brassica oleracea* var*. capitata* | Bol035493 |  | *Oryza sativa Kitaake* | OsKitaake09g015300 |
| *Brassica oleracea* var*. capitata* | Bol036182 |  | *Oryza sativa Kitaake* | OsKitaake09g159700 |
| *Brassica oleracea* var*. capitata* | Bol036197 |  | *Oryza sativa Kitaake* | OsKitaake09g175500 |
| *Brassica oleracea* var*. capitata* | Bol036283 |  | *Oryza sativa Kitaake* | OsKitaake10g126300 |
| *Brassica oleracea* var*. capitata* | Bol037935 |  | *Oryza sativa Kitaake* | OsKitaake10g190900 |
| *Brassica oleracea* var*. capitata* | Bol038395 |  | *Oryza sativa Kitaake* | OsKitaake11g038800 |
| *Brassica oleracea* var*. capitata* | Bol039582 |  | *Oryza sativa Kitaake* | OsKitaake12g000700 |
| *Brassica oleracea* var*. capitata* | Bol040898 |  | *Oryza sativa Kitaake* | OsKitaake12g000900 |
| *Brassica oleracea* var*. capitata* | Bol040971 |  | *Oryza sativa Kitaake* | OsKitaake12g088200 |
| *Brassica oleracea* var*. capitata* | Bol041098 |  | *Panicum hallii* | Pahal.A00073.1 |
| *Brassica oleracea* var*. capitata* | Bol041805 |  | *Panicum hallii* | Pahal.A00280.1 |
| *Brassica oleracea* var*. capitata* | Bol042168 |  | *Panicum hallii* | Pahal.A00517.1 |
| *Brassica oleracea* var*. capitata* | Bol042263 |  | *Panicum hallii* | Pahal.A02488.1 |
| *Brassica oleracea* var*. capitata* | Bol043091 |  | *Panicum hallii* | Pahal.A02562.1 |
| *Brassica oleracea* var*. capitata* | Bol043150 |  | *Panicum hallii* | Pahal.A03213.1 |
| *Brassica oleracea* var*. capitata* | Bol043289 |  | *Panicum hallii* | Pahal.A03356.1 |
| *Brassica oleracea* var*. capitata* | Bol043295 |  | *Panicum hallii* | Pahal.B01360.1 |
| *Brassica oleracea* var*. capitata* | Bol043362 |  | *Panicum hallii* | Pahal.B02187.1 |
| *Brassica rapa* FPsc | Brara.A01452 |  | *Panicum hallii* | Pahal.B02603.1 |
| *Brassica rapa* FPsc | Brara.A01594 |  | *Panicum hallii* | Pahal.B02827.1 |
| *Brassica rapa* FPsc | Brara.A02750 |  | *Panicum hallii* | Pahal.B03611.1 |
| *Brassica rapa* FPsc | Brara.A02786 |  | *Panicum hallii* | Pahal.B04939.1 |
| *Brassica rapa* FPsc | Brara.A03306 |  | *Panicum hallii* | Pahal.C00061.1 |
| *Brassica rapa* FPsc | Brara.A03597 |  | *Panicum hallii* | Pahal.C01674.1 |
| *Brassica rapa* FPsc | Brara.A03895 |  | *Panicum hallii* | Pahal.C02661.1 |
| *Brassica rapa* FPsc | Brara.B00066 |  | *Panicum hallii* | Pahal.D00009.1 |
| *Brassica rapa* FPsc | Brara.B00548 |  | *Panicum hallii* | Pahal.D00662.1 |
| *Brassica rapa* FPsc | Brara.B00625 |  | *Panicum hallii* | Pahal.D01227.1 |
| *Brassica rapa* FPsc | Brara.B01053 |  | *Panicum hallii* | Pahal.D01761.1 |
| *Brassica rapa* FPsc | Brara.B01196 |  | *Panicum hallii* | Pahal.D02797.1 |
| *Brassica rapa* FPsc | Brara.B01229 |  | *Panicum hallii* | Pahal.E01052.1 |
| *Brassica rapa* FPsc | Brara.B01452 |  | *Panicum hallii* | Pahal.F00117.1 |
| *Brassica rapa* FPsc | Brara.B01797 |  | *Panicum hallii* | Pahal.F00582.1 |
| *Brassica rapa* FPsc | Brara.B03428 |  | *Panicum hallii* | Pahal.F01418.1 |
| *Brassica rapa* FPsc | Brara.B03680 |  | *Panicum hallii* | Pahal.G00773.1 |
| *Brassica rapa* FPsc | Brara.C00622 |  | *Panicum hallii* | Pahal.H00760.1 |
| *Brassica rapa* FPsc | Brara.C01392 |  | *Panicum hallii* | Pahal.H01416.1 |
| *Brassica rapa* FPsc | Brara.C02983 |  | *Panicum hallii* | Pahal.I00343.1 |
| *Brassica rapa* FPsc | Brara.C03223 |  | *Panicum hallii* | Pahal.I01844.1 |
| *Brassica rapa* FPsc | Brara.C03438 |  | *Panicum hallii* | Pahal.I02044.1 |
| *Brassica rapa* FPsc | Brara.C04252 |  | *Panicum hallii* | Pahal.I02227.1 |
| *Brassica rapa* FPsc | Brara.D01142 |  | *Panicum hallii* | Pahal.I03108.1 |
| *Brassica rapa* FPsc | Brara.D01502 |  | *Panicum hallii* | Pahal.I04041.1 |
| *Brassica rapa* FPsc | Brara.D02027 |  | *Panicum hallii* | Pahal.I04471.1 |
| *Brassica rapa* FPsc | Brara.E00008 |  | *Panicum hallii* | Pahal.J01115.1 |
| *Brassica rapa* FPsc | Brara.E00247 |  | *Panicum hallii HAL2* | PhHAL.1G006600 |
| *Brassica rapa* FPsc | Brara.E01059 |  | *Panicum hallii HAL2* | PhHAL.1G034100 |
| *Brassica rapa* FPsc | Brara.E01711 |  | *Panicum hallii HAL2* | PhHAL.1G057300 |
| *Brassica rapa* FPsc | Brara.E02096 |  | *Panicum hallii HAL2* | PhHAL.1G295800 |
| *Brassica rapa* FPsc | Brara.E02819 |  | *Panicum hallii HAL2* | PhHAL.1G302800 |
| *Brassica rapa* FPsc | Brara.E03186 |  | *Panicum hallii HAL2* | PhHAL.1G369300 |
| *Brassica rapa* FPsc | Brara.F02666 |  | *Panicum hallii HAL2* | PhHAL.1G383700 |
| *Brassica rapa* FPsc | Brara.F02700 |  | *Panicum hallii HAL2* | PhHAL.2G086000 |
| *Brassica rapa* FPsc | Brara.F03023 |  | *Panicum hallii HAL2* | PhHAL.2G333100 |
| *Brassica rapa* FPsc | Brara.G00824 |  | *Panicum hallii HAL2* | PhHAL.2G473800 |
| *Brassica rapa* FPsc | Brara.G00872 |  | *Panicum hallii HAL2* | PhHAL.3G001000 |
| *Brassica rapa* FPsc | Brara.G02520 |  | *Panicum hallii HAL2* | PhHAL.3G143500 |
| *Brassica rapa* FPsc | Brara.H00206 |  | *Panicum hallii HAL2* | PhHAL.3G237300 |
| *Brassica rapa* FPsc | Brara.H01505 |  | *Panicum hallii HAL2* | PhHAL.4G027300 |
| *Brassica rapa* FPsc | Brara.H02022 |  | *Panicum hallii HAL2* | PhHAL.4G062600 |
| *Brassica rapa* FPsc | Brara.H02107 |  | *Panicum hallii HAL2* | PhHAL.4G197100 |
| *Brassica rapa* FPsc | Brara.H02942 |  | *Panicum hallii HAL2* | PhHAL.4G237900 |
| *Brassica rapa* FPsc | Brara.H03045 |  | *Panicum hallii HAL2* | PhHAL.4G294200 |
| *Brassica rapa* FPsc | Brara.I00559 |  | *Panicum hallii HAL2* | PhHAL.4G370500 |
| *Brassica rapa* FPsc | Brara.I00569 |  | *Panicum hallii HAL2* | PhHAL.5G101900 |
| *Brassica rapa* FPsc | Brara.I00658 |  | *Panicum hallii HAL2* | PhHAL.6G018600 |
| *Brassica rapa* FPsc | Brara.I01379 |  | *Panicum hallii HAL2* | PhHAL.6G089300 |
| *Brassica rapa* FPsc | Brara.I02977 |  | *Panicum hallii HAL2* | PhHAL.6G269100 |
| *Brassica rapa* FPsc | Brara.I03083 |  | *Panicum hallii HAL2* | PhHAL.7G017000 |
| *Brassica rapa* FPsc | Brara.I05454 |  | *Panicum hallii HAL2* | PhHAL.7G198100 |
| *Brassica rapa* FPsc | Brara.J00262 |  | *Panicum hallii HAL2* | PhHAL.8G002100 |
| *Brassica rapa* FPsc | Brara.J00486 |  | *Panicum hallii HAL2* | PhHAL.8G033300 |
| *Brassica rapa* FPsc | Brara.J00842 |  | *Panicum hallii HAL2* | PhHAL.8G209800 |
| *Brassica rapa* FPsc | Brara.J01143 |  | *Panicum hallii HAL2* | PhHAL.9G095300 |
| *Brassica rapa* FPsc | Brara.J01176 |  | *Panicum hallii HAL2* | PhHAL.9G114600 |
| *Brassica rapa* FPsc | Brara.J01364 |  | *Panicum hallii HAL2* | PhHAL.9G130500 |
| *Brassica rapa* FPsc | Brara.J01369 |  | *Panicum hallii HAL2* | PhHAL.9G220400 |
| *Brassica rapa* FPsc | Brara.J01926 |  | *Panicum hallii HAL2* | PhHAL.9G400500 |
| *Brassica rapa* FPsc | Brara.J01927 |  | *Panicum hallii HAL2* | PhHAL.9G476200 |
| *Brassica rapa* FPsc | Brara.J02021 |  | *Panicum hallii HAL2* | PhHAL.9G516900 |
| *Brassica rapa* FPsc | Brara.J02854 |  | *Panicum hallii HAL2* | PhHAL.9G620700 |
| *Brassica rapa* FPsc | Brara.K00169 |  | *Panicum virgatum* | Pavir.1KG008500 |
| *Brassica rapa* FPsc | Brara.K01501 |  | *Panicum virgatum* | Pavir.1KG046300 |
| *Capsella grandiflora* | Cagra.0050s0021 |  | *Panicum virgatum* | Pavir.1KG080800 |
| *Capsella grandiflora* | Cagra.0084s0085 |  | *Panicum virgatum* | Pavir.1KG377300 |
| *Capsella grandiflora* | Cagra.0133s0112 |  | *Panicum virgatum* | Pavir.1KG385300 |
| *Capsella grandiflora* | Cagra.0301s0032 |  | *Panicum virgatum* | Pavir.1KG487100 |
| *Capsella grandiflora* | Cagra.0301s0048 |  | *Panicum virgatum* | Pavir.1NG007200 |
| *Capsella grandiflora* | Cagra.0542s0031 |  | *Panicum virgatum* | Pavir.1NG024700 |
| *Capsella grandiflora* | Cagra.0551s0042 |  | *Panicum virgatum* | Pavir.1NG038700 |
| *Capsella grandiflora* | Cagra.0876s0020 |  | *Panicum virgatum* | Pavir.1NG073400 |
| *Capsella grandiflora* | Cagra.1004s0003 |  | *Panicum virgatum* | Pavir.1NG342600 |
| *Capsella grandiflora* | Cagra.11928s0002 |  | *Panicum virgatum* | Pavir.1NG350900 |
| *Capsella grandiflora* | Cagra.1194s0065 |  | *Panicum virgatum* | Pavir.1NG463100 |
| *Capsella grandiflora* | Cagra.1225s0107 |  | *Panicum virgatum* | Pavir.2KG108900 |
| *Capsella grandiflora* | Cagra.1317s0007 |  | *Panicum virgatum* | Pavir.2KG163400 |
| *Capsella grandiflora* | Cagra.1317s0018 |  | *Panicum virgatum* | Pavir.2KG379300 |
| *Capsella grandiflora* | Cagra.14026s0004 |  | *Panicum virgatum* | Pavir.2KG572700 |
| *Capsella grandiflora* | Cagra.1613s0011 |  | *Panicum virgatum* | Pavir.2NG149700 |
| *Capsella grandiflora* | Cagra.1671s0068 |  | *Panicum virgatum* | Pavir.2NG215300 |
| *Capsella grandiflora* | Cagra.1671s0152 |  | *Panicum virgatum* | Pavir.2NG448600 |
| *Capsella grandiflora* | Cagra.1671s0327 |  | *Panicum virgatum* | Pavir.2NG624700 |
| *Capsella grandiflora* | Cagra.1757s0004 |  | *Panicum virgatum* | Pavir.3NG256500 |
| *Capsella grandiflora* | Cagra.1873s0012 |  | *Panicum virgatum* | Pavir.4KG134100 |
| *Capsella grandiflora* | Cagra.2129s0015 |  | *Panicum virgatum* | Pavir.4KG163000 |
| *Capsella grandiflora* | Cagra.2171s0014 |  | *Panicum virgatum* | Pavir.4KG184700 |
| *Capsella grandiflora* | Cagra.2240s0024 |  | *Panicum virgatum* | Pavir.4KG363800 |
| *Capsella grandiflora* | Cagra.2346s0015 |  | *Panicum virgatum* | Pavir.4KG377700 |
| *Capsella grandiflora* | Cagra.2622s0025 |  | *Panicum virgatum* | Pavir.4NG119700 |
| *Capsella grandiflora* | Cagra.2848s0001 |  | *Panicum virgatum* | Pavir.4NG153800 |
| *Capsella grandiflora* | Cagra.2848s0048 |  | *Panicum virgatum* | Pavir.4NG159900 |
| *Capsella grandiflora* | Cagra.3166s0042 |  | *Panicum virgatum* | Pavir.4NG268500 |
| *Capsella grandiflora* | Cagra.3364s0007 |  | *Panicum virgatum* | Pavir.4NG310300 |
| *Capsella grandiflora* | Cagra.3392s0012 |  | *Panicum virgatum* | Pavir.5KG544800 |
| *Capsella grandiflora* | Cagra.3467s0039 |  | *Panicum virgatum* | Pavir.5KG616000 |
| *Capsella grandiflora* | Cagra.5641s0053 |  | *Panicum virgatum* | Pavir.5NG223300 |
| *Capsella grandiflora* | Cagra.6845s0028 |  | *Panicum virgatum* | Pavir.5NG401600 |
| *Capsella grandiflora* | Cagra.6845s0029 |  | *Panicum virgatum* | Pavir.5NG578200 |
| *Capsella grandiflora* | Cagra.7487s0003 |  | *Panicum virgatum* | Pavir.6KG022000 |
| *Capsella grandiflora* | Cagra.7993s0010 |  | *Panicum virgatum* | Pavir.6KG134900 |
| *Capsella rubella* | Carubv10000314m |  | *Panicum virgatum* | Pavir.6KG177000 |
| *Capsella rubella* | Carubv10000561m |  | *Panicum virgatum* | Pavir.6NG020300 |
| *Capsella rubella* | Carubv10000600m |  | *Panicum virgatum* | Pavir.6NG330000 |
| *Capsella rubella* | Carubv10001192m |  | *Panicum virgatum* | Pavir.7KG225200 |
| *Capsella rubella* | Carubv10001230m |  | *Panicum virgatum* | Pavir.7NG086000 |
| *Capsella rubella* | Carubv10001279m |  | *Panicum virgatum* | Pavir.7NG253900 |
| *Capsella rubella* | Carubv10001369m |  | *Panicum virgatum* | Pavir.7NG305400 |
| *Capsella rubella* | Carubv10001371m |  | *Panicum virgatum* | Pavir.8KG017000 |
| *Capsella rubella* | Carubv10001375m |  | *Panicum virgatum* | Pavir.8KG072800 |
| *Capsella rubella* | Carubv10004999m |  | *Panicum virgatum* | Pavir.8NG007400 |
| *Capsella rubella* | Carubv10005404m |  | *Panicum virgatum* | Pavir.8NG050900 |
| *Capsella rubella* | Carubv10006202m |  | *Panicum virgatum* | Pavir.8NG097900 |
| *Capsella rubella* | Carubv10006355m |  | *Panicum virgatum* | Pavir.9KG048100 |
| *Capsella rubella* | Carubv10007619m |  | *Panicum virgatum* | Pavir.9KG194900 |
| *Capsella rubella* | Carubv10009230m |  | *Panicum virgatum* | Pavir.9KG330800 |
| *Capsella rubella* | Carubv10009410m |  | *Panicum virgatum* | Pavir.9KG444400 |
| *Capsella rubella* | Carubv10009731m |  | *Panicum virgatum* | Pavir.9KG482200 |
| *Capsella rubella* | Carubv10009866m |  | *Panicum virgatum* | Pavir.9KG582300 |
| *Capsella rubella* | Carubv10011200m |  | *Panicum virgatum* | Pavir.9NG114400 |
| *Capsella rubella* | Carubv10011948m |  | *Panicum virgatum* | Pavir.9NG134200 |
| *Capsella rubella* | Carubv10013971m |  | *Panicum virgatum* | Pavir.9NG157100 |
| *Capsella rubella* | Carubv10015501m |  | *Panicum virgatum* | Pavir.9NG308600 |
| *Capsella rubella* | Carubv10016131m |  | *Panicum virgatum* | Pavir.9NG456000 |
| *Capsella rubella* | Carubv10016264m |  | *Panicum virgatum* | Pavir.9NG587000 |
| *Capsella rubella* | Carubv10016442m |  | *Panicum virgatum* | Pavir.9NG594300 |
| *Capsella rubella* | Carubv10020384m |  | *Panicum virgatum* | Pavir.9NG633600 |
| *Capsella rubella* | Carubv10020393m |  | *Panicum virgatum* | Pavir.9NG690300 |
| *Capsella rubella* | Carubv10022040m |  | *Panicum virgatum* | Pavir.9NG763700 |
| *Capsella rubella* | Carubv10023307m |  | *Phaseolus vulgaris* | Phvul.001G074900 |
| *Capsella rubella* | Carubv10023323m |  | *Phaseolus vulgaris* | Phvul.001G087300 |
| *Capsella rubella* | Carubv10023453m |  | *Phaseolus vulgaris* | Phvul.001G137925 |
| *Capsella rubella* | Carubv10023601m |  | *Phaseolus vulgaris* | Phvul.001G203000 |
| *Capsella rubella* | Carubv10023746m |  | *Phaseolus vulgaris* | Phvul.002G158500 |
| *Capsella rubella* | Carubv10026312m |  | *Phaseolus vulgaris* | Phvul.002G213800 |
| *Capsella rubella* | Carubv10026357m |  | *Phaseolus vulgaris* | Phvul.002G291600 |
| *Capsella rubella* | Carubv10026429m |  | *Phaseolus vulgaris* | Phvul.002G316100 |
| *Capsella rubella* | Carubv10026653m |  | *Phaseolus vulgaris* | Phvul.003G036000 |
| *Capsella rubella* | Carubv10026930m |  | *Phaseolus vulgaris* | Phvul.003G141200 |
| *Capsella rubella* | Carubv10026959m |  | *Phaseolus vulgaris* | Phvul.003G149000 |
| *Capsella rubella* | Carubv10028191m |  | *Phaseolus vulgaris* | Phvul.003G188400 |
| *Capsella rubella* | Carubv10028382m |  | *Phaseolus vulgaris* | Phvul.004G046601 |
| *Carica papaya* | evm.model.supercontig_1.291 | | *Phaseolus vulgaris* | Phvul.004G157300 |
| *Carica papaya* | evm.model.supercontig_123.53 | | *Phaseolus vulgaris* | Phvul.005G049801 |
| *Carica papaya* | evm.model.supercontig_1332.1 | | *Phaseolus vulgaris* | Phvul.005G113300 |
| *Carica papaya* | evm.model.supercontig_136.56 | | *Phaseolus vulgaris* | Phvul.006G005200 |
| *Carica papaya* | evm.model.supercontig_139.32 | | *Phaseolus vulgaris* | Phvul.007G027200 |
| *Carica papaya* | evm.model.supercontig_155.28 | | *Phaseolus vulgaris* | Phvul.007G162500 |
| *Carica papaya* | evm.model.supercontig_180.14 | | *Phaseolus vulgaris* | Phvul.007G166100 |
| *Carica papaya* | evm.model.supercontig_19.26 | | *Phaseolus vulgaris* | Phvul.007G270600 |
| *Carica papaya* | evm.model.supercontig_193.20 | | *Phaseolus vulgaris* | Phvul.008G022800 |
| *Carica papaya* | evm.model.supercontig_2.373 | | *Phaseolus vulgaris* | Phvul.008G022925 |
| *Carica papaya* | evm.model.supercontig_20.16 | | *Phaseolus vulgaris* | Phvul.008G147400 |
| *Carica papaya* | evm.model.supercontig_3.152 | | *Phaseolus vulgaris* | Phvul.008G182900 |
| *Carica papaya* | evm.model.supercontig_3.516 | | *Phaseolus vulgaris* | Phvul.008G190200 |
| *Carica papaya* | evm.model.supercontig_3.74 | | *Phaseolus vulgaris* | Phvul.008G254600 |
| *Carica papaya* | evm.model.supercontig_3189.2 | | *Phaseolus vulgaris* | Phvul.008G257300 |
| *Carica papaya* | evm.model.supercontig_331.5 | | *Phaseolus vulgaris* | Phvul.009G035300 |
| *Carica papaya* | evm.model.supercontig_44.6 | | *Phaseolus vulgaris* | Phvul.009G035400 |
| *Carica papaya* | evm.model.supercontig_70.6 | | *Phaseolus vulgaris* | Phvul.009G045000 |
| *Carica papaya* | evm.model.supercontig_8.239 | | *Phaseolus vulgaris* | Phvul.009G053300 |
| *Carica papaya* | evm.model.supercontig_862.1 | | *Phaseolus vulgaris* | Phvul.009G085300 |
| *Carica papaya* | evm.model.supercontig_9.175 | | *Phaseolus vulgaris* | Phvul.009G118600 |
| *Carica papaya* | evm.model.supercontig_95.76 | | *Phaseolus vulgaris* | Phvul.009G185500 |
| *Carica papaya* | evm.model.supercontig_95.8 | | *Phaseolus vulgaris* | Phvul.009G258300 |
| *Carica papaya* | evm.TU.contig_35126.1 | | *Phaseolus vulgaris* | Phvul.010G119700 |
| *Carica papaya* | evm.TU.contig_46357.1 | | *Phaseolus vulgaris* | Phvul.011G069800 |
| *Chenopodium quinoa* | AUR62001356-RA |  | *Phaseolus vulgaris* | Phvul.011G070600 |
| *Chenopodium quinoa* | AUR62001856-RA |  | *Phaseolus vulgaris* | Phvul.L001944 |
| *Chenopodium quinoa* | AUR62002867-RA |  | *Phaseolus vulgaris* | Phvul.L002465 |
| *Chenopodium quinoa* | AUR62004561-RA |  | *Populus deltoides WV94* | Podel.01G067900 |
| *Chenopodium quinoa* | AUR62005692-RA |  | *Populus deltoides WV94* | Podel.01G106800 |
| *Chenopodium quinoa* | AUR62005995-RA |  | *Populus deltoides WV94* | Podel.01G107000 |
| *Chenopodium quinoa* | AUR62006104-RA |  | *Populus deltoides WV94* | Podel.01G147400 |
| *Chenopodium quinoa* | AUR62008459-RA |  | *Populus deltoides WV94* | Podel.01G244100 |
| *Chenopodium quinoa* | AUR62008578-RA |  | *Populus deltoides WV94* | Podel.01G245200 |
| *Chenopodium quinoa* | AUR62009440-RA |  | *Populus deltoides WV94* | Podel.01G299100 |
| *Chenopodium quinoa* | AUR62009650-RA |  | *Populus deltoides WV94* | Podel.01G342900 |
| *Chenopodium quinoa* | AUR62012114-RA |  | *Populus deltoides WV94* | Podel.01G361400 |
| *Chenopodium quinoa* | AUR62014361-RA |  | *Populus deltoides WV94* | Podel.02G121100 |
| *Chenopodium quinoa* | AUR62016173-RA |  | *Populus deltoides WV94* | Podel.02G121200 |
| *Chenopodium quinoa* | AUR62019102-RA |  | *Populus deltoides WV94* | Podel.02G198800 |
| *Chenopodium quinoa* | AUR62019388-RA |  | *Populus deltoides WV94* | Podel.02G230600 |
| *Chenopodium quinoa* | AUR62020307-RA |  | *Populus deltoides WV94* | Podel.02G239600 |
| *Chenopodium quinoa* | AUR62021636-RA |  | *Populus deltoides WV94* | Podel.03G139600 |
| *Chenopodium quinoa* | AUR62022677-RA |  | *Populus deltoides WV94* | Podel.03G177600 |
| *Chenopodium quinoa* | AUR62023118-RA |  | *Populus deltoides WV94* | Podel.04G117900 |
| *Chenopodium quinoa* | AUR62025058-RA |  | *Populus deltoides WV94* | Podel.05G109100 |
| *Chenopodium quinoa* | AUR62025648-RA |  | *Populus deltoides WV94* | Podel.05G164200 |
| *Chenopodium quinoa* | AUR62026898-RA |  | *Populus deltoides WV94* | Podel.05G164400 |
| *Chenopodium quinoa* | AUR62027097-RA |  | *Populus deltoides WV94* | Podel.05G175400 |
| *Chenopodium quinoa* | AUR62028076-RA |  | *Populus deltoides WV94* | Podel.06G081200 |
| *Chenopodium quinoa* | AUR62028867-RA |  | *Populus deltoides WV94* | Podel.06G181500 |
| *Chenopodium quinoa* | AUR62030486-RA |  | *Populus deltoides WV94* | Podel.06G283200 |
| *Chenopodium quinoa* | AUR62030805-RA |  | *Populus deltoides WV94* | Podel.07G074900 |
| *Chenopodium quinoa* | AUR62030939-RA |  | *Populus deltoides WV94* | Podel.07G126800 |
| *Chenopodium quinoa* | AUR62035217-RA |  | *Populus deltoides WV94* | Podel.07G126900 |
| *Chenopodium quinoa* | AUR62035221-RA |  | *Populus deltoides WV94* | Podel.07G131400 |
| *Chenopodium quinoa* | AUR62036007-RA |  | *Populus deltoides WV94* | Podel.08G057100 |
| *Chenopodium quinoa* | AUR62037140-RA |  | *Populus deltoides WV94* | Podel.08G141500 |
| *Chenopodium quinoa* | AUR62039984-RA |  | *Populus deltoides WV94* | Podel.08G195400 |
| *Chenopodium quinoa* | AUR62040293-RA |  | *Populus deltoides WV94* | Podel.10G060600 |
| *Chenopodium quinoa* | AUR62040721-RA |  | *Populus deltoides WV94* | Podel.10G124400 |
| *Chenopodium quinoa* | AUR62041089-RA |  | *Populus deltoides WV94* | Podel.10G220500 |
| *Chenopodium quinoa* | AUR62041615-RA |  | *Populus deltoides WV94* | Podel.10G259300 |
| *Citrus clementina* | Ciclev10001532m |  | *Populus deltoides WV94* | Podel.12G018100 |
| *Citrus clementina* | Ciclev10001533m |  | *Populus deltoides WV94* | Podel.14G109300 |
| *Citrus clementina* | Ciclev10001537m |  | *Populus deltoides WV94* | Podel.14G179600 |
| *Citrus clementina* | Ciclev10002006m |  | *Populus deltoides WV94* | Podel.15G002200 |
| *Citrus clementina* | Ciclev10002056m |  | *Populus deltoides WV94* | Podel.15G014300 |
| *Citrus clementina* | Ciclev10002060m |  | *Populus deltoides WV94* | Podel.15G057200 |
| *Citrus clementina* | Ciclev10002131m |  | *Populus deltoides WV94* | Podel.15G064000 |
| *Citrus clementina* | Ciclev10002330m |  | *Populus deltoides WV94* | Podel.17G040700 |
| *Citrus clementina* | Ciclev10002535m |  | *Populus deltoides WV94* | Podel.17G043000 |
| *Citrus clementina* | Ciclev10004980m |  | *Populus deltoides WV94* | Podel.17G111700 |
| *Citrus clementina* | Ciclev10005692m |  | *Populus deltoides WV94* | Podel.17G114700 |
| *Citrus clementina* | Ciclev10008308m |  | *Populus deltoides WV94* | Podel.18G013600 |
| *Citrus clementina* | Ciclev10008366m |  | *Populus deltoides WV94* | Podel.18G098700 |
| *Citrus clementina* | Ciclev10008402m |  | *Populus deltoides WV94* | Podel.18G147400 |
| *Citrus clementina* | Ciclev10008438m |  | *Populus deltoides WV94* | Podel.T030200 |
| *Citrus clementina* | Ciclev10008813m |  | *Populus deltoides WV94* | Podel.T041400 |
| *Citrus clementina* | Ciclev10011108m |  | *Populus trichocarpa* | Potri.001G061800 |
| *Citrus clementina* | Ciclev10011112m |  | *Populus trichocarpa* | Potri.001G101200 |
| *Citrus clementina* | Ciclev10011311m |  | *Populus trichocarpa* | Potri.001G136700 |
| *Citrus clementina* | Ciclev10012022m |  | *Populus trichocarpa* | Potri.001G233600 |
| *Citrus clementina* | Ciclev10014969m |  | *Populus trichocarpa* | Potri.001G281700 |
| *Citrus clementina* | Ciclev10015173m |  | *Populus trichocarpa* | Potri.001G323500 |
| *Citrus clementina* | Ciclev10015305m |  | *Populus trichocarpa* | Potri.001G339200 |
| *Citrus clementina* | Ciclev10015362m |  | *Populus trichocarpa* | Potri.002G110800 |
| *Citrus clementina* | Ciclev10015364m |  | *Populus trichocarpa* | Potri.002G110900 |
| *Citrus clementina* | Ciclev10015595m |  | *Populus trichocarpa* | Potri.002G179800 |
| *Citrus clementina* | Ciclev10015864m |  | *Populus trichocarpa* | Potri.002G208100 |
| *Citrus clementina* | Ciclev10019177m |  | *Populus trichocarpa* | Potri.002G214500 |
| *Citrus clementina* | Ciclev10019375m |  | *Populus trichocarpa* | Potri.002G220732 |
| *Citrus clementina* | Ciclev10019493m |  | *Populus trichocarpa* | Potri.003G130500 |
| *Citrus clementina* | Ciclev10019534m |  | *Populus trichocarpa* | Potri.003G166000 |
| *Citrus clementina* | Ciclev10019535m |  | *Populus trichocarpa* | Potri.003G166032 |
| *Citrus clementina* | Ciclev10019539m |  | *Populus trichocarpa* | Potri.004G108300 |
| *Citrus clementina* | Ciclev10019540m |  | *Populus trichocarpa* | Potri.005G097700 |
| *Citrus clementina* | Ciclev10019802m |  | *Populus trichocarpa* | Potri.005G152500 |
| *Citrus clementina* | Ciclev10019867m |  | *Populus trichocarpa* | Potri.005G152800 |
| *Citrus clementina* | Ciclev10019868m |  | *Populus trichocarpa* | Potri.006G075200 |
| *Citrus clementina* | Ciclev10020440m |  | *Populus trichocarpa* | Potri.006G173600 |
| *Citrus clementina* | Ciclev10020545m |  | *Populus trichocarpa* | Potri.006G267700 |
| *Citrus clementina* | Ciclev10020722m |  | *Populus trichocarpa* | Potri.007G066800 |
| *Citrus clementina* | Ciclev10021189m |  | *Populus trichocarpa* | Potri.007G116500 |
| *Citrus clementina* | Ciclev10022287m |  | *Populus trichocarpa* | Potri.007G116700 |
| *Citrus clementina* | Ciclev10022421m |  | *Populus trichocarpa* | Potri.007G121200 |
| *Citrus clementina* | Ciclev10023753m |  | *Populus trichocarpa* | Potri.008G046200 |
| *Citrus clementina* | Ciclev10027306m |  | *Populus trichocarpa* | Potri.008G120400 |
| *Citrus clementina* | Ciclev10027794m |  | *Populus trichocarpa* | Potri.008G125200 |
| *Citrus clementina* | Ciclev10027909m |  | *Populus trichocarpa* | Potri.008G171500 |
| *Citrus clementina* | Ciclev10028671m |  | *Populus trichocarpa* | Potri.009G077100 |
| *Citrus clementina* | Ciclev10028736m |  | *Populus trichocarpa* | Potri.010G066100 |
| *Citrus clementina* | Ciclev10028987m |  | *Populus trichocarpa* | Potri.010G125100 |
| *Citrus clementina* | Ciclev10029075m |  | *Populus trichocarpa* | Potri.010G215200 |
| *Citrus clementina* | Ciclev10029146m |  | *Populus trichocarpa* | Potri.010G251600 |
| *Citrus clementina* | Ciclev10029153m |  | *Populus trichocarpa* | Potri.012G005900 |
| *Citrus clementina* | Ciclev10029170m |  | *Populus trichocarpa* | Potri.012G014000 |
| *Citrus clementina* | Ciclev10030911m |  | *Populus trichocarpa* | Potri.014G106000 |
| *Citrus clementina* | Ciclev10033431m |  | *Populus trichocarpa* | Potri.014G134601 |
| *Citrus sinensis* | orange1.1g003994m |  | *Populus trichocarpa* | Potri.014G170600 |
| *Citrus sinensis* | orange1.1g003995m |  | *Populus trichocarpa* | Potri.015G002300 |
| *Citrus sinensis* | orange1.1g004001m |  | *Populus trichocarpa* | Potri.015G014000 |
| *Citrus sinensis* | orange1.1g004184m |  | *Populus trichocarpa* | Potri.015G054600 |
| *Citrus sinensis* | orange1.1g005631m |  | *Populus trichocarpa* | Potri.015G061900 |
| *Citrus sinensis* | orange1.1g005920m |  | *Populus trichocarpa* | Potri.017G039300 |
| *Citrus sinensis* | orange1.1g007196m |  | *Populus trichocarpa* | Potri.017G039301 |
| *Citrus sinensis* | orange1.1g007205m |  | *Populus trichocarpa* | Potri.017G042200 |
| *Citrus sinensis* | orange1.1g007462m |  | *Populus trichocarpa* | Potri.017G107500 |
| *Citrus sinensis* | orange1.1g008649m |  | *Populus trichocarpa* | Potri.018G013800 |
| *Citrus sinensis* | orange1.1g008655m |  | *Populus trichocarpa* | Potri.018G142100 |
| *Citrus sinensis* | orange1.1g008761m |  | *Populus trichocarpa* | Potri.T016900 |
| *Citrus sinensis* | orange1.1g010665m |  | *Populus trichocarpa* | Potri.T094400 |
| *Citrus sinensis* | orange1.1g011192m |  | *Populus trichocarpa* | Potri.T125800 |
| *Citrus sinensis* | orange1.1g011208m |  | *Prunus persica* | Prupe.1G047400 |
| *Citrus sinensis* | orange1.1g011217m |  | *Prunus persica* | Prupe.1G074900 |
| *Citrus sinensis* | orange1.1g011625m |  | *Prunus persica* | Prupe.1G093900 |
| *Citrus sinensis* | orange1.1g012558m |  | *Prunus persica* | Prupe.1G219800 |
| *Citrus sinensis* | orange1.1g013244m |  | *Prunus persica* | Prupe.1G310900 |
| *Citrus sinensis* | orange1.1g013329m |  | *Prunus persica* | Prupe.1G398700 |
| *Citrus sinensis* | orange1.1g013500m |  | *Prunus persica* | Prupe.1G534300 |
| *Citrus sinensis* | orange1.1g014072m |  | *Prunus persica* | Prupe.1G534400 |
| *Citrus sinensis* | orange1.1g014102m |  | *Prunus persica* | Prupe.2G198500 |
| *Citrus sinensis* | orange1.1g014662m |  | *Prunus persica* | Prupe.2G310900 |
| *Citrus sinensis* | orange1.1g014667m |  | *Prunus persica* | Prupe.2G318900 |
| *Citrus sinensis* | orange1.1g014687m |  | *Prunus persica* | Prupe.3G019800 |
| *Citrus sinensis* | orange1.1g014844m |  | *Prunus persica* | Prupe.3G194500 |
| *Citrus sinensis* | orange1.1g015643m |  | *Prunus persica* | Prupe.3G220900 |
| *Citrus sinensis* | orange1.1g016553m |  | *Prunus persica* | Prupe.3G245100 |
| *Citrus sinensis* | orange1.1g016554m |  | *Prunus persica* | Prupe.3G296700 |
| *Citrus sinensis* | orange1.1g016775m |  | *Prunus persica* | Prupe.4G138100 |
| *Citrus sinensis* | orange1.1g017013m |  | *Prunus persica* | Prupe.5G104100 |
| *Citrus sinensis* | orange1.1g017166m |  | *Prunus persica* | Prupe.5G197900 |
| *Citrus sinensis* | orange1.1g017195m |  | *Prunus persica* | Prupe.5G198000 |
| *Citrus sinensis* | orange1.1g017321m |  | *Prunus persica* | Prupe.5G221000 |
| *Citrus sinensis* | orange1.1g017348m |  | *Prunus persica* | Prupe.6G050300 |
| *Citrus sinensis* | orange1.1g017356m |  | *Prunus persica* | Prupe.6G088100 |
| *Citrus sinensis* | orange1.1g017409m |  | *Prunus persica* | Prupe.6G244200 |
| *Citrus sinensis* | orange1.1g017738m |  | *Prunus persica* | Prupe.8G071200 |
| *Citrus sinensis* | orange1.1g017778m |  | *Prunus persica* | Prupe.8G071300 |
| *Citrus sinensis* | orange1.1g017819m |  | *Prunus persica* | Prupe.8G253900 |
| *Citrus sinensis* | orange1.1g017834m |  | *Prunus persica* | Pum0229s0031 |
| *Citrus sinensis* | orange1.1g018277m |  | *Prunus persica* | Pum0268s0027 |
| *Citrus sinensis* | orange1.1g018624m |  | *Prunus persica* | Pum1029s0001 |
| *Citrus sinensis* | orange1.1g018626m |  | *Ricinus communis* | 27506.m000051 |
| *Citrus sinensis* | orange1.1g018641m |  | *Ricinus communis* | 27524.m000294 |
| *Citrus sinensis* | orange1.1g018653m |  | *Ricinus communis* | 28207.m000104 |
| *Citrus sinensis* | orange1.1g018981m |  | *Ricinus communis* | 29496.m000135 |
| *Citrus sinensis* | orange1.1g019028m |  | *Ricinus communis* | 29596.m000719 |
| *Citrus sinensis* | orange1.1g019855m |  | *Ricinus communis* | 29596.m000720 |
| *Citrus sinensis* | orange1.1g021461m |  | *Ricinus communis* | 29633.m000912 |
| *Citrus sinensis* | orange1.1g022728m |  | *Ricinus communis* | 29647.m002019 |
| *Citrus sinensis* | orange1.1g022924m |  | *Ricinus communis* | 29758.m000642 |
| *Citrus sinensis* | orange1.1g023394m |  | *Ricinus communis* | 29805.m001503 |
| *Citrus sinensis* | orange1.1g023560m |  | *Ricinus communis* | 29838.m001723 |
| *Citrus sinensis* | orange1.1g023942m |  | *Ricinus communis* | 29838.m001724 |
| *Citrus sinensis* | orange1.1g024594m |  | *Ricinus communis* | 29848.m004493 |
| *Citrus sinensis* | orange1.1g024832m |  | *Ricinus communis* | 29866.m000621 |
| *Citrus sinensis* | orange1.1g025137m |  | *Ricinus communis* | 30005.m001282 |
| *Citrus sinensis* | orange1.1g025320m |  | *Ricinus communis* | 30008.m000813 |
| *Citrus sinensis* | orange1.1g025411m |  | *Ricinus communis* | 30039.m000233 |
| *Citrus sinensis* | orange1.1g026022m |  | *Ricinus communis* | 30076.m004450 |
| *Citrus sinensis* | orange1.1g026465m |  | *Ricinus communis* | 30128.m009041 |
| *Citrus sinensis* | orange1.1g026490m |  | *Ricinus communis* | 30130.m000280 |
| *Citrus sinensis* | orange1.1g027672m |  | *Ricinus communis* | 30131.m007007 |
| *Citrus sinensis* | orange1.1g027756m |  | *Ricinus communis* | 30138.m004044 |
| *Citrus sinensis* | orange1.1g030237m |  | *Ricinus communis* | 30156.m001737 |
| *Citrus sinensis* | orange1.1g033800m |  | *Ricinus communis* | 30169.m006339 |
| *Citrus sinensis* | orange1.1g036310m |  | *Ricinus communis* | 30169.m006492 |
| *Citrus sinensis* | orange1.1g038567m |  | *Ricinus communis* | 30190.m011250 |
| *Citrus sinensis* | orange1.1g039762m |  | *Ricinus communis* | 30198.m000843 |
| *Citrus sinensis* | orange1.1g042936m |  | *Ricinus communis* | 30199.m000884 |
| *Citrus sinensis* | orange1.1g042954m |  | *Salix purpurea* | SapurV1A.0001s2780 |
| *Cucumis sativus* | Cucsa.011020.1 |  | *Salix purpurea* | SapurV1A.0003s0450 |
| *Cucumis sativus* | Cucsa.032080.1 |  | *Salix purpurea* | SapurV1A.0004s0630 |
| *Cucumis sativus* | Cucsa.065710.1 |  | *Salix purpurea* | SapurV1A.0004s0640 |
| *Cucumis sativus* | Cucsa.072900.1 |  | *Salix purpurea* | SapurV1A.0008s0760 |
| *Cucumis sativus* | Cucsa.089010.1 |  | *Salix purpurea* | SapurV1A.0036s1170 |
| *Cucumis sativus* | Cucsa.089490.1 |  | *Salix purpurea* | SapurV1A.0038s0310 |
| *Cucumis sativus* | Cucsa.092180.1 |  | *Salix purpurea* | SapurV1A.0053s0110 |
| *Cucumis sativus* | Cucsa.092190.1 |  | *Salix purpurea* | SapurV1A.0054s0630 |
| *Cucumis sativus* | Cucsa.095220.1 |  | *Salix purpurea* | SapurV1A.0078s0420 |
| *Cucumis sativus* | Cucsa.100130.1 |  | *Salix purpurea* | SapurV1A.0079s0160 |
| *Cucumis sativus* | Cucsa.112930.1 |  | *Salix purpurea* | SapurV1A.0085s0460 |
| *Cucumis sativus* | Cucsa.118380.1 |  | *Salix purpurea* | SapurV1A.0098s0210 |
| *Cucumis sativus* | Cucsa.120160.1 |  | *Salix purpurea* | SapurV1A.0119s0010 |
| *Cucumis sativus* | Cucsa.151890.1 |  | *Salix purpurea* | SapurV1A.0123s0380 |
| *Cucumis sativus* | Cucsa.160520.1 |  | *Salix purpurea* | SapurV1A.0140s0190 |
| *Cucumis sativus* | Cucsa.160530.1 |  | *Salix purpurea* | SapurV1A.0163s0470 |
| *Cucumis sativus* | Cucsa.163000.1 |  | *Salix purpurea* | SapurV1A.0174s0170 |
| *Cucumis sativus* | Cucsa.174610.1 |  | *Salix purpurea* | SapurV1A.0184s0410 |
| *Cucumis sativus* | Cucsa.199050.1 |  | *Salix purpurea* | SapurV1A.0187s0150 |
| *Cucumis sativus* | Cucsa.202100.1 |  | *Salix purpurea* | SapurV1A.0187s0160 |
| *Cucumis sativus* | Cucsa.202520.1 |  | *Salix purpurea* | SapurV1A.0191s0260 |
| *Cucumis sativus* | Cucsa.213590.1 |  | *Salix purpurea* | SapurV1A.0195s0330 |
| *Cucumis sativus* | Cucsa.218190.1 |  | *Salix purpurea* | SapurV1A.0257s0010 |
| *Cucumis sativus* | Cucsa.239670.1 |  | *Salix purpurea* | SapurV1A.0284s0090 |
| *Cucumis sativus* | Cucsa.283570.1 |  | *Salix purpurea* | SapurV1A.0312s0080 |
| *Cucumis sativus* | Cucsa.303340.1 |  | *Salix purpurea* | SapurV1A.0314s0280 |
| *Cucumis sativus* | Cucsa.359570.1 |  | *Salix purpurea* | SapurV1A.0320s0100 |
| *Cucumis sativus* | Cucsa.365300.1 |  | *Salix purpurea* | SapurV1A.0329s0160 |
| *Cucumis sativus* | Cucsa.379690.1 |  | *Salix purpurea* | SapurV1A.0484s0180 |
| *Cucumis sativus* | Cucsa.383330.1 |  | *Salix purpurea* | SapurV1A.0524s0050 |
| *Daucus carota* | DCAR_000626 |  | *Salix purpurea* | SapurV1A.0566s0070 |
| *Daucus carota* | DCAR_001708 |  | *Salix purpurea* | SapurV1A.0674s0030 |
| *Daucus carota* | DCAR_001835 |  | *Salix purpurea* | SapurV1A.0727s0100 |
| *Daucus carota* | DCAR_002299 |  | *Salix purpurea* | SapurV1A.0790s0180 |
| *Daucus carota* | DCAR_003040 |  | *Salix purpurea* | SapurV1A.0875s0050 |
| *Daucus carota* | DCAR_005294 |  | *Salix purpurea* | SapurV1A.1204s0050 |
| *Daucus carota* | DCAR_006019 |  | *Salix purpurea* | SapurV1A.1216s0090 |
| *Daucus carota* | DCAR_007535 |  | *Salix purpurea* | SapurV1A.1433s0050 |
| *Daucus carota* | DCAR_007537 |  | *Salix purpurea* | SapurV1A.1539s0030 |
| *Daucus carota* | DCAR_008365 |  | *Salix purpurea* | SapurV1A.1617s0030 |
| *Daucus carota* | DCAR_008751 |  | *Salix purpurea* | SapurV1A.1785s0020 |
| *Daucus carota* | DCAR_008917 |  | *Salix purpurea* | SapurV1A.3312s0010 |
| *Daucus carota* | DCAR_009553 |  | *Salix purpurea* | SapurV1A.3359s0020 |
| *Daucus carota* | DCAR_009909 |  | *Salix purpurea* | SapurV1A.3835s0020 |
| *Daucus carota* | DCAR_010574 |  | *Salix purpurea* | SapurV1A.6289s0010 |
| *Daucus carota* | DCAR_011276 |  | *Setaria italica* | Seita.1G006700 |
| *Daucus carota* | DCAR_011541 |  | *Setaria italica* | Seita.1G065300 |
| *Daucus carota* | DCAR_012422 |  | *Setaria italica* | Seita.1G228800 |
| *Daucus carota* | DCAR_012741 |  | *Setaria italica* | Seita.1G236100 |
| *Daucus carota* | DCAR_012851 |  | *Setaria italica* | Seita.1G301300 |
| *Daucus carota* | DCAR_013732 |  | *Setaria italica* | Seita.1G304900 |
| *Daucus carota* | DCAR_014543 |  | *Setaria italica* | Seita.1G308800 |
| *Daucus carota* | DCAR_014564 |  | *Setaria italica* | Seita.2G175000 |
| *Daucus carota* | DCAR_015140 |  | *Setaria italica* | Seita.2G286100 |
| *Daucus carota* | DCAR_015255 |  | *Setaria italica* | Seita.2G423300 |
| *Daucus carota* | DCAR_016576 |  | *Setaria italica* | Seita.2G444300 |
| *Daucus carota* | DCAR_018030 |  | *Setaria italica* | Seita.3G119200 |
| *Daucus carota* | DCAR_018357 |  | *Setaria italica* | Seita.3G212400 |
| *Daucus carota* | DCAR_019663 |  | *Setaria italica* | Seita.3G285700 |
| *Daucus carota* | DCAR_019774 |  | *Setaria italica* | Seita.3G285800 |
| *Daucus carota* | DCAR_020110 |  | *Setaria italica* | Seita.4G001600 |
| *Daucus carota* | DCAR_020374 |  | *Setaria italica* | Seita.4G116600 |
| *Daucus carota* | DCAR_021082 |  | *Setaria italica* | Seita.4G122700 |
| *Daucus carota* | DCAR_021189 |  | *Setaria italica* | Seita.4G192300 |
| *Daucus carota* | DCAR_021905 |  | *Setaria italica* | Seita.4G243700 |
| *Daucus carota* | DCAR_023659 |  | *Setaria italica* | Seita.5G372500 |
| *Daucus carota* | DCAR_024191 |  | *Setaria italica* | Seita.6G082100 |
| *Daucus carota* | DCAR_024639 |  | *Setaria italica* | Seita.6G096800 |
| *Daucus carota* | DCAR_026289 |  | *Setaria italica* | Seita.6G226200 |
| *Daucus carota* | DCAR_030155 |  | *Setaria italica* | Seita.7G007800 |
| *Daucus carota* | DCAR_030760 |  | *Setaria italica* | Seita.7G153500 |
| *Daucus carota* | DCAR_032204 |  | *Setaria italica* | Seita.7G334500 |
| *Eutrema salsugineum* | Thhalv10000258m |  | *Setaria italica* | Seita.8G001200 |
| *Eutrema salsugineum* | Thhalv10001426m |  | *Setaria italica* | Seita.8G040100 |
| *Eutrema salsugineum* | Thhalv10001511m |  | *Setaria italica* | Seita.8G159000 |
| *Eutrema salsugineum* | Thhalv10001548m |  | *Setaria italica* | Seita.9G020100 |
| *Eutrema salsugineum* | Thhalv10003894m |  | *Setaria italica* | Seita.9G103000 |
| *Eutrema salsugineum* | Thhalv10003924m |  | *Setaria italica* | Seita.9G119800 |
| *Eutrema salsugineum* | Thhalv10004422m |  | *Setaria italica* | Seita.9G138400 |
| *Eutrema salsugineum* | Thhalv10004423m |  | *Setaria italica* | Seita.9G323600 |
| *Eutrema salsugineum* | Thhalv10007673m |  | *Setaria italica* | Seita.9G404500 |
| *Eutrema salsugineum* | Thhalv10007685m |  | *Setaria italica* | Seita.9G445200 |
| *Eutrema salsugineum* | Thhalv10007843m |  | *Setaria italica* | Seita.9G545700 |
| *Eutrema salsugineum* | Thhalv10008808m |  | *Setaria italica* | Seita.J028500 |
| *Eutrema salsugineum* | Thhalv10009385m |  | *Setaria viridis* | Sevir.1G006900 |
| *Eutrema salsugineum* | Thhalv10011633m |  | *Setaria viridis* | Sevir.1G064400 |
| *Eutrema salsugineum* | Thhalv10011649m |  | *Setaria viridis* | Sevir.1G087500 |
| *Eutrema salsugineum* | Thhalv10011695m |  | *Setaria viridis* | Sevir.1G232600 |
| *Eutrema salsugineum* | Thhalv10012789m |  | *Setaria viridis* | Sevir.1G241000 |
| *Eutrema salsugineum* | Thhalv10012793m |  | *Setaria viridis* | Sevir.1G307300 |
| *Eutrema salsugineum* | Thhalv10013099m |  | *Setaria viridis* | Sevir.1G314800 |
| *Eutrema salsugineum* | Thhalv10013118m |  | *Setaria viridis* | Sevir.2G181300 |
| *Eutrema salsugineum* | Thhalv10013182m |  | *Setaria viridis* | Sevir.2G296500 |
| *Eutrema salsugineum* | Thhalv10013517m |  | *Setaria viridis* | Sevir.2G435400 |
| *Eutrema salsugineum* | Thhalv10013561m |  | *Setaria viridis* | Sevir.2G456400 |
| *Eutrema salsugineum* | Thhalv10013895m |  | *Setaria viridis* | Sevir.3G121200 |
| *Eutrema salsugineum* | Thhalv10013919m |  | *Setaria viridis* | Sevir.3G216900 |
| *Eutrema salsugineum* | Thhalv10014044m |  | *Setaria viridis* | Sevir.3G293600 |
| *Eutrema salsugineum* | Thhalv10014378m |  | *Setaria viridis* | Sevir.4G002000 |
| *Eutrema salsugineum* | Thhalv10014534m |  | *Setaria viridis* | Sevir.4G117300 |
| *Eutrema salsugineum* | Thhalv10014750m |  | *Setaria viridis* | Sevir.4G194900 |
| *Eutrema salsugineum* | Thhalv10015486m |  | *Setaria viridis* | Sevir.4G202100 |
| *Eutrema salsugineum* | Thhalv10016744m |  | *Setaria viridis* | Sevir.4G256600 |
| *Eutrema salsugineum* | Thhalv10018663m |  | *Setaria viridis* | Sevir.5G378000 |
| *Eutrema salsugineum* | Thhalv10020880m |  | *Setaria viridis* | Sevir.6G082000 |
| *Eutrema salsugineum* | Thhalv10020965m |  | *Setaria viridis* | Sevir.6G104600 |
| *Eutrema salsugineum* | Thhalv10020967m |  | *Setaria viridis* | Sevir.6G233400 |
| *Eutrema salsugineum* | Thhalv10020970m |  | *Setaria viridis* | Sevir.7G031000 |
| *Eutrema salsugineum* | Thhalv10022310m |  | *Setaria viridis* | Sevir.7G161700 |
| *Eutrema salsugineum* | Thhalv10023638m |  | *Setaria viridis* | Sevir.7G340537 |
| *Eutrema salsugineum* | Thhalv10025322m |  | *Setaria viridis* | Sevir.8G020600 |
| *Eutrema salsugineum* | Thhalv10025713m |  | *Setaria viridis* | Sevir.8G038500 |
| *Eutrema salsugineum* | Thhalv10025793m |  | *Setaria viridis* | Sevir.8G169600 |
| *Eutrema salsugineum* | Thhalv10025795m |  | *Setaria viridis* | Sevir.9G019800 |
| *Eutrema salsugineum* | Thhalv10026081m |  | *Setaria viridis* | Sevir.9G101100 |
| *Eutrema salsugineum* | Thhalv10028337m |  | *Setaria viridis* | Sevir.9G118400 |
| *Fragaria vesca* | mrna03650.1-v1.0-hybrid | | *Setaria viridis* | Sevir.9G136800 |
| *Fragaria vesca* | mrna03651.1-v1.0-hybrid | | *Setaria viridis* | Sevir.9G228300 |
| *Fragaria vesca* | mrna03742.1-v1.0-hybrid | | *Setaria viridis* | Sevir.9G329600 |
| *Fragaria vesca* | mrna04172.1-v1.0-hybrid | | *Setaria viridis* | Sevir.9G407900 |
| *Fragaria vesca* | mrna10242.1-v1.0-hybrid | | *Setaria viridis* | Sevir.9G449300 |
| *Fragaria vesca* | mrna11568.1-v1.0-hybrid | | *Setaria viridis* | Sevir.9G550400 |
| *Fragaria vesca* | mrna12454.1-v1.0-hybrid | | *Solanum lycopersicum* | Solyc01g106030.2.1 |
| *Fragaria vesca* | mrna12862.1-v1.0-hybrid | | *Solanum lycopersicum* | Solyc01g106040.2.1 |
| *Fragaria vesca* | mrna14015.1-v1.0-hybrid | | *Solanum lycopersicum* | Solyc02g089520.1.1 |
| *Fragaria vesca* | mrna14981.1-v1.0-hybrid | | *Solanum lycopersicum* | Solyc02g089540.2.1 |
| *Fragaria vesca* | mrna15552.1-v1.0-hybrid | | *Solanum lycopersicum* | Solyc02g093590.2.1 |
| *Fragaria vesca* | mrna17250.1-v1.0-hybrid | | *Solanum lycopersicum* | Solyc03g081240.2.1 |
| *Fragaria vesca* | mrna18151.1-v1.0-hybrid | | *Solanum lycopersicum* | Solyc03g081270.1.1 |
| *Fragaria vesca* | mrna18611.1-v1.0-hybrid | | *Solanum lycopersicum* | Solyc03g083400.2.1 |
| *Fragaria vesca* | mrna21943.1-v1.0-hybrid | | *Solanum lycopersicum* | Solyc03g115770.2.1 |
| *Fragaria vesca* | mrna23905.1-v1.0-hybrid | | *Solanum lycopersicum* | Solyc03g119540.2.1 |
| *Fragaria vesca* | mrna25171.1-v1.0-hybrid | | *Solanum lycopersicum* | Solyc04g007210.2.1 |
| *Fragaria vesca* | mrna26055.1-v1.0-hybrid | | *Solanum lycopersicum* | Solyc04g049670.2.1 |
| *Fragaria vesca* | mrna26537.1-v1.0-hybrid | | *Solanum lycopersicum* | Solyc04g076530.2.1 |
| *Fragaria vesca* | mrna26538.1-v1.0-hybrid | | *Solanum lycopersicum* | Solyc05g009310.2.1 |
| *Fragaria vesca* | mrna27383.1-v1.0-hybrid | | *Solanum lycopersicum* | Solyc05g020020.2.1 |
| *Fragaria vesca* | mrna27406.1-v1.0-hybrid | | *Solanum lycopersicum* | Solyc05g024010.2.1 |
| *Fragaria vesca* | mrna27999.1-v1.0-hybrid | | *Solanum lycopersicum* | Solyc05g046040.1.1 |
| *Fragaria vesca* | mrna28201.1-v1.0-hybrid | | *Solanum lycopersicum* | Solyc06g069690.2.1 |
| *Fragaria vesca* | mrna30045.1-v1.0-hybrid | | *Solanum lycopersicum* | Solyc07g006630.2.1 |
| *Fragaria vesca* | mrna31805.1-v1.0-hybrid | | *Solanum lycopersicum* | Solyc07g008540.2.1 |
| *Glycine max* | Glyma.01G221100 |  | *Solanum lycopersicum* | Solyc07g045180.2.1 |
| *Glycine max* | Glyma.02G152900 |  | *Solanum lycopersicum* | Solyc07g066510.2.1 |
| *Glycine max* | Glyma.02G215600 |  | *Solanum lycopersicum* | Solyc08g006530.2.1 |
| *Glycine max* | Glyma.02G223700 |  | *Solanum lycopersicum* | Solyc08g081350.2.1 |
| *Glycine max* | Glyma.02G230800 |  | *Solanum lycopersicum* | Solyc09g074560.2.1 |
| *Glycine max* | Glyma.03G209800 |  | *Solanum lycopersicum* | Solyc09g090650.2.1 |
| *Glycine max* | Glyma.03G261300 |  | *Solanum lycopersicum* | Solyc10g005030.2.1 |
| *Glycine max* | Glyma.04G058900 |  | *Solanum lycopersicum* | Solyc10g047640.1.1 |
| *Glycine max* | Glyma.04G096200 |  | *Solanum lycopersicum* | Solyc11g072850.1.1 |
| *Glycine max* | Glyma.04G096300 |  | *Solanum lycopersicum* | Solyc12g096500.1.1 |
| *Glycine max* | Glyma.04G166300 |  | *Solanum tuberosum* | PGSC0003DMP400001175 |
| *Glycine max* | Glyma.04G228300 |  | *Solanum tuberosum* | PGSC0003DMP400002288 |
| *Glycine max* | Glyma.04G236400 |  | *Solanum tuberosum* | PGSC0003DMP400009430 |
| *Glycine max* | Glyma.05G025000 |  | *Solanum tuberosum* | PGSC0003DMP400009431 |
| *Glycine max* | Glyma.05G209800 |  | *Solanum tuberosum* | PGSC0003DMP400009918 |
| *Glycine max* | Glyma.05G233700 |  | *Solanum tuberosum* | PGSC0003DMP400011329 |
| *Glycine max* | Glyma.06G059600 |  | *Solanum tuberosum* | PGSC0003DMP400013670 |
| *Glycine max* | Glyma.06G097900 |  | *Solanum tuberosum* | PGSC0003DMP400016063 |
| *Glycine max* | Glyma.06G098000 |  | *Solanum tuberosum* | PGSC0003DMP400016064 |
| *Glycine max* | Glyma.06G127900 |  | *Solanum tuberosum* | PGSC0003DMP400016533 |
| *Glycine max* | Glyma.06G136600 |  | *Solanum tuberosum* | PGSC0003DMP400016534 |
| *Glycine max* | Glyma.06G196200 |  | *Solanum tuberosum* | PGSC0003DMP400016535 |
| *Glycine max* | Glyma.07G049400 |  | *Solanum tuberosum* | PGSC0003DMP400016536 |
| *Glycine max* | Glyma.07G091400 |  | *Solanum tuberosum* | PGSC0003DMP400017796 |
| *Glycine max* | Glyma.07G182700 |  | *Solanum tuberosum* | PGSC0003DMP400017799 |
| *Glycine max* | Glyma.08G016500 |  | *Solanum tuberosum* | PGSC0003DMP400017800 |
| *Glycine max* | Glyma.08G041100 |  | *Solanum tuberosum* | PGSC0003DMP400018893 |
| *Glycine max* | Glyma.08G067200 |  | *Solanum tuberosum* | PGSC0003DMP400018894 |
| *Glycine max* | Glyma.08G168900 |  | *Solanum tuberosum* | PGSC0003DMP400018895 |
| *Glycine max* | Glyma.08G221800 |  | *Solanum tuberosum* | PGSC0003DMP400018897 |
| *Glycine max* | Glyma.08G255200 |  | *Solanum tuberosum* | PGSC0003DMP400020015 |
| *Glycine max* | Glyma.09G184600 |  | *Solanum tuberosum* | PGSC0003DMP400020149 |
| *Glycine max* | Glyma.10G021400 |  | *Solanum tuberosum* | PGSC0003DMP400021550 |
| *Glycine max* | Glyma.10G048100 |  | *Solanum tuberosum* | PGSC0003DMP400021551 |
| *Glycine max* | Glyma.10G134400 |  | *Solanum tuberosum* | PGSC0003DMP400022350 |
| *Glycine max* | Glyma.10G190300 |  | *Solanum tuberosum* | PGSC0003DMP400022351 |
| *Glycine max* | Glyma.10G274300 |  | *Solanum tuberosum* | PGSC0003DMP400022352 |
| *Glycine max* | Glyma.11G022600 |  | *Solanum tuberosum* | PGSC0003DMP400025661 |
| *Glycine max* | Glyma.12G074400 |  | *Solanum tuberosum* | PGSC0003DMP400025662 |
| *Glycine max* | Glyma.12G196100 |  | *Solanum tuberosum* | PGSC0003DMP400027202 |
| *Glycine max* | Glyma.13G009300 |  | *Solanum tuberosum* | PGSC0003DMP400029950 |
| *Glycine max* | Glyma.13G050300 |  | *Solanum tuberosum* | PGSC0003DMP400029951 |
| *Glycine max* | Glyma.13G093800 |  | *Solanum tuberosum* | PGSC0003DMP400030430 |
| *Glycine max* | Glyma.13G100700 |  | *Solanum tuberosum* | PGSC0003DMP400030431 |
| *Glycine max* | Glyma.13G135900 |  | *Solanum tuberosum* | PGSC0003DMP400030432 |
| *Glycine max* | Glyma.13G306400 |  | *Solanum tuberosum* | PGSC0003DMP400033920 |
| *Glycine max* | Glyma.13G331000 |  | *Solanum tuberosum* | PGSC0003DMP400033921 |
| *Glycine max* | Glyma.14G182800 |  | *Solanum tuberosum* | PGSC0003DMP400038243 |
| *Glycine max* | Glyma.14G190400 |  | *Solanum tuberosum* | PGSC0003DMP400039846 |
| *Glycine max* | Glyma.14G197900 |  | *Solanum tuberosum* | PGSC0003DMP400039847 |
| *Glycine max* | Glyma.15G219800 |  | *Solanum tuberosum* | PGSC0003DMP400044086 |
| *Glycine max* | Glyma.15G258200 |  | *Solanum tuberosum* | PGSC0003DMP400044087 |
| *Glycine max* | Glyma.16G018000 |  | *Solanum tuberosum* | PGSC0003DMP400045674 |
| *Glycine max* | Glyma.16G050900 |  | *Solanum tuberosum* | PGSC0003DMP400045675 |
| *Glycine max* | Glyma.16G067000 |  | *Solanum tuberosum* | PGSC0003DMP400046377 |
| *Glycine max* | Glyma.17G059100 |  | *Solanum tuberosum* | PGSC0003DMP400047781 |
| *Glycine max* | Glyma.17G066600 |  | *Solanum tuberosum* | PGSC0003DMP400050196 |
| *Glycine max* | Glyma.17G102200 |  | *Solanum tuberosum* | PGSC0003DMP400050197 |
| *Glycine max* | Glyma.18G278100 |  | *Solanum tuberosum* | PGSC0003DMP400050198 |
| *Glycine max* | Glyma.19G039000 |  | *Solanum tuberosum* | PGSC0003DMP400050199 |
| *Glycine max* | Glyma.19G079600 |  | *Solanum tuberosum* | PGSC0003DMP400050932 |
| *Glycine max* | Glyma.19G099700 |  | *Solanum tuberosum* | PGSC0003DMP400051145 |
| *Glycine max* | Glyma.19G207100 |  | *Solanum tuberosum* | PGSC0003DMP400055803 |
| *Glycine max* | Glyma.19G260400 |  | *Solanum tuberosum* | PGSC0003DMP400055804 |
| *Glycine max* | Glyma.20G060400 |  | *Solanum tuberosum* | PGSC0003DMP400055805 |
| *Glycine max* | Glyma.20G115600 |  | *Solanum tuberosum* | PGSC0003DMP400055806 |
| *Glycine max* | Glyma.20G200400 |  | *Solanum tuberosum* | PGSC0003DMP400056067 |
| *Glycine max* | Glyma.U033700 |  | *Solanum tuberosum* | PGSC0003DMP400056068 |
| *Gossypium hirsutum* | Gohir.A01G021400 |  | *Solanum tuberosum* | PGSC0003DMP400064444 |
| *Gossypium hirsutum* | Gohir.A01G095600 |  | *Sorghum bicolor* | Sobic.001G100100 |
| *Gossypium hirsutum* | Gohir.A01G126000 |  | *Sorghum bicolor* | Sobic.001G118100 |
| *Gossypium hirsutum* | Gohir.A01G185300 |  | *Sorghum bicolor* | Sobic.001G135400 |
| *Gossypium hirsutum* | Gohir.A02G042600 |  | *Sorghum bicolor* | Sobic.001G225800 |
| *Gossypium hirsutum* | Gohir.A03G036900 |  | *Sorghum bicolor* | Sobic.001G298400 |
| *Gossypium hirsutum* | Gohir.A03G067700 |  | *Sorghum bicolor* | Sobic.001G372700 |
| *Gossypium hirsutum* | Gohir.A03G073100 |  | *Sorghum bicolor* | Sobic.001G411400 |
| *Gossypium hirsutum* | Gohir.A03G151100 |  | *Sorghum bicolor* | Sobic.001G510500 |
| *Gossypium hirsutum* | Gohir.A03G163400 |  | *Sorghum bicolor* | Sobic.002G275100 |
| *Gossypium hirsutum* | Gohir.A03G177600 |  | *Sorghum bicolor* | Sobic.002G408500 |
| *Gossypium hirsutum* | Gohir.A04G051500 |  | *Sorghum bicolor* | Sobic.003G347680 |
| *Gossypium hirsutum* | Gohir.A05G002200 |  | *Sorghum bicolor* | Sobic.004G007400 |
| *Gossypium hirsutum* | Gohir.A05G062400 |  | *Sorghum bicolor* | Sobic.004G040100 |
| *Gossypium hirsutum* | Gohir.A05G099300 |  | *Sorghum bicolor* | Sobic.004G063200 |
| *Gossypium hirsutum* | Gohir.A05G108600 |  | *Sorghum bicolor* | Sobic.004G211200 |
| *Gossypium hirsutum* | Gohir.A05G264500 |  | *Sorghum bicolor* | Sobic.004G216700 |
| *Gossypium hirsutum* | Gohir.A05G334900 |  | *Sorghum bicolor* | Sobic.004G249500 |
| *Gossypium hirsutum* | Gohir.A05G343600 |  | *Sorghum bicolor* | Sobic.004G252300 |
| *Gossypium hirsutum* | Gohir.A05G414200 |  | *Sorghum bicolor* | Sobic.004G256200 |
| *Gossypium hirsutum* | Gohir.A05G416000 |  | *Sorghum bicolor* | Sobic.005G000400 |
| *Gossypium hirsutum* | Gohir.A06G031200 |  | *Sorghum bicolor* | Sobic.005G044400 |
| *Gossypium hirsutum* | Gohir.A06G031300 |  | *Sorghum bicolor* | Sobic.006G004400 |
| *Gossypium hirsutum* | Gohir.A07G034300 |  | *Sorghum bicolor* | Sobic.006G135100 |
| *Gossypium hirsutum* | Gohir.A07G088200 |  | *Sorghum bicolor* | Sobic.007G023100 |
| *Gossypium hirsutum* | Gohir.A07G094600 |  | *Sorghum bicolor* | Sobic.007G092000 |
| *Gossypium hirsutum* | Gohir.A07G163000 |  | *Sorghum bicolor* | Sobic.007G189800 |
| *Gossypium hirsutum* | Gohir.A07G193400 |  | *Sorghum bicolor* | Sobic.008G000700 |
| *Gossypium hirsutum* | Gohir.A08G022000 |  | *Sorghum bicolor* | Sobic.009G167300 |
| *Gossypium hirsutum* | Gohir.A08G049100 |  | *Sorghum bicolor* | Sobic.009G259100 |
| *Gossypium hirsutum* | Gohir.A08G086800 |  | *Sorghum bicolor* | Sobic.010G001200 |
| *Gossypium hirsutum* | Gohir.A08G093600 |  | *Sorghum bicolor* | Sobic.010G108500 |
| *Gossypium hirsutum* | Gohir.A08G094500 |  | *Sorghum bicolor* | Sobic.010G115800 |
| *Gossypium hirsutum* | Gohir.A08G116500 |  | *Sorghum bicolor* | Sobic.010G123500 |
| *Gossypium hirsutum* | Gohir.A08G184100 |  | *Sorghum bicolor* | Sobic.010G214000 |
| *Gossypium hirsutum* | Gohir.A09G031400 |  | *Sorghum bicolor* | Sobic.010G249200 |
| *Gossypium hirsutum* | Gohir.A09G047500 |  | *Sorghum bicolor Rio* | SbRio.01G104300 |
| *Gossypium hirsutum* | Gohir.A09G059500 |  | *Sorghum bicolor Rio* | SbRio.01G123200 |
| *Gossypium hirsutum* | Gohir.A09G160600 |  | *Sorghum bicolor Rio* | SbRio.01G141200 |
| *Gossypium hirsutum* | Gohir.A09G192000 |  | *Sorghum bicolor Rio* | SbRio.01G240200 |
| *Gossypium hirsutum* | Gohir.A10G109100 |  | *Sorghum bicolor Rio* | SbRio.01G322700 |
| *Gossypium hirsutum* | Gohir.A11G014700 |  | *Sorghum bicolor Rio* | SbRio.01G396600 |
| *Gossypium hirsutum* | Gohir.A11G016500 |  | *Sorghum bicolor Rio* | SbRio.01G437700 |
| *Gossypium hirsutum* | Gohir.A11G101100 |  | *Sorghum bicolor Rio* | SbRio.01G545300 |
| *Gossypium hirsutum* | Gohir.A11G147600 |  | *Sorghum bicolor Rio* | SbRio.02G289800 |
| *Gossypium hirsutum* | Gohir.A11G325400 |  | *Sorghum bicolor Rio* | SbRio.02G429100 |
| *Gossypium hirsutum* | Gohir.A12G008800 |  | *Sorghum bicolor Rio* | SbRio.03G374600 |
| *Gossypium hirsutum* | Gohir.A12G016900 |  | *Sorghum bicolor Rio* | SbRio.04G008100 |
| *Gossypium hirsutum* | Gohir.A12G057200 |  | *Sorghum bicolor Rio* | SbRio.04G044800 |
| *Gossypium hirsutum* | Gohir.A12G231000 |  | *Sorghum bicolor Rio* | SbRio.04G068500 |
| *Gossypium hirsutum* | Gohir.A12G246400 |  | *Sorghum bicolor Rio* | SbRio.04G224900 |
| *Gossypium hirsutum* | Gohir.A12G250400 |  | *Sorghum bicolor Rio* | SbRio.04G230500 |
| *Gossypium hirsutum* | Gohir.A13G050400 |  | *Sorghum bicolor Rio* | SbRio.04G265700 |
| *Gossypium hirsutum* | Gohir.A13G059300 |  | *Sorghum bicolor Rio* | SbRio.04G268900 |
| *Gossypium hirsutum* | Gohir.A13G178800 |  | *Sorghum bicolor Rio* | SbRio.04G273100 |
| *Gossypium hirsutum* | Gohir.A13G211600 |  | *Sorghum bicolor Rio* | SbRio.05G001100 |
| *Gossypium hirsutum* | Gohir.D01G019800 |  | *Sorghum bicolor Rio* | SbRio.05G046700 |
| *Gossypium hirsutum* | Gohir.D01G079800 |  | *Sorghum bicolor Rio* | SbRio.06G064500 |
| *Gossypium hirsutum* | Gohir.D01G113500 |  | *Sorghum bicolor Rio* | SbRio.06G149400 |
| *Gossypium hirsutum* | Gohir.D01G176000 |  | *Sorghum bicolor Rio* | SbRio.07G024200 |
| *Gossypium hirsutum* | Gohir.D02G048200 |  | *Sorghum bicolor Rio* | SbRio.07G098300 |
| *Gossypium hirsutum* | Gohir.D02G174100 |  | *Sorghum bicolor Rio* | SbRio.07G202000 |
| *Gossypium hirsutum* | Gohir.D02G186800 |  | *Sorghum bicolor Rio* | SbRio.08G001100 |
| *Gossypium hirsutum* | Gohir.D02G201300 |  | *Sorghum bicolor Rio* | SbRio.09G176500 |
| *Gossypium hirsutum* | Gohir.D03G094200 |  | *Sorghum bicolor Rio* | SbRio.09G274500 |
| *Gossypium hirsutum* | Gohir.D03G099600 |  | *Sorghum bicolor Rio* | SbRio.10G001300 |
| *Gossypium hirsutum* | Gohir.D03G131400 |  | *Sorghum bicolor Rio* | SbRio.10G132400 |
| *Gossypium hirsutum* | Gohir.D04G000100 |  | *Sorghum bicolor Rio* | SbRio.10G141500 |
| *Gossypium hirsutum* | Gohir.D04G001900 |  | *Sorghum bicolor Rio* | SbRio.10G152400 |
| *Gossypium hirsutum* | Gohir.D04G082000 |  | *Sorghum bicolor Rio* | SbRio.10G278300 |
| *Gossypium hirsutum* | Gohir.D05G002600 |  | *Sorghum bicolor Rio* | SbRio.10G322300 |
| *Gossypium hirsutum* | Gohir.D05G002800 |  | *Spirodela polyrhiza* | Spipo0G0060600 |
| *Gossypium hirsutum* | Gohir.D05G064900 |  | *Spirodela polyrhiza* | Spipo0G0060800 |
| *Gossypium hirsutum* | Gohir.D05G099200 |  | *Spirodela polyrhiza* | Spipo0G0122800 |
| *Gossypium hirsutum* | Gohir.D05G099700 |  | *Spirodela polyrhiza* | Spipo0G0183600 |
| *Gossypium hirsutum* | Gohir.D05G108700 |  | *Spirodela polyrhiza* | Spipo10G0007900 |
| *Gossypium hirsutum* | Gohir.D05G267000 |  | *Spirodela polyrhiza* | Spipo10G0011300 |
| *Gossypium hirsutum* | Gohir.D05G316200 |  | *Spirodela polyrhiza* | Spipo11G0033500 |
| *Gossypium hirsutum* | Gohir.D05G329800 |  | *Spirodela polyrhiza* | Spipo11G0061800 |
| *Gossypium hirsutum* | Gohir.D06G030400 |  | *Spirodela polyrhiza* | Spipo12G0012900 |
| *Gossypium hirsutum* | Gohir.D06G030500 |  | *Spirodela polyrhiza* | Spipo12G0020000 |
| *Gossypium hirsutum* | Gohir.D07G037800 |  | *Spirodela polyrhiza* | Spipo12G0052800 |
| *Gossypium hirsutum* | Gohir.D07G093100 |  | *Spirodela polyrhiza* | Spipo12G0060200 |
| *Gossypium hirsutum* | Gohir.D07G098900 |  | *Spirodela polyrhiza* | Spipo14G0027800 |
| *Gossypium hirsutum* | Gohir.D07G170100 |  | *Spirodela polyrhiza* | Spipo15G0031700 |
| *Gossypium hirsutum* | Gohir.D07G199600 |  | *Spirodela polyrhiza* | Spipo16G0014400 |
| *Gossypium hirsutum* | Gohir.D08G032600 |  | *Spirodela polyrhiza* | Spipo17G0024000 |
| *Gossypium hirsutum* | Gohir.D08G059100 |  | *Spirodela polyrhiza* | Spipo18G0016800 |
| *Gossypium hirsutum* | Gohir.D08G096200 |  | *Spirodela polyrhiza* | Spipo1G0083700 |
| *Gossypium hirsutum* | Gohir.D08G104200 |  | *Spirodela polyrhiza* | Spipo22G0034500 |
| *Gossypium hirsutum* | Gohir.D08G105900 |  | *Spirodela polyrhiza* | Spipo23G0029000 |
| *Gossypium hirsutum* | Gohir.D08G137300 |  | *Spirodela polyrhiza* | Spipo28G0017700 |
| *Gossypium hirsutum* | Gohir.D08G202600 |  | *Spirodela polyrhiza* | Spipo29G0015300 |
| *Gossypium hirsutum* | Gohir.D09G031500 |  | *Spirodela polyrhiza* | Spipo4G0060000 |
| *Gossypium hirsutum* | Gohir.D09G048500 |  | *Spirodela polyrhiza* | Spipo5G0007300 |
| *Gossypium hirsutum* | Gohir.D09G058800 |  | *Spirodela polyrhiza* | Spipo6G0043800 |
| *Gossypium hirsutum* | Gohir.D09G156300 |  | *Theobroma cacao* | Thecc1EG002458t1 |
| *Gossypium hirsutum* | Gohir.D09G186300 |  | *Theobroma cacao* | Thecc1EG004548t1 |
| *Gossypium hirsutum* | Gohir.D10G158700 |  | *Theobroma cacao* | Thecc1EG005034t1 |
| *Gossypium hirsutum* | Gohir.D11G014800 |  | *Theobroma cacao* | Thecc1EG007358t1 |
| *Gossypium hirsutum* | Gohir.D11G016300 |  | *Theobroma cacao* | Thecc1EG007360t1 |
| *Gossypium hirsutum* | Gohir.D11G105700 |  | *Theobroma cacao* | Thecc1EG007770t1 |
| *Gossypium hirsutum* | Gohir.D11G154500 |  | *Theobroma cacao* | Thecc1EG008446t1 |
| *Gossypium hirsutum* | Gohir.D11G344400 |  | *Theobroma cacao* | Thecc1EG009367t2 |
| *Gossypium hirsutum* | Gohir.D12G008500 |  | *Theobroma cacao* | Thecc1EG009901t1 |
| *Gossypium hirsutum* | Gohir.D12G017600 |  | *Theobroma cacao* | Thecc1EG012333t1 |
| *Gossypium hirsutum* | Gohir.D12G056900 |  | *Theobroma cacao* | Thecc1EG014102t1 |
| *Gossypium hirsutum* | Gohir.D12G232100 |  | *Theobroma cacao* | Thecc1EG014441t1 |
| *Gossypium hirsutum* | Gohir.D12G247100 |  | *Theobroma cacao* | Thecc1EG015399t1 |
| *Gossypium hirsutum* | Gohir.D12G251200 |  | *Theobroma cacao* | Thecc1EG015513t1 |
| *Gossypium hirsutum* | Gohir.D13G049200 |  | *Theobroma cacao* | Thecc1EG016302t1 |
| *Gossypium hirsutum* | Gohir.D13G062900 |  | *Theobroma cacao* | Thecc1EG017038t1 |
| *Gossypium hirsutum* | Gohir.D13G186900 |  | *Theobroma cacao* | Thecc1EG017297t1 |
| *Gossypium hirsutum* | Gohir.D13G215600 |  | *Theobroma cacao* | Thecc1EG019107t1 |
| *Gossypium raimondii* | Gorai.001G039500 |  | *Theobroma cacao* | Thecc1EG020983t1 |
| *Gossypium raimondii* | Gorai.001G099700 |  | *Theobroma cacao* | Thecc1EG022066t1 |
| *Gossypium raimondii* | Gorai.001G106600 |  | *Theobroma cacao* | Thecc1EG027858t1 |
| *Gossypium raimondii* | Gorai.001G190000 |  | *Theobroma cacao* | Thecc1EG032561t1 |
| *Gossypium raimondii* | Gorai.001G223800 |  | *Theobroma cacao* | Thecc1EG034530t1 |
| *Gossypium raimondii* | Gorai.002G022300 |  | *Theobroma cacao* | Thecc1EG034531t1 |
| *Gossypium raimondii* | Gorai.002G104600 |  | *Theobroma cacao* | Thecc1EG036860t1 |
| *Gossypium raimondii* | Gorai.002G138300 |  | *Theobroma cacao* | Thecc1EG038829t1 |
| *Gossypium raimondii* | Gorai.002G218400 |  | *Theobroma cacao* | Thecc1EG039452t1 |
| *Gossypium raimondii* | Gorai.003G098300 |  | *Theobroma cacao* | Thecc1EG041942t1 |
| *Gossypium raimondii* | Gorai.003G104600 |  | *Theobroma cacao* | Thecc1EG041985t1 |
| *Gossypium raimondii* | Gorai.003G138200 |  | *Theobroma cacao* | Thecc1EG042213t1 |
| *Gossypium raimondii* | Gorai.004G030300 |  | *Theobroma cacao* | Thecc1EG043038t1 |
| *Gossypium raimondii* | Gorai.004G061000 |  | *Trifolium pratense* | Tp57577_TGAC_v2_mRNA1095 |
| *Gossypium raimondii* | Gorai.004G102000 |  | *Trifolium pratense* | Tp57577_TGAC_v2_mRNA11947 |
| *Gossypium raimondii* | Gorai.004G111500 |  | *Trifolium pratense* | Tp57577_TGAC_v2_mRNA12224 |
| *Gossypium raimondii* | Gorai.004G113000 |  | *Trifolium pratense* | Tp57577_TGAC_v2_mRNA12225 |
| *Gossypium raimondii* | Gorai.004G140600 |  | *Trifolium pratense* | Tp57577_TGAC_v2_mRNA12226 |
| *Gossypium raimondii* | Gorai.004G214000 |  | *Trifolium pratense* | Tp57577_TGAC_v2_mRNA12435 |
| *Gossypium raimondii* | Gorai.005G053500 |  | *Trifolium pratense* | Tp57577_TGAC_v2_mRNA13371 |
| *Gossypium raimondii* | Gorai.005G097100 |  | *Trifolium pratense* | Tp57577_TGAC_v2_mRNA1515 |
| *Gossypium raimondii* | Gorai.005G195800 |  | *Trifolium pratense* | Tp57577_TGAC_v2_mRNA1876 |
| *Gossypium raimondii* | Gorai.005G209100 |  | *Trifolium pratense* | Tp57577_TGAC_v2_mRNA20517 |
| *Gossypium raimondii* | Gorai.005G226000 |  | *Trifolium pratense* | Tp57577_TGAC_v2_mRNA24104 |
| *Gossypium raimondii* | Gorai.006G036400 |  | *Trifolium pratense* | Tp57577_TGAC_v2_mRNA24105 |
| *Gossypium raimondii* | Gorai.006G061600 |  | *Trifolium pratense* | Tp57577_TGAC_v2_mRNA24806 |
| *Gossypium raimondii* | Gorai.006G073000 |  | *Trifolium pratense* | Tp57577_TGAC_v2_mRNA28409 |
| *Gossypium raimondii* | Gorai.006G177800 |  | *Trifolium pratense* | Tp57577_TGAC_v2_mRNA2924 |
| *Gossypium raimondii* | Gorai.006G209000 |  | *Trifolium pratense* | Tp57577_TGAC_v2_mRNA29819 |
| *Gossypium raimondii* | Gorai.007G015200 |  | *Trifolium pratense* | Tp57577_TGAC_v2_mRNA3062 |
| *Gossypium raimondii* | Gorai.007G017100 |  | *Trifolium pratense* | Tp57577_TGAC_v2_mRNA30727 |
| *Gossypium raimondii* | Gorai.007G023900 |  | *Trifolium pratense* | Tp57577_TGAC_v2_mRNA31914 |
| *Gossypium raimondii* | Gorai.007G113000 |  | *Trifolium pratense* | Tp57577_TGAC_v2_mRNA33383 |
| *Gossypium raimondii* | Gorai.007G164600 |  | *Trifolium pratense* | Tp57577_TGAC_v2_mRNA33384 |
| *Gossypium raimondii* | Gorai.007G374800 |  | *Trifolium pratense* | Tp57577_TGAC_v2_mRNA3378 |
| *Gossypium raimondii* | Gorai.008G008400 |  | *Trifolium pratense* | Tp57577_TGAC_v2_mRNA33790 |
| *Gossypium raimondii* | Gorai.008G017800 |  | *Trifolium pratense* | Tp57577_TGAC_v2_mRNA34087 |
| *Gossypium raimondii* | Gorai.008G059900 |  | *Trifolium pratense* | Tp57577_TGAC_v2_mRNA34992 |
| *Gossypium raimondii* | Gorai.008G248400 |  | *Trifolium pratense* | Tp57577_TGAC_v2_mRNA40145 |
| *Gossypium raimondii* | Gorai.008G265100 |  | *Trifolium pratense* | Tp57577_TGAC_v2_mRNA4279 |
| *Gossypium raimondii* | Gorai.008G269300 |  | *Trifolium pratense* | Tp57577_TGAC_v2_mRNA4937 |
| *Gossypium raimondii* | Gorai.009G002400 |  | *Trifolium pratense* | Tp57577_TGAC_v2_mRNA5475 |
| *Gossypium raimondii* | Gorai.009G065600 |  | *Trifolium pratense* | Tp57577_TGAC_v2_mRNA5943 |
| *Gossypium raimondii* | Gorai.009G103100 |  | *Trifolium pratense* | Tp57577_TGAC_v2_mRNA683 |
| *Gossypium raimondii* | Gorai.009G103700 |  | *Trifolium pratense* | Tp57577_TGAC_v2_mRNA6932 |
| *Gossypium raimondii* | Gorai.009G113000 |  | *Triticum aestivum* | Traes_1AL_24A602D25.2 |
| *Gossypium raimondii* | Gorai.009G279200 |  | *Triticum aestivum* | Traes_1AL_2EE300C85.1 |
| *Gossypium raimondii* | Gorai.009G338600 |  | *Triticum aestivum* | Traes_1AL_91AA77622.2 |
| *Gossypium raimondii* | Gorai.009G353200 |  | *Triticum aestivum* | Traes_1AL_A2CED32B7.1 |
| *Gossypium raimondii* | Gorai.010G033400 |  | *Triticum aestivum* | Traes_1AL_F4FE96E3B.1 |
| *Gossypium raimondii* | Gorai.010G033500 |  | *Triticum aestivum* | Traes_1BL_244DA8D2D.1 |
| *Gossypium raimondii* | Gorai.010G245200 |  | *Triticum aestivum* | Traes_1BL_688EF6A1A.1 |
| *Gossypium raimondii* | Gorai.011G178300 |  | *Triticum aestivum* | Traes_1BL_843D97D6C.1 |
| *Gossypium raimondii* | Gorai.012G001000 |  | *Triticum aestivum* | Traes_1BL_A2968F649.1 |
| *Gossypium raimondii* | Gorai.012G003000 |  | *Triticum aestivum* | Traes_1DL_531B7FA16.1 |
| *Gossypium raimondii* | Gorai.012G083100 |  | *Triticum aestivum* | Traes_1DL_797D879AB.1 |
| *Gossypium raimondii* | Gorai.012G092600 |  | *Triticum aestivum* | Traes_1DL_8A2869298.1 |
| *Gossypium raimondii* | Gorai.013G055000 |  | *Triticum aestivum* | Traes_1DL_9BA0850ED.1 |
| *Gossypium raimondii* | Gorai.013G071300 |  | *Triticum aestivum* | Traes_2AL_0E3F538E0.1 |
| *Gossypium raimondii* | Gorai.013G246000 |  | *Triticum aestivum* | Traes_2AS_2FCD59730.1 |
| *Helianthus annuus* | HanXRQChr01g0022961 |  | *Triticum aestivum* | Traes_2BL_119814659.1 |
| *Helianthus annuus* | HanXRQChr01g0027331 |  | *Triticum aestivum* | Traes_2BL_98439EA10.1 |
| *Helianthus annuus* | HanXRQChr02g0043181 |  | *Triticum aestivum* | Traes_2BS_8BED816B1.1 |
| *Helianthus annuus* | HanXRQChr02g0059301 |  | *Triticum aestivum* | Traes_2BS_B01D70A77.1 |
| *Helianthus annuus* | HanXRQChr03g0065501 |  | *Triticum aestivum* | Traes_2DS_2A961F39D.1 |
| *Helianthus annuus* | HanXRQChr03g0085921 |  | *Triticum aestivum* | Traes_3B_41DBECC0E.1 |
| *Helianthus annuus* | HanXRQChr04g0100211 |  | *Triticum aestivum* | Traes_4AL_406C57F67.1 |
| *Helianthus annuus* | HanXRQChr04g0121971 |  | *Triticum aestivum* | Traes_4AL_72C3E1EAD.1 |
| *Helianthus annuus* | HanXRQChr04g0126381 |  | *Triticum aestivum* | Traes_4AL_B6992AAA6.1 |
| *Helianthus annuus* | HanXRQChr05g0137501 |  | *Triticum aestivum* | Traes_4AS_512DDDECB.1 |
| *Helianthus annuus* | HanXRQChr05g0157831 |  | *Triticum aestivum* | Traes_4AS_B2CDBFB54.1 |
| *Helianthus annuus* | HanXRQChr06g0169231 |  | *Triticum aestivum* | Traes_4BL_1FE4A71E6.1 |
| *Helianthus annuus* | HanXRQChr06g0182791 |  | *Triticum aestivum* | Traes_4BL_430501C10.1 |
| *Helianthus annuus* | HanXRQChr07g0200931 |  | *Triticum aestivum* | Traes_4BL_979083044.1 |
| *Helianthus annuus* | HanXRQChr07g0206531 |  | *Triticum aestivum* | Traes_4BS_46930D906.1 |
| *Helianthus annuus* | HanXRQChr08g0210141 |  | *Triticum aestivum* | Traes_4BS_4BA543232.1 |
| *Helianthus annuus* | HanXRQChr08g0213841 |  | *Triticum aestivum* | Traes_4BS_4BCA03A6C.1 |
| *Helianthus annuus* | HanXRQChr08g0219331 |  | *Triticum aestivum* | Traes_4BS_8C20E76AA.1 |
| *Helianthus annuus* | HanXRQChr08g0225931 |  | *Triticum aestivum* | Traes_4BS_ACD70539F.1 |
| *Helianthus annuus* | HanXRQChr08g0228701 |  | *Triticum aestivum* | Traes_4DL_19236866D.1 |
| *Helianthus annuus* | HanXRQChr08g0230561 |  | *Triticum aestivum* | Traes_4DL_B337DEEC7.1 |
| *Helianthus annuus* | HanXRQChr08g0236641 |  | *Triticum aestivum* | Traes_4DL_EE41726EA.1 |
| *Helianthus annuus* | HanXRQChr08g0236661 |  | *Triticum aestivum* | Traes_4DS_3D34E7E83.1 |
| *Helianthus annuus* | HanXRQChr09g0245431 |  | *Triticum aestivum* | Traes_4DS_50A614BFB.1 |
| *Helianthus annuus* | HanXRQChr09g0257381 |  | *Triticum aestivum* | Traes_4DS_A8B6C23C4.1 |
| *Helianthus annuus* | HanXRQChr09g0263321 |  | *Triticum aestivum* | Traes_4DS_AFCEDDE67.1 |
| *Helianthus annuus* | HanXRQChr10g0283841 |  | *Triticum aestivum* | Traes_5AL_2525FF4C7.1 |
| *Helianthus annuus* | HanXRQChr10g0297451 |  | *Triticum aestivum* | Traes_5AL_852A1474C.1 |
| *Helianthus annuus* | HanXRQChr10g0297751 |  | *Triticum aestivum* | Traes_5BL_417551368.1 |
| *Helianthus annuus* | HanXRQChr10g0306781 |  | *Triticum aestivum* | Traes_5BL_7C6818E7C.1 |
| *Helianthus annuus* | HanXRQChr10g0308851 |  | *Triticum aestivum* | Traes_5BL_DB6E9FDB9.2 |
| *Helianthus annuus* | HanXRQChr11g0329481 |  | *Triticum aestivum* | Traes_5DL_50FD4CFF8.1 |
| *Helianthus annuus* | HanXRQChr11g0335061 |  | *Triticum aestivum* | Traes_5DL_8CE2482E6.1 |
| *Helianthus annuus* | HanXRQChr12g0358091 |  | *Triticum aestivum* | Traes_5DL_BB69EAD36.1 |
| *Helianthus annuus* | HanXRQChr12g0370381 |  | *Triticum aestivum* | Traes_6AL_595777E8B.1 |
| *Helianthus annuus* | HanXRQChr12g0374051 |  | *Triticum aestivum* | Traes_6AL_A0A31AA9F.1 |
| *Helianthus annuus* | HanXRQChr13g0389911 |  | *Triticum aestivum* | Traes_6AS_4106E8E28.1 |
| *Helianthus annuus* | HanXRQChr13g0393101 |  | *Triticum aestivum* | Traes_6AS_8FFD6CC72.1 |
| *Helianthus annuus* | HanXRQChr13g0394131 |  | *Triticum aestivum* | Traes_6BL_6ECA375B9.1 |
| *Helianthus annuus* | HanXRQChr13g0410811 |  | *Triticum aestivum* | Traes_6BL_ED40C8806.1 |
| *Helianthus annuus* | HanXRQChr14g0434831 |  | *Triticum aestivum* | Traes_6BS_AB5909D02.1 |
| *Helianthus annuus* | HanXRQChr14g0448001 |  | *Triticum aestivum* | Traes_6BS_FB06D4612.1 |
| *Helianthus annuus* | HanXRQChr14g0453441 |  | *Triticum aestivum* | Traes_6DL_6FAD063EE.1 |
| *Helianthus annuus* | HanXRQChr14g0460851 |  | *Triticum aestivum* | Traes_6DL_9CC066885.1 |
| *Helianthus annuus* | HanXRQChr15g0465341 |  | *Triticum aestivum* | Traes_6DL_C215BACFD.1 |
| *Helianthus annuus* | HanXRQChr15g0466171 |  | *Triticum aestivum* | Traes_6DS_5E37DA72B.1 |
| *Helianthus annuus* | HanXRQChr15g0474581 |  | *Triticum aestivum* | Traes_6DS_6268D31AE.1 |
| *Helianthus annuus* | HanXRQChr15g0475781 |  | *Triticum aestivum* | Traes_7AL_04A38CA6B.1 |
| *Helianthus annuus* | HanXRQChr15g0497461 |  | *Triticum aestivum* | Traes_7AL_304975395.1 |
| *Helianthus annuus* | HanXRQChr16g0514521 |  | *Triticum aestivum* | Traes_7AL_8783C1471.1 |
| *Helianthus annuus* | HanXRQChr17g0547741 |  | *Triticum aestivum* | Traes_7AL_EA6F4FFDE.1 |
| *Helianthus annuus* | HanXRQChr17g0549411 |  | *Triticum aestivum* | Traes_7AS_B2DC3787B.1 |
| *Helianthus annuus* | HanXRQChr17g0552071 |  | *Triticum aestivum* | Traes_7AS_DEB4FC0D6.1 |
| *Hordeum vulgare* | HORVU0Hr1G021050.2 |  | *Triticum aestivum* | Traes_7AS_F46AC277B.1 |
| *Hordeum vulgare* | HORVU1Hr1G039150.1 |  | *Triticum aestivum* | Traes_7BL_7DC689032.1 |
| *Hordeum vulgare* | HORVU1Hr1G056120.2 |  | *Triticum aestivum* | Traes_7BL_D111CAEF4.1 |
| *Hordeum vulgare* | HORVU1Hr1G095390.1 |  | *Triticum aestivum* | Traes_7BS_673360C6B.1 |
| *Hordeum vulgare* | HORVU1Hr1G095410.2 |  | *Triticum aestivum* | Traes_7BS_C655E6C9B.1 |
| *Hordeum vulgare* | HORVU2Hr1G013400.32 | | *Triticum aestivum* | Traes_7BS_E337E82FB.1 |
| *Hordeum vulgare* | HORVU2Hr1G020760.2 |  | *Triticum aestivum* | Traes_7DL_384E3B098.1 |
| *Hordeum vulgare* | HORVU2Hr1G085910.21 | | *Triticum aestivum* | Traes_7DL_A5ECEDA95.1 |
| *Hordeum vulgare* | HORVU3Hr1G087180.2 |  | *Triticum aestivum* | Traes_7DL_D8DEE65D6.1 |
| *Hordeum vulgare* | HORVU4Hr1G004080.10 | | *Triticum aestivum* | Traes_7DL_E14B4063D.1 |
| *Hordeum vulgare* | HORVU4Hr1G007420.1 |  | *Triticum aestivum* | Traes_7DS_46E811D74.1 |
| *Hordeum vulgare* | HORVU4Hr1G010840.1 |  | *Triticum aestivum* | Traes_7DS_5F61859E4.1 |
| *Hordeum vulgare* | HORVU4Hr1G021000.2 |  | *Triticum aestivum* | Traes_7DS_9B463F375.1 |
| *Hordeum vulgare* | HORVU4Hr1G024330.1 |  | *Vigna unguiculata* | Vigun01g054600 |
| *Hordeum vulgare* | HORVU4Hr1G048700.1 |  | *Vigna unguiculata* | Vigun01g065500 |
| *Hordeum vulgare* | HORVU4Hr1G057550.2 |  | *Vigna unguiculata* | Vigun01g075800 |
| *Hordeum vulgare* | HORVU4Hr1G084020.11 | | *Vigna unguiculata* | Vigun01g187100 |
| *Hordeum vulgare* | HORVU5Hr1G049310.1 |  | *Vigna unguiculata* | Vigun02g042100 |
| *Hordeum vulgare* | HORVU5Hr1G049330.1 |  | *Vigna unguiculata* | Vigun02g177100 |
| *Hordeum vulgare* | HORVU5Hr1G051230.1 |  | *Vigna unguiculata* | Vigun03g017700 |
| *Hordeum vulgare* | HORVU5Hr1G063640.1 |  | *Vigna unguiculata* | Vigun03g042600 |
| *Hordeum vulgare* | HORVU5Hr1G081620.1 |  | *Vigna unguiculata* | Vigun03g124600 |
| *Hordeum vulgare* | HORVU6Hr1G021460.1 |  | *Vigna unguiculata* | Vigun03g337800 |
| *Hordeum vulgare* | HORVU6Hr1G021570.1 |  | *Vigna unguiculata* | Vigun03g379600 |
| *Hordeum vulgare* | HORVU6Hr1G030080.3 |  | *Vigna unguiculata* | Vigun03g387700 |
| *Hordeum vulgare* | HORVU6Hr1G056000.2 |  | *Vigna unguiculata* | Vigun04g021700 |
| *Hordeum vulgare* | HORVU6Hr1G057630.1 |  | *Vigna unguiculata* | Vigun04g068400 |
| *Hordeum vulgare* | HORVU6Hr1G071950.10 | | *Vigna unguiculata* | Vigun05g225000 |
| *Hordeum vulgare* | HORVU6Hr1G072620.8 |  | *Vigna unguiculata* | Vigun05g253600 |
| *Hordeum vulgare* | HORVU6Hr1G073170.1 |  | *Vigna unguiculata* | Vigun06g006800 |
| *Hordeum vulgare* | HORVU7Hr1G027560.1 |  | *Vigna unguiculata* | Vigun07g011700 |
| *Hordeum vulgare* | HORVU7Hr1G043030.14 | | *Vigna unguiculata* | Vigun07g078600 |
| *Hordeum vulgare* | HORVU7Hr1G074960.3 |  | *Vigna unguiculata* | Vigun07g116500 |
| *Hordeum vulgare* | HORVU7Hr1G097630.1 |  | *Vigna unguiculata* | Vigun07g181300 |
| *Hordeum vulgare* | HORVU7Hr1G097840.1 |  | *Vigna unguiculata* | Vigun07g270500 |
| *Kalanchoe fedtschenkoi* | Kaladp0001s0264 |  | *Vigna unguiculata* | Vigun08g080300 |
| *Kalanchoe fedtschenkoi* | Kaladp0005s0054 |  | *Vigna unguiculata* | Vigun08g109700 |
| *Kalanchoe fedtschenkoi* | Kaladp0011s0227 |  | *Vigna unguiculata* | Vigun08g116900 |
| *Kalanchoe fedtschenkoi* | Kaladp0011s0957 |  | *Vigna unguiculata* | Vigun08g124000 |
| *Kalanchoe fedtschenkoi* | Kaladp0029s0144 |  | *Vigna unguiculata* | Vigun08g127200 |
| *Kalanchoe fedtschenkoi* | Kaladp0031s0063 |  | *Vigna unguiculata* | Vigun09g004700 |
| *Kalanchoe fedtschenkoi* | Kaladp0032s0115 |  | *Vigna unguiculata* | Vigun09g076300 |
| *Kalanchoe fedtschenkoi* | Kaladp0032s0319 |  | *Vigna unguiculata* | Vigun09g140300 |
| *Kalanchoe fedtschenkoi* | Kaladp0039s0496 |  | *Vigna unguiculata* | Vigun09g150200 |
| *Kalanchoe fedtschenkoi* | Kaladp0040s0446 |  | *Vigna unguiculata* | Vigun09g180800 |
| *Kalanchoe fedtschenkoi* | Kaladp0042s0203 |  | *Vigna unguiculata* | Vigun09g180900 |
| *Kalanchoe fedtschenkoi* | Kaladp0045s0272 |  | *Vigna unguiculata* | Vigun09g217500 |
| *Kalanchoe fedtschenkoi* | Kaladp0047s0094 |  | *Vigna unguiculata* | Vigun09g276400 |
| *Kalanchoe fedtschenkoi* | Kaladp0048s0472 |  | *Vigna unguiculata* | Vigun10g152100 |
| *Kalanchoe fedtschenkoi* | Kaladp0048s0596 |  | *Vigna unguiculata* | Vigun11g149100 |
| *Kalanchoe fedtschenkoi* | Kaladp0050s0175 |  | *Vigna unguiculata* | Vigun11g150000 |
| *Kalanchoe fedtschenkoi* | Kaladp0050s0302 |  | *Vitis vinifera* | GSVIVT01000951001 |
| *Kalanchoe fedtschenkoi* | Kaladp0052s0007 |  | *Vitis vinifera* | GSVIVT01003473001 |
| *Kalanchoe fedtschenkoi* | Kaladp0058s0661 |  | *Vitis vinifera* | GSVIVT01003983001 |
| *Kalanchoe fedtschenkoi* | Kaladp0059s0317 |  | *Vitis vinifera* | GSVIVT01007965001 |
| *Kalanchoe fedtschenkoi* | Kaladp0060s0443 |  | *Vitis vinifera* | GSVIVT01009197001 |
| *Kalanchoe fedtschenkoi* | Kaladp0066s0114 |  | *Vitis vinifera* | GSVIVT01009198001 |
| *Kalanchoe fedtschenkoi* | Kaladp0071s0246 |  | *Vitis vinifera* | GSVIVT01010855001 |
| *Kalanchoe fedtschenkoi* | Kaladp0076s0405 |  | *Vitis vinifera* | GSVIVT01010991001 |
| *Kalanchoe fedtschenkoi* | Kaladp0081s0148 |  | *Vitis vinifera* | GSVIVT01011820001 |
| *Kalanchoe fedtschenkoi* | Kaladp0087s0131 |  | *Vitis vinifera* | GSVIVT01011897001 |
| *Kalanchoe fedtschenkoi* | Kaladp0088s0022 |  | *Vitis vinifera* | GSVIVT01012518001 |
| *Kalanchoe fedtschenkoi* | Kaladp0095s0551 |  | *Vitis vinifera* | GSVIVT01019393001 |
| *Kalanchoe fedtschenkoi* | Kaladp0095s0743 |  | *Vitis vinifera* | GSVIVT01020352001 |
| *Kalanchoe fedtschenkoi* | Kaladp0099s0080 |  | *Vitis vinifera* | GSVIVT01024661001 |
| *Kalanchoe fedtschenkoi* | Kaladp0101s0041 |  | *Vitis vinifera* | GSVIVT01025065001 |
| *Kalanchoe fedtschenkoi* | Kaladp0102s0039 |  | *Vitis vinifera* | GSVIVT01025087001 |
| *Kalanchoe fedtschenkoi* | Kaladp0809s0081 |  | *Vitis vinifera* | GSVIVT01027443001 |
| *Kalanchoe fedtschenkoi* | Kaladp0809s0114 |  | *Vitis vinifera* | GSVIVT01027910001 |
| *Kalanchoe fedtschenkoi* | Kaladp0833s0004 |  | *Vitis vinifera* | GSVIVT01029593001 |
| *Kalanchoe fedtschenkoi* | Kaladp0878s0025 |  | *Vitis vinifera* | GSVIVT01030127001 |
| *Kalanchoe laxiflora* | Kalax.0002s0239 |  | *Vitis vinifera* | GSVIVT01030628001 |
| *Kalanchoe laxiflora* | Kalax.0008s0180 |  | *Vitis vinifera* | GSVIVT01032644001 |
| *Kalanchoe laxiflora* | Kalax.0012s0112 |  | *Vitis vinifera* | GSVIVT01033017001 |
| *Kalanchoe laxiflora* | Kalax.0012s0205 |  | *Vitis vinifera* | GSVIVT01036037001 |
| *Kalanchoe laxiflora* | Kalax.0012s0256 |  | *Vitis vinifera* | GSVIVT01036499001 |
| *Kalanchoe laxiflora* | Kalax.0013s0056 |  | *Vitis vinifera* | GSVIVT01038647001 |
| *Kalanchoe laxiflora* | Kalax.0014s0187 |  | *Vitis vinifera* | GSVIVT01038707001 |
| *Kalanchoe laxiflora* | Kalax.0020s0086 |  | *Zostera marina* | Zosma103g00180 |
| *Kalanchoe laxiflora* | Kalax.0024s0012 |  | *Zostera marina* | Zosma105g00170 |
| *Kalanchoe laxiflora* | Kalax.0027s0020 |  | *Zostera marina* | Zosma123g00020 |
| *Kalanchoe laxiflora* | Kalax.0044s0037 |  | *Zostera marina* | Zosma144g00090 |
| *Kalanchoe laxiflora* | Kalax.0057s0142 |  | *Zostera marina* | Zosma19g00510 |
| *Kalanchoe laxiflora* | Kalax.0060s0059 |  | *Zostera marina* | Zosma218g00180 |
| *Kalanchoe laxiflora* | Kalax.0067s0044 |  | *Zostera marina* | Zosma261g00090 |
| *Kalanchoe laxiflora* | Kalax.0085s0074 |  | *Zostera marina* | Zosma261g00110 |
| *Kalanchoe laxiflora* | Kalax.0091s0009 |  | *Zostera marina* | Zosma307g00140 |
| *Kalanchoe laxiflora* | Kalax.0101s0050 |  | *Zostera marina* | Zosma425g00060 |
| *Kalanchoe laxiflora* | Kalax.0102s0101 |  | *Zostera marina* | Zosma47g00320 |
| *Kalanchoe laxiflora* | Kalax.0109s0042 |  | *Zostera marina* | Zosma4g02070 |
| *Kalanchoe laxiflora* | Kalax.0114s0058 |  | *Zostera marina* | Zosma58g00530 |
| *Kalanchoe laxiflora* | Kalax.0120s0007 |  | *Zostera marina* | Zosma7g01370 |
| *Kalanchoe laxiflora* | Kalax.0124s0057 |  | *Zostera marina* | Zosma86g00040 |
| *Kalanchoe laxiflora* | Kalax.0131s0037 |  | *Zostera marina* | Zosma87g00760 |
| *Kalanchoe laxiflora* | Kalax.0136s0104 |  | *Zostera marina* | Zosma91g00340 |
| *Kalanchoe laxiflora* | Kalax.0137s0069 |  | *Zostera marina* | Zosma9g00420 |
| *Kalanchoe laxiflora* | Kalax.0150s0004 |  | *Zostera marina* | Zosma9g01100 |
| *Kalanchoe laxiflora* | Kalax.0172s0016 |  |  |  |
| *Kalanchoe laxiflora* | Kalax.0177s0061 |  |  |  |
| *Kalanchoe laxiflora* | Kalax.0179s0003 |  |  |  |
| *Kalanchoe laxiflora* | Kalax.0202s0006 |  |  |  |
| *Kalanchoe laxiflora* | Kalax.0205s0038 |  |  |  |
| *Kalanchoe laxiflora* | Kalax.0215s0004 |  |  |  |
| *Kalanchoe laxiflora* | Kalax.0216s0009 |  |  |  |
| *Kalanchoe laxiflora* | Kalax.0218s0020 |  |  |  |
| *Kalanchoe laxiflora* | Kalax.0260s0038 |  |  |  |
| *Kalanchoe laxiflora* | Kalax.0278s0023 |  |  |  |
|  |  |  |  |  |
