# Supplementary material for: Analyses of open-access multi-omics data sets reveal genetic and expression characteristics of maize ZmCCT family genes
Source: AoB Plants. 2021 Aug 16;13(5):plab048. doi: 10.1093/aobpla/plab048 (PMC8459886; doi:10.1093/aobpla/plab048)
Supplement: plab048_suppl_Supplementary_Table_S3 [file plab048_suppl_supplementary_table_s3.docx]

**Table S3** Accession no. of *ZmCCT*s in maize inbred lines of B73, Huangzao4, W22, Mo17 and SK

*Note:* ZmCCT, Maize CCT domain-containing protein; *ZmCCT*, ZmCCT gene

| **B73** | |  | **W22** | |  | **Mo17** | |
| --- | --- | --- | --- | --- | --- | --- | --- |
| ***ZmCCT*** | **Gene ID** |  | ***ZmCCT*** | **Gene ID** |  | ***ZmCCT*** | **Gene ID** |
| *ZmCCT1* | Zm00001d027598 |  | *ZmCCT1* | Zm00004b001750 |  | *ZmCCT1* | Zm00014a001344 |
| *ZmCCT2* | Zm00001d029149 |  | *ZmCCT2* | Zm00004b002906 |  | *ZmCCT2* | Zm00014a001768 |
| *ZmCCT3* | Zm00001d029885 |  | *ZmCCT3* | Zm00004b004370 |  | *ZmCCT3* | Zm00014a002554 |
| *ZmCCT4* | Zm00001d030229 |  | *ZmCCT4* | Zm00004b004371 |  | *ZmCCT4* | Zm00014a004613 |
| *ZmCCT5* | Zm00001d031662 |  | *ZmCCT5* | Zm00004b004751 |  | *ZmCCT5* | Zm00014a004791 |
| *ZmCCT6* | Zm00001d032768 |  | *ZmCCT6* | Zm00004b004911 |  | *ZmCCT6* | Zm00014a004856 |
| *ZmCCT7* | Zm00001d033523 |  | *ZmCCT7* | Zm00004b005492 |  | *ZmCCT7* | Zm00014a006020 |
| *ZmCCT8* | Zm00001d033719 |  | *ZmCCT8* | Zm00004b006088 |  | *ZmCCT8* | Zm00014a006238 |
| *ZmCCT9* | Zm00001d003162 |  | *ZmCCT9* | Zm00004b006410 |  | *ZmCCT9* | Zm00014a006615 |
| *ZmCCT10* | Zm00001d003571 |  | *ZmCCT10* | Zm00004b007041 |  | *ZmCCT10* | Zm00014a009007 |
| *ZmCCT11* | Zm00001d004875 |  | *ZmCCT11* | Zm00004b007307 |  | *ZmCCT11* | Zm00014a010332 |
| *ZmCCT12* | Zm00001d006212 |  | *ZmCCT12* | Zm00004b007640 |  | *ZmCCT12* | Zm00014a010338 |
| *ZmCCT13* | Zm00001d007107 |  | *ZmCCT13* | Zm00004b007842 |  | *ZmCCT13* | Zm00014a013367 |
| *ZmCCT14* | Zm00001d007240 |  | *ZmCCT14* | Zm00004b007925 |  | *ZmCCT14* | Zm00014a014945 |
| *ZmCCT15* | Zm00001d042958 |  | *ZmCCT15* | Zm00004b008343 |  | *ZmCCT15* | Zm00014a015792 |
| *ZmCCT16* | Zm00001d043783 |  | *ZmCCT16* | Zm00004b008980 |  | *ZmCCT16* | Zm00014a017484 |
| *ZmCCT17* | Zm00001d044598 |  | *ZmCCT17* | Zm00004b009062 |  | *ZmCCT17* | Zm00014a017805 |
| *ZmCCT18* | Zm00001d049347 |  | *ZmCCT18* | Zm00004b009398 |  | *ZmCCT18* | Zm00014a017907 |
| *ZmCCT19* | Zm00001d049651 |  | *ZmCCT19* | Zm00004b009908 |  | *ZmCCT19* | Zm00014a020188 |
| *ZmCCT20* | Zm00001d051047 |  | *ZmCCT20* | Zm00004b011299 |  | *ZmCCT20* | Zm00014a021914 |
| *ZmCCT21* | Zm00001d051114 |  | *ZmCCT21* | Zm00004b011390 |  | *ZmCCT21* | Zm00014a022016 |
| *ZmCCT22* | Zm00001d051684 |  | *ZmCCT22* | Zm00004b012083 |  | *ZmCCT22* | Zm00014a022056 |
| *ZmCCT23* | Zm00001d052781 |  | *ZmCCT23* | Zm00004b012084 |  | *ZmCCT23* | Zm00014a023131 |
| *ZmCCT24* | Zm00001d053880 |  | *ZmCCT24* | Zm00004b012087 |  | *ZmCCT24* | Zm00014a023702 |
| *ZmCCT25* | Zm00001d013331 |  | *ZmCCT25* | Zm00004b012217 |  | *ZmCCT25* | Zm00014a024089 |
| *ZmCCT26* | Zm00001d013443 |  | *ZmCCT26* | Zm00004b012386 |  | *ZmCCT26* | Zm00014a025483 |
| *ZmCCT27* | Zm00001d014074 |  | *ZmCCT27* | Zm00004b012887 |  | *ZmCCT27* | Zm00014a026685 |
| *ZmCCT28* | Zm00001d014656 |  | *ZmCCT28* | Zm00004b013036 |  | *ZmCCT28* | Zm00014a028130 |
| *ZmCCT29* | Zm00001d014963 |  | *ZmCCT29* | Zm00004b013059 |  | *ZmCCT29* | Zm00014a030637 |
| *ZmCCT30* | Zm00001d015268 |  | *ZmCCT30* | Zm00004b013662 |  | *ZmCCT30* | Zm00014a032370 |
| *ZmCCT31* | Zm00001d015468 |  | *ZmCCT31* | Zm00004b014341 |  | *ZmCCT31* | Zm00014a032640 |
| *ZmCCT32* | Zm00001d017176 |  | *ZmCCT32* | Zm00004b014391 |  | *ZmCCT32* | Zm00014a034653 |
| *ZmCCT33* | Zm00001d017241 |  | *ZmCCT33* | Zm00004b017004 |  | *ZmCCT33* | Zm00014a035581 |
| *ZmCCT34* | Zm00001d017885 |  | *ZmCCT34* | Zm00004b017830 |  | *ZmCCT34* | Zm00014a036096 |
| *ZmCCT35* | Zm00001d017939 |  | *ZmCCT35* | Zm00004b017836 |  | *ZmCCT35* | Zm00014a037276 |
| *ZmCCT36* | Zm00001d035134 |  | *ZmCCT36* | Zm00004b019262 |  | *ZmCCT36* | Zm00014a037753 |
| *ZmCCT37* | Zm00001d036494 |  | *ZmCCT37* | Zm00004b020068 |  | *ZmCCT37* | Zm00014a040386 |
| *ZmCCT38* | Zm00001d037327 |  | *ZmCCT38* | Zm00004b020696 |  | *ZmCCT38* | Zm00014a040840 |
| *ZmCCT39* | Zm00001d038407 |  | *ZmCCT39* | Zm00004b021513 |  | *ZmCCT39* | Zm00014a040841 |
| *ZmCCT40* | Zm00001d039222 |  | *ZmCCT40* | Zm00004b022009 |  | *ZmCCT40* | Zm00014a040842 |
| *ZmCCT41* | Zm00001d021291 |  | *ZmCCT41* | Zm00004b022058 |  | *ZmCCT41* | Zm00014a040924 |
| *ZmCCT42* | Zm00001d022500 |  | *ZmCCT42* | Zm00004b024374 |  | *ZmCCT42* | Zm00014a041970 |
| *ZmCCT43* | Zm00001d022590 |  | *ZmCCT43* | Zm00004b024970 |  | *ZmCCT43* | Zm00014a042358 |
| *ZmCCT44* | Zm00001d008886 |  | *ZmCCT44* | Zm00004b025536 |  | *ZmCCT44* | Zm00014a042860 |
| *ZmCCT45* | Zm00001d009773 |  | *ZmCCT45* | Zm00004b029303 |  | *ZmCCT45* | Zm00014a043267 |
| *ZmCCT46* | Zm00001d012441 |  | *ZmCCT46* | Zm00004b029944 |  | *ZmCCT46* | Zm00014a043608 |
| *ZmCCT47* | Zm00001d012445 |  | *ZmCCT47* | Zm00004b032201 |  | *ZmCCT47* | Zm00014a044239 |
| *ZmCCT48* | Zm00001d045636 |  | *ZmCCT48* | Zm00004b032693 |  | *ZmCCT48* | Zm00014a044552 |
| *ZmCCT49* | Zm00001d045661 |  | *ZmCCT49* | Zm00004b033157 |  |  |  |
| *ZmCCT50* | Zm00001d045735 |  | *ZmCCT50* | Zm00004b034344 |  |  |  |
| *ZmCCT51* | Zm00001d045804 |  | *ZmCCT51* | Zm00004b034345 |  |  |  |
| *ZmCCT52* | Zm00001d046925 |  | *ZmCCT52* | Zm00004b034799 |  |  |  |
| *ZmCCT53* | Zm00001d047761 |  | *ZmCCT53* | Zm00004b035920 |  |  |  |
| *ZmCCT54* | Zm00001d048369 |  | *ZmCCT54* | Zm00004b036592 |  |  |  |
| *ZmCCT55* | Zm00001d024200 |  | *ZmCCT55* | Zm00004b036609 |  |  |  |
| *ZmCCT56* | Zm00001d024909 |  | *ZmCCT56* | Zm00004b037309 |  |  |  |
| *ZmCCT57* | Zm00001d025770 |  | *ZmCCT57* | Zm00004b038721 |  |  |  |
| *ZmCCT58* | Zm00001d000176 |  | *ZmCCT58* | Zm00004b039816 |  |  |  |
|  |  |  | *ZmCCT59* | Zm00004b040435 |  |  |  |

**Table S3 *Continued***

| **Huangzao4** | |  | **SK** | |
| --- | --- | --- | --- | --- |
| ***ZmCCT*** | **Gene ID** |  | ***ZmCCT*** | **Gene ID** |
| *ZmCCT1* | Chr1_pilon_17820.7_495 |  | *ZmCCT1* | Zm00015a000420 |
| *ZmCCT2* | Chr1_pilon_17840_1874 |  | *ZmCCT2* | Zm00015a000571 |
| *ZmCCT3* | Chr1_pilon_17846.8_549 |  | *ZmCCT3* | Zm00015a000573 |
| *ZmCCT4* | Chr1_pilon_245690.9_2711 |  | *ZmCCT4* | Zm00015a000574 |
| *ZmCCT5* | Chr1_pilon_245714.4_15328 |  | *ZmCCT5* | Zm00015a001821 |
| *ZmCCT6* | Chr1_pilon_247868.7_12189 |  | *ZmCCT6* | Zm00015a004744 |
| *ZmCCT7* | Chr1_pilon_261738.8_5328 |  | *ZmCCT7* | Zm00015a004745 |
| *ZmCCT8* | Chr1_pilon_268637_4464 |  | *ZmCCT8* | Zm00015a004746 |
| *ZmCCT9* | Chr1_pilon_70481.2_2473 |  | *ZmCCT9* | Zm00015a005156 |
| *ZmCCT10* | Chr10_pilon_130041.3_1355 |  | *ZmCCT10* | Zm00015a005313 |
| *ZmCCT11* | Chr10_pilon_145549.8_17911 |  | *ZmCCT11* | Zm00015a006542 |
| *ZmCCT12* | Chr2_pilon_102805.2_351 |  | *ZmCCT12* | Zm00015a006600 |
| *ZmCCT13* | Chr2_pilon_146201.7_5215 |  | *ZmCCT13* | Zm00015a006859 |
| *ZmCCT14* | Chr2_pilon_185245.5_2814 |  | *ZmCCT14* | Zm00015a007481 |
| *ZmCCT15* | Chr2_pilon_189594_19286 |  | *ZmCCT15* | Zm00015a007773 |
| *ZmCCT16* | Chr2_pilon_201392.2_5897 |  | *ZmCCT16* | Zm00015a008115 |
| *ZmCCT17* | Chr2_pilon_4830.8_3794 |  | *ZmCCT17* | Zm00015a008330 |
| *ZmCCT18* | Chr2_pilon_6139.2_11571 |  | *ZmCCT18* | Zm00015a008423 |
| *ZmCCT19* | Chr2_pilon_67674.6_2867 |  | *ZmCCT19* | Zm00015a008877 |
| *ZmCCT20* | Chr2_pilon_90386.1_1021 |  | *ZmCCT20* | Zm00015a009323 |
| *ZmCCT21* | Chr3_pilon_110296.7_621 |  | *ZmCCT21* | Zm00015a009497 |
| *ZmCCT22* | Chr3_pilon_242228.1_104594 |  | *ZmCCT22* | Zm00015a009540 |
| *ZmCCT23* | Chr4_pilon_139681.7_1341 |  | *ZmCCT23* | Zm00015a009624 |
| *ZmCCT24* | Chr4_pilon_196415.5_6031 |  | *ZmCCT24* | Zm00015a009965 |
| *ZmCCT25* | Chr4_pilon_239603.9_3457 |  | *ZmCCT25* | Zm00015a010513 |
| *ZmCCT26* | Chr4_pilon_25613.6_8151 |  | *ZmCCT26* | Zm00015a013037 |
| *ZmCCT27* | Chr4_pilon_83808.6_2576 |  | *ZmCCT27* | Zm00015a015263 |
| *ZmCCT28* | Chr5_pilon_11349.6_1760 |  | *ZmCCT28* | Zm00015a016575 |
| *ZmCCT29* | Chr5_pilon_154462.6_684 |  | *ZmCCT29* | Zm00015a017432 |
| *ZmCCT30* | Chr5_pilon_162518.5_74971 |  | *ZmCCT30* | Zm00015a017928 |
| *ZmCCT31* | Chr5_pilon_187712.4_1416 |  | *ZmCCT31* | Zm00015a017990 |
| *ZmCCT32* | Chr5_pilon_189725.9_2925 |  | *ZmCCT32* | Zm00015a018038 |
| *ZmCCT33* | Chr5_pilon_38396.5_51582 |  | *ZmCCT33* | Zm00015a019477 |
| *ZmCCT34* | Chr5_pilon_38465.2_1282 |  | *ZmCCT34* | Zm00015a020365 |
| *ZmCCT35* | Chr5_pilon_38597.3_483 |  | *ZmCCT35* | Zm00015a021023 |
| *ZmCCT36* | Chr5_pilon_47200.4_10111 |  | *ZmCCT36* | Zm00015a021115 |
| *ZmCCT37* | Chr5_pilon_57432.9_7523 |  | *ZmCCT37* | Zm00015a021492 |
| *ZmCCT38* | Chr5_pilon_69856.1_4273 |  | *ZmCCT38* | Zm00015a021814 |
| *ZmCCT39* | Chr5_pilon_81275.5_3917 |  | *ZmCCT39* | Zm00015a021815 |
| *ZmCCT40* | Chr5_pilon_8624.9_8514 |  | *ZmCCT40* | Zm00015a021816 |
| *ZmCCT41* | Chr5_pilon_91361.4_9919 |  | *ZmCCT41* | Zm00015a021945 |
| *ZmCCT42* | Chr6_pilon_114580.9_2423 |  | *ZmCCT42* | Zm00015a022165 |
| *ZmCCT43* | Chr6_pilon_13009.2_13717 |  | *ZmCCT43* | Zm00015a022431 |
| *ZmCCT44* | Chr6_pilon_85228.8_4693 |  | *ZmCCT44* | Zm00015a022668 |
| *ZmCCT45* | Chr7_pilon_112895.2_2867 |  | *ZmCCT45* | Zm00015a022848 |
| *ZmCCT46* | Chr7_pilon_125937.4_2072 |  | *ZmCCT46* | Zm00015a024208 |
| *ZmCCT47* | Chr7_pilon_148568.7_2770 |  | *ZmCCT47* | Zm00015a024265 |
| *ZmCCT48* | Chr7_pilon_148948.9_5406 |  | *ZmCCT48* | Zm00015a024864 |
| *ZmCCT49* | Chr7_pilon_172969.8_2326 |  | *ZmCCT49* | Zm00015a026594 |
| *ZmCCT50* | Chr7_pilon_6587.9_2759 |  | *ZmCCT50* | Zm00015a027209 |
| *ZmCCT51* | Chr9_pilon_115410.6_1717 |  | *ZmCCT51* | Zm00015a027301 |
| *ZmCCT52* | Chr9_pilon_124315.8_25942 |  | *ZmCCT52* | Zm00015a029178 |
| *ZmCCT53* | Chr9_pilon_146391.2_8965 |  | *ZmCCT53* | Zm00015a029923 |
| *ZmCCT54* | Chr9_pilon_158893.9_78636 |  | *ZmCCT54* | Zm00015a030497 |
| *ZmCCT55* | Chr9_pilon_158905.1_32684 |  | *ZmCCT55* | Zm00015a030702 |
| *ZmCCT56* | Chr9_pilon_31946.4_1312 |  | *ZmCCT56* | Zm00015a031261 |
| *ZmCCT57* | Chr9_pilon_90169.6_42380 |  | *ZmCCT57* | Zm00015a031835 |
|  |  |  | *ZmCCT58* | Zm00015a032003 |
|  |  |  | *ZmCCT59* | Zm00015a037048 |
|  |  |  | *ZmCCT60* | Zm00015a038022 |
|  |  |  | *ZmCCT61* | Zm00015a038486 |
|  |  |  | *ZmCCT62* | Zm00015a038796 |
|  |  |  | *ZmCCT63* | Zm00015a039252 |
|  |  |  | *ZmCCT64* | Zm00015a039253 |
|  |  |  | *ZmCCT65* | Zm00015a040544 |
|  |  |  | *ZmCCT66* | Zm00015a041644 |
|  |  |  | *ZmCCT67* | Zm00015a042245 |
|  |  |  | *ZmCCT68* | Zm00015a042451 |
