# Supplementary material for: Analyses of open-access multi-omics data sets reveal genetic and expression characteristics of maize ZmCCT family genes
Source: AoB Plants. 2021 Aug 16;13(5):plab048. doi: 10.1093/aobpla/plab048 (PMC8459886; doi:10.1093/aobpla/plab048)
Supplement: plab048_suppl_Supplementary_Table_S4 [file plab048_suppl_supplementary_table_s4.docx]

**Table S4** *ZmCCT*s and ZmCCTs in maize inbred line B73

*Note:* The 53 *ZmCCT*s were previously reported by Jin et al. (2018) which was based on v2 B73 genome. Jin et al. (2018) *ZmCOL3*, a CCT gene represses flowering in maize by interfering with the circadian clock and activating expression of *ZmCCT*. J Integr Plant Biol 60:465-480.

**-:** Not reported before; GRAVY: grand average of hydropathy. ZmCCT, Maize CCT domain-containing protein; *ZmCCT*, ZmCCT gene.

|  | | | **Gene: *ZmCCT*** | | | |  | **Protein: ZmCCT** | | | | | | |
| --- | --- | --- | --- | --- | --- | --- | --- | --- | --- | --- | --- | --- | --- | --- |
| **Name** | **Name in MaizeGDB database）** | **Gene ID**  **(v4 B73 genome)** | | **Transcript ID**  **(v4 B73 genome)** | **Intron number (v4 B73 genome)** | **ID (v3 B73 genome) of 53 *ZmCCT*s corresponding to the previous report（Jin et al. 2018）** |  | **ID**  **(v4 B73 genome)** | **Length (amino acid number)** | **Molecular weight (Dalton)** | **pI** | **Subcellular localization** | **Overall average of hydropathicity** | **Conserved domain** |
| ***ZmCCT1*** | - | Zm00001d027598 | | Zm00001d027598_T001 | 3 | GRMZM2G474258 |  | Zm00001d027598_P001 | 780 | 28957.5 | 5.25 | Nuclear | -0.667568 | CCT |
|  |  |  |  | Zm00001d027598_T002 | 3 |  |  | Zm00001d027598_P002 | 753 | 27874.2 | 5.13 | Nuclear | -0.6756 | CCT |
| ***ZmCCT2*** | col15 | Zm00001d029149 | | Zm00001d029149_T001 | 3 | GRMZM2G134671 |  | Zm00001d029149_P001 | 1194 | 42767.3 | 5.26 | Nuclear | -0.673552 | CCT |
| ***ZmCCT3*** | - | Zm00001d029885 | | Zm00001d029885_T001 | 1 | GRMZM2G154580 |  | Zm00001d029885_P001 | 852 | 29264.5 | 6.55 | Nuclear | -0.39258 | CCT |
| ***ZmCCT4*** | - | Zm00001d030229 | | Zm00001d030229_T001 | 1 | GRMZM2G097454 |  | Zm00001d030229_P001 | 789 | 28519.5 | 5.56 | Nuclear | -0.494656 | CCT |
| ***ZmCCT5*** | col16 | Zm00001d031662 | | Zm00001d031662_T001 | 3 | GRMZM2G159996 |  | Zm00001d031662_P001 | 1470 | 51380.3 | 5.88 | Nuclear | -0.395501 | CCT |
| ***ZmCCT6*** | - | Zm00001d032768 | | Zm00001d032768_T001 | 5 | GRMZM2G133555 |  | Zm00001d032768_P001 | 609 | 22154.1 | 6.91 | Nuclear | -0.538614 | CCT |
|  |  |  |  | Zm00001d032768_T002 | 4 |  |  | Zm00001d032768_P002 | 384 | 14743.7 | 9.67 | Nuclear | -0.977953 | CCT |
|  |  |  |  | Zm00001d032768_T003 | 5 |  |  | Zm00001d032768_P003 | 609 | 22154.1 | 6.91 | Nuclear | -0.538614 | CCT |
|  |  |  |  | Zm00001d032768_T004 | 4 |  |  | Zm00001d032768_P004 | 1251 | 44070.3 | 4.59 | Nuclear | -0.302404 | CCT |
|  |  |  |  | Zm00001d032768_T005 | 5 |  |  | Zm00001d032768_P005 | 1272 | 44850.1 | 4.6 | Nuclear | -0.31182 | CCT |
|  |  |  |  | Zm00001d032768_T006 | 5 |  |  | Zm00001d032768_P006 | 1269 | 44811.3 | 4.64 | Nuclear | -0.267536 | CCT |
| ***ZmCCT7*** | - | Zm00001d033523 | | Zm00001d033523_T001 | 7 | - |  | Zm00001d033523_P001 | 867 | 30654.9 | 4.61 | Nuclear | -0.731944 | CCT,  GATA |
| ***ZmCCT8*** | col2 | Zm00001d033719 | | Zm00001d033719_T001 | 2 | GRMZM2G144346 |  | Zm00001d033719_P001 | 1413 | 51868.4 | 5.96 | Nuclear | -0.661571 | CCT,  zf-B |
| ***ZmCCT9*** | col11 | Zm00001d003162 | | Zm00001d003162_T001 | 1 | GRMZM2G095598 |  | Zm00001d003162_P001 | 963 | 34070 | 5.43 | Nuclear | -0.382188 | CCT,  zf-B |
| ***ZmCCT10*** | - | Zm00001d003571 | | Zm00001d003571_T001 | 2 | GRMZM2G106108 |  | Zm00001d003571_P001 | 774 | 28910.6 | 8.99 | Nuclear | -0.319066 | CCT |
| ***ZmCCT11*** | TIDP3698 | Zm00001d004875 | | Zm00001d004875_T001 | 2 | GRMZM2G135446 |  | Zm00001d004875_P001 | 717 | 26171.1 | 8.76 | Nuclear | -0.893277 | CCT |
|  |  |  |  | Zm00001d004875_T002 | 7 |  |  | Zm00001d004875_P002 | 2088 | 75610.2 | 8.48 | Nuclear | -0.733525 | CCT,  Response_reg |
|  |  |  |  | Zm00001d004875_T004 | 3 |  |  | Zm00001d004875_P004 | 1377 | 50349.8 | 8.82 | Nuclear | -0.922271 | CCT |
|  |  |  |  | Zm00001d004875_T006 | 6 |  |  | Zm00001d004875_P006 | 1209 | 44501.4 | 8.81 | Nuclear | -0.929602 | CCT |
|  |  |  |  | Zm00001d004875_T007 | 6 |  |  | Zm00001d004875_P007 | 1209 | 44501.4 | 8.81 | Nuclear | -0.929602 | CCT |
|  |  |  |  | Zm00001d004875_T008 | 3 |  |  | Zm00001d004875_P008 | 1209 | 44501.4 | 8.81 | Nuclear | -0.929602 | CCT |
|  |  |  |  | Zm00001d004875_T009 | 4 |  |  | Zm00001d004875_P009 | 1209 | 44501.4 | 8.81 | Nuclear | -0.929602 | CCT |
| ***ZmCCT12*** | prrh1 | Zm00001d006212 | | Zm00001d006212_T001 | 8 | GRMZM2G367834 |  | Zm00001d006212_P001 | 1896 | 70722.9 | 5.84 | Nuclear | -0.820127 | CCT,  Response_reg |
|  |  |  |  | Zm00001d006212_T002 | 8 |  |  | Zm00001d006212_P002 | 1899 | 70794 | 5.84 | Nuclear | -0.815981 | CCT,  Response_reg |
|  |  |  |  | Zm00001d006212_T003 | 7 |  |  | Zm00001d006212_P003 | 1896 | 70722.9 | 5.84 | Nuclear | -0.820127 | CCT,  Response_reg |
|  |  |  |  | Zm00001d006212_T004 | 8 |  |  | Zm00001d006212_P004 | 1785 | 66975 | 6.78 | Nuclear | -0.81229 | CCT,  Response_reg |
| ***ZmCCT13*** | bbx7 | Zm00001d007107 | | Zm00001d007107_T001 | 8 | GRMZM5G813532 |  | Zm00001d007107_P001 | 1230 | 43499.4 | 4.9 | Cytoplasmic | -0.41687 | CCT |
| ***ZmCCT14*** | - | Zm00001d007240 | | Zm00001d007240_T001 | 6 | GRMZM2G033962 |  | Zm00001d007240_P001 | 1779 | 63493 | 9.27 | Nuclear | -0.932939 | CCT,  Response_reg |
| ***ZmCCT15*** | - | Zm00001d042958 | | Zm00001d042958_T001 | 2 | GRMZM2G062218 |  | Zm00001d042958_P001 | 987 | 35255.4 | 6.25 | Nuclear | -0.67378 | CCT |
| ***ZmCCT16*** | - | Zm00001d043783 | | Zm00001d043783_T001 | 1 | AC233946.1 |  | Zm00001d043783_P001 | 639 | 23267.2 | 5.51 | Nuclear | -0.600472 | CCT |
| ***ZmCCT17*** | - | Zm00001d044598 | | Zm00001d044598_T001 | 1 | GRMZM2G001447 |  | Zm00001d044598_P001 | 687 | 24704.5 | 6.06 | Nuclear | -0.386842 | CCT |
| ***ZmCCT18*** | col17 | Zm00001d049347 | | Zm00001d049347_T001 | 0 | GRMZM2G176173 |  | Zm00001d049347_P001 | 840 | 28455.4 | 5.73 | Nuclear | -0.145878 | CCT |
| ***ZmCCT19*** | - | Zm00001d049651 | | Zm00001d049651_T001 | 1 | GRMZM2G008482 |  | Zm00001d049651_P001 | 816 | 29447 | 10.31 | Nuclear | -0.769742 | CCT |
| ***ZmCCT20*** | bbx11 | Zm00001d051047 | | Zm00001d051047_T001 | 1 | - |  | Zm00001d051047_P001 | 945 | 33284 | 5.41 | Nuclear | -0.382166 | CCT,zf-B |
| ***ZmCCT21*** | toc1 | Zm00001d051114 | | Zm00001d051114_T001 | 6 | GRMZM2G020081 |  | Zm00001d051114_P001 | 1641 | 60864.4 | 6.45 | Nuclear | -0.592674 | CCT,  Response_reg |
|  |  |  |  | Zm00001d051114_T002 | 3 |  |  | Zm00001d051114_P002 | 897 | 33045.6 | 6.72 | Nuclear | -0.782215 | CCT |
|  |  |  |  | Zm00001d051114_T003 | 2 |  |  | Zm00001d051114_P003 | 618 | 23148.8 | 6.44 | Nuclear | -0.807317 | CCT |
|  |  |  |  | Zm00001d051114_T004 | 4 |  |  | Zm00001d051114_P004 | 897 | 33045.6 | 6.72 | Nuclear | -0.782215 | CCT |
|  |  |  |  | Zm00001d051114_T005 | 5 |  |  | Zm00001d051114_P005 | 1236 | 46024 | 6.05 | Nuclear | -0.817762 | CCT |
|  |  |  |  | Zm00001d051114_T006 | 3 |  |  | Zm00001d051114_P006 | 618 | 23148.8 | 6.44 | Nuclear | -0.807317 | CCT |
| ***ZmCCT22*** | col14/col9 | Zm00001d051684 | | Zm00001d051684_T001 | 5 | GRMZM2G042198 |  | Zm00001d051684_P001 | 1221 | 44509.9 | 5.3 | Nuclear | -0.519704 | CCT,  zf-B |
|  |  |  |  | Zm00001d051684_T002 | 4 |  |  | Zm00001d051684_P002 | 1221 | 44509.9 | 5.3 | Nuclear | -0.519704 | CCT,  zf-B |
|  |  |  |  | Zm00001d051684_T004 | 5 |  |  | Zm00001d051684_P004 | 1221 | 44509.9 | 5.3 | Nuclear | -0.519704 | CCT,  zf-B |
|  |  |  |  | Zm00001d051684_T007 | 3 |  |  | Zm00001d051684_P007 | 1221 | 44509.9 | 5.3 | Nuclear | -0.519704 | CCT,  zf-B |
| ***ZmCCT23*** | - | Zm00001d052781 | | Zm00001d052781_T002 | 6 | GRMZM2G488465 |  | Zm00001d052781_P002 | 1692 | 61468.1 | 8.56 | Nuclear | -0.81048 | CCT |
|  |  |  |  | Zm00001d052781_T003 | 4 |  |  | Zm00001d052781_P003 | 633 | 23221.1 | 9.03 | Nuclear | -0.773333 | CCT |
|  |  |  |  | Zm00001d052781_T005 | 7 |  |  | Zm00001d052781_P005 | 2085 | 75101.8 | 8.47 | Nuclear | -0.630259 | CCT,  Response_reg |
|  |  |  |  | Zm00001d052781_T006 | 8 |  |  | Zm00001d052781_P006 | 1203 | 43715.6 | 8.6 | Nuclear | -0.80825 | CCT |
|  |  |  |  | Zm00001d052781_T008 | 5 |  |  | Zm00001d052781_P008 | 1203 | 43715.6 | 8.6 | Nuclear | -0.80825 | CCT |
|  |  |  |  | Zm00001d052781_T009 | 4 |  |  | Zm00001d052781_P009 | 1203 | 43715.6 | 8.6 | Nuclear | -0.80825 | CCT |
|  |  |  |  | Zm00001d052781_T010 | 2 |  |  | Zm00001d052781_P010 | 633 | 23221.1 | 9.03 | Nuclear | -0.773333 | CCT |
|  |  |  |  | Zm00001d052781_T011 | 4 |  |  | Zm00001d052781_P011 | 1203 | 43715.6 | 8.6 | Nuclear | -0.80825 | CCT |
| ***ZmCCT24*** | IDP1459 | Zm00001d053880 | | Zm00001d053880_T001 | 1 | GRMZM2G155370 |  | Zm00001d053880_P001 | 1422 | 49309.5 | 8.49 | Nuclear | -0.41797 | CCT |
| ***ZmCCT25*** | zim2 | Zm00001d013331 | | Zm00001d013331_T001 | 7 | GRMZM2G065896 |  | Zm00001d013331_P001 | 837 | 29770.8 | 8.6 | Nuclear | -0.453237 | CCT,  GATA,  tify |
|  |  |  |  | Zm00001d013331_T002 | 7 |  |  | Zm00001d013331_P002 | 711 | 26006.6 | 9.13 | Nuclear | -0.634746 | CCT,  GATA,  tify |
| ***ZmCCT26*** | col8 | Zm00001d013443 | | Zm00001d013443_T001 | 1 | GRMZM2G114137 |  | Zm00001d013443_P001 | 1242 | 45368.2 | 6.68 | Nuclear | -0.622518 | CCT,  zf-B |
| ***ZmCCT27*** | - | Zm00001d014074 | | Zm00001d014074_T001 | 4 | GRMZM2G123550 |  | Zm00001d014074_P001 | 1287 | 45304 | 4.43 | Nuclear | -0.353972 | CCT |
| ***ZmCCT28*** | zim36 | Zm00001d014656 | | Zm00001d014656_T001 | 7 | GRMZM2G058479 |  | Zm00001d014656_P001 | 882 | 30872.8 | 9.23 | Nuclear | -0.461775 | CCT,  GATA,  tify |
|  |  |  |  | Zm00001d014656_T002 | 7 |  |  | Zm00001d014656_P002 | 1074 | 38002 | 5.17 | Nuclear | -0.666667 | CCT,  GATA,t  ify |
|  |  |  |  | Zm00001d014656_T003 | 8 |  |  | Zm00001d014656_P003 | 1077 | 38130.1 | 5.17 | Nuclear | -0.674581 | CCT,  GATA,  tify |
|  |  |  |  | Zm00001d014656_T004 | 7 |  |  | Zm00001d014656_P004 | 828 | 29063.5 | 6.15 | Nuclear | -0.500364 | CCT,  GATA,t  ify |
|  |  |  |  | Zm00001d014656_T005 | 8 |  |  | Zm00001d014656_P005 | 861 | 30374.3 | 9.36 | Nuclear | -0.517133 | CCT,  GATA,  tify |
|  |  |  |  | Zm00001d014656_T006 | 6 |  |  | Zm00001d014656_P006 | 1074 | 38002 | 5.17 | Nuclear | -0.666667 | CCT,  GATA,  tify |
|  |  |  |  | Zm00001d014656_T007 | 7 |  |  | Zm00001d014656_P007 | 876 | 30730.7 | 9.23 | Nuclear | -0.47732 | CCT,  GATA,  tify |
|  |  |  |  | Zm00001d014656_T008 | 6 |  |  | Zm00001d014656_P008 | 948 | 34420.5 | 5.58 | Nuclear | -0.640635 | CCT,  GATA,  tify |
| ***ZmCCT29*** | - | Zm00001d014963 | | Zm00001d014963_T001 | 2 | AC215811.3 |  | Zm00001d014963_P001 | 786 | 28672.8 | 8.97 | Nuclear | -0.812261 | CCT |
| ***ZmCCT30*** | - | Zm00001d015268 | | Zm00001d015268_T001 | 2 | GRMZM2G062885 |  | Zm00001d015268_P001 | 1419 | 49623.9 | 6.58 | Chloroplast | -0.429449 | CCT |
| ***ZmCCT31*** | col18 | Zm00001d015468 | | Zm00001d015468_T001 | 2 | GRMZM2G148772 |  | Zm00001d015468_P001 | 1122 | 38834.5 | 6.09 | Chloroplast | -0.220912 | CCT,  zf-B |
| ***ZmCCT32*** | col3 | Zm00001d017176 | | Zm00001d017176_T001 | 1 | GRMZM2G021777 |  | Zm00001d017176_P001 | 1008 | 35391.5 | 5.04 | Nuclear | -0.241493 | CCT,  zf-B |
| ***ZmCCT33*** | toc2 | Zm00001d017241 | | Zm00001d017241_T001 | 6 | GRMZM2G148453 |  | Zm00001d017241_P001 | 1554 | 57559.4 | 6.3 | Nuclear | -0.599807 | CCT,  Response_reg |
|  |  |  |  | Zm00001d017241_T002 | 5 |  |  | Zm00001d017241_P002 | 1554 | 57559.4 | 6.3 | Nuclear | -0.599807 | CCT,  Response_reg |
|  |  |  |  | Zm00001d017241_T003 | 4 |  |  | Zm00001d017241_P003 | 837 | 31137.5 | 6.59 | Nuclear | -0.738129 | CCT |
|  |  |  |  | Zm00001d017241_T004 | 3 |  |  | Zm00001d017241_P004 | 837 | 31137.5 | 6.59 | Nuclear | -0.738129 | CCT |
|  |  |  |  | Zm00001d017241_T005 | 5 |  |  | Zm00001d017241_P005 | 1230 | 45842.7 | 5.98 | Nuclear | -0.776039 | CCT |
|  |  |  |  | Zm00001d017241_T006 | 2 |  |  | Zm00001d017241_P006 | 837 | 31137.5 | 6.59 | Nuclear | -0.738129 | CCT |
|  |  |  |  | Zm00001d017241_T007 | 3 |  |  | Zm00001d017241_P007 | 837 | 31137.5 | 6.59 | Nuclear | -0.738129 | CCT |
| ***ZmCCT34*** | col5 | Zm00001d017885 | | Zm00001d017885_T001 | 6 | GRMZM2G075562 |  | Zm00001d017885_P001 | 1386 | 51025.2 | 5.33 | Nuclear | -0.484382 | CCT,  zf-B |
|  |  |  |  | Zm00001d017885_T002 | 5 |  |  | Zm00001d017885_P002 | 1275 | 46550.8 | 5.2 | Nuclear | -0.596698 | CCT,  zf-B |
|  |  |  |  | Zm00001d017885_T003 | 4 |  |  | Zm00001d017885_P003 | 1224 | 44477.3 | 5.23 | Nuclear | -0.629238 | CCT,  zf-B |
|  |  |  |  | Zm00001d017885_T004 | 4 |  |  | Zm00001d017885_P004 | 1224 | 44477.3 | 5.23 | Nuclear | -0.629238 | CCT,  zf-B |
| ***ZmCCT35*** | col12 | Zm00001d017939 | | Zm00001d017939_T001 | 2 | GRMZM2G041991 |  | Zm00001d017939_P001 | 1398 | 50506.7 | 5.84 | Nuclear | -0.575699 | CCT,  zf-B |
| ***ZmCCT36*** | - | Zm00001d035134 | | Zm00001d035134_T001 | 1 | GRMZM2G134066 |  | Zm00001d035134_P001 | 804 | 28974.7 | 9.88 | Nuclear | -0.702996 | CCT |
| ***ZmCCT37*** | zim20 | Zm00001d036494 | | Zm00001d036494_T001 | 6 | GRMZM2G080509 |  | Zm00001d036494_P001 | 1077 | 38126.4 | 5.09 | Nuclear | -0.567877 | CCT,  GATA,  tify |
|  |  |  |  | Zm00001d036494_T002 | 7 |  |  | Zm00001d036494_P002 | 876 | 30897.1 | 9.25 | Nuclear | -0.409966 | CCT,  GATA,  tify |
|  |  |  |  | Zm00001d036494_T003 | 7 |  |  | Zm00001d036494_P003 | 870 | 30696.9 | 9.39 | Nuclear | -0.40692 | CCT,  GATA,  tify |
|  |  |  |  | Zm00001d036494_T004 | 6 |  |  | Zm00001d036494_P004 | 1074 | 37998.3 | 5.09 | Nuclear | -0.559664 | CCT,  GATA,  tify |
|  |  |  |  | Zm00001d036494_T005 | 6 |  |  | Zm00001d036494_P005 | 825 | 29137.8 | 5.91 | Nuclear | -0.418978 | CCT,  GATA,  tify |
| ***ZmCCT38*** | col10 | Zm00001d037327 | | Zm00001d037327_T001 | 1 | GRMZM2G012717 |  | Zm00001d037327_P001 | 1359 | 48379.9 | 5.24 | Nuclear | -0.475885 | CCT,  zf-B |
| ***ZmCCT39*** | - | Zm00001d038407 | | Zm00001d038407_T001 | 5 | GRMZM2G318992 |  | Zm00001d038407_P001 | 990 | 35854.6 | 5.99 | Nuclear | -0.589058 | CCT |
|  |  |  |  | Zm00001d038407_T002 | 4 |  |  | Zm00001d038407_P002 | 990 | 35854.6 | 5.99 | Nuclear | -0.589058 | CCT |
|  |  |  |  | Zm00001d038407_T003 | 3 |  |  | Zm00001d038407_P003 | 990 | 35854.6 | 5.99 | Nuclear | -0.589058 | CCT |
| ***ZmCCT40*** | - | Zm00001d039222 | | Zm00001d039222_T001 | 3 | GRMZM2G127426 |  | Zm00001d039222_P001 | 795 | 29235.5 | 5.13 | Nuclear | -0.334091 | CCT |
| ***ZmCCT41*** | - | Zm00001d021291 | | Zm00001d021291_T001 | 9 | GRMZM2G179024 |  | Zm00001d021291_P001 | 1926 | 71006.6 | 6 | Nuclear | -0.680811 | CCT,  Response_reg |
|  |  |  |  | Zm00001d021291_T002 | 9 |  |  | Zm00001d021291_P002 | 2040 | 75388.3 | 6.05 | Nuclear | -0.72651 | CCT,  Response_reg |
|  |  |  |  | Zm00001d021291_T003 | 7 |  |  | Zm00001d021291_P003 | 1773 | 65500.3 | 5.73 | Nuclear | -0.710508 | CCT,  Response_reg |
|  |  |  |  | Zm00001d021291_T004 | 9 |  |  | Zm00001d021291_P004 | 1791 | 66777.9 | 6.75 | Nuclear | -0.776174 | CCT,  Response_reg |
|  |  |  |  | Zm00001d021291_T005 | 9 |  |  | Zm00001d021291_P005 | 1887 | 69882.1 | 5.79 | Nuclear | -0.758121 | CCT,  Response_reg |
|  |  |  |  | Zm00001d021291_T006 | 7 |  |  | Zm00001d021291_P006 | 1890 | 70008.3 | 5.86 | Nuclear | -0.760095 | CCT,  Response_reg |
| ***ZmCCT42*** | col20 | Zm00001d022500 | | Zm00001d022500_T001 | 2 | - |  | Zm00001d022500_P001 | 546 | 19517.8 | 5.33 | Cytoplasmic | -0.548619 | CCT |
| ***ZmCCT43*** | - | Zm00001d022590 | | Zm00001d022590_T002 | 9 | GRMZM2G005732 |  | Zm00001d022590_P002 | 1800 | 63870.5 | 6.69 | Nuclear | -0.987145 | CCT |
|  |  |  |  | Zm00001d022590_T003 | 10 |  |  | Zm00001d022590_P003 | 1800 | 63870.5 | 6.69 | Nuclear | -0.987145 | CCT |
|  |  |  |  | Zm00001d022590_T004 | 8 |  |  | Zm00001d022590_P004 | 1734 | 62427.7 | 8.81 | Nuclear | -0.939341 | CCT |
|  |  |  |  | Zm00001d022590_T005 | 9 |  |  | Zm00001d022590_P005 | 1944 | 69185.1 | 6.72 | Nuclear | -1.035085 | CCT |
|  |  |  |  | Zm00001d022590_T006 | 10 |  |  | Zm00001d022590_P006 | 1944 | 69185.1 | 6.72 | Nuclear | -1.035085 | CCT |
|  |  |  |  | Zm00001d022590_T007 | 9 |  |  | Zm00001d022590_P007 | 2025 | 72521.1 | 7.96 | Nuclear | -1.016469 | CCT |
|  |  |  |  | Zm00001d022590_T008 | 8 |  |  | Zm00001d022590_P008 | 1878 | 67742.3 | 8.76 | Nuclear | -0.99264 | CCT |
|  |  |  |  | Zm00001d022590_T009 | 8 |  |  | Zm00001d022590_P009 | 2265 | 82162.7 | 7.26 | Nuclear | -0.859151 | CCT,  Response_reg |
|  |  |  |  | Zm00001d022590_T012 | 5 |  |  | Zm00001d022590_P012 | 1815 | 64515.3 | 8.27 | Nuclear | -1.000497 | CCT |
|  |  |  |  | Zm00001d022590_T014 | 5 |  |  | Zm00001d022590_P014 | 1725 | 61859.6 | 7.66 | Nuclear | -0.989721 | CCT |
|  |  |  |  | Zm00001d022590_T016 | 4 |  |  | Zm00001d022590_P016 | 1683 | 60357.8 | 8.06 | Nuclear | -1.045714 | CCT |
|  |  |  |  | Zm00001d022590_T017 | 4 |  |  | Zm00001d022590_P017 | 924 | 32673.7 | 9.75 | Nuclear | -0.932248 | CCT |
|  |  |  |  | Zm00001d022590_T018 | 4 |  |  | Zm00001d022590_P018 | 1548 | 55342.5 | 8.38 | Nuclear | -1 | CCT |
|  |  |  |  | Zm00001d022590_T019 | 5 |  |  | Zm00001d022590_P019 | 1590 | 56985.2 | 8.38 | Nuclear | -1.039319 | CCT |
| ***ZmCCT44*** | mbd126 | Zm00001d008886 | | Zm00001d008886_T001 | 9 | GRMZM2G161447 |  | Zm00001d008886_P001 | 1182 | 43460.6 | 9.66 | Nuclear | -0.494148 | CCT,  MBD |
| ***ZmCCT45*** | - | Zm00001d009773 | | Zm00001d009773_T001 | 3 | GRMZM2G126026 |  | Zm00001d009773_P001 | 945 | 34614.5 | 4.9 | Nuclear | -0.375796 | CCT |
|  |  |  |  | Zm00001d009773_T002 | 3 |  |  | Zm00001d009773_P002 | 909 | 33116.6 | 4.54 | Nuclear | -0.322517 | CCT |
|  |  |  |  | Zm00001d009773_T003 | 4 |  |  | Zm00001d009773_P003 | 588 | 22062.4 | 8.68 | Nuclear | -0.632308 | CCT |
| ***ZmCCT46*** | - | Zm00001d012441 | | Zm00001d012441_T001 | 2 | - |  | Zm00001d012441_P001 | 912 | 32959.1 | 6.71 | Nuclear | -0.775248 | CCT |
| ***ZmCCT47*** | cadtfr8 | Zm00001d012445 | | Zm00001d012445_T002 | 30 | GRMZM5G866699 |  | Zm00001d012445_P002 | 921 | 33076.1 | 6.6 | Nuclear | -0.75719 | CCT |
| ***ZmCCT48*** | - | Zm00001d045636 | | Zm00001d045636_T001 | 2 | GRMZM2G167216 |  | Zm00001d045636_P001 | 696 | 25581.6 | 8.47 | Extracellular | -0.287879 | CCT |
| ***ZmCCT49*** | col4 | Zm00001d045661 | | Zm00001d045661_T001 | 1 | GRMZM2G107886 |  | Zm00001d045661_P001 | 1371 | 49286.7 | 5.09 | Nuclear | -0.561404 | CCT,  zf-B |
| ***ZmCCT50*** | conz1 | Zm00001d045735 | | Zm00001d045735_T002 | 4 | GRMZM2G405368 |  | Zm00001d045735_P002 | 1287 | 46210.8 | 5.56 | Nuclear | -0.246963 | CCT,  zf-B |
|  |  |  |  | Zm00001d045735_T004 | 1 |  |  | Zm00001d045735_P004 | 522 | 19172.8 | 9.15 | Nuclear | -0.482081 | CCT |
| ***ZmCCT51*** | col6 | Zm00001d045804 | | Zm00001d045804_T003 | 3 | GRMZM2G013398 |  | Zm00001d045804_P003 | 1311 | 47322.1 | 5.69 | Nuclear | -0.337385 | CCT,  zf-B |
|  |  |  |  | Zm00001d045804_T004 | 4 |  |  | Zm00001d045804_P004 | 1227 | 43827.8 | 5.29 | Nuclear | -0.42451 | CCT,  zf-B |
|  |  |  |  | Zm00001d045804_T005 | 4 |  |  | Zm00001d045804_P005 | 1311 | 47322.1 | 5.69 | Nuclear | -0.337385 | CCT,  zf-B |
|  |  |  |  | Zm00001d045804_T006 | 4 |  |  | Zm00001d045804_P006 | 1242 | 44392.5 | 5.21 | Nuclear | -0.427603 | CCT,  zf-B |
|  |  |  |  | Zm00001d045804_T009 | 4 |  |  | Zm00001d045804_P009 | 1224 | 43699.7 | 5.29 | Nuclear | -0.416953 | CCT,  zf-B |
|  |  |  |  | Zm00001d045804_T013 | 4 |  |  | Zm00001d045804_P013 | 1224 | 43699.7 | 5.29 | Nuclear | -0.416953 | CCT,  zf-B |
| ***ZmCCT52*** | col13 | Zm00001d046925 | | Zm00001d046925_T001 | 2 | GRMZM2G038783 |  | Zm00001d046925_P001 | 1095 | 38808.4 | 5.81 | Nuclear | -0.275 | CCT,  zf-B |
| ***ZmCCT53*** | - | Zm00001d047761 | | Zm00001d047761_T001 | 9 | - |  | Zm00001d047761_P001 | 900 | 32176.2 | 9.47 | Nuclear | -0.935117 | CCT |
|  |  |  |  | Zm00001d047761_T005 | 11 |  |  | Zm00001d047761_P005 | 2301 | 83764.1 | 6.12 | Nuclear | -0.887076 | CCT,  Response_reg |
|  |  |  |  | Zm00001d047761_T006 | 10 |  |  | Zm00001d047761_P006 | 1812 | 65589.5 | 6.65 | Nuclear | -1.008789 | CCT |
|  |  |  |  | Zm00001d047761_T008 | 11 |  |  | Zm00001d047761_P008 | 2301 | 83764.1 | 6.12 | Nuclear | -0.887076 | CCT,  Response_reg |
|  |  |  |  | Zm00001d047761_T014 | 7 |  |  | Zm00001d047761_P014 | 1890 | 68370 | 6.93 | Nuclear | -0.933068 | CCT,  Response_reg |
| ***ZmCCT54*** | - | Zm00001d048369 | | Zm00001d048369_T001 | 4 | GRMZM2G057529 |  | Zm00001d048369_P001 | 1161 | 42006.5 | 4.49 | Nuclear | -0.461658 | CCT |
|  |  |  |  | Zm00001d048369_T002 | 4 |  |  | Zm00001d048369_P002 | 1170 | 42239.7 | 4.38 | Nuclear | -0.439075 | CCT |
| ***ZmCCT55*** | col19 | Zm00001d024200 | | Zm00001d024200_T001 | 0 | GRMZM2G092363 |  | Zm00001d024200_P001 | 837 | 28163.2 | 6.08 | Nuclear | -0.02446 | CCT |
| ***ZmCCT56*** | cct1 | Zm00001d024909 | | Zm00001d024909_T001 | 1 | GRMZM2G381691 |  | Zm00001d024909_P001 | 723 | 26212 | 5.83 | Nuclear | -0.692083 | CCT |
| ***ZmCCT57*** | col7 | Zm00001d025770 | | Zm00001d025770_T001 | 1 | AC233888.1 |  | Zm00001d025770_P001 | 972 | 34011.9 | 5.2 | Cytoplasmic | -0.329412 | CCT,  zf-B |
| ***ZmCCT58*** | cct2 | Zm00001d000176 | | Zm00001d000176_T001 | 1 | GRMZM2G004483 |  | Zm00001d000176_P001 | 735 | 25419.2 | 6.83 | Nuclear | -0.452245 | CCT |
