# Supplementary material for: Analyses of open-access multi-omics data sets reveal genetic and expression characteristics of maize ZmCCT family genes
Source: AoB Plants. 2021 Aug 16;13(5):plab048. doi: 10.1093/aobpla/plab048 (PMC8459886; doi:10.1093/aobpla/plab048)
Supplement: plab048_suppl_Supplementary_Table_S5 [file plab048_suppl_supplementary_table_s5.docx]

**Table S5** Conserved domains/motifs of maize ZmCCTs

*Note*: ZmCCT, Maize CCT domain-containing protein; *ZmCCT*, ZmCCT gene.

| Motif |  | E-value | Sites | The number of amino acid residues | Conserved amino acid sequence | Annotation |
| --- | --- | --- | --- | --- | --- | --- |
| Motif1 |  | 4.9e-1066 | 58 | 29 | RKXR[NR]FDKKIRY[AE][SCV]RK[AT][LY]A[DE]SRPRV[KR]G[RQ]F | CCT_domain, IPR010402 |
| Motif2 |  | 5.7e-358 | 21 | 39 | [AR][PR]A[AR][VW][YT]CRAD[AD]A[AF]LC[AQL][AS]CD[AR][DS][VI]HSANPLA[RS]RHER[LV][PR][LV][CA]P | Znf_B-box domain, IPR000315 |
| Motif3 |  | 1.10E-273 | 57 | 21 | XXXX[RA]XX[AG]EERE[AE][RAK][LVI]M[RK]Y[RK]EK | - |
| Motif4 |  | 7.10E-127 | 6 | 49 | [VI]IMMS[SN][HR]D[SE]VS[MTV]V[FV]KC[LM][LR][KL]GA[AV][DE][FY]LVKP[LI]R[KT]NEL[RL]NLW[QT]HVWR[RK][RQ][LR][MA][NL]G | Receiver domain, IPR001789 |
| Motif5 |  | 7.50E-49 | 6 | 41 | [WI][DLN][IL][LP][EKN][EPY][DEK][GHS][GF][ENQ][IK][DE][GIL][AIV][LT][TA]EV[DEF][LF][PA][DLV][SM][DKS][CGP][FY][LK][DLM]L[DKS][FTY][IA][AMR][ERS][HMN][DK][DA][ILS][KR][HNT]I | - |
| Motif6 |  | 4.00E-45 | 7 | 21 | ARAH[GH]A[GR]S[RG]H[AE]RVW[LV]CEVCE[HC] | - |
| Motif7 |  | 7.20E-63 | 6 | 41 | [EL]C[QH][NH]C[GD][ITW][NS][AG][HT]A[TA]P[AMS]MRRG[PH][ADK][GR][PQ]R[TI][LN]C[NY][AS][CG][GC][LP][MS][WS]A[NE][KL][GS][LRT][LI][RW][SD] | GATA domain, IPR000679 |
| Motif8 |  | 7.70E-43 | 9 | 25 | [DEN][FY][ET][AES][LAP][FC][GP][LTV][RS][RH][AIV][FYR][ST][ET][GE][DL][FIL][QD][DGN][ALM][GN][AGI][DNS][ST][PY] | - |
| Motif9 |  | 2.00E-41 | 11 | 21 | [LM]XL[KT]L[DN]Y[ED][EA][IV][IL]A[AS]W[GV][DS]S[GP][GW][MT]G | - |
| Motif10 |  | 1.50E-34 | 5 | 27 | R[PA]CD[AG]C[AGR]A[EAR][PA][AS][RV][LV][YH]C[RH]AD[AGT]A[FY]LC[AP][GS]C[DN] | Znf_B-box domain, IPR000315 |
| Motif11 |  | 7.20E-42 | 3 | 40 | IQND[AP]LLSEVLYEC[EK]KELMEKS[AT]IEETISELMDVKIPMLQ | - |
| Motif12 |  | 9.10E-35 | 3 | 41 | ME[NK]EK[RQ]S[IV]PECSLQKSVSSGCLNSAD[WR]MNG[AP][VA]RPNFLDFQG | - |
| Motif13 |  | 5.00E-27 | 4 | 49 | [EV]G[SV][GS][GKM][EAG][QMT][QGP][FM][LV][DP]R[MS][KP]VR[IV]LL[AC][DE]GD[AD][ST][ST][RS][HR][EV][IV][LS][AR]LL[CR][KN]C[GS]Y[HR]V[AT][AC]A[KS][DS][GP][RV][KQ] | Receiver domain, IPR001789 |
| Motif14 |  | 6.90E-26 | 5 | 26 | [GNS]F[GDH][PV][TP]D[AM][ED]L[RA][ES]FAADME[AS]LL[GM][GHQY][GV][LD]D[DG] | - |
| Motif15 |  | 4.40E-27 | 5 | 29 | [EQG][DEG][DEP][ED][EG][QDEN][LV]LY[RC]VP[VIT][FL]D[PR][AM]LAE[FL][CY][SN]P[PVQ][PA][DLP][EV][DG] | - |
| Motif16 |  | 8.00E-25 | 7 | 29 | N[NP][IG]QR[SA][DV]SS[HL][CS][LI]P[MLV]R[LQ][QV][RLP][SG][SD][VI]S[LH][GS][GL][SR]GL[TY] | - |
| Motif17 |  | 1.90E-23 | 8 | 14 | [TDR][GDF]D[DNG][GQ][EAP][WF]W[WMN][RGW][PTA][PA][VG][AP] | - |
| Motif18 |  | 2.00E-23 | 3 | 39 | RYHTS[AV]ASNQGG[TA][GR][LY][VG][GE]S[CS]SP[HQ]DNSSEA[VM]KTDST[YC][NK]MKS | Tify domain (IPR010399) |
| Motif19 |  | 3.50E-33 | 4 | 49 | [KS][KQ][QR][TEG][CF][FL][NS][SDE][AN][NGS]S[EN][RGS][ALMT][SV][MR][DI][GM]E[LY][QV][HK][IM]M[ED][GN][EQ][GQ][KL][THY][TDN]T[QY][DRW][DE][TV]D[ST]M[RKQ][GT][TA][FS][NR][TGR]N[AD]E[KM] | - |
| Motif20 |  | 2.70E-24 | 2 | 50 | FEMP[AV]QYPMV[CW]FSSS[NS]MHMERS[NS]EGHNDTSGTPPAYHFPFYYPGMVEHNM | - |
