# Supplementary material for: Analyses of open-access multi-omics data sets reveal genetic and expression characteristics of maize ZmCCT family genes
Source: AoB Plants. 2021 Aug 16;13(5):plab048. doi: 10.1093/aobpla/plab048 (PMC8459886; doi:10.1093/aobpla/plab048)
Supplement: plab048_suppl_Supplementary_Table_S6 [file plab048_suppl_supplementary_table_s6.docx]

| **Table S6** Distribution of SVs of *ZmCCT*s in genomes of 521 maize inbred lines  *Note:* AA, the same structure as B73 but different structure from SK; NN, unable to evaluate; SV, Structural variations; TT, the same structure as SK but different structure from B73; ZmCCT, Maize CCT domain-containing protein; *ZmCCT*, ZmCCT gene. | | | | | | | | | | | | | | | | | | | | | | | |
| --- | --- | --- | --- | --- | --- | --- | --- | --- | --- | --- | --- | --- | --- | --- | --- | --- | --- | --- | --- | --- | --- | --- | --- |
|  |  | Maize inbred line | | | | | | | | | | | | | | | | | | | | | |
| *ZmCCT* | SV number | 04K5672 | 04K5686 | 04K5702 | 05W002 | 05WN230 | 07KS4 | 1323 | 1462 | 150 | 177 | 18-599 | 238 | 268 | 303WX | 3411 | 384-2 | 3H-2 | 4019 | 4F1 | 501 | 5237 | 526018 |
| *ZmCCT8* | SV1 | AA | NN | NN | AA | AA | AA | AA | NN | NN | AA | AA | AA | AA | AA | AA | AA | AA | NN | NN | AA | NN | AA |
| *ZmCCT29* | SV2 | NN | NN | NN | NN | NN | NN | NN | NN | NN | NN | NN | NN | NN | NN | NN | NN | NN | NN | NN | NN | NN | NN |
|  | SV3 | TT | AA | NN | TT | TT | TT | TT | NN | NN | TT | TT | AA | TT | AA | TT | TT | TT | NN | NN | NN | NN | TT |
| *ZmCCT30* | SV4 | NN | NN | NN | NN | NN | NN | NN | NN | AA | NN | NN | NN | NN | NN | NN | NN | NN | NN | NN | NN | NN | NN |
|  | SV5 | TT | NN | TT | NN | TT | TT | NN | NN | NN | TT | TT | NN | TT | TT | TT | TT | TT | TT | NN | TT | TT | NN |
| *ZmCCT41* | SV6 | NN | NN | NN | NN | NN | NN | NN | NN | NN | NN | NN | NN | NN | AA | NN | NN | NN | NN | NN | NN | NN | NN |
|  | SV7 | TT | TT | TT | TT | NN | TT | TT | NN | TT | TT | NN | NN | TT | NN | NN | TT | NN | TT | TT | TT | TT | TT |
| *ZmCCT43* | SV8 | NN | NN | NN | NN | NN | NN | NN | NN | NN | NN | NN | NN | NN | NN | AA | NN | NN | NN | NN | NN | NN | NN |
|  | SV9 | TT | TT | TT | TT | TT | TT | TT | TT | TT | TT | TT | TT | TT | TT | NN | TT | TT | TT | TT | TT | TT | TT |
|  | SV10 | TT | TT | TT | TT | TT | TT | TT | TT | TT | TT | TT | TT | TT | TT | NN | TT | TT | TT | TT | TT | TT | TT |
|  | SV11 | TT | TT | TT | TT | TT | TT | NN | TT | TT | TT | TT | TT | TT | TT | NN | TT | TT | TT | TT | TT | TT | TT |
|  | SV12 | TT | TT | TT | TT | TT | TT | TT | TT | TT | TT | TT | TT | TT | TT | NN | TT | TT | TT | TT | TT | TT | TT |
|  | SV13 | TT | TT | NN | TT | TT | TT | NN | TT | NN | TT | TT | NN | TT | TT | NN | TT | TT | TT | NN | TT | TT | TT |
|  | SV14 | TT | TT | NN | TT | TT | TT | NN | TT | NN | TT | TT | NN | TT | TT | AA | TT | NN | TT | NN | TT | TT | TT |
|  | SV15 | TT | TT | NN | TT | TT | TT | NN | TT | NN | TT | TT | NN | TT | TT | NN | TT | NN | TT | NN | TT | TT | TT |

| **Table S6** *Continued*  AA, no difference in this locus when compared to B73 genome, but difference when compared to SK genome; NN, unable to judge; SV, structural variation; TT, no difference in this locus when compared to SK genome but difference when compared to B73 genome. | | | | | | | | | | | | | | | | | | | | | | | |
| --- | --- | --- | --- | --- | --- | --- | --- | --- | --- | --- | --- | --- | --- | --- | --- | --- | --- | --- | --- | --- | --- | --- | --- |
|  |  | Maize inbred line | | | | | | | | | | | | | | | | | | | | | |
| *ZmCCT* | SV | 5311 | 647 | 7327 | 7381 | 7884-4HT | 81162 | 812 | 832 | 835B | 8902 | 9642 | 975-12 | 9782 | A619 | B11 | B110 | B111 | B113 | B114 | B151 | B73 | B77 |
| *ZmCCT8* | SV1 | AA | NN | AA | AA | AA | NN | AA | AA | AA | AA | AA | AA | AA | AA | AA | AA | AA | AA | AA | NN | AA | NN |
| *ZmCCT29* | SV2 | AA | NN | AA | NN | AA | NN | AA | NN | NN | NN | NN | NN | NN | NN | AA | NN | AA | NN | NN | AA | AA | AA |
|  | SV3 | AA | TT | AA | NN | NN | TT | AA | TT | TT | TT | NN | TT | TT | TT | NN | TT | NN | TT | NN | AA | AA | NN |
| *ZmCCT30* | SV4 | NN | AA | AA | NN | NN | NN | AA | NN | NN | NN | AA | NN | NN | NN | NN | NN | NN | NN | NN | NN | NN | NN |
|  | SV5 | NN | NN | NN | TT | TT | TT | NN | TT | TT | TT | TT | TT | NN | NN | TT | TT | TT | TT | TT | TT | NN | NN |
| *ZmCCT41* | SV6 | AA | NN | NN | NN | NN | NN | AA | AA | AA | AA | NN | NN | NN | NN | NN | NN | NN | NN | NN | AA | NN | AA |
|  | SV7 | NN | TT | NN | NN | TT | TT | NN | NN | NN | NN | NN | TT | TT | TT | NN | NN | TT | TT | TT | NN | NN | TT |
| *ZmCCT43* | SV8 | NN | NN | NN | NN | NN | NN | NN | AA | NN | NN | NN | NN | NN | NN | NN | AA | NN | NN | NN | NN | AA | NN |
|  | SV9 | TT | NN | TT | TT | TT | TT | TT | NN | TT | TT | TT | TT | NN | TT | TT | NN | TT | TT | TT | TT | NN | TT |
|  | SV10 | TT | NN | TT | TT | TT | TT | TT | NN | TT | TT | TT | TT | NN | TT | TT | NN | TT | TT | TT | TT | NN | TT |
|  | SV11 | TT | NN | TT | TT | TT | TT | TT | NN | TT | TT | TT | TT | NN | TT | TT | NN | TT | TT | TT | TT | NN | TT |
|  | SV12 | TT | NN | TT | TT | TT | TT | TT | NN | TT | TT | TT | TT | NN | TT | TT | NN | TT | TT | TT | TT | NN | TT |
|  | SV13 | TT | NN | TT | TT | TT | TT | TT | NN | TT | TT | TT | NN | NN | TT | TT | NN | TT | NN | TT | TT | NN | TT |
|  | SV14 | TT | NN | TT | TT | TT | TT | TT | NN | TT | TT | TT | NN | NN | TT | TT | NN | TT | NN | TT | TT | NN | NN |
|  | SV15 | TT | NN | TT | TT | TT | TT | TT | NN | TT | TT | TT | NN | NN | TT | TT | NN | TT | TT | TT | TT | NN | NN |

| **Table S6** *Continued*  AA, no difference in this locus when compared to B73 genome but difference when compared to SK genome; NN, unable to judge; SV, structural variation; TT, no difference in this locus when compared to SK genome but difference when compared to B73 genome. | | | | | | | | | | | | | | | | | | | | | | | |
| --- | --- | --- | --- | --- | --- | --- | --- | --- | --- | --- | --- | --- | --- | --- | --- | --- | --- | --- | --- | --- | --- | --- | --- |
|  |  | Maize inbred line | | | | | | | | | | | | | | | | | | | | | |
| *ZmCCT* | SV | BEM | BGY | BK | BS16 | BT1 | BY4839 | BY4944 | BY4960 | BY804 | BY807 | BY809 | BY813 | BY815 | BY843 | BY855 | BZN | C8605 | CF3 | CHANG3 | CHANG7-2 | CHENG698 | CHUAN48-2 |
| *ZmCCT8* | SV1 | AA | NN | AA | AA | AA | NN | AA | AA | AA | NN | AA | AA | AA | AA | AA | NN | AA | AA | AA | AA | TT | NN |
| *ZmCCT29* | SV2 | NN | AA | NN | NN | AA | NN | NN | NN | NN | NN | NN | NN | NN | NN | NN | NN | NN | AA | NN | AA | AA | NN |
|  | SV3 | TT | NN | TT | NN | AA | TT | TT | TT | TT | TT | TT | TT | TT | NN | NN | TT | TT | AA | NN | NN | AA | TT |
| *ZmCCT30* | SV4 | NN | NN | NN | NN | NN | NN | NN | NN | AA | NN | AA | AA | AA | NN | NN | NN | NN | AA | NN | AA | AA | NN |
|  | SV5 | NN | NN | NN | TT | NN | TT | TT | NN | TT | NN | NN | NN | NN | NN | TT | TT | TT | TT | NN | NN | NN | NN |
| *ZmCCT41* | SV6 | NN | NN | NN | NN | NN | NN | NN | NN | NN | NN | NN | NN | NN | NN | NN | NN | NN | NN | NN | NN | NN | NN |
|  | SV7 | NN | NN | NN | TT | TT | NN | TT | TT | NN | NN | TT | TT | TT | NN | TT | NN | TT | TT | TT | NN | NN | NN |
| *ZmCCT43* | SV8 | NN | NN | NN | NN | NN | NN | NN | NN | NN | NN | NN | NN | NN | NN | NN | NN | AA | AA | NN | NN | NN | NN |
|  | SV9 | TT | TT | TT | TT | TT | TT | TT | TT | TT | NN | TT | TT | TT | TT | TT | TT | NN | TT | TT | TT | TT | TT |
|  | SV10 | TT | TT | TT | TT | TT | TT | TT | TT | TT | NN | TT | TT | TT | TT | TT | TT | NN | TT | TT | TT | TT | TT |
|  | SV11 | TT | TT | TT | TT | TT | TT | TT | TT | TT | NN | TT | TT | TT | TT | TT | TT | NN | NN | TT | NN | TT | TT |
|  | SV12 | TT | TT | TT | TT | TT | TT | TT | TT | TT | NN | TT | TT | TT | TT | TT | TT | NN | NN | TT | TT | TT | TT |
|  | SV13 | NN | NN | TT | TT | TT | TT | NN | TT | NN | NN | TT | TT | TT | TT | TT | TT | NN | NN | TT | TT | NN | TT |
|  | SV14 | TT | NN | NN | TT | TT | TT | TT | NN | TT | NN | NN | TT | TT | TT | TT | TT | TT | NN | NN | TT | TT | NN |
|  | SV15 | TT | NN | NN | TT | TT | TT | TT | NN | TT | NN | NN | TT | TT | TT | TT | TT | TT | NN | NN | TT | TT | NN |

| **Table S6** *Continued*  AA, no difference in this locus when compared to B73 genome but difference in when compared to SK genome; NN, unable to judge ; SV, structural variation; TT, no difference in this locus when compared to SK genome but difference in when compared to B73 genome. | | | | | | | | | | | | | | | | | | | | | | | |
| --- | --- | --- | --- | --- | --- | --- | --- | --- | --- | --- | --- | --- | --- | --- | --- | --- | --- | --- | --- | --- | --- | --- | --- |
|  |  | Maize inbred line | | | | | | | | | | | | | | | | | | | | | |
| *ZmCCT* | SV | CI7 | CIMBL1 | CIMBL10 | CIMBL100 | CIMBL101 | CIMBL102 | CIMBL104 | CIMBL105 | CIMBL106 | CIMBL107 | CIMBL108 | CIMBL109 | CIMBL11 | CIMBL110 | CIMBL111 | CIMBL112 | CIMBL113 | CIMBL114 | CIMBL115 | CIMBL116 | CIMBL117 | CIMBL118 |
| *ZmCCT8* | SV1 | NN | AA | NN | AA | NN | AA | AA | NN | AA | AA | AA | AA | NN | AA | AA | NN | AA | AA | AA | AA | AA | NN |
| *ZmCCT29* | SV2 | NN | NN | NN | NN | AA | AA | AA | NN | NN | NN | NN | NN | NN | NN | NN | NN | AA | NN | NN | AA | NN | NN |
|  | SV3 | TT | TT | TT | NN | NN | NN | NN | NN | NN | NN | NN | NN | NN | NN | TT | TT | NN | AA | NN | NN | TT | TT |
| *ZmCCT30* | SV4 | NN | AA | NN | AA | AA | NN | NN | NN | NN | NN | NN | NN | NN | NN | NN | NN | NN | NN | AA | NN | AA | NN |
|  | SV5 | NN | TT | NN | NN | TT | TT | NN | TT | NN | TT | TT | NN | TT | NN | NN | NN | NN | NN | TT | TT | TT | NN |
| *ZmCCT41* | SV6 | NN | NN | NN | NN | NN | NN | NN | NN | NN | NN | NN | NN | NN | NN | NN | NN | NN | NN | NN | NN | NN | NN |
|  | SV7 | NN | TT | NN | TT | TT | TT | NN | TT | NN | NN | NN | NN | TT | TT | TT | NN | TT | TT | NN | TT | TT | TT |
| *ZmCCT43* | SV8 | NN | NN | NN | NN | NN | NN | NN | NN | NN | NN | NN | NN | NN | NN | NN | NN | NN | NN | NN | NN | NN | NN |
|  | SV9 | TT | TT | NN | TT | TT | TT | TT | TT | TT | TT | TT | TT | TT | NN | TT | TT | TT | TT | TT | TT | TT | TT |
|  | SV10 | TT | TT | NN | TT | TT | TT | TT | TT | TT | TT | TT | TT | TT | NN | TT | TT | TT | TT | TT | TT | TT | TT |
|  | SV11 | TT | TT | NN | TT | TT | TT | TT | TT | TT | NN | TT | TT | TT | NN | TT | TT | TT | TT | TT | TT | TT | TT |
|  | SV12 | TT | TT | NN | TT | TT | TT | TT | TT | TT | TT | TT | TT | TT | NN | TT | TT | TT | TT | TT | TT | TT | TT |
|  | SV13 | TT | TT | NN | NN | TT | TT | TT | TT | NN | NN | NN | TT | TT | NN | TT | TT | NN | TT | TT | TT | TT | TT |
|  | SV14 | TT | TT | TT | NN | NN | TT | TT | TT | TT | NN | NN | NN | TT | TT | NN | TT | TT | NN | TT | TT | TT | TT |
|  | SV15 | TT | TT | TT | NN | NN | TT | TT | TT | TT | AA | NN | NN | TT | TT | NN | TT | TT | NN | TT | TT | TT | TT |

| **Table S6** *Continued*  AA, no difference in this locus when compared to B73 genome but difference in when compared to SK genome; NN, unable to judge ; SV, structural variation; TT, no difference in this locus when compared to SK genome but difference in when compared to B73 genome. | | | | | | | | | | | | | | | | | | | | | | | |
| --- | --- | --- | --- | --- | --- | --- | --- | --- | --- | --- | --- | --- | --- | --- | --- | --- | --- | --- | --- | --- | --- | --- | --- |
|  |  | Maize inbred line | | | | | | | | | | | | | | | | | | | | | |
| *ZmCCT* | SV | CIMBL119 | CIMBL120 | CIMBL121 | CIMBL122 | CIMBL123 | CIMBL124 | CIMBL125 | CIMBL126 | CIMBL127 | CIMBL128 | CIMBL129 | CIMBL13 | CIMBL130 | CIMBL131 | CIMBL132 | CIMBL133 | CIMBL134 | CIMBL135 | CIMBL136 | CIMBL137 | CIMBL138 | CIMBL139 |
| *ZmCCT8* | SV1 | AA | AA | NN | NN | NN | NN | AA | NN | NN | NN | AA | NN | NN | NN | AA | AA | AA | AA | NN | NN | NN | AA |
| *ZmCCT29* | SV2 | NN | NN | AA | NN | NN | NN | AA | NN | NN | NN | NN | NN | NN | NN | NN | NN | NN | NN | NN | NN | NN | NN |
|  | SV3 | NN | NN | NN | NN | NN | NN | NN | NN | NN | TT | TT | TT | NN | NN | NN | TT | NN | NN | TT | NN | AA | NN |
| *ZmCCT30* | SV4 | NN | NN | AA | AA | NN | NN | AA | NN | NN | NN | NN | NN | NN | AA | AA | NN | NN | NN | NN | NN | NN | AA |
|  | SV5 | TT | NN | NN | NN | NN | TT | NN | NN | NN | TT | TT | NN | TT | NN | TT | TT | TT | TT | NN | TT | NN | TT |
| *ZmCCT41* | SV6 | NN | AA | NN | NN | NN | AA | NN | NN | AA | NN | NN | NN | AA | NN | NN | NN | AA | NN | AA | NN | NN | AA |
|  | SV7 | NN | NN | NN | NN | NN | NN | TT | NN | TT | TT | TT | NN | TT | TT | NN | NN | TT | NN | NN | TT | NN | NN |
| *ZmCCT43* | SV8 | NN | NN | NN | NN | NN | NN | NN | NN | NN | NN | NN | AA | NN | NN | NN | NN | NN | NN | NN | NN | NN | NN |
|  | SV9 | TT | TT | TT | TT | TT | NN | TT | TT | TT | TT | TT | NN | TT | TT | TT | TT | TT | TT | TT | TT | TT | TT |
|  | SV10 | TT | TT | TT | TT | TT | TT | TT | TT | TT | TT | TT | NN | TT | TT | TT | TT | TT | TT | TT | TT | TT | TT |
|  | SV11 | TT | TT | NN | TT | NN | NN | TT | TT | TT | TT | TT | NN | TT | TT | TT | NN | TT | TT | TT | TT | TT | TT |
|  | SV12 | NN | TT | NN | TT | TT | TT | TT | TT | TT | TT | TT | NN | TT | TT | TT | NN | TT | TT | TT | TT | TT | TT |
|  | SV13 | TT | TT | NN | TT | TT | NN | NN | NN | TT | TT | TT | NN | TT | TT | TT | TT | TT | TT | NN | TT | TT | TT |
|  | SV14 | TT | TT | TT | NN | TT | TT | NN | NN | NN | TT | TT | TT | NN | TT | TT | TT | TT | TT | TT | NN | TT | TT |
|  | SV15 | TT | TT | TT | NN | TT | NN | NN | TT | NN | TT | TT | TT | NN | TT | TT | TT | NN | TT | TT | NN | TT | TT |

| **Table S6** *Continued*  AA, no difference in this locus when compared to B73 genome but difference in when compared to SK genome; NN, unable to judge ; SV, structural variation; TT, no difference in this locus when compared to SK genome but difference in when compared to B73 genome. | | | | | | | | | | | | | | | | | | | | | | | |
| --- | --- | --- | --- | --- | --- | --- | --- | --- | --- | --- | --- | --- | --- | --- | --- | --- | --- | --- | --- | --- | --- | --- | --- |
|  |  | Maize inbred line | | | | | | | | | | | | | | | | | | | | | |
| *ZmCCT* | SV | CIMBL14 | CIMBL140 | CIMBL141 | CIMBL142 | CIMBL143 | CIMBL144 | CIMBL145 | CIMBL146 | CIMBL147 | CIMBL148 | CIMBL149 | CIMBL15 | CIMBL150 | CIMBL151 | CIMBL152 | CIMBL153 | CIMBL154 | CIMBL155 | CIMBL156 | CIMBL157 | CIMBL16 | CIMBL17 |
| *ZmCCT8* | SV1 | AA | AA | TT | AA | NN | AA | NN | AA | NN | AA | AA | NN | AA | AA | AA | TT | AA | AA | AA | AA | AA | AA |
| *ZmCCT29* | SV2 | NN | NN | NN | NN | NN | NN | AA | NN | NN | NN | NN | NN | NN | NN | NN | NN | NN | NN | NN | NN | NN | NN |
|  | SV3 | NN | NN | NN | AA | NN | TT | AA | NN | AA | NN | NN | TT | NN | NN | NN | TT | NN | TT | TT | TT | NN | TT |
| *ZmCCT30* | SV4 | NN | NN | NN | NN | NN | NN | NN | NN | AA | NN | NN | AA | NN | AA | AA | NN | NN | AA | AA | AA | NN | NN |
|  | SV5 | TT | NN | NN | TT | NN | NN | TT | TT | TT | TT | TT | TT | NN | TT | NN | NN | NN | TT | TT | NN | NN | NN |
| *ZmCCT41* | SV6 | AA | NN | NN | AA | NN | NN | AA | AA | NN | NN | AA | AA | NN | NN | NN | NN | NN | NN | NN | AA | NN | NN |
|  | SV7 | NN | NN | NN | NN | TT | NN | NN | NN | TT | NN | NN | TT | NN | TT | TT | NN | TT | TT | NN | TT | NN | NN |
| *ZmCCT43* | SV8 | NN | NN | NN | AA | AA | AA | AA | NN | NN | NN | NN | NN | NN | NN | NN | NN | NN | NN | NN | NN | NN | NN |
|  | SV9 | TT | TT | TT | NN | TT | TT | NN | TT | TT | TT | TT | TT | TT | TT | TT | TT | TT | TT | TT | TT | TT | TT |
|  | SV10 | TT | TT | TT | NN | TT | NN | NN | TT | TT | TT | TT | TT | TT | TT | TT | TT | TT | TT | TT | TT | TT | TT |
|  | SV11 | TT | TT | TT | NN | TT | NN | NN | TT | TT | TT | TT | TT | TT | TT | TT | TT | TT | TT | TT | NN | TT | TT |
|  | SV12 | TT | TT | TT | NN | TT | NN | NN | TT | TT | TT | TT | TT | TT | TT | TT | TT | TT | TT | TT | NN | TT | TT |
|  | SV13 | NN | TT | TT | NN | TT | NN | NN | TT | NN | TT | TT | TT | TT | TT | TT | NN | TT | TT | TT | NN | TT | TT |
|  | SV14 | TT | NN | TT | TT | NN | TT | NN | NN | TT | NN | TT | TT | TT | TT | TT | TT | NN | TT | TT | TT | NN | TT |
|  | SV15 | TT | TT | TT | TT | NN | TT | NN | NN | TT | NN | TT | TT | TT | TT | TT | TT | NN | TT | TT | TT | NN | TT |

| **Table S6** *Continued*  AA, no difference in this locus when compared to B73 genome but difference in when compared to SK genome; NN, unable to judge ; SV, structural variation; TT, no difference in this locus when compared to SK genome but difference in when compared to B73 genome. | | | | | | | | | | | | | | | | | | | | | | | |
| --- | --- | --- | --- | --- | --- | --- | --- | --- | --- | --- | --- | --- | --- | --- | --- | --- | --- | --- | --- | --- | --- | --- | --- |
|  |  | Maize inbred line | | | | | | | | | | | | | | | | | | | | | |
| *ZmCCT8* | SV | CIMBL18 | CIMBL19 | CIMBL2 | CIMBL20 | CIMBL21 | CIMBL22 | CIMBL23 | CIMBL24 | CIMBL25 | CIMBL26 | CIMBL27 | CIMBL28 | CIMBL29 | CIMBL3 | CIMBL30 | CIMBL31 | CIMBL32 | CIMBL33 | CIMBL34 | CIMBL35 | CIMBL36 | CIMBL37 |
| *ZmCCT* | SV1 | AA | AA | AA | AA | TT | AA | AA | AA | NN | NN | NN | NN | NN | AA | AA | AA | AA | AA | AA | AA | NN | AA |
| *ZmCCT29* | SV2 | NN | NN | NN | NN | NN | NN | NN | NN | NN | NN | NN | NN | NN | NN | NN | NN | NN | NN | NN | NN | NN | NN |
|  | SV3 | AA | NN | TT | NN | NN | NN | NN | TT | AA | NN | NN | NN | NN | TT | AA | TT | TT | TT | TT | AA | TT | TT |
| *ZmCCT30* | SV4 | NN | AA | AA | NN | NN | NN | NN | AA | AA | NN | NN | NN | NN | AA | NN | NN | NN | NN | NN | AA | NN | NN |
|  | SV5 | TT | NN | NN | NN | NN | NN | NN | NN | TT | NN | NN | NN | NN | NN | NN | TT | TT | TT | NN | TT | TT | TT |
| *ZmCCT41* | SV6 | AA | NN | NN | AA | NN | AA | AA | NN | NN | NN | NN | NN | NN | NN | NN | NN | NN | NN | NN | NN | NN | NN |
|  | SV7 | NN | TT | NN | TT | TT | NN | TT | NN | TT | NN | NN | NN | TT | NN | TT | NN | TT | NN | NN | TT | TT | NN |
| *ZmCCT43* | SV8 | NN | NN | NN | NN | NN | NN | NN | NN | NN | NN | NN | NN | NN | NN | NN | NN | NN | NN | NN | NN | NN | NN |
|  | SV9 | TT | TT | TT | TT | TT | TT | TT | TT | TT | TT | TT | TT | TT | TT | TT | TT | TT | TT | TT | TT | TT | TT |
|  | SV10 | TT | TT | TT | TT | TT | TT | TT | TT | TT | TT | TT | TT | TT | TT | TT | TT | TT | TT | TT | TT | TT | TT |
|  | SV11 | TT | TT | TT | TT | TT | TT | TT | TT | TT | TT | TT | TT | TT | TT | TT | TT | TT | TT | TT | TT | TT | TT |
|  | SV12 | TT | TT | TT | TT | TT | TT | TT | TT | TT | TT | TT | TT | TT | TT | TT | TT | TT | TT | TT | TT | TT | TT |
|  | SV13 | TT | TT | TT | TT | NN | TT | TT | TT | TT | TT | TT | TT | TT | TT | TT | TT | TT | TT | TT | TT | TT | TT |
|  | SV14 | TT | TT | TT | TT | TT | NN | TT | TT | TT | TT | TT | TT | TT | TT | TT | TT | TT | TT | TT | TT | TT | TT |
|  | SV15 | TT | TT | TT | TT | TT | NN | TT | TT | TT | TT | TT | TT | TT | TT | TT | TT | TT | TT | TT | TT | TT | TT |

| **Table S6** *Continued*  AA, no difference in this locus when compared to B73 genome but difference in when compared to SK genome; NN, unable to judge ; SV, structural variation; TT, no difference in this locus when compared to SK genome but difference in when compared to B73 genome. | | | | | | | | | | | | | | | | | | | | | | | |
| --- | --- | --- | --- | --- | --- | --- | --- | --- | --- | --- | --- | --- | --- | --- | --- | --- | --- | --- | --- | --- | --- | --- | --- |
|  |  | Maize inbred line | | | | | | | | | | | | | | | | | | | | | |
| *ZmCCT* | SV | CIMBL38 | CIMBL39 | CIMBL4 | CIMBL40 | CIMBL41 | CIMBL42 | CIMBL43 | CIMBL44 | CIMBL45 | CIMBL46 | CIMBL47 | CIMBL48 | CIMBL49 | CIMBL5 | CIMBL50 | CIMBL51 | CIMBL52 | CIMBL53 | CIMBL54 | CIMBL56 | CIMBL57 | CIMBL58 |
| *ZmCCT8* | SV1 | AA | AA | NN | NN | AA | NN | AA | AA | AA | NN | NN | AA | AA | AA | AA | AA | AA | AA | AA | TT | AA | AA |
| *ZmCCT29* | SV2 | NN | NN | NN | NN | NN | NN | NN | NN | AA | NN | NN | NN | NN | NN | NN | NN | NN | NN | NN | AA | NN | NN |
|  | SV3 | TT | AA | TT | NN | TT | NN | NN | TT | NN | NN | NN | NN | NN | TT | AA | TT | NN | AA | NN | AA | AA | NN |
| *ZmCCT30* | SV4 | NN | NN | NN | AA | AA | NN | AA | NN | NN | NN | NN | NN | AA | AA | AA | NN | AA | AA | NN | NN | NN | AA |
|  | SV5 | NN | TT | TT | TT | NN | NN | TT | NN | TT | NN | TT | TT | TT | NN | NN | NN | TT | TT | TT | TT | TT | TT |
| *ZmCCT41* | SV6 | AA | NN | AA | NN | AA | NN | NN | NN | NN | NN | NN | AA | AA | NN | AA | NN | NN | NN | NN | NN | NN | NN |
|  | SV7 | TT | NN | TT | NN | NN | NN | NN | NN | NN | NN | NN | NN | TT | NN | TT | NN | TT | NN | TT | TT | TT | TT |
| *ZmCCT43* | SV8 | NN | NN | NN | NN | NN | NN | NN | NN | NN | NN | NN | NN | NN | NN | NN | NN | NN | NN | NN | NN | NN | NN |
|  | SV9 | TT | TT | TT | TT | TT | TT | TT | TT | TT | TT | TT | TT | TT | TT | TT | TT | TT | TT | TT | TT | TT | TT |
|  | SV10 | TT | TT | TT | TT | TT | TT | TT | TT | TT | TT | TT | TT | TT | TT | TT | TT | TT | TT | TT | TT | TT | TT |
|  | SV11 | TT | TT | TT | TT | TT | TT | TT | TT | TT | TT | NN | TT | TT | TT | TT | TT | TT | TT | TT | TT | TT | TT |
|  | SV12 | TT | TT | TT | TT | TT | TT | TT | TT | TT | TT | TT | TT | TT | TT | TT | TT | TT | TT | TT | TT | TT | TT |
|  | SV13 | TT | TT | TT | TT | TT | NN | TT | TT | TT | TT | TT | TT | NN | TT | TT | TT | TT | TT | TT | TT | NN | TT |
|  | SV14 | TT | TT | TT | TT | TT | TT | NN | TT | TT | TT | TT | TT | TT | NN | TT | TT | TT | TT | TT | TT | TT | NN |
|  | SV15 | TT | TT | TT | TT | TT | TT | TT | TT | TT | TT | TT | TT | TT | NN | TT | TT | TT | TT | TT | TT | TT | NN |

| **Table S6** *Continued*  AA, no difference in this locus when compared to B73 genome but difference in when compared to SK genome; NN, unable to judge ; SV, structural variation; TT, no difference in this locus when compared to SK genome but difference in when compared to B73 genome. | | | | | | | | | | | | | | | | | | | | | | | |
| --- | --- | --- | --- | --- | --- | --- | --- | --- | --- | --- | --- | --- | --- | --- | --- | --- | --- | --- | --- | --- | --- | --- | --- |
|  |  | Maize inbred line | | | | | | | | | | | | | | | | | | | | | |
| *ZmCCT* | SV | CIMBL59 | CIMBL6 | CIMBL60 | CIMBL61 | CIMBL62 | CIMBL63 | CIMBL65 | CIMBL66 | CIMBL67 | CIMBL68 | CIMBL69 | CIMBL7 | CIMBL70 | CIMBL71 | CIMBL72 | CIMBL73 | CIMBL74 | CIMBL75 | CIMBL76 | CIMBL77 | CIMBL78 | CIMBL79 |
| *ZmCCT8* | SV1 | AA | AA | AA | AA | AA | AA | TT | AA | AA | AA | AA | AA | AA | AA | AA | NN | AA | AA | AA | NN | AA | AA |
| *ZmCCT29* | SV2 | NN | NN | NN | NN | NN | NN | NN | NN | NN | NN | NN | AA | NN | NN | NN | NN | NN | NN | NN | NN | NN | NN |
|  | SV3 | TT | AA | TT | TT | NN | AA | NN | NN | NN | NN | AA | NN | NN | AA | TT | AA | TT | TT | NN | TT | TT | TT |
| *ZmCCT30* | SV4 | NN | NN | NN | NN | AA | NN | AA | AA | AA | NN | AA | NN | AA | NN | AA | NN | AA | AA | NN | NN | NN | AA |
|  | SV5 | NN | NN | TT | TT | NN | TT | NN | TT | NN | TT | NN | NN | NN | TT | TT | NN | NN | NN | NN | TT | NN | TT |
| *ZmCCT41* | SV6 | NN | NN | NN | AA | NN | NN | NN | AA | AA | NN | AA | AA | NN | AA | NN | NN | AA | NN | AA | AA | AA | AA |
|  | SV7 | TT | TT | NN | TT | TT | TT | NN | NN | TT | NN | NN | TT | NN | TT | TT | NN | TT | NN | NN | NN | NN | NN |
| *ZmCCT43* | SV8 | NN | NN | NN | NN | NN | NN | NN | NN | NN | NN | NN | NN | NN | NN | NN | NN | NN | NN | NN | NN | NN | NN |
|  | SV9 | TT | TT | TT | TT | TT | TT | TT | TT | TT | TT | TT | TT | TT | TT | TT | TT | TT | TT | TT | NN | TT | NN |
|  | SV10 | NN | TT | TT | TT | TT | TT | TT | TT | TT | TT | TT | TT | TT | TT | TT | TT | TT | TT | TT | TT | TT | NN |
|  | SV11 | NN | TT | TT | TT | TT | TT | TT | TT | TT | TT | TT | TT | TT | TT | TT | TT | TT | TT | TT | NN | TT | NN |
|  | SV12 | NN | TT | TT | TT | TT | TT | TT | TT | TT | TT | TT | TT | TT | TT | TT | TT | TT | TT | TT | TT | TT | NN |
|  | SV13 | NN | TT | TT | TT | NN | TT | TT | TT | TT | TT | TT | TT | TT | TT | TT | NN | TT | TT | TT | NN | TT | NN |
|  | SV14 | TT | NN | TT | TT | TT | NN | TT | TT | TT | TT | TT | TT | TT | TT | TT | TT | NN | TT | TT | TT | NN | TT |
|  | SV15 | TT | NN | TT | TT | TT | NN | TT | TT | TT | TT | TT | TT | TT | TT | TT | TT | TT | TT | TT | TT | NN | TT |

| **Table S6** *Continued*  AA, no difference in this locus when compared to B73 genome but difference in when compared to SK genome; NN, unable to judge ; SV, structural variation; TT, no difference in this locus when compared to SK genome but difference in when compared to B73 genome. | | | | | | | | | | | | | | | | | | | | | | | |
| --- | --- | --- | --- | --- | --- | --- | --- | --- | --- | --- | --- | --- | --- | --- | --- | --- | --- | --- | --- | --- | --- | --- | --- |
|  |  | Maize inbred line | | | | | | | | | | | | | | | | | | | | | |
| *ZmCCT* | SV | CIMBL8 | CIMBL80 | CIMBL81 | CIMBL82 | CIMBL83 | CIMBL84 | CIMBL85 | CIMBL86 | CIMBL87 | CIMBL88 | CIMBL89 | CIMBL9 | CIMBL90 | CIMBL91 | CIMBL92 | CIMBL93 | CIMBL94 | CIMBL95 | CIMBL96 | CIMBL97 | CIMBL98 | CIMBL99 |
| *ZmCCT8* | SV1 | TT | AA | TT | AA | TT | AA | AA | AA | AA | AA | AA | AA | AA | AA | AA | NN | AA | AA | AA | AA | AA | NN |
| *ZmCCT29* | SV2 | NN | NN | AA | AA | AA | NN | NN | NN | NN | NN | NN | NN | NN | NN | NN | NN | AA | AA | NN | NN | NN | NN |
|  | SV3 | NN | NN | AA | AA | NN | NN | NN | TT | NN | NN | NN | AA | AA | NN | NN | NN | NN | NN | TT | TT | NN | TT |
| *ZmCCT30* | SV4 | NN | AA | NN | AA | AA | AA | NN | NN | NN | NN | NN | NN | AA | NN | NN | AA | AA | AA | NN | NN | NN | NN |
|  | SV5 | NN | TT | TT | NN | NN | NN | NN | TT | AA | NN | TT | NN | TT | NN | TT | TT | NN | NN | NN | TT | TT | TT |
| *ZmCCT41* | SV6 | AA | AA | AA | NN | NN | NN | NN | NN | NN | NN | NN | NN | NN | NN | AA | AA | NN | NN | NN | NN | NN | NN |
|  | SV7 | NN | NN | TT | NN | NN | NN | TT | NN | TT | NN | TT | NN | TT | NN | NN | TT | TT | TT | TT | TT | TT | TT |
| *ZmCCT43* | SV8 | NN | NN | NN | NN | AA | NN | NN | NN | NN | NN | AA | NN | NN | AA | NN | NN | NN | NN | NN | NN | NN | NN |
|  | SV9 | TT | TT | TT | TT | TT | TT | TT | TT | TT | TT | TT | TT | TT | TT | TT | TT | TT | TT | TT | TT | TT | TT |
|  | SV10 | TT | TT | TT | TT | TT | TT | TT | TT | TT | TT | TT | TT | TT | TT | TT | TT | TT | TT | TT | TT | TT | TT |
|  | SV11 | TT | TT | TT | TT | TT | TT | TT | TT | TT | TT | TT | TT | TT | TT | TT | TT | TT | TT | TT | TT | TT | TT |
|  | SV12 | TT | TT | TT | TT | TT | TT | TT | TT | TT | TT | TT | TT | TT | TT | TT | TT | TT | TT | TT | TT | TT | TT |
|  | SV13 | TT | TT | TT | TT | TT | NN | TT | TT | TT | TT | NN | TT | TT | TT | NN | TT | TT | TT | NN | NN | TT | NN |
|  | SV14 | NN | TT | TT | TT | TT | TT | NN | TT | TT | TT | TT | NN | TT | TT | TT | NN | TT | TT | TT | NN | NN | TT |
|  | SV15 | NN | TT | TT | TT | TT | TT | NN | TT | TT | TT | TT | NN | TT | TT | TT | NN | TT | TT | TT | TT | TT | TT |

| **Table S6** *Continued*  AA, no difference in this locus when compared to B73 genome but difference in when compared to SK genome; NN, unable to judge ; SV, structural variation; TT, no difference in this locus when compared to SK genome but difference in when compared to B73 genome. | | | | | | | | | | | | | | | | | | | | | | | |
| --- | --- | --- | --- | --- | --- | --- | --- | --- | --- | --- | --- | --- | --- | --- | --- | --- | --- | --- | --- | --- | --- | --- | --- |
|  |  | Maize inbred line | | | | | | | | | | | | | | | | | | | | | |
| *ZmCCT* | SV | CML113 | CML114 | CML115 | CML118 | CML121 | CML122 | CML130 | CML134 | CML139 | CML162 | CML163 | CML165 | CML168 | CML169 | CML170 | CML171 | CML172 | CML189 | CML191 | CML192 | CML20 | CML223 |
| *ZmCCT8* | SV1 | AA | TT | AA | AA | AA | NN | AA | AA | NN | AA | NN | NN | AA | AA | AA | AA | AA | AA | NN | AA | AA | AA |
| *ZmCCT29* | SV2 | AA | NN | NN | NN | AA | NN | NN | NN | AA | NN | NN | NN | NN | NN | NN | NN | NN | NN | NN | NN | NN | NN |
|  | SV3 | NN | TT | NN | AA | NN | NN | NN | NN | NN | TT | NN | NN | NN | TT | NN | TT | TT | TT | NN | TT | TT | NN |
| *ZmCCT30* | SV4 | AA | NN | NN | NN | NN | AA | NN | AA | AA | AA | AA | NN | NN | NN | NN | NN | NN | NN | NN | AA | AA | AA |
|  | SV5 | TT | TT | NN | NN | TT | NN | TT | TT | TT | TT | NN | NN | TT | NN | TT | NN | NN | NN | NN | NN | NN | TT |
| *ZmCCT41* | SV6 | NN | NN | NN | NN | NN | NN | NN | NN | NN | NN | NN | AA | NN | NN | NN | NN | NN | NN | NN | NN | NN | AA |
|  | SV7 | TT | TT | TT | TT | TT | TT | TT | TT | TT | TT | NN | TT | TT | TT | TT | TT | TT | TT | TT | NN | TT | TT |
| *ZmCCT43* | SV8 | NN | NN | NN | NN | NN | NN | AA | NN | NN | NN | NN | NN | NN | NN | NN | NN | NN | NN | NN | NN | NN | NN |
|  | SV9 | TT | TT | TT | TT | TT | TT | TT | TT | TT | TT | TT | TT | TT | TT | NN | TT | TT | TT | TT | TT | TT | TT |
|  | SV10 | TT | TT | TT | TT | TT | TT | TT | TT | TT | TT | TT | TT | TT | TT | NN | TT | TT | TT | TT | TT | TT | TT |
|  | SV11 | TT | TT | TT | TT | TT | TT | TT | TT | TT | TT | TT | TT | TT | TT | NN | TT | TT | TT | TT | TT | NN | TT |
|  | SV12 | TT | TT | TT | TT | TT | TT | TT | TT | TT | TT | TT | TT | TT | TT | NN | TT | TT | TT | TT | TT | NN | TT |
|  | SV13 | TT | TT | NN | TT | NN | TT | TT | TT | TT | NN | TT | TT | TT | NN | NN | TT | TT | NN | TT | TT | NN | NN |
|  | SV14 | NN | TT | TT | NN | TT | NN | TT | TT | TT | TT | NN | TT | TT | TT | NN | NN | TT | TT | NN | TT | TT | NN |
|  | SV15 | NN | NN | TT | NN | TT | TT | TT | TT | TT | TT | NN | TT | TT | TT | NN | NN | TT | TT | NN | TT | TT | NN |

| **Table S6** *Continued*  AA, no difference in this locus when compared to B73 genome but difference in when compared to SK genome; NN, unable to judge ; SV, structural variation; TT, no difference in this locus when compared to SK genome but difference in when compared to B73 genome. | | | | | | | | | | | | | | | | | | | | | | | |
| --- | --- | --- | --- | --- | --- | --- | --- | --- | --- | --- | --- | --- | --- | --- | --- | --- | --- | --- | --- | --- | --- | --- | --- |
|  |  | Maize inbred line | | | | | | | | | | | | | | | | | | | | | |
| *ZmCCT* | SV | CML225 | CML226 | CML228 | CML229 | CML27 | CML28 | CML282 | CML285 | CML286 | CML287 | CML289 | CML29 | CML290 | CML298 | CML300 | CML304 | CML305 | CML307 | CML31 | CML32 | CML323 | CML324 |
| *ZmCCT8* | SV1 | NN | AA | AA | AA | AA | AA | AA | NN | AA | NN | AA | AA | TT | AA | AA | NN | AA | NN | AA | AA | AA | AA |
| *ZmCCT29* | SV2 | NN | NN | NN | NN | AA | NN | NN | NN | NN | AA | NN | NN | NN | NN | NN | AA | NN | NN | NN | NN | NN | NN |
|  | SV3 | TT | NN | NN | NN | NN | NN | TT | NN | TT | NN | TT | NN | TT | TT | TT | AA | TT | NN | NN | NN | NN | NN |
| *ZmCCT30* | SV4 | NN | AA | AA | NN | AA | NN | NN | NN | AA | AA | AA | AA | NN | AA | AA | NN | AA | NN | AA | AA | AA | NN |
|  | SV5 | NN | NN | TT | TT | TT | NN | NN | TT | TT | NN | NN | NN | NN | NN | NN | NN | NN | NN | NN | NN | TT | TT |
| *ZmCCT41* | SV6 | NN | NN | AA | NN | NN | NN | AA | NN | AA | NN | NN | NN | NN | AA | AA | AA | AA | AA | AA | AA | NN | NN |
|  | SV7 | NN | NN | NN | TT | NN | NN | TT | TT | TT | TT | NN | NN | NN | NN | NN | NN | NN | NN | NN | TT | NN | NN |
| *ZmCCT43* | SV8 | NN | NN | NN | NN | NN | NN | NN | NN | NN | NN | NN | NN | NN | NN | NN | NN | NN | NN | NN | NN | NN | NN |
|  | SV9 | TT | TT | TT | TT | TT | TT | TT | TT | TT | TT | TT | TT | TT | TT | TT | TT | TT | TT | TT | TT | TT | TT |
|  | SV10 | TT | TT | TT | TT | TT | TT | TT | TT | TT | TT | TT | TT | TT | TT | TT | TT | TT | TT | TT | TT | TT | TT |
|  | SV11 | NN | TT | TT | TT | TT | TT | TT | TT | TT | TT | TT | TT | TT | TT | TT | TT | TT | TT | TT | TT | TT | TT |
|  | SV12 | NN | TT | TT | TT | TT | TT | TT | TT | TT | TT | TT | TT | TT | TT | TT | TT | TT | TT | TT | TT | TT | TT |
|  | SV13 | NN | NN | TT | TT | TT | TT | TT | TT | TT | TT | TT | TT | NN | NN | NN | TT | TT | TT | TT | TT | TT | TT |
|  | SV14 | NN | NN | NN | TT | TT | TT | TT | TT | TT | TT | TT | TT | TT | NN | NN | NN | TT | TT | TT | TT | TT | TT |
|  | SV15 | NN | NN | NN | TT | TT | TT | TT | TT | TT | TT | TT | TT | TT | NN | NN | TT | TT | TT | TT | TT | TT | TT |

| **Table S6** *Continued*  AA, no difference in this locus when compared to B73 genome but difference in when compared to SK genome; NN, unable to judge; SV, structural variation; TT, no difference in this locus when compared to SK genome but difference in when compared to B73 genome. | | | | | | | | | | | | | | | | | | | | | | | |
| --- | --- | --- | --- | --- | --- | --- | --- | --- | --- | --- | --- | --- | --- | --- | --- | --- | --- | --- | --- | --- | --- | --- | --- |
|  |  | Maize inbred line | | | | | | | | | | | | | | | | | | | | | |
| *ZmCCT* | SV | CML325 | CML326 | CML327 | CML338 | CML360 | CML361 | CML364 | CML40 | CML408 | CML411 | CML412 | CML415 | CML422 | CML423 | CML426 | CML428 | CML431 | CML432 | CML433 | CML451 | CML454 | CML465 |
| *ZmCCT8* | SV1 | AA | AA | AA | TT | AA | AA | AA | AA | AA | NN | NN | AA | AA | AA | TT | AA | AA | AA | AA | NN | AA | AA |
| *ZmCCT29* | SV2 | NN | NN | AA | AA | NN | AA | NN | NN | NN | NN | NN | NN | NN | NN | NN | NN | NN | NN | NN | NN | NN | AA |
|  | SV3 | TT | TT | NN | NN | TT | AA | AA | NN | NN | NN | AA | TT | TT | TT | AA | NN | NN | NN | NN | NN | TT | NN |
| *ZmCCT30* | SV4 | NN | NN | AA | AA | AA | AA | AA | NN | AA | AA | NN | AA | NN | NN | NN | NN | NN | NN | AA | NN | AA | NN |
|  | SV5 | TT | NN | TT | NN | NN | NN | TT | TT | NN | NN | NN | TT | NN | NN | NN | TT | NN | NN | TT | TT | NN | TT |
| *ZmCCT41* | SV6 | NN | NN | NN | NN | NN | NN | NN | NN | NN | NN | NN | NN | NN | NN | AA | NN | AA | NN | NN | NN | NN | NN |
|  | SV7 | TT | NN | TT | TT | NN | TT | TT | NN | TT | TT | NN | NN | NN | NN | NN | NN | TT | NN | NN | NN | TT | TT |
| *ZmCCT43* | SV8 | NN | NN | NN | NN | NN | NN | NN | NN | NN | NN | NN | NN | NN | NN | NN | NN | NN | NN | NN | NN | NN | NN |
|  | SV9 | TT | TT | TT | TT | TT | TT | TT | TT | TT | TT | TT | TT | TT | TT | TT | TT | TT | TT | TT | NN | TT | TT |
|  | SV10 | TT | TT | TT | TT | NN | TT | TT | TT | TT | TT | TT | TT | TT | TT | TT | TT | TT | TT | TT | NN | TT | TT |
|  | SV11 | TT | TT | TT | TT | NN | TT | TT | TT | TT | TT | TT | TT | TT | TT | TT | TT | TT | TT | TT | NN | TT | TT |
|  | SV12 | TT | TT | TT | TT | NN | TT | TT | TT | TT | TT | TT | TT | TT | TT | TT | TT | TT | TT | TT | NN | TT | TT |
|  | SV13 | NN | TT | TT | TT | NN | TT | TT | TT | TT | TT | TT | TT | TT | TT | TT | TT | TT | TT | TT | NN | TT | TT |
|  | SV14 | TT | NN | TT | TT | TT | NN | TT | TT | TT | TT | TT | TT | TT | TT | TT | TT | TT | TT | TT | TT | NN | TT |
|  | SV15 | TT | TT | TT | TT | TT | NN | TT | TT | TT | TT | TT | NN | TT | TT | TT | TT | TT | TT | TT | TT | NN | TT |

| **Table S6** *Continued*  AA, no difference in this locus when compared to B73 genome but difference in when compared to SK genome; NN, unable to judge; SV, structural variation; TT, no difference in this locus when compared to SK genome but difference in when compared to B73 genome. | | | | | | | | | | | | | | | | | | | | | | | |
| --- | --- | --- | --- | --- | --- | --- | --- | --- | --- | --- | --- | --- | --- | --- | --- | --- | --- | --- | --- | --- | --- | --- | --- |
|  |  | Maize inbred line | | | | | | | | | | | | | | | | | | | | | |
| *ZmCCT* | SV | CML468 | CML470 | CML471 | CML473 | CML479 | CML480 | CML486 | CML493 | CML496 | CML497 | CML50 | CML51 | CML69 | CY72 | D047 | D863F | DAN3130 | DAN340 | DAN360 | DAN4245 | DAN598 | DAN599 |
| *ZmCCT8* | SV1 | AA | NN | NN | AA | NN | AA | AA | AA | NN | AA | AA | AA | NN | NN | AA | AA | NN | AA | AA | NN | NN | AA |
| *ZmCCT29* | SV2 | NN | NN | NN | NN | NN | NN | NN | NN | NN | NN | NN | NN | NN | NN | NN | NN | NN | NN | NN | NN | NN | NN |
|  | SV3 | TT | AA | NN | NN | NN | TT | AA | NN | NN | TT | AA | NN | NN | NN | NN | TT | TT | TT | TT | TT | NN | TT |
| *ZmCCT30* | SV4 | AA | NN | AA | AA | NN | NN | NN | AA | AA | AA | AA | AA | NN | NN | AA | NN | NN | NN | NN | NN | NN | NN |
|  | SV5 | NN | TT | TT | TT | NN | NN | NN | NN | NN | TT | NN | TT | NN | TT | NN | NN | TT | TT | NN | TT | TT | NN |
| *ZmCCT41* | SV6 | NN | NN | AA | NN | NN | NN | NN | AA | AA | AA | NN | AA | AA | NN | NN | AA | AA | NN | NN | NN | NN | NN |
|  | SV7 | TT | NN | TT | TT | NN | TT | NN | NN | NN | TT | NN | NN | NN | TT | NN | NN | TT | NN | NN | NN | NN | NN |
| *ZmCCT43* | SV8 | NN | NN | NN | NN | NN | NN | NN | NN | NN | NN | NN | NN | NN | NN | NN | NN | AA | NN | NN | AA | NN | NN |
|  | SV9 | TT | TT | TT | TT | TT | TT | TT | TT | TT | TT | TT | TT | TT | TT | TT | TT | NN | NN | TT | TT | TT | TT |
|  | SV10 | TT | TT | TT | TT | TT | TT | TT | TT | TT | TT | TT | TT | NN | TT | TT | TT | NN | NN | TT | TT | TT | TT |
|  | SV11 | TT | TT | TT | TT | TT | TT | TT | TT | TT | TT | TT | TT | NN | TT | TT | TT | NN | NN | TT | NN | NN | TT |
|  | SV12 | TT | TT | TT | TT | TT | TT | TT | TT | TT | TT | TT | TT | NN | TT | TT | TT | NN | NN | TT | NN | TT | TT |
|  | SV13 | TT | TT | TT | TT | TT | TT | TT | TT | TT | TT | TT | TT | NN | TT | TT | TT | NN | NN | TT | NN | NN | TT |
|  | SV14 | TT | TT | TT | TT | TT | TT | TT | TT | TT | TT | TT | TT | TT | NN | TT | TT | TT | NN | NN | TT | NN | NN |
|  | SV15 | TT | TT | TT | TT | TT | TT | TT | TT | TT | TT | TT | TT | TT | NN | TT | TT | TT | NN | NN | TT | NN | NN |

| **Table S6** *Continued*  AA, no difference in this locus when compared to B73 genome but difference in when compared to SK genome; NN, unable to judge; SV, structural variation; TT, no difference in this locus when compared to SK genome but difference in when compared to B73 genome. | | | | | | | | | | | | | | | | | | | | | | | |
| --- | --- | --- | --- | --- | --- | --- | --- | --- | --- | --- | --- | --- | --- | --- | --- | --- | --- | --- | --- | --- | --- | --- | --- |
|  |  | Maize inbred line | | | | | | | | | | | | | | | | | | | | | |
| *ZmCCT* | SV | DAN9046 | DE.EX | DH29 | DH3732 | DONG237 | DONG46 | DSB | E28 | EN25 | ES40 | FCD0602 | GEMS1 | GEMS10 | GEMS11 | GEMS12 | GEMS13 | GEMS14 | GEMS15 | GEMS16 | GEMS17 | GEMS18 | GEMS19 |
| *ZmCCT8* | SV1 | AA | AA | AA | AA | NN | AA | AA | AA | NN | NN | AA | AA | AA | AA | NN | AA | AA | AA | AA | AA | AA | NN |
| *ZmCCT29* | SV2 | NN | NN | NN | AA | NN | AA | NN | AA | NN | AA | AA | NN | AA | NN | NN | AA | AA | NN | NN | NN | AA | NN |
|  | SV3 | NN | AA | NN | NN | AA | NN | NN | NN | TT | AA | NN | TT | NN | AA | TT | NN | NN | NN | AA | NN | NN | NN |
| *ZmCCT30* | SV4 | NN | NN | NN | NN | AA | AA | AA | NN | NN | NN | AA | NN | AA | AA | NN | AA | AA | NN | AA | AA | AA | NN |
|  | SV5 | TT | TT | TT | NN | TT | NN | NN | NN | NN | NN | TT | NN | NN | NN | NN | TT | NN | NN | NN | NN | TT | NN |
| *ZmCCT41* | SV6 | AA | NN | NN | NN | NN | NN | NN | NN | NN | NN | NN | NN | AA | AA | NN | NN | NN | NN | AA | AA | NN | AA |
|  | SV7 | NN | TT | TT | TT | TT | TT | TT | TT | NN | NN | TT | NN | NN | NN | TT | NN | NN | NN | NN | NN | NN | TT |
| *ZmCCT43* | SV8 | NN | NN | NN | NN | NN | NN | NN | NN | NN | NN | NN | NN | AA | AA | NN | AA | NN | NN | NN | NN | AA | AA |
|  | SV9 | NN | TT | TT | TT | TT | TT | TT | TT | NN | NN | TT | TT | NN | NN | TT | TT | TT | TT | TT | TT | NN | NN |
|  | SV10 | NN | TT | TT | TT | TT | TT | TT | TT | NN | NN | TT | TT | NN | NN | TT | TT | TT | TT | TT | TT | NN | NN |
|  | SV11 | NN | TT | TT | TT | TT | TT | NN | TT | NN | NN | TT | TT | NN | NN | TT | NN | TT | TT | TT | TT | NN | NN |
|  | SV12 | TT | TT | TT | TT | TT | TT | TT | TT | TT | NN | TT | TT | NN | NN | TT | NN | TT | TT | TT | TT | NN | NN |
|  | SV13 | NN | TT | TT | TT | TT | TT | TT | NN | NN | NN | TT | TT | NN | NN | TT | NN | TT | TT | TT | TT | NN | NN |
|  | SV14 | TT | NN | TT | TT | TT | TT | TT | TT | NN | NN | NN | TT | TT | NN | NN | TT | NN | TT | TT | TT | TT | NN |
|  | SV15 | TT | NN | TT | TT | TT | TT | TT | TT | NN | NN | NN | TT | TT | NN | NN | TT | NN | TT | TT | TT | TT | NN |

| **Table S6** *Continued*  AA, no difference in this locus when compared to B73 genome but difference in when compared to SK genome; NN, unable to judge; SV, structural variation; TT, no difference in this locus when compared to SK genome but difference in when compared to B73 genome. | | | | | | | | | | | | | | | | | | | | | | | |
| --- | --- | --- | --- | --- | --- | --- | --- | --- | --- | --- | --- | --- | --- | --- | --- | --- | --- | --- | --- | --- | --- | --- | --- |
|  |  | Maize inbred line | | | | | | | | | | | | | | | | | | | | | |
| *ZmCCT* | SV | GEMS2 | GEMS20 | GEMS21 | GEMS23 | GEMS24 | GEMS25 | GEMS27 | GEMS28 | GEMS29 | GEMS3 | GEMS30 | GEMS31 | GEMS32 | GEMS33 | GEMS35 | GEMS36 | GEMS37 | GEMS39 | GEMS4 | GEMS40 | GEMS41 | GEMS42 |
| *ZmCCT8* | SV1 | TT | AA | AA | AA | TT | AA | NN | TT | TT | AA | NN | AA | AA | NN | NN | AA | NN | AA | AA | AA | AA | NN |
| *ZmCCT29* | SV2 | NN | AA | AA | NN | NN | NN | NN | NN | NN | NN | NN | NN | NN | AA | NN | NN | AA | NN | AA | NN | NN | NN |
|  | SV3 | NN | NN | AA | TT | NN | AA | NN | AA | AA | AA | NN | TT | NN | NN | TT | TT | NN | TT | NN | TT | TT | NN |
| *ZmCCT30* | SV4 | NN | AA | AA | AA | NN | NN | NN | NN | NN | AA | AA | AA | AA | NN | NN | NN | NN | NN | AA | NN | NN | AA |
|  | SV5 | NN | NN | NN | NN | NN | TT | NN | TT | TT | TT | NN | NN | NN | TT | TT | TT | TT | TT | TT | NN | NN | TT |
| *ZmCCT41* | SV6 | NN | AA | AA | AA | AA | AA | NN | AA | AA | NN | NN | AA | NN | NN | NN | NN | NN | NN | NN | AA | NN | NN |
|  | SV7 | TT | NN | NN | NN | NN | TT | NN | NN | TT | TT | NN | TT | TT | TT | TT | NN | TT | TT | NN | TT | TT | NN |
| *ZmCCT43* | SV8 | NN | NN | AA | AA | NN | NN | NN | NN | NN | NN | NN | NN | NN | NN | NN | NN | NN | NN | AA | AA | NN | NN |
|  | SV9 | TT | TT | TT | NN | TT | TT | TT | TT | TT | TT | TT | TT | TT | TT | TT | TT | TT | TT | TT | TT | TT | TT |
|  | SV10 | TT | TT | TT | NN | TT | TT | TT | TT | TT | TT | TT | TT | TT | TT | TT | TT | TT | TT | TT | TT | TT | TT |
|  | SV11 | TT | TT | NN | NN | TT | TT | TT | TT | TT | TT | TT | TT | TT | TT | TT | TT | TT | TT | NN | TT | TT | TT |
|  | SV12 | TT | TT | TT | NN | TT | TT | TT | TT | TT | TT | TT | TT | TT | TT | TT | TT | TT | TT | NN | TT | TT | TT |
|  | SV13 | TT | TT | NN | NN | NN | TT | TT | TT | TT | TT | TT | NN | TT | TT | TT | TT | TT | TT | NN | TT | TT | TT |
|  | SV14 | NN | TT | TT | NN | NN | NN | TT | TT | TT | TT | TT | TT | NN | TT | TT | TT | TT | TT | TT | NN | TT | TT |
|  | SV15 | NN | TT | TT | NN | NN | NN | TT | TT | TT | TT | TT | TT | NN | TT | TT | TT | TT | TT | TT | NN | TT | TT |

| **Table S6** *Continued*  AA, no difference in this locus when compared to B73 genome but difference in when compared to SK genome; NN, unable to judge; SV, structural variation; TT, no difference in this locus when compared to SK genome but difference in when compared to B73 genome. | | | | | | | | | | | | | | | | | | | | | | | |
| --- | --- | --- | --- | --- | --- | --- | --- | --- | --- | --- | --- | --- | --- | --- | --- | --- | --- | --- | --- | --- | --- | --- | --- |
|  |  | Maize inbred line | | | | | | | | | | | | | | | | | | | | | |
| *ZmCCT* | SV | GEMS43 | GEMS44 | GEMS45 | GEMS46 | GEMS47 | GEMS48 | GEMS49 | GEMS5 | GEMS50 | GEMS51 | GEMS52 | GEMS53 | GEMS54 | GEMS55 | GEMS56 | GEMS57 | GEMS58 | GEMS59 | GEMS6 | GEMS60 | GEMS61 | GEMS62 |
| *ZmCCT8* | SV1 | AA | AA | NN | AA | AA | AA | AA | NN | AA | AA | AA | AA | AA | AA | NN | NN | NN | AA | AA | NN | NN | AA |
| *ZmCCT29* | SV2 | NN | NN | NN | AA | NN | NN | AA | AA | AA | AA | AA | AA | NN | AA | NN | NN | NN | NN | NN | NN | NN | NN |
|  | SV3 | TT | NN | NN | NN | TT | AA | AA | AA | AA | AA | NN | NN | NN | AA | TT | TT | TT | TT | AA | TT | TT | NN |
| *ZmCCT30* | SV4 | NN | NN | NN | NN | NN | NN | NN | AA | NN | AA | AA | AA | NN | AA | NN | NN | NN | AA | NN | NN | NN | NN |
|  | SV5 | NN | TT | NN | NN | TT | NN | TT | NN | NN | NN | NN | NN | NN | TT | NN | NN | TT | TT | NN | NN | NN | NN |
| *ZmCCT41* | SV6 | NN | NN | NN | NN | NN | NN | NN | NN | NN | NN | NN | NN | AA | NN | AA | NN | NN | NN | NN | AA | NN | AA |
|  | SV7 | TT | NN | TT | NN | TT | NN | TT | TT | NN | TT | TT | NN | NN | NN | NN | TT | TT | NN | NN | NN | NN | NN |
| *ZmCCT43* | SV8 | NN | NN | NN | NN | NN | AA | NN | NN | AA | NN | NN | AA | AA | AA | NN | NN | NN | NN | NN | NN | AA | NN |
|  | SV9 | TT | TT | TT | TT | TT | TT | TT | TT | NN | TT | TT | NN | NN | TT | TT | TT | TT | TT | TT | TT | NN | TT |
|  | SV10 | TT | TT | TT | TT | TT | TT | TT | TT | NN | TT | TT | NN | NN | TT | TT | TT | TT | TT | TT | TT | NN | TT |
|  | SV11 | TT | TT | TT | TT | TT | TT | TT | TT | NN | TT | TT | NN | NN | NN | TT | TT | TT | TT | TT | TT | NN | TT |
|  | SV12 | TT | TT | TT | TT | TT | TT | TT | TT | NN | TT | TT | NN | NN | TT | TT | TT | TT | TT | TT | TT | NN | TT |
|  | SV13 | NN | NN | TT | TT | TT | TT | TT | TT | NN | TT | TT | NN | NN | NN | TT | TT | TT | TT | NN | TT | NN | TT |
|  | SV14 | TT | NN | NN | TT | TT | TT | TT | TT | TT | NN | TT | TT | NN | NN | NN | TT | TT | TT | TT | NN | TT | NN |
|  | SV15 | TT | NN | NN | TT | TT | TT | TT | TT | TT | NN | TT | TT | NN | NN | NN | TT | TT | NN | TT | NN | TT | NN |

| **Table S6** *Continued*  AA, no difference in this locus when compared to B73 genome but difference in when compared to SK genome; NN, unable to judge; SV, structural variation; TT, no difference in this locus when compared to SK genome but difference in when compared to B73 genome. | | | | | | | | | | | | | | | | | | | | | | | |
| --- | --- | --- | --- | --- | --- | --- | --- | --- | --- | --- | --- | --- | --- | --- | --- | --- | --- | --- | --- | --- | --- | --- | --- |
|  |  | Maize inbred line | | | | | | | | | | | | | | | | | | | | | |
| *ZmCCT* | SV | GEMS63 | GEMS64 | GEMS65 | GEMS66 | GEMS9 | GY1032 | GY220 | GY237 | GY386 | GY386B | GY462 | GY798 | GY923 | H21 | HB | HSBN | HTH-17 | HU803 | HUA83-2 | HUANGC | HYS | HZS |
| *ZmCCT8* | SV1 | AA | AA | AA | NN | AA | TT | NN | TT | NN | TT | NN | AA | NN | AA | AA | TT | NN | AA | AA | AA | AA | AA |
| *ZmCCT29* | SV2 | AA | AA | AA | NN | NN | NN | NN | NN | NN | NN | NN | AA | NN | NN | NN | AA | NN | AA | NN | NN | NN | NN |
|  | SV3 | NN | NN | NN | NN | NN | NN | NN | NN | TT | TT | AA | NN | NN | NN | TT | AA | TT | NN | TT | TT | NN | NN |
| *ZmCCT30* | SV4 | AA | AA | AA | NN | NN | NN | NN | NN | NN | NN | NN | NN | AA | NN | NN | NN | NN | AA | NN | NN | NN | NN |
|  | SV5 | TT | NN | NN | NN | NN | NN | NN | TT | NN | TT | NN | TT | TT | NN | TT | NN | NN | NN | NN | NN | TT | NN |
| *ZmCCT41* | SV6 | NN | AA | AA | NN | NN | AA | NN | NN | NN | NN | NN | NN | NN | NN | NN | NN | AA | NN | NN | NN | NN | NN |
|  | SV7 | NN | NN | TT | TT | NN | TT | TT | TT | TT | TT | TT | TT | TT | TT | TT | NN | TT | TT | TT | TT | NN | TT |
| *ZmCCT43* | SV8 | AA | AA | AA | NN | NN | NN | NN | NN | NN | NN | NN | NN | NN | NN | NN | NN | NN | NN | NN | NN | NN | NN |
|  | SV9 | NN | NN | NN | TT | TT | TT | TT | TT | TT | TT | TT | TT | TT | TT | TT | TT | TT | TT | TT | TT | TT | TT |
|  | SV10 | NN | NN | NN | TT | TT | TT | TT | TT | TT | TT | TT | TT | TT | TT | TT | TT | TT | TT | TT | TT | TT | TT |
|  | SV11 | NN | NN | NN | TT | TT | TT | TT | TT | TT | TT | TT | TT | TT | TT | TT | TT | TT | TT | TT | TT | TT | TT |
|  | SV12 | NN | NN | NN | TT | TT | TT | TT | TT | TT | TT | TT | TT | TT | TT | TT | TT | TT | TT | TT | TT | TT | TT |
|  | SV13 | NN | NN | NN | NN | TT | TT | TT | TT | NN | TT | TT | TT | TT | TT | TT | NN | TT | TT | TT | NN | TT | TT |
|  | SV14 | TT | NN | NN | NN | NN | TT | TT | TT | TT | NN | TT | TT | TT | TT | TT | TT | NN | TT | TT | TT | NN | TT |
|  | SV15 | TT | NN | NN | NN | NN | TT | TT | TT | TT | TT | TT | TT | TT | TT | TT | TT | NN | TT | TT | TT | TT | TT |

| **Table S6** *Continued*  AA, no difference in this locus when compared to B73 genome but difference in when compared to SK genome; NN, unable to judge; SV, structural variation; TT, no difference in this locus when compared to SK genome but difference in when compared to B73 genome. | | | | | | | | | | | | | | | | | | | | | | | |
| --- | --- | --- | --- | --- | --- | --- | --- | --- | --- | --- | --- | --- | --- | --- | --- | --- | --- | --- | --- | --- | --- | --- | --- |
|  |  | Maize inbred line | | | | | | | | | | | | | | | | | | | | | |
| *ZmCCT* | SV | IRF291 | IRF314 | JH59 | JH96C | JI53 | JI63 | JI842 | JI846 | JI853 | JIAO51 | JING24 | JING724 | JY01 | K10 | K12 | K14 | K22 | L3180 | LG001 | LIAO138 | LIAO159 | LIAO5114 |
| *ZmCCT8* | SV1 | AA | NN | TT | AA | NN | NN | NN | AA | AA | NN | AA | NN | NN | AA | NN | AA | AA | NN | AA | AA | NN | AA |
| *ZmCCT29* | SV2 | NN | NN | NN | NN | NN | NN | NN | NN | NN | NN | AA | NN | NN | NN | NN | NN | NN | NN | NN | NN | AA | NN |
|  | SV3 | TT | TT | TT | TT | TT | NN | TT | TT | NN | NN | NN | TT | TT | NN | NN | TT | TT | AA | NN | NN | NN | TT |
| *ZmCCT30* | SV4 | NN | NN | NN | NN | NN | NN | NN | NN | NN | NN | NN | NN | NN | NN | AA | NN | NN | AA | NN | NN | AA | NN |
|  | SV5 | NN | TT | NN | TT | TT | NN | NN | NN | TT | NN | NN | TT | NN | TT | NN | NN | TT | TT | NN | TT | NN | NN |
| *ZmCCT41* | SV6 | NN | NN | NN | NN | NN | NN | NN | NN | NN | NN | AA | NN | NN | AA | NN | AA | NN | NN | NN | NN | NN | NN |
|  | SV7 | TT | NN | TT | TT | TT | TT | TT | TT | TT | NN | TT | NN | NN | TT | NN | TT | TT | TT | TT | TT | TT | TT |
| *ZmCCT43* | SV8 | NN | NN | NN | NN | NN | NN | NN | NN | NN | NN | NN | NN | NN | NN | AA | NN | NN | NN | NN | NN | NN | AA |
|  | SV9 | TT | TT | TT | TT | TT | TT | TT | TT | TT | NN | TT | TT | TT | TT | NN | TT | TT | TT | TT | TT | TT | NN |
|  | SV10 | TT | TT | TT | TT | TT | TT | TT | TT | TT | NN | TT | TT | TT | TT | NN | TT | TT | TT | TT | TT | TT | NN |
|  | SV11 | TT | TT | TT | TT | TT | TT | TT | TT | TT | NN | TT | TT | TT | NN | NN | TT | TT | TT | TT | TT | TT | NN |
|  | SV12 | TT | TT | TT | TT | TT | TT | TT | TT | TT | TT | TT | TT | TT | TT | NN | TT | TT | TT | TT | TT | TT | NN |
|  | SV13 | TT | TT | TT | TT | TT | TT | TT | TT | NN | NN | NN | TT | TT | NN | NN | TT | NN | NN | TT | TT | TT | NN |
|  | SV14 | TT | TT | TT | TT | TT | TT | TT | TT | TT | NN | NN | NN | TT | TT | NN | NN | TT | NN | NN | TT | TT | TT |
|  | SV15 | TT | TT | TT | TT | TT | TT | TT | TT | TT | NN | NN | NN | TT | TT | NN | NN | TT | TT | NN | TT | TT | TT |

| **Table S6** *Continued*  AA, no difference in this locus when compared to B73 genome but difference in when compared to SK genome; NN, unable to judge; SV, structural variation; TT, no difference in this locus when compared to SK genome but difference in when compared to B73 genome. | | | | | | | | | | | | | | | | | | | | | | | |
| --- | --- | --- | --- | --- | --- | --- | --- | --- | --- | --- | --- | --- | --- | --- | --- | --- | --- | --- | --- | --- | --- | --- | --- |
|  |  | Maize inbred line | | | | | | | | | | | | | | | | | | | | | |
| *ZmCCT* | SV | LIAO5262 | LIAO5263 | LK11 | LV28 | LXN | LY | LY042 | M153 | M165 | M97 | MN | MO113 | MO17 | NAN21-3 | NMJT | P138 | P178 | P6WC | PH4VC | Q1261 | QI205 | QI319 |
| *ZmCCT8* | SV1 | NN | NN | NN | NN | NN | AA | AA | AA | AA | NN | AA | NN | NN | AA | AA | NN | AA | NN | NN | AA | NN | AA |
| *ZmCCT29* | SV2 | NN | NN | NN | NN | NN | NN | NN | NN | NN | NN | NN | NN | NN | AA | AA | NN | NN | AA | NN | NN | AA | NN |
|  | SV3 | TT | TT | TT | NN | AA | TT | TT | TT | TT | TT | NN | TT | TT | NN | AA | NN | TT | NN | TT | NN | NN | NN |
| *ZmCCT30* | SV4 | NN | NN | NN | NN | NN | NN | NN | NN | NN | NN | NN | NN | NN | NN | NN | NN | NN | NN | NN | AA | NN | NN |
|  | SV5 | NN | TT | NN | NN | NN | TT | NN | TT | TT | NN | TT | NN | NN | NN | TT | TT | TT | TT | TT | NN | NN | NN |
| *ZmCCT41* | SV6 | NN | NN | NN | NN | NN | NN | NN | NN | NN | NN | NN | AA | NN | NN | NN | NN | NN | NN | NN | NN | NN | AA |
|  | SV7 | NN | NN | TT | TT | NN | TT | TT | TT | TT | TT | NN | NN | TT | TT | TT | TT | TT | TT | NN | TT | NN | TT |
| *ZmCCT43* | SV8 | NN | AA | NN | NN | NN | NN | NN | NN | NN | NN | NN | NN | NN | NN | NN | NN | NN | AA | NN | AA | NN | NN |
|  | SV9 | TT | NN | TT | TT | TT | TT | TT | TT | TT | TT | TT | TT | TT | TT | TT | TT | TT | NN | TT | NN | TT | TT |
|  | SV10 | TT | NN | TT | TT | TT | TT | TT | TT | TT | TT | TT | TT | TT | TT | TT | TT | TT | NN | TT | NN | TT | TT |
|  | SV11 | TT | NN | TT | TT | TT | TT | TT | TT | TT | TT | TT | TT | TT | TT | TT | TT | TT | NN | TT | NN | TT | TT |
|  | SV12 | TT | NN | TT | TT | TT | TT | TT | TT | TT | TT | TT | TT | TT | TT | TT | TT | TT | NN | TT | NN | TT | TT |
|  | SV13 | TT | NN | NN | NN | TT | TT | TT | NN | TT | TT | TT | NN | TT | TT | TT | NN | NN | NN | TT | NN | TT | TT |
|  | SV14 | NN | TT | NN | NN | NN | TT | TT | TT | NN | TT | TT | TT | NN | TT | TT | TT | NN | NN | NN | TT | NN | TT |
|  | SV15 | NN | TT | NN | NN | NN | TT | TT | TT | NN | TT | TT | TT | NN | TT | TT | TT | NN | NN | NN | TT | NN | TT |

| **Table S6** *Continued*  AA, no difference in this locus when compared to B73 genome but difference in when compared to SK genome; NN, unable to judge; SV, structural variation; TT, no difference in this locus when compared to SK genome but difference in when compared to B73 genome. | | | | | | | | | | | | | | | | | | | | | | | |
| --- | --- | --- | --- | --- | --- | --- | --- | --- | --- | --- | --- | --- | --- | --- | --- | --- | --- | --- | --- | --- | --- | --- | --- |
|  |  | Maize inbred line | | | | | | | | | | | | | | | | | | | | | |
| *ZmCCT* | SV | R08 | R15 | R15X1141 | RY684 | RY697 | RY713 | RY729 | RY732 | RY737 | S22 | S37 | SC55 | SHEN137 | SHEN5003 | SI273 | SI434 | SI444 | SI446 | SK | SW1611 | SW92E114 | SY1032 |
| *ZmCCT8* | SV1 | NN | NN | AA | NN | AA | NN | NN | NN | TT | AA | AA | AA | AA | AA | AA | AA | AA | AA | NN | AA | AA | AA |
| *ZmCCT29* | SV2 | NN | NN | NN | AA | NN | NN | NN | NN | NN | NN | NN | NN | NN | NN | NN | NN | AA | NN | NN | AA | NN | NN |
|  | SV3 | AA | TT | TT | NN | TT | TT | NN | NN | AA | TT | TT | NN | NN | TT | NN | NN | NN | TT | TT | AA | TT | TT |
| *ZmCCT30* | SV4 | NN | NN | NN | AA | NN | NN | NN | NN | NN | NN | NN | NN | NN | NN | NN | AA | NN | NN | NN | AA | AA | NN |
|  | SV5 | TT | NN | NN | NN | NN | NN | TT | TT | NN | NN | TT | TT | NN | NN | TT | NN | NN | TT | TT | TT | NN | NN |
| *ZmCCT41* | SV6 | NN | NN | NN | NN | NN | NN | NN | NN | NN | NN | NN | NN | NN | AA | NN | NN | AA | NN | NN | NN | AA | AA |
|  | SV7 | NN | TT | TT | TT | TT | TT | TT | TT | TT | NN | TT | NN | NN | TT | NN | NN | TT | NN | TT | NN | TT | TT |
| *ZmCCT43* | SV8 | NN | NN | NN | NN | NN | NN | NN | NN | NN | NN | NN | NN | NN | NN | NN | NN | NN | NN | NN | NN | NN | NN |
|  | SV9 | TT | TT | TT | TT | NN | TT | TT | TT | TT | TT | TT | TT | NN | TT | TT | TT | TT | TT | TT | TT | TT | TT |
|  | SV10 | TT | TT | TT | TT | NN | TT | TT | TT | TT | TT | TT | TT | NN | TT | TT | TT | TT | TT | TT | TT | TT | TT |
|  | SV11 | TT | TT | TT | TT | NN | TT | TT | TT | TT | TT | TT | TT | TT | TT | TT | TT | TT | TT | TT | TT | TT | TT |
|  | SV12 | TT | TT | TT | TT | NN | TT | TT | TT | TT | TT | TT | TT | NN | TT | TT | TT | TT | TT | TT | TT | TT | TT |
|  | SV13 | TT | TT | TT | NN | NN | TT | NN | TT | TT | TT | TT | TT | NN | TT | TT | TT | NN | TT | TT | TT | NN | TT |
|  | SV14 | TT | TT | TT | TT | NN | NN | TT | NN | TT | TT | TT | TT | TT | NN | TT | TT | TT | NN | TT | TT | TT | NN |
|  | SV15 | TT | TT | TT | TT | NN | NN | TT | NN | TT | TT | TT | TT | TT | NN | TT | TT | TT | NN | TT | TT | TT | TT |

| **Table S6** *Continued*  AA, no difference in this locus when compared to B73 genome but difference in when compared to SK genome; NN, unable to judge; SV, structural variation; TT, no difference in this locus when compared to SK genome but difference in when compared to B73 genome. | | | | | | | | | | | | | | | | | | | | | | | |
| --- | --- | --- | --- | --- | --- | --- | --- | --- | --- | --- | --- | --- | --- | --- | --- | --- | --- | --- | --- | --- | --- | --- | --- |
|  |  | Maize inbred line | | | | | | | | | | | | | | | | | | | | | |
| *ZmCCT* | SV | SY1035 | SY1039 | SY1052 | SY1077 | SY1128 | SY3073 | SY998 | SY999 | TIAN77 | TIE7922 | TT16 | TX5 | TY1 | TY10 | TY11 | TY2 | TY3 | TY4 | TY5 | TY6 | TY7 | TY8 |
| *ZmCCT8* | SV1 | NN | TT | TT | NN | NN | AA | AA | AA | NN | AA | NN | AA | AA | AA | AA | AA | AA | AA | AA | AA | AA | AA |
| *ZmCCT29* | SV2 | NN | NN | NN | NN | NN | NN | NN | NN | AA | AA | NN | NN | NN | NN | NN | NN | NN | NN | NN | NN | NN | NN |
|  | SV3 | TT | NN | TT | TT | TT | TT | NN | NN | AA | AA | NN | NN | NN | TT | TT | AA | NN | AA | AA | TT | TT | TT |
| *ZmCCT30* | SV4 | NN | AA | NN | NN | NN | NN | NN | AA | NN | AA | NN | NN | NN | NN | NN | NN | NN | NN | NN | NN | NN | NN |
|  | SV5 | NN | NN | NN | NN | NN | NN | TT | NN | NN | TT | NN | NN | NN | NN | TT | TT | NN | NN | NN | NN | TT | TT |
| *ZmCCT41* | SV6 | NN | NN | NN | NN | NN | NN | NN | NN | NN | AA | NN | NN | NN | NN | NN | NN | NN | NN | NN | NN | NN | NN |
|  | SV7 | TT | TT | TT | NN | TT | TT | TT | TT | NN | TT | TT | TT | TT | TT | TT | TT | TT | TT | TT | TT | TT | TT |
| *ZmCCT43* | SV8 | AA | NN | NN | NN | NN | NN | NN | NN | NN | AA | NN | NN | NN | NN | NN | NN | NN | NN | NN | AA | NN | NN |
|  | SV9 | NN | TT | TT | TT | TT | TT | TT | TT | TT | TT | TT | TT | TT | TT | TT | TT | TT | TT | TT | TT | TT | TT |
|  | SV10 | NN | TT | TT | TT | TT | TT | TT | TT | TT | TT | TT | TT | TT | TT | TT | TT | TT | TT | TT | TT | TT | TT |
|  | SV11 | NN | TT | TT | TT | TT | TT | TT | TT | TT | NN | TT | TT | TT | TT | TT | TT | TT | TT | TT | TT | TT | TT |
|  | SV12 | NN | TT | TT | TT | TT | TT | TT | TT | TT | NN | TT | TT | TT | TT | TT | TT | TT | TT | TT | TT | TT | TT |
|  | SV13 | NN | TT | NN | TT | TT | TT | TT | TT | TT | NN | TT | TT | TT | TT | TT | NN | TT | TT | TT | NN | NN | TT |
|  | SV14 | TT | NN | TT | NN | TT | TT | TT | TT | TT | TT | NN | TT | TT | TT | TT | TT | NN | TT | TT | TT | NN | NN |
|  | SV15 | TT | NN | TT | NN | TT | TT | TT | TT | TT | TT | NN | TT | TT | TT | TT | TT | TT | TT | TT | TT | NN | NN |

| **Table S6** *Continued*  AA, no difference in this locus when compared to B73 genome but difference in when compared to SK genome; NN, unable to judge; SV, structural variation; TT, no difference in this locus when compared to SK genome but difference in when compared to B73 genome. | | | | | | | | | | | | | | | | | | | | | | | |
| --- | --- | --- | --- | --- | --- | --- | --- | --- | --- | --- | --- | --- | --- | --- | --- | --- | --- | --- | --- | --- | --- | --- | --- |
|  |  | Maize inbred line | | | | | | | | | | | | | | | | | | | | | |
| *ZmCCT* | SV | TY9 | U8112 | W138 | WH413 | WMR | WU109 | XI502 | XUN971 | XZ698 | YAN414 | YE107 | YE478 | YE488 | YE515 | YE52106 | YE8001 | YU374 | YU87-1 | Z2018F | ZAC546 | ZB648 | ZH68 |
| *ZmCCT8* | SV1 | AA | AA | AA | NN | AA | NN | AA | AA | AA | NN | AA | AA | AA | AA | AA | AA | NN | AA | AA | AA | AA | AA |
| *ZmCCT29* | SV2 | NN | AA | NN | AA | NN | NN | NN | NN | NN | AA | NN | NN | NN | NN | NN | NN | NN | NN | AA | AA | NN | AA |
|  | SV3 | TT | AA | TT | NN | TT | TT | NN | TT | TT | NN | TT | TT | TT | NN | NN | TT | NN | TT | NN | AA | NN | AA |
| *ZmCCT30* | SV4 | NN | AA | NN | NN | NN | NN | NN | NN | NN | NN | NN | NN | NN | NN | NN | NN | NN | NN | AA | AA | NN | NN |
|  | SV5 | TT | TT | NN | TT | TT | TT | NN | TT | TT | TT | NN | NN | NN | TT | NN | TT | NN | NN | TT | NN | NN | NN |
| *ZmCCT41* | SV6 | NN | NN | AA | NN | NN | NN | NN | NN | TT | AA | NN | NN | NN | NN | NN | AA | NN | NN | AA | NN | NN | NN |
|  | SV7 | TT | NN | NN | TT | TT | NN | TT | TT | NN | TT | NN | TT | TT | NN | NN | TT | TT | NN | TT | TT | TT | TT |
| *ZmCCT43* | SV8 | NN | AA | NN | NN | NN | NN | NN | NN | NN | NN | AA | NN | NN | NN | NN | NN | NN | NN | NN | NN | NN | AA |
|  | SV9 | TT | NN | TT | TT | TT | TT | TT | TT | TT | TT | NN | TT | TT | TT | NN | TT | TT | TT | TT | TT | TT | NN |
|  | SV10 | TT | NN | TT | TT | TT | TT | TT | TT | TT | TT | NN | TT | TT | TT | NN | TT | TT | TT | TT | TT | TT | NN |
|  | SV11 | TT | NN | TT | TT | TT | TT | TT | TT | TT | TT | NN | TT | TT | TT | NN | TT | TT | TT | TT | TT | NN | NN |
|  | SV12 | TT | NN | TT | TT | TT | TT | TT | TT | TT | TT | NN | TT | TT | TT | NN | TT | TT | TT | TT | TT | TT | NN |
|  | SV13 | TT | NN | TT | TT | NN | TT | TT | TT | TT | TT | NN | TT | TT | TT | NN | TT | NN | TT | TT | TT | NN | NN |
|  | SV14 | TT | TT | NN | TT | TT | NN | TT | TT | TT | TT | TT | NN | TT | TT | TT | NN | TT | NN | TT | TT | TT | NN |
|  | SV15 | TT | TT | NN | TT | TT | NN | TT | TT | TT | TT | TT | NN | TT | TT | TT | NN | TT | NN | TT | TT | TT | NN |

| **Table S6** *Continued*  AA, no difference in this locus when compared to B73 genome but difference in when compared to SK genome; NN, unable to judge; SV, structural variation; TT, no difference in this locus when compared to SK genome but difference in when compared to B73 genome. | | | | | | | | | | | | | | | | | | |
| --- | --- | --- | --- | --- | --- | --- | --- | --- | --- | --- | --- | --- | --- | --- | --- | --- | --- | --- |
|  |  | Maize inbred line | | | | | | | | | | | | | | | |  |
| *ZmCCT* | SV | ZHENG22 | ZHENG28 | ZHENG29 | ZHENG30 | ZHENG32 | ZHENG35 | ZHENG58 | ZHENG653 | ZHI41 | ZHONG69 | ZI330 | ZONG3 | ZONG31 | ZZ01 | ZZ03 |  |  |
| *ZmCCT8* | SV1 | AA | AA | AA | AA | AA | AA | AA | AA | AA | AA | NN | TT | NN | NN | AA |  |  |
| *ZmCCT29* | SV2 | NN | NN | NN | NN | NN | NN | NN | NN | NN | NN | NN | NN | NN | NN | NN |  |  |
|  | SV3 | NN | TT | TT | TT | TT | TT | TT | TT | AA | AA | TT | TT | TT | TT | TT |  |  |
| *ZmCCT30* | SV4 | NN | NN | AA | NN | NN | AA | NN | NN | NN | AA | NN | NN | NN | NN | NN |  |  |
|  | SV5 | NN | NN | NN | NN | TT | TT | NN | TT | NN | NN | TT | NN | TT | NN | NN |  |  |
| *ZmCCT41* | SV6 | NN | NN | NN | NN | NN | NN | NN | NN | NN | NN | NN | NN | NN | NN | NN |  |  |
|  | SV7 | TT | TT | TT | TT | NN | TT | NN | TT | TT | NN | NN | TT | TT | NN | TT |  |  |
| *ZmCCT43* | SV8 | NN | NN | NN | NN | NN | NN | NN | NN | NN | NN | NN | NN | NN | NN | NN |  |  |
|  | SV9 | TT | TT | TT | TT | TT | TT | TT | TT | TT | TT | NN | TT | TT | TT | TT |  |  |
|  | SV10 | TT | TT | TT | TT | TT | TT | TT | TT | TT | TT | NN | TT | TT | TT | TT |  |  |
|  | SV11 | TT | NN | TT | TT | TT | TT | TT | TT | TT | TT | NN | TT | TT | TT | TT |  |  |
|  | SV12 | TT | TT | TT | TT | TT | TT | TT | TT | TT | TT | NN | TT | TT | TT | TT |  |  |
|  | SV13 | TT | NN | NN | TT | TT | TT | TT | TT | TT | TT | NN | TT | TT | TT | NN |  |  |
|  | SV14 | NN | TT | NN | NN | TT | TT | TT | TT | TT | TT | TT | NN | TT | TT | TT |  |  |
|  | SV15 | NN | TT | NN | NN | TT | TT | TT | TT | TT | TT | TT | NN | TT | TT | TT |  |  |
