# Supplementary material for: Analyses of open-access multi-omics data sets reveal genetic and expression characteristics of maize ZmCCT family genes
Source: AoB Plants. 2021 Aug 16;13(5):plab048. doi: 10.1093/aobpla/plab048 (PMC8459886; doi:10.1093/aobpla/plab048)
Supplement: plab048_suppl_Supplementary_Table_S7 [file plab048_suppl_supplementary_table_s7.docx]

**Table S7** Genes co-expressed with *ZmCCT*s in maize inbred line B73

*Note*: ZmCCT, Maize CCT domain-containing protein; *ZmCCT*, ZmCCT gene.

| **Gene A** | **Gene B associated with Gene A** | **Mutual rank index** |
| --- | --- | --- |
| GRMZM2G023591 | Zm00001d016072 | 11.45 |
| GRMZM2G023591 | Zm00001d019898 | 11.88 |
| LOC100192616 | Zm00001d026285 | 4.63 |
| LOC100192616 | Zm00001d029865 | 4.39 |
| LOC100274515 | Zm00001d029898 bHLH | 9.6 |
| LOC100274515 | Zm00001d049883 | 4.51 |
| LOC100281451 | Zm00001d011735 | 38.15 |
| LOC100281451 | Zm00001d043735 | 41.12 |
| LOC100283508 | LOC103646821 | 18.33 |
| LOC100283508 | Zm00001d041648 | 19.98 |
| LOC100502179 | Zm00001d052620 | 16.64 |
| LOC100502179 | Zm00001d053559 | 32.69 |
| LOC103636146 | Zm00001d041232 | 3.95 |
| LOC103644139 | Zm00001d029898 bHLH | 17.39 |
| Zm00001d000401 | Zm00001d037420 | 7.38 |
| Zm00001d002532 | *ZmCCT9* | 12.46 |
| Zm00001d002545 | *ZmCCT51* | 2.1 |
| Zm00001d002545 | Zm00001d027936 | 7.71 |
| Zm00001d002550 | *ZmCCT9* | 4.37 |
| Zm00001d002550 | Zm00001d036489 | 1.59 |
| Zm00001d002550 | Zm00001d039194 | 7.23 |
| Zm00001d002758 | Zm00001d007445 | 18.71 |
| Zm00001d002758 | Zm00001d045340 | 62.32 |
| Zm00001d002759 | *ZmCCT41* | 34.06 |
| Zm00001d002759 | Zm00001d039530 | 18.12 |
| Zm00001d002759 | Zm00001d043573 | 27.82 |
| Zm00001d002856 | Zm00001d026632 | 5.1 |
| Zm00001d002856 | Zm00001d043573 | 3.92 |
| Zm00001d003195 | Zm00001d027846 Dof | 4.4 |
| Zm00001d003195 | *ZmCCT9* | 4.17 |
| Zm00001d003767 | Zm00001d041819 | 1.95 |
| Zm00001d003931 | Zm00001d005329 | 10.89 |
| Zm00001d004006 | Zm00001d043573 | 5.85 |
| Zm00001d004006 | Zm00001d050043 | 5.16 |
| Zm00001d004301 | LOC103644139 | 7.52 |
| Zm00001d004301 | Zm00001d039314 | 10.02 |
| Zm00001d004705 | Zm00001d033797 | 6.75 |
| Zm00001d004716 | Zm00001d018318 | 13.51 |
| Zm00001d004716 | Zm00001d051545 | 12.51 |
| Zm00001d004716 | Zm00001d053076 | 4.41 |
| Zm00001d005051 | *ZmCCT52* | 6.98 |
| Zm00001d005051 | Zm00001d017147 | 9.02 |
| Zm00001d005099 | Zm00001d032933 | 6.35 |
| Zm00001d005099 | Zm00001d044903 | 7.62 |
| Zm00001d005329 | LOC100192616 | 4.93 |
| Zm00001d005446 | Zm00001d041819 | 2.14 |
| Zm00001d006026 | Zm00001d011785 G2-like | 2.46 |
| Zm00001d006026 | *ZmCCT33* | 5.45 |
| Zm00001d006026 | Zm00001d029424 | 2.5 |
| Zm00001d006059 | Zm00001d021006 | 1.78 |
| Zm00001d006587 | Zm00001d003767 | 9.3 |
| Zm00001d006587 | Zm00001d019518 | 8.88 |
| Zm00001d006587 | Zm00001d021763 | 4.97 |
| Zm00001d006603 | Zm00001d038532 | 10.76 |
| Zm00001d006881 | *ZmCCT33* | 4.44 |
| Zm00001d006881 | Zm00001d027936 | 6.94 |
| Zm00001d006881 | Zm00001d039156 | 15.11 |
| Zm00001d008764 | *ZmCCT31* | 79.42 |
| Zm00001d008764 | *ZmCCT9* | 64.89 |
| Zm00001d008764 | Zm00001d039643 | 4.69 |
| Zm00001d008826 | Zm00001d030995 bZIP | 6.68 |
| Zm00001d008826 | *ZmCCT11* | 7.64 |
| Zm00001d008826 | Zm00001d039589 | 1 |
| Zm00001d009017 Nin-like | Zm00001d022388 | 6.23 |
| Zm00001d009481 | Zm00001d018318 | 34.55 |
| Zm00001d009481 | Zm00001d039589 | 13.82 |
| Zm00001d010627 | Zm00001d002532 | 27.58 |
| Zm00001d010627 | Zm00001d017041 | 34.59 |
| Zm00001d010871 | Zm00001d015385 | 11.96 |
| Zm00001d010871 | Zm00001d021763 | 8.28 |
| Zm00001d010871 | Zm00001d046786 | 9.76 |
| Zm00001d011073 | Zm00001d019518 | 20.21 |
| Zm00001d011073 | Zm00001d041819 | 26.38 |
| Zm00001d011735 | Zm00001d004705 | 12.76 |
| Zm00001d011735 | Zm00001d033797 | 13.62 |
| Zm00001d011758 | Zm00001d043573 | 3.92 |
| Zm00001d011758 | Zm00001d047555 | 10.65 |
| Zm00001d011785 G2-like | *ZmCCT33* | 2.19 |
| Zm00001d012322 | Zm00001d032458 | 4.72 |
| Zm00001d013406 | Zm00001d004705 | 8.18 |
| Zm00001d014109 | Zm00001d006026 | 18.66 |
| Zm00001d014109 | Zm00001d027936 | 20.31 |
| Zm00001d014193 | Zm00001d029898 bHLH | 3.29 |
| Zm00001d014193 | *ZmCCT3* | 8.06 |
| Zm00001d014193 | Zm00001d014990 | 8.06 |
| Zm00001d014317 | Zm00001d037150 | 7.29 |
| Zm00001d014317 | Zm00001d047618 | 2.51 |
| Zm00001d014600 | Zm00001d015385 | 11.95 |
| Zm00001d014600 | Zm00001d046786 | 11.51 |
| Zm00001d014755 | Zm00001d014317 | 25.2 |
| Zm00001d014755 | Zm00001d028806 | 7.84 |
| Zm00001d014811 | LOC100192616 | 13.43 |
| Zm00001d014811 | Zm00001d005329 | 16.94 |
| Zm00001d014811 | Zm00001d038747 | 7.8 |
| Zm00001d015307 | Zm00001d028751 | 6.84 |
| Zm00001d015307 | Zm00001d032776 | 5.34 |
| Zm00001d015385 | Zm00001d021763 | 1.5 |
| Zm00001d015385 | Zm00001d046786 | 1.61 |
| Zm00001d015477 | Zm00001d031636 | 5.31 |
| Zm00001d015477 | Zm00001d038532 | 7.35 |
| Zm00001d015743 bZIP | *ZmCCT58* | 7.71 |
| Zm00001d015743 bZIP | Zm00001d014990 | 4.5 |
| Zm00001d016361 GATA | Zm00001d044785 G2-like | 5.93 |
| Zm00001d016361 GATA | Zm00001d038683 TCP | 13.39 |
| Zm00001d016674 HSF | Zm00001d007445 | 11.87 |
| Zm00001d016698 | Zm00001d015743 bZIP | 4.93 |
| Zm00001d016698 | *ZmCCT3* | 9.58 |
| Zm00001d016698 | Zm00001d014990 | 3.19 |
| Zm00001d016731 | Zm00001d018318 | 6.54 |
| Zm00001d016731 | Zm00001d053076 | 4.81 |
| Zm00001d016817 | *ZmCCT9* | 19.15 |
| Zm00001d016817 | Zm00001d050918 | 19.05 |
| Zm00001d016898 | *ZmCCT9* | 21.35 |
| Zm00001d016898 | Zm00001d017147 | 15.73 |
| Zm00001d016941 | Zm00001d000401 | 17.14 |
| Zm00001d016941 | Zm00001d008826 | 11.33 |
| Zm00001d016941 | Zm00001d050043 | 16.17 |
| Zm00001d016991 | Zm00001d005446 | 4.73 |
| Zm00001d016991 | Zm00001d019518 | 4.54 |
| Zm00001d017041 | Zm00001d041232 | 15.44 |
| Zm00001d017041 | Zm00001d050918 | 2.42 |
| Zm00001d017379 | *ZmCCT9* | 32.46 |
| Zm00001d017379 | Zm00001d003195 | 33.35 |
| Zm00001d017412 DBB | *ZmCCT38* | 110.6 |
| Zm00001d017412 DBB | Zm00001d011073 | 57.51 |
| Zm00001d017941 | Zm00001d044136 | 7.15 |
| Zm00001d018318 | Zm00001d053076 | 7.33 |
| Zm00001d018393 | Zm00001d006059 | 12.19 |
| Zm00001d018393 | Zm00001d021773 | 7.64 |
| Zm00001d018393 | Zm00001d049016 | 6.26 |
| Zm00001d018416 bHLH | Zm00001d024701 HD-ZIP | 9.2 |
| Zm00001d018416 bHLH | Zm00001d026036 | 2.02 |
| Zm00001d018718 | Zm00001d045528 | 21.12 |
| Zm00001d018781 | *ZmCCT9* | 2.75 |
| Zm00001d018781 | Zm00001d046440 | 18.57 |
| Zm00001d018781 | Zm00001d051018 | 25.36 |
| Zm00001d019518 | Zm00001d003767 | 2.69 |
| Zm00001d019518 | Zm00001d005446 | 3.77 |
| Zm00001d019518 | Zm00001d041819 | 2.97 |
| Zm00001d019898 | Zm00001d016072 | 2.25 |
| Zm00001d019898 | Zm00001d052216 | 2.94 |
| Zm00001d019956 | Zm00001d019898 | 5.91 |
| Zm00001d020396 | Zm00001d039156 | 4.11 |
| Zm00001d020877 | Zm00001d015385 | 5.71 |
| Zm00001d020877 | Zm00001d046786 | 6.89 |
| Zm00001d021416 | *ZmCCT57* | 6.19 |
| Zm00001d021416 | Zm00001d043786 | 7.24 |
| Zm00001d021763 | Zm00001d046786 | 3.84 |
| Zm00001d021773 | Zm00001d006059 | 4.21 |
| Zm00001d021773 | Zm00001d029438 | 4.18 |
| Zm00001d021906 | Zm00001d003767 | 5.19 |
| Zm00001d021906 | Zm00001d041819 | 5.08 |
| Zm00001d021906 | Zm00001d046786 | 6.42 |
| Zm00001d021951 | Zm00001d031636 | 11.08 |
| Zm00001d022082 | Zm00001d023931 MYB related | 5.02 |
| Zm00001d022082 | Zm00001d018618 | 5.45 |
| Zm00001d022082 | Zm00001d039946 | 5.63 |
| Zm00001d022326 | *ZmCCT9* | 44.43 |
| Zm00001d023314 | *ZmCCT52* | 15.55 |
| Zm00001d023314 | *ZmCCT9* | 7.67 |
| Zm00001d023314 | Zm00001d017147 | 12.83 |
| Zm00001d023931 MYB related | Zm00001d018618 | 7.12 |
| Zm00001d024534 C3H | Zm00001d018718 | 7.48 |
| Zm00001d024534 C3H | Zm00001d045528 | 3.88 |
| Zm00001d024591 | Zm00001d024534 C3H | 27.62 |
| Zm00001d024591 | Zm00001d049557 | 23.88 |
| Zm00001d025720 Trihelix | Zm00001d026036 | 13.73 |
| Zm00001d026017 MYB related | *ZmCCT34* | 22.95 |
| Zm00001d026017 MYB related | *ZmCCT51* | 16.56 |
| Zm00001d026017 MYB related | Zm00001d002545 | 1.63 |
| Zm00001d026285 | Zm00001d005329 | 2.27 |
| Zm00001d026401 | Zm00001d046096 bHLH | 1.15 |
| Zm00001d026632 | Zm00001d006059 | 4.14 |
| Zm00001d026632 | Zm00001d021006 | 3.11 |
| Zm00001d027292 GRAS | LOC103636146 | 4.96 |
| Zm00001d027292 GRAS | Zm00001d031201 | 2.29 |
| Zm00001d027292 GRAS | Zm00001d041232 | 4.55 |
| Zm00001d027936 | *ZmCCT33* | 2.24 |
| Zm00001d028011 | Zm00001d042560 LBD | 10.07 |
| Zm00001d028011 | Zm00001d017941 | 1.6 |
| Zm00001d028011 | Zm00001d044136 | 9.39 |
| Zm00001d028751 | Zm00001d032776 | 1.65 |
| Zm00001d028806 | Zm00001d047618 | 22.77 |
| Zm00001d028820 | Zm00001d007445 | 9.98 |
| Zm00001d028820 | Zm00001d053091 | 11.78 |
| Zm00001d029027 | Zm00001d019898 | 4.16 |
| Zm00001d029027 | Zm00001d052216 | 6.1 |
| Zm00001d029102 | Zm00001d001837 MYB | 4.31 |
| Zm00001d029102 | Zm00001d047159 | 5.84 |
| Zm00001d029201 | Zm00001d019898 | 9.59 |
| Zm00001d029201 | Zm00001d019956 | 10.61 |
| Zm00001d029258 | Zm00001d003209 | 9.17 |
| Zm00001d029258 | Zm00001d038971 | 15.63 |
| Zm00001d029424 | *ZmCCT51* | 1.99 |
| Zm00001d029424 | Zm00001d030550 | 37.42 |
| Zm00001d029438 | Zm00001d006059 | 5.66 |
| Zm00001d029570 | *ZmCCT3* | 2.08 |
| Zm00001d029570 | *ZmCCT58* | 3.13 |
| Zm00001d029570 | Zm00001d053327 | 6.81 |
| Zm00001d029761 | *ZmCCT58* | 12.95 |
| Zm00001d029761 | Zm00001d029570 | 32.19 |
| Zm00001d029761 | Zm00001d043786 | 30.84 |
| Zm00001d029865 | *ZmCCT2* | 14.65 |
| Zm00001d029865 | Zm00001d005329 | 15.63 |
| Zm00001d029898 bHLH | Zm00001d046402 bZIP | 4.5 |
| Zm00001d029898 bHLH | *ZmCCT58* | 5.81 |
| Zm00001d030220 | Zm00001d003209 | 6.65 |
| Zm00001d030220 | Zm00001d003931 | 7.64 |
| Zm00001d030468 | LOC103646821 | 13.88 |
| Zm00001d030468 | Zm00001d036841 | 13.11 |
| Zm00001d030468 | Zm00001d041402 | 19.14 |
| Zm00001d030550 | Zm00001d011785 G2-like | 3.29 |
| Zm00001d030550 | *ZmCCT33* | 3.02 |
| Zm00001d030550 | Zm00001d027936 | 3.1 |
| Zm00001d030678 MYB | *ZmCCT45* | 20.63 |
| Zm00001d030678 MYB | Zm00001d037150 | 32.73 |
| Zm00001d030995 bZIP | Zm00001d000401 | 2.08 |
| Zm00001d030995 bZIP | Zm00001d007445 | 6.35 |
| Zm00001d031201 | Zm00001d041232 | 2.13 |
| Zm00001d031636 | Zm00001d006603 | 2.26 |
| Zm00001d031636 | Zm00001d038532 | 3.34 |
| Zm00001d031703 | Zm00001d009695 | 5.23 |
| Zm00001d031703 | Zm00001d049656 | 13.64 |
| Zm00001d032197 | Zm00001d003767 | 11.11 |
| Zm00001d032197 | Zm00001d015385 | 6.13 |
| Zm00001d032197 | Zm00001d046786 | 4.16 |
| Zm00001d032810 | Zm00001d020396 | 6.87 |
| Zm00001d032810 | Zm00001d026632 | 5.02 |
| Zm00001d032810 | Zm00001d039156 | 7.43 |
| Zm00001d032828 | *ZmCCT58* | 27.77 |
| Zm00001d032828 | Zm00001d029570 | 18.03 |
| Zm00001d032933 | Zm00001d043592 | 4.96 |
| Zm00001d033150 | Zm00001d003767 | 14.01 |
| Zm00001d033150 | Zm00001d019518 | 15.34 |
| Zm00001d033196 | LOC103646821 | 6 |
| Zm00001d033339 | Zm00001d011785 G2-like | 3.99 |
| Zm00001d033339 | *ZmCCT33* | 16.84 |
| Zm00001d033339 | Zm00001d030550 | 26.62 |
| Zm00001d033797 | LOC100192616 | 6.51 |
| Zm00001d033797 | Zm00001d013406 | 1.31 |
| Zm00001d034067 | *ZmCCT9* | 60.63 |
| Zm00001d034067 | Zm00001d021416 | 89.49 |
| Zm00001d034067 | Zm00001d022326 | 73.22 |
| Zm00001d035634 | Zm00001d005329 | 38.28 |
| Zm00001d035634 | Zm00001d051225 | 39.65 |
| Zm00001d035907 TALE | Zm00001d014193 | 8.4 |
| Zm00001d036035 | Zm00001d049557 | 21.82 |
| Zm00001d036489 | *ZmCCT9* | 3.38 |
| Zm00001d036489 | Zm00001d039194 | 4.46 |
| Zm00001d036535 | Zm00001d015385 | 5.06 |
| Zm00001d036535 | Zm00001d046786 | 7.54 |
| Zm00001d036597 | Zm00001d036035 | 4.35 |
| Zm00001d036841 | LOC103646821 | 2.61 |
| Zm00001d036841 | Zm00001d033196 | 5.46 |
| Zm00001d036841 | Zm00001d041648 | 4.97 |
| Zm00001d037420 | Zm00001d039589 | 7.64 |
| Zm00001d038490 | LOC100283508 | 83.96 |
| Zm00001d038490 | Zm00001d041402 | 86.54 |
| Zm00001d038490 | Zm00001d041648 | 57.47 |
| Zm00001d038683 TCP | Zm00001d046354 GATA | 6.28 |
| Zm00001d038747 | Zm00001d034255 | 5.43 |
| Zm00001d038747 | Zm00001d051545 | 5.1 |
| Zm00001d038764 | *ZmCCT2* | 11.71 |
| Zm00001d038764 | Zm00001d012322 | 9.9 |
| Zm00001d038764 | Zm00001d032458 | 3.94 |
| Zm00001d038925 | Zm00001d039589 | 17.52 |
| Zm00001d038971 | Zm00001d003209 | 15.64 |
| Zm00001d039156 | Zm00001d026632 | 9.48 |
| Zm00001d039194 | Zm00001d005051 | 11.67 |
| Zm00001d039197 | *ZmCCT57* | 9.8 |
| Zm00001d039197 | *ZmCCT9* | 18.99 |
| Zm00001d039197 | Zm00001d039270 | 17.13 |
| Zm00001d039270 | *ZmCCT57* | 21.84 |
| Zm00001d039314 | *ZmCCT3* | 6.93 |
| Zm00001d039530 | Zm00001d043573 | 24.03 |
| Zm00001d039530 | Zm00001d050043 | 14.06 |
| Zm00001d039589 | *ZmCCT12* | 2.3 |
| Zm00001d039589 | Zm00001d007445 | 3.74 |
| Zm00001d039643 | Zm00001d011735 | 16.17 |
| Zm00001d039946 | Zm00001d018618 | 5.83 |
| Zm00001d041402 | LOC103646821 | 5.36 |
| Zm00001d041402 | Zm00001d033196 | 9.56 |
| Zm00001d041402 | Zm00001d036841 | 5.56 |
| Zm00001d041648 | LOC103646821 | 5.51 |
| Zm00001d043031 | Zm00001d017941 | 21.71 |
| Zm00001d043031 | Zm00001d044136 | 37.77 |
| Zm00001d043201 | Zm00001d016072 | 7.28 |
| Zm00001d043201 | Zm00001d019898 | 3.98 |
| Zm00001d043389 | Zm00001d046530 | 27.48 |
| Zm00001d043536 HSF | Zm00001d011785 G2-like | 13.55 |
| Zm00001d043536 HSF | Zm00001d038925 | 21.08 |
| Zm00001d043573 | Zm00001d050043 | 1.97 |
| Zm00001d043735 | Zm00001d011735 | 23.14 |
| Zm00001d043735 | Zm00001d033797 | 28.79 |
| Zm00001d043786 | Zm00001d002550 | 10.83 |
| Zm00001d044053 | Zm00001d015307 | 53.52 |
| Zm00001d044053 | Zm00001d022082 | 62.43 |
| Zm00001d044136 | Zm00001d024701 HD-ZIP | 9.27 |
| Zm00001d044136 | Zm00001d042560 LBD | 2.7 |
| Zm00001d044182 | Zm00001d026401 | 19.21 |
| Zm00001d044903 | Zm00001d032933 | 4.27 |
| Zm00001d044903 | Zm00001d043592 | 5.23 |
| Zm00001d045340 | Zm00001d027936 | 8.1 |
| Zm00001d045340 | Zm00001d037420 | 11.25 |
| Zm00001d046096 bHLH | Zm00001d044182 | 2.82 |
| Zm00001d046354 GATA | Zm00001d044785 G2-like | 5.65 |
| Zm00001d046354 GATA | Zm00001d016361 GATA | 5.76 |
| Zm00001d046354 GATA | *ZmCCT30* | 2.21 |
| Zm00001d046402 bZIP | *ZmCCT58* | 7.87 |
| Zm00001d046402 bZIP | Zm00001d014990 | 8.62 |
| Zm00001d046440 | *ZmCCT9* | 15.37 |
| Zm00001d046786 | Zm00001d003767 | 3.26 |
| Zm00001d046786 | Zm00001d041819 | 2.23 |
| Zm00001d047159 | Zm00001d001837 MYB | 22.55 |
| Zm00001d047159 | Zm00001d021006 | 19.88 |
| Zm00001d047555 | Zm00001d021006 | 3.54 |
| Zm00001d047555 | Zm00001d043573 | 5.77 |
| Zm00001d048246 | Zm00001d006026 | 8.29 |
| Zm00001d048246 | Zm00001d027936 | 2.46 |
| Zm00001d048246 | Zm00001d030550 | 6.66 |
| Zm00001d048397 | LOC103639552 | 25.07 |
| Zm00001d048414 | LOC100192616 | 8.77 |
| Zm00001d048414 | Zm00001d005329 | 8.88 |
| Zm00001d049016 | Zm00001d006059 | 2.62 |
| Zm00001d049016 | Zm00001d021773 | 3.21 |
| Zm00001d049016 | Zm00001d029438 | 1.72 |
| Zm00001d049557 | Zm00001d036597 | 13.88 |
| Zm00001d049650 | Zm00001d015385 | 14.25 |
| Zm00001d049650 | Zm00001d016991 | 14.22 |
| Zm00001d049650 | Zm00001d019518 | 12.93 |
| Zm00001d049656 | Zm00001d009695 | 7.43 |
| Zm00001d049834 | Zm00001d031636 | 21.99 |
| Zm00001d049834 | Zm00001d038532 | 17.81 |
| Zm00001d049883 | Zm00001d035907 TALE | 17.14 |
| Zm00001d049883 | *ZmCCT58* | 10.93 |
| Zm00001d050918 | Zm00001d041232 | 9 |
| Zm00001d051018 | Zm00001d027846 Dof | 8.92 |
| Zm00001d051018 | *ZmCCT52* | 2.28 |
| Zm00001d051018 | Zm00001d017147 | 2.99 |
| Zm00001d051225 | Zm00001d003931 | 26.15 |
| Zm00001d051225 | Zm00001d005329 | 36.74 |
| Zm00001d051321 | Zm00001d022388 | 5.05 |
| Zm00001d051321 | Zm00001d053076 | 1.74 |
| Zm00001d051440 | Zm00001d007445 | 5.53 |
| Zm00001d051440 | Zm00001d028820 | 16.02 |
| Zm00001d051440 | Zm00001d053091 | 16.33 |
| Zm00001d051545 | Zm00001d053076 | 3.67 |
| Zm00001d052630 | Zm00001d019898 | 6.78 |
| Zm00001d052630 | Zm00001d019956 | 13.42 |
| Zm00001d053091 | Zm00001d016674 HSF | 2.55 |
| Zm00001d053091 | Zm00001d007445 | 1.26 |
| Zm00001d053091 | Zm00001d039589 | 6.31 |
| Zm00001d053327 | Zm00001d029898 bHLH | 6.52 |
| Zm00001d053391 bHLH | Zm00001d046096 bHLH | 2.87 |
| Zm00001d053391 bHLH | Zm00001d026401 | 9.91 |
| Zm00001d053559 | *ZmCCT43* | 11.89 |
| Zm00001d053559 | Zm00001d052620 | 7.94 |
| *ZmCCT11* | *ZmCCT23* | 4.36 |
| *ZmCCT11* | Zm00001d007445 | 4.44 |
| *ZmCCT11* | Zm00001d037420 | 4.32 |
| *ZmCCT12* | Zm00001d007445 | 6.04 |
| *ZmCCT13* | Zm00001d038532 | 18.02 |
| *ZmCCT13* | Zm00001d044136 | 15 |
| *ZmCCT15* | Zm00001d027292 GRAS | 23.19 |
| *ZmCCT18* | Zm00001d010627 | 49.24 |
| *ZmCCT18* | Zm00001d043735 | 37.03 |
| *ZmCCT2* | Zm00001d005329 | 16.05 |
| *ZmCCT21* | Zm00001d011785 G2-like | 24.09 |
| *ZmCCT21* | *ZmCCT33* | 1.43 |
| *ZmCCT21* | Zm00001d006026 | 10.79 |
| *ZmCCT23* | *ZmCCT41* | 11.17 |
| *ZmCCT26* | *ZmCCT35* | 9.33 |
| *ZmCCT26* | *ZmCCT8* | 5.37 |
| *ZmCCT26* | Zm00001d046786 | 7.79 |
| *ZmCCT3* | Zm00001d029898 bHLH | 6.57 |
| *ZmCCT3* | *ZmCCT58* | 1.8 |
| *ZmCCT30* | Zm00001d019898 | 12.91 |
| *ZmCCT31* | *ZmCCT58* | 31.68 |
| *ZmCCT32* | Zm00001d021773 | 91.81 |
| *ZmCCT32* | Zm00001d021951 | 85.18 |
| *ZmCCT32* | Zm00001d029102 | 64.7 |
| *ZmCCT34* | Zm00001d038925 | 12.22 |
| *ZmCCT34* | Zm00001d039156 | 14.38 |
| *ZmCCT34* | Zm00001d043573 | 22.44 |
| *ZmCCT37* | Zm00001d031703 | 56.87 |
| *ZmCCT37* | Zm00001d045528 | 44.57 |
| *ZmCCT37* | Zm00001d049557 | 45.6 |
| *ZmCCT38* | *ZmCCT35* | 5.03 |
| *ZmCCT38* | Zm00001d019518 | 28.56 |
| *ZmCCT40* | Zm00001d025720 Trihelix | 146.8 |
| *ZmCCT40* | *ZmCCT45* | 25.28 |
| *ZmCCT41* | *ZmCCT12* | 1.4 |
| *ZmCCT41* | Zm00001d034255 | 9.36 |
| *ZmCCT41* | Zm00001d039589 | 4.41 |
| *ZmCCT43* | Zm00001d044903 | 5.79 |
| *ZmCCT45* | Zm00001d043389 | 14.02 |
| *ZmCCT45* | Zm00001d046530 | 11.08 |
| *ZmCCT5* | *ZmCCT33* | 46.91 |
| *ZmCCT5* | Zm00001d007445 | 17.51 |
| *ZmCCT51* | Zm00001d014109 | 14.34 |
| *ZmCCT52* | *ZmCCT31* | 5.05 |
| *ZmCCT52* | Zm00001d017147 | 1.93 |
| *ZmCCT53* | Zm00001d009017 Nin-like | 6.9 |
| *ZmCCT53* | Zm00001d053076 | 8.57 |
| *ZmCCT53* | Zm00001d053559 | 13.71 |
| *ZmCCT54* | LOC103639552 | 14.46 |
| *ZmCCT55* | *ZmCCT18* | 9.19 |
| *ZmCCT55* | Zm00001d014317 | 26.87 |
| *ZmCCT55* | Zm00001d038971 | 27.37 |
| *ZmCCT57* | *ZmCCT9* | 3.91 |
| *ZmCCT6* | Zm00001d030468 | 183.12 |
| *ZmCCT6* | Zm00001d038490 | 108.65 |
| *ZmCCT6* | Zm00001d044053 | 155.61 |
| *ZmCCT8* | Zm00001d041819 | 25.65 |
